# Atypical Condensation Domains Guide Discovery and Illuminate Biosynthesis of Myxoglucamides Featuring Vinyl‐Substituted, α‐Oxidized γ‐Amino Acids

**DOI:** 10.1002/anie.6995139

**Published:** 2026-08-07

**Authors:** Tingting Wang, Alexander Popoff, Maja Hunter, Guangzhi Dai, Ronald Garcia, Huixin Xu, Haowen Zhao, Asfandyar Sikandar, Liujie Huo, Rolf Müller, Chengzhang Fu

**Affiliations:** ^1^ Helmholtz Institute for Pharmaceutical Research Saarland (HIPS) Helmholtz Centre for Infection Research (HZI) and Department of Pharmacy Saarland University Saarbrücken Germany; ^2^ Helmholtz International Lab for Anti‐Infectives Helmholtz Centre for Infection Research Braunschweig Germany; ^3^ Helmholtz International Laboratory State Key Laboratory of Microbial Technology Shandong University Qingdao P. R. China; ^4^ Department of Pharmaceutical and Medicinal Chemistry Institute of Pharmacy Paracelsus Medical University Salzburg Austria; ^5^ School of Pharmaceutical Sciences and Institute of Materia Medica Shandong First Medical University Jinan P. R. China; ^6^ German Centre for Infection Research (DZIF) Partner Site Hannover Braunschweig Germany

**Keywords:** condensation domains, glycolipopeptides, *O*‐acylation, β‐hydroxylation, γ‐amino acids

## Abstract

Condensation (C) domains in nonribosomal peptide synthetase (NRPS) pathways exhibit versatile functions that drive biosynthetic and chemical novelty. Through genome mining for atypical C domains, we identified a hybrid NRPS/polyketide synthase (PKS) biosynthetic gene cluster (*mxg*) from *Cystobacterineae* sp. MCy9003 and discovered myxoglucamides, a family of glycolipopeptides featuring an unprecedented vinyl‐substituted γ‐amino acid bearing an α‐hydroxy/α‐ketoamide functionality. Heterologous expression of the promoter‐refactored pathway revealed new *O*‐acylated myxoglucamides, and subsequent studies unveiled the C domain‐like enzyme MxgH as a promiscuous *O*‐acyltransferase decorating the glucose moiety with short‐chain acyl groups. Biosynthetic investigations demonstrated that the unusual γ‐amino acid originates from l‐glutamate. Completion of the cryptic β‐hydroxylation of peptidyl carrier protein‐tethered glutamate by the α‐ketoglutarate‐dependent dioxygenase Ox_MxgA_ occurs only concomitantly with upstream chain extension, revealing a bidirectional checkpoint for substrate fidelity. Unexpectedly, the C‐domain‐like interface domain I_MxgB_ is dispensable for this coupled transformation. Mutational analysis of the FMN‐dependent monooxygenase encoded by *mxgE*, together with characterization of a shunt metabolite, supported its role in α‐oxidation for α‐hydroxy/α‐ketoamide formation during γ‐amino acid assembly. Together, these findings uncover an unrecognized biosynthetic logic for generating vinyl‐substituted, α‐oxidized γ‐amino acids and substantially expand the functional repertoire of NRPS/PKS assembly lines.

## Graphical abstract

Genome mining of atypical condensation (C) domains leads to the discovery of myxoglucamides, glycolipopeptides featuring an unprecedented vinyl‐substituted, α‐oxidized γ‐amino acid. Biosynthetic studies reveal a discrete C‐domain‐like *O*‐acyltransferase, a β‐hydroxylation‐dependent chain‐extension checkpoint, and an unusual pathway for γ‐amino acid assembly that expands the functional repertoire of NRPS/PKS systems.

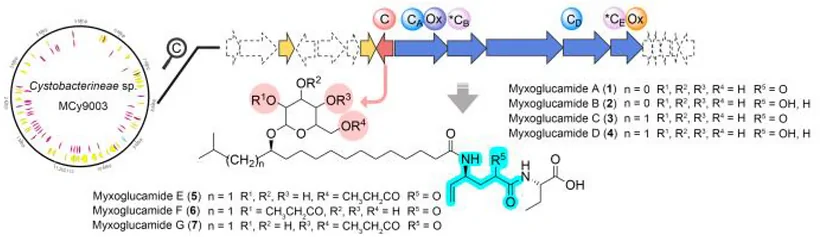

## 1 Introduction

Myxobacteria are soil‐dwelling gram‐negative bacteria distinguished by their social behaviors and complex life cycles, exemplified by a “wolf‐pack” predation mode that employs diverse bioactive secondary metabolites to lyse and consume other microorganisms [1]. Over the past decades, numerous myxobacterial natural products with remarkable chemical diversity and broad bioactivities have been discovered [2]. Nevertheless, bacterial natural products accessed to date remain limited relative to their immense biosynthetic genomic potential [3]. Myxobacteria are among the underexplored bacterial taxa, harboring an exceptional abundance of natural product biosynthetic gene clusters (BGCs), most of which remain silent under laboratory conditions. This disparity underscores the critical importance of genome mining and pathway activation strategies for natural product discovery [4, 5]. Some of the most successful genome‐guided activation approaches include heterologous expression and promoter refactoring of BGCs, as well as noncanonical methods such as ACTIMOT to mobilize and amplify the targeted BGC [6, 7, 8, 9].

Nonribosomal peptides (NRPs) and polyketides (PKs) represent major classes of natural products with diverse architectures and significant pharmaceutical potential. Their biosynthesis relies on modular, template‐directed assembly lines that operate via covalently tethered intermediates [10]. While ketosynthase (KS) domains catalyze chain elongation in PK biosynthesis, condensation (C) domains mediate peptide bond formation in NRPS pathways. Beyond canonical amide bond formation, C domains have increasingly been associated with a wide range of noncanonical functions, including ester formation, β‐lactam formation, dehydration, hydrolysis, and enzyme recruitment [11]. This emerging functional diversity positions C domains as key drivers of structural diversification and biosynthetic innovation in NRPS‐derived natural products.

Incorporation of unusual building blocks, particularly non‐proteinogenic amino acids, greatly expands peptide structural diversity and often underpins biological activity. Among these, γ‐amino acids and α‐ketoamides are especially noteworthy, as they introduce distinctive reactivity and conformational features into peptide scaffolds and are therefore regarded as privileged functionalities in bioactive natural products, as well as attractive targets for drug design, non‐natural peptide synthesis, and chemical biology applications [12, 13].

In this study, we report the discovery of a new family of glycolipopeptides, termed myxoglucamides, featuring unprecedented γ‐amino acid residues. Their production was revealed through activation of a cryptic NRPS/PKS hybrid BGC (*mxg*) from *Cystobacterineae* sp. MCy9003 [14], which was prioritized by genome mining based on the presence of multiple atypical C‐domain‐like elements of unknown function. Guided by this unusual domain architecture, we uncover unexpected, mechanistically diverse roles of C domains in myxoglucamide biosynthesis, revealing promiscuous *O*‐acylation of the glucose moiety and a previously unrecognized enzymatic logic for assembling highly oxidized γ‐amino acid scaffolds in NRPS/PKS assembly lines. Notably, complementary total synthesis and chemical biology studies building on the compounds identified here have demonstrated that myxoglucamide A engages defined human protein targets, underscoring that the biosynthetic innovations described in this work give rise to biologically meaningful chemical space [15].

## 2 Results and Discussion

### 2.1 Identification of One NRPS/PKS BGC Harboring Noncanonical C Domains

Recent large‐scale bioinformatic analyses have identified myxobacteria as one of the most abundant yet underexplored sources of BGCs [3]. Consistent with this view, our previous metabolomic analysis of ∼2300 *Myxococcales* strains demonstrated that the likelihood of discovering novel metabolites increases substantially when phylogenetically distant strains are examined [16]. This trend is further supported by recent discoveries of unusual natural products from members of the *Pendulisporaceae*, a newly described BGC‐rich myxobacterial family [17].

Guided by these insights, we investigated the myxobacterial strain *Cystobacterineae* sp. MCy9003, a phylogenetically distant strain that likely represents a novel genus [14]. To prioritize BGCs with the potential to encode unconventional chemical scaffolds, we systematically analyzed the MCy9003 genome using antiSMASH [18] and NaPDoS [19], with particular emphasis on noncanonical C domains, given their emerging roles in driving biosynthetic innovation. This analysis led to the identification of a cryptic NRPS/PKS hybrid BGC, designated *mxg* (GenBank accession number: OR000721).

The *mxg* locus comprises an operon (*mxgA*‐*E*) encoding a hybrid assembly line, including four multidomain NRPS proteins (MxgA, MxgB, MxgD, and MxgE) and one NRPS/PKS hybrid enzyme (MxgC). This BGC was prioritized because it encodes three noncanonical C‐domain‐like elements (Figure 1a). Canonical C domains are typically defined by the conserved HHxxxDG motif, in which the second histidine is generally considered critical for peptide bond formation, although its precise catalytic role remains debated [20]. In contrast, MxgB harbors a C domain lacking this motif and clusters within the interface domain group based on phylogenetic analysis (Figure 1b). MxgE represents an unusual didomain protein comprising an N‐terminal C domain lacking the second conserved histidine, which groups within the dual C domain clade and is fused to a C‐terminal FMN‐dependent monooxygenase domain. Finally, MxgH is a discrete C‐domain‐like protein that forms a distinct clade in the C‐domain phylogenetic tree. The pathway exhibits several other unusual features, including an α‐ketoglutarate (α‐KG)‐dependent dioxygenase domain located at the C‐terminus of MxgA, a cytochrome P450 (MxgG), a reductase (Orf5), and two glycosyltransferases (MxgF and Orf10). Collectively, the enrichment of noncanonical C domains within this otherwise unconventional enzymatic context suggests that *mxg* encodes an unusual biosynthetic logic, motivating its prioritization for experimental activation and chemical characterization (Figure 1a, Table S1).

**FIGURE 1:**
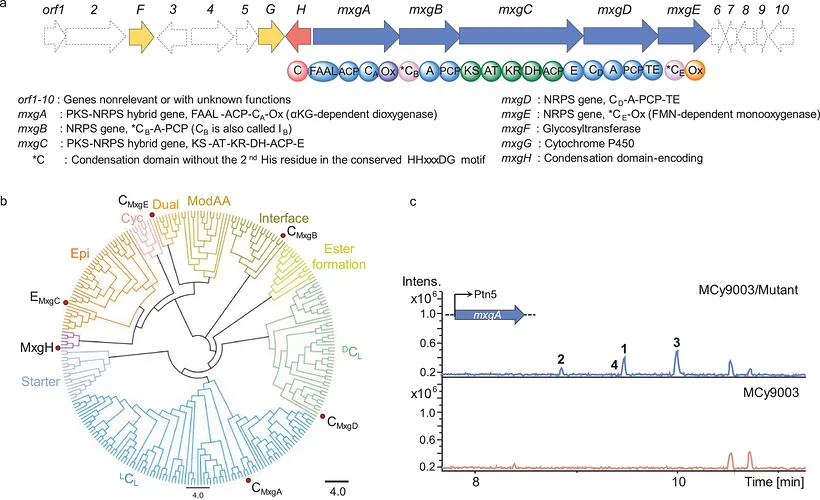
Identification and activation of the myxoglucamide biosynthetic gene cluster (*mxg*) enriched in noncanonical condensation (C) domains. (a) Organization of the prioritized *mxg* from *Cystobacterineae* sp. MCy9003. Genes and the encoded protein domains putatively involved in myxoglucamide biosynthesis are highlighted in color, whereas flanking genes with unknown or nonessential functions are shown in dashed arrows. (b) Phylogenetic analysis of C and C‐like domains involved in the *mxg* pathway together with representative domains from the NaPDoS database [19] and recently reported C domains with unusual functions [21, 22, 23, 24]. Domain subtypes are indicated by different colors, and *mxg*‐derived domains are highlighted by red dots. (c) Promoter exchange of the *mxg* activates myxoglucamide production in *Cystobacterineae* sp. MCy9003. Base peak chromatograms (BPCs) of the wild‐type strain (coral) and the *mxg* activation mutant (blue) are shown, with myxoglucamides (**1**–**4**) detected only upon pathway activation. A schematic representation of promoter refactoring in the native host is shown above.

### 2.2 Activation of *mxg* Reveals Novel Glycolipopeptides Confirmed by Comprehensive Structural Elucidation

To access the products of the cryptic *mxg* biosynthetic gene cluster, we activated the pathway by inserting a constitutive *tn5* promoter upstream of the first NRPS gene, *mxgA*, in *Cystobacterineae* sp. MCy9003. Comparative ultra‐high‐performance liquid chromatography high‐resolution mass spectrometry (UPLC‐HRMS) analysis of the mutant and wild‐type strains revealed four new metabolites (Figures 1c and S1–S4). Large‐scale cultivation (9 L) and subsequent structural characterization identified these compounds as a new family of glycolipopeptides, designated myxoglucamides A–D (**1**–**4**).

The HRMS showed the single charged ion [M+H]^+^ at *m*/*z* 645.40 for myxoglucamide A (**1**), consistent with the calculated molecular formula of C_32_H_57_N_2_O_11_. Analysis of ^1^H, HSQC, and COSY NMR spectra indicated the presence of a β‐pyranose sugar, based on the characteristic anomeric signal at H‐1′ (4.30 ppm), C‐1′ (103.9 ppm) and the specific coupling constant ^3^ *J* = 7.7 Hz (Figures 2a, S5–S11, and Table S2). Using the characteristic carbon chemical shifts [25], we assigned the pyranose as glucose. In addition, we identified an iso‐branched hydroxylated fatty acid chain based on COSY correlations of two methyl signals H‐1 (0.94 ppm) and H‐2 (0.93 ppm) to a methine proton H‐3 (1.92 ppm), which shows further COSY correlations to a second methine proton H‐4 (3.49 ppm), that in turn has COSY correlations in a longer alkyl chain (Figure 2a, Table S2). Due to overlapping methylene signals between 1.29 and 1.32 ppm, we used MS^2^ fragmentation data to determine the iso‐branched fatty acid chain to be 13‐hydroxy‐14‐methyl‐pentadecanoic acid (HMPA) (Figure S1). Further analysis of the HSQC and COSY spectra indicated the presence of α‐amino‐butyric acid (Abu). In addition, an unprecedented γ‐amino acid containing an α‐keto moiety and a vinylic moiety at γ‐position was identified and is hereafter referred to as vinyl‐substituted α‐keto/hydroxyl‐γ‐amino acid (Aoe/Ahe). The characteristic vinylic protons at H‐27a/b (5.19/5.11 ppm) form a spin system with one olefinic proton at H‐26 (5.86 ppm), the γ‐proton at H‐18 (4.88 ppm) and two diastereotopic protons at H‐19a/b (3.23/2.99 ppm), as shown by COSY correlations. HMBC correlations from H‐18 (4.88 ppm) to C‐20 (196.3 ppm), combined with HMBC correlations from H‐19a/b (3.23/2.99 ppm) to C‐20 and C‐21 (162.0 ppm), clearly depict this structural moiety (Table S2).

**FIGURE 2:**
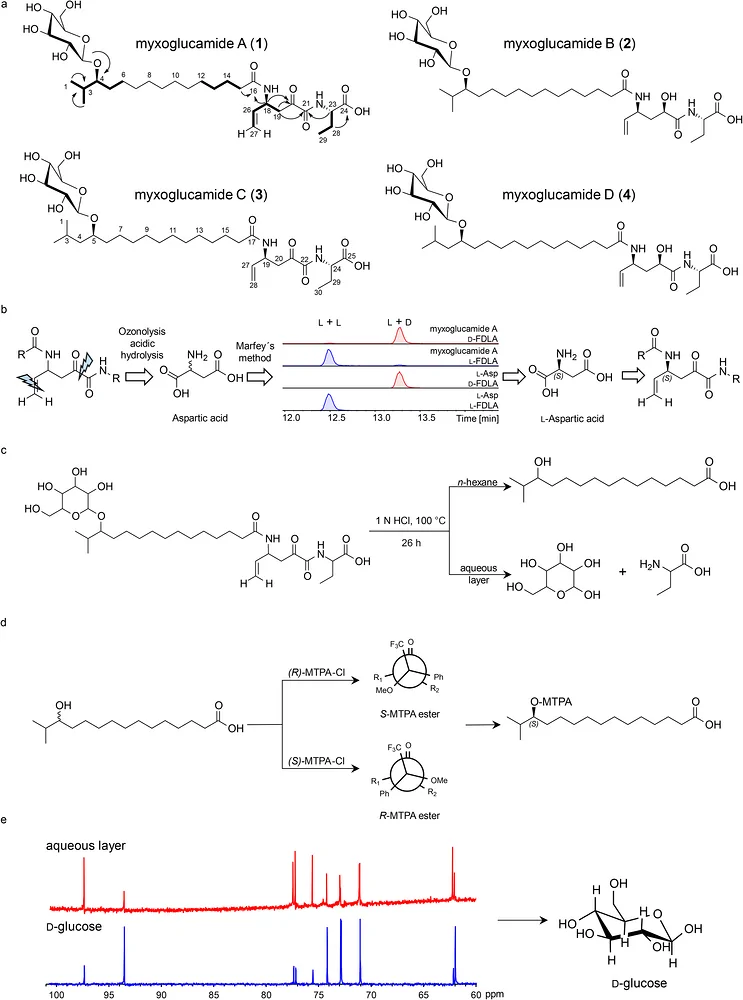
(a) Structures of myxoglucamides A–D. COSY (bold line) and HMBC (arrows) correlations of myxoglucamide A are indicated. (b) Determination of the vinyl group stereocenter by ozonolysis, oxidative workup and Marfey's derivatization with d‐ and l‐FDLA. Extracted ion chromatogram (EIC) at *m/z* = 428.14, corresponding to the Marfey's derivative. (c) Hydrolysis procedure of myxoglucamide A and workup using sequential liquid‐liquid partitioning. (d) Determination of the stereochemistry of the hydroxy‐methylpentadecanoic acid (HMPA): Esters of both MTPA variants were prepared and proton shift differences Δδ_(_ *_S‐R_*_)_ were determined. The absolute configuration was assigned as (*S*). (e) Determination of the pyranose type: ^13^C NMR spectrum of the aqueous layer (125 MHz, D_2_O, blue) and reference ^13^C NMR spectrum of d‐glucose (175 MHz, D_2_O, red).

Connectivity between glucose, HMPA chain, γ‐vinylic acid and Abu was established by examination of HMBC correlations. The glucose is linked to position 4 of the HMPA chain via an ether bridge, as indicated by HMBC from H‐1' (4.30 ppm) to C‐4 (85.2 ppm). Furthermore, the HMPA chain is connected via its carboxyl terminus to the γ‐vinylic acid, as shown by an HMBC correlation from H‐18 (4.88 ppm) to the HMPA carboxyl group at C‐16 (175.6 ppm). In addition, an HMBC signal from H‐23 (4.29 ppm) to C‐21 (162.0 ppm) is observed, implying that the Abu is linked via a peptide bond to the γ‐vinylic acid (Table S2). The structures of myxoglucamides B–D (**2**–**4**) strongly resemble **1** (Figure 2a, Tables S2–S5). One difference was identified to be a 14‐hydroxy‐15‐methyl‐hexadecanoic acid (HMHA) in **3** and **4** instead of the HMPA chain in **1** and **2**, as indicated by signals from additional diastereotopic methylene protons at H‐4a/b (1.27/1.51 ppm) and corresponding COSY correlations to the two methine protons at H‐3 (1.81 ppm) and H‐5 (3.74 ppm). Another difference was identified in **1** and **3** compared to **2** and **4**: an HSQC signal characteristic for an oxymethine at H‐20 (4.03 ppm)/C‐20 (70.0 ppm) in **2** and H‐21 (4.04 ppm)/C‐21 (70.2 ppm) in **4** replaced the carbon resonance observed for the ketone in **1** and **3**, implying that the ketone is reduced to an alcohol in **2** and **4** (Figures 2a, S5–S28, and Tables S2–S5). To determine the absolute configuration, we used a combination of ozonolysis, Marfey's method, and a *J*‐based analysis. By conducting Marfey's method, we determined that the C‐terminal Abu is l‐configured (Figures S29–S30, Table S6). The ozonolysis with oxidative workup and subsequent acid hydrolysis degraded the γ‐vinylic acid into free aspartic acid, which was determined to be l‐configured by Marfey's method (Figure 2b, Table S7). Since the stereocenter is not altered during ozonolysis and workup, the γ‐position can be assigned as *(S)* (Figure 2b). The *J*‐based analysis conducted for **4** suggests that positions 19 and 21 are syn‐configured, therefore position 21 can be assigned as *(R)* (Figures S31‐S35, Table S8). For **1** and **3**, we performed acid hydrolysis followed by isolating the products (Figures S36–S45, Tables S9–S14). The HMPA and HMHA chains were extracted from the reaction mixture using *n*‐hexane and subsequently derivatized with *(R)‐* and *(S)*‐α‐methoxy‐α‐trifluoromethylphenylacetic acid chlorides (MTPA‐Cl) [26] followed by NMR analysis to assign the hydroxyl position as *(S)* configured in both molecules (Figures 2c,d, S46–S55). The aqueous extract of the hydrolysis mixtures contained d‐glucose, which was proven by analysis of ^13^C NMR and the optical rotation (Figure 2e, Tables S10, and S13).

### 2.3 Heterologous Expression of the *mxg* Pathway Uncovers Various Acylated Myxoglucamide Congeners

To facilitate genetic manipulation and functional analysis of the *mxg* BGC, we heterologously expressed the pathway in the model myxobacterium *Myxococcus xanthus* DK1622. A cosmid (D9) carrying the complete *mxg* cluster from *Cystobacterineae* sp. MCy9003 was introduced into DK1622, and a bidirectional promoter cassette was integrated between *mxgH* and *mxgA* via double‐crossover recombination, to activate the pathway under the control of constitutive *tn5* and inducible *van* promoters (Figure S56).

UPLC‐HRMS/MS analysis revealed that only the DK1622 mutant harboring the bidirectional promoter construct (BiPD09), but not the strain retaining the native intergenic region (Mx8D09), produced detectable myxoglucamides **1–4** (Figure 3a). Notably, BiPD09 additionally produced many higher‐mass metabolites with longer retention times, which had previously been overlooked in MCy9003/mutant strain due to their trace amounts. MS/MS fragmentation analysis classified these compounds as myxoglucamide congeners, as their spectra closely resembled those of **1–4** and featured a characteristic aglycone fragment at *m/z* 479.35, indicative of conserved core scaffolds with variable glycosyl modifications (Figures 3a and S57).

**FIGURE 3:**
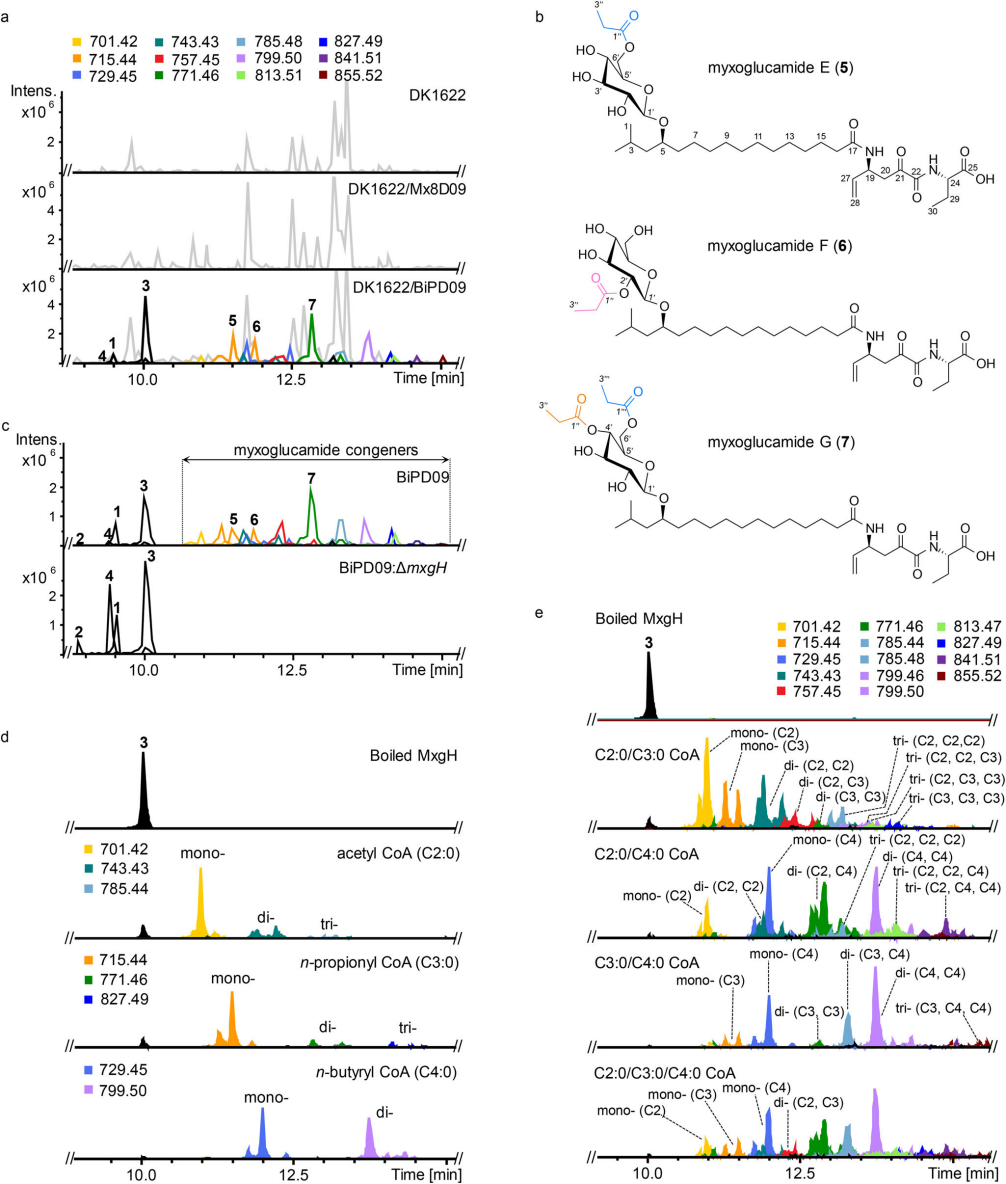
Discovery of various *O*‐acylated myxoglucamide congeners and characterization of the discrete C‐domain‐like *O*‐acyltransferase MxgH. (a) Heterologous expression of *mxg* in *M. xanthus* DK1622. BPCs of DK1622, DK1622 harboring Mx8D09, and BiPD09 are shown in grey. EICs of myxoglucamides A–D (**1**–**4**) are shown in black, while EICs of *O*‐acylated myxoglucamide congeners are highlighted in color. (b) Structures of *O*‐acylated myxoglucamide congeners **5–7**. (c) Deletion of *mxgH* abolishes production of *O*‐acylated myxoglucamides. (d) In vitro *O*‐acylation of **3** by recombinant MxgH using single fatty acyl‐CoA substrates of varying chain lengths (C2–C4), revealing formation of mono‐, di‐, and tri‐acylated products. (e) In vitro *O*‐acylation of **3** using mixtures of fatty acyl‐CoAs at equal molar ratios. As different *O*‐acylation patterns on the glucose moiety can give rise to products with identical *m/z* values, proposed product species and acyl compositions are annotated where applicable. A complete summary of the proposed product species, acyl compositions, and corresponding calculated and observed *m/z* values is provided in Table S18.

To elucidate these modifications, three major congeners (**5**‐**7**) were isolated (Figures 3b and S58–S60) and structurally characterized by NMR spectroscopy. For myxoglucamide E (**5**), an additional methyl signal at H‐3″ (1.13 ppm) formed a COSY spin system with a methylene proton at H‐2″ (2.36 ppm) (Figures S61–S65 and Table S15). Both protons showed HMBC correlations to a carbonyl carbon at C‐1″ (175.5 ppm), consistent with a propionyl group. HMBC correlations from the downfield‐shifted glucose methylene protons (H‐6′a/b, 4.16/4.41 ppm) to this carbonyl established attachment of the propionyl group at C‐6′ of the glucose. Similarly, myxoglucamide F (**6**) carries a propionyl substituent at C‐2′ of the glucose, while myxoglucamide G (**7**) bears two propionyl groups at C‐4′ and C‐6′ (Figures S66–S75 and Tables S16–S17).

### 2.4 The Discrete C‐Domain‐Like Protein MxgH Catalyzes Regioflexible *O*‐Acylation of the Glucose Moiety

The production of *O*‐acylated myxoglucamides in the heterologous expression mutant *M. xanthus* BiPD09 suggested that the discrete C‐domain‐like protein MxgH mediates these modifications. To test this hypothesis, *mxgH* was deleted from the expression construct, yielding BiPD09:Δ*mxgH*. Strikingly, production of the *O*‐acylated congeners **5–7** was completely abolished, while levels of the non‐acylated myxoglucamides **1–4** increased slightly (Figure 3c), indicating that MxgH functions as a tailoring enzyme responsible for glucose *O*‐acylation.

Structural comparison of **1–4** and **5–7** suggested that MxgH installs propionyl groups at different hydroxyl positions of the glucose moiety of **3**. Consistent with this, phylogenetic analysis placed MxgH within the Coenzyme A (CoA)‐dependent acyltransferase clan CL0149, to which the C‐domain family (PF00668) belongs, forming a distinct branch proximal to the PF16911 family (pathiocerol/phthiodiolone dimycocerosyl transferases; Figure S76). To directly probe its activity, recombinant MxgH was incubated in vitro with **3** and acyl‐CoAs of varying chain lengths. UPLC‐HRMS analysis revealed efficient conversion of **3** into higher‐mass products when C2–C8 acyl‐CoAs were supplied, whereas longer acyl donors (≥C10) were not accepted (Figures 3d and S77).

Multiple chromatographic peaks with identical masses but distinct retention times were observed, indicating regioflexible acylation of different sugar hydroxyl groups. Structural elucidation of congeners **5–7** confirmed modification at the C‐2′, C‐4′, and C‐6′ positions of glucose (Tables S15–S17). When *n*‐propionyl‐CoA was used, mono‐ ([M+H]^+^ = 715.44), di‐ ([M+H]^+^ = 771.46), and tri‐acylated ([M+H]^+^ = 827.49) products were detected, consistent with successive acylation events based on +56 Da mass increments (Figures 3d and S77–S78). Moreover, reactions containing mixtures of acyl‐CoAs generated additional products not observed with single donors, e.g., a product with [M+H]^+^ = 757.45 detected exclusively in reactions containing both C2 and C3 acyl‐CoAs, demonstrating that mixed multi‐acylation contributes to the observed metabolite diversity (Figure 3e, Table S18). While these data strongly support enzyme‐mediated regioflexible acylation, post‐catalytic acyl migration cannot be entirely excluded, as such rearrangements have been reported for carbohydrate esters under certain conditions [27].

Collectively, these results establish MxgH as a promiscuous, regioflexible *O*‐acyltransferase that tolerates a range of short‐chain acyl‐CoAs and generates extensive structural diversity. Site‐directed mutagenesis further identified the second histidine (H142) of the conserved HHxxxDG motif [28] as essential for activity, whereas neighboring residues were less critical (Figure S79), underscoring that MxgH represents a functional yet atypical C‐domain‐like member of the CoA‐dependent acyltransferase clan.

### 2.5 The Biosynthesis of Myxoglucamides Initiates with Fatty Acid Activation

The lipopeptide architecture of the myxoglucamides suggests that biosynthesis is initiated by the N‐terminal fatty acyl‐adenylate ligase (FAAL) domain of MxgA, which activates 14‐methyl‐pentadecanoic acid (MPA) or 15‐methyl‐hexadecanoic acid (MHA) in an ATP‐dependent manner, followed by transfer of the activated acyl group to the 4’‐phosphopantetheine arm of the cognate acyl carrier protein (ACP) domain. Consistent with this proposal, inactivation of *mxgA* completely abolished myxoglucamide production in the *mxg* heterologous expression system, demonstrating its essential role in pathway initiation (Figures S80–S81).

To directly validate this hypothesis, we expressed and purified a didomain FAAL‐ACP protein comprising the FAAL and ACP domains of MxgA in *E. coli* (Figure S82). As the protein was predominantly obtained in its apo form, the ACP domain was converted to holo form by incubation with the myxobacterial phosphopantetheinyl transferase MtaA [29] and CoA. Successful phosphopantetheinylation was confirmed by HRMS through the expected 340 Da mass increase (Figure 4a). Subsequent incubation of holo‐FAAL‐ACP with ATP and either MPA or MHA resulted in additional mass shifts of +238 Da or +252 Da, respectively, consistent with fatty acyl loading onto the ACP domain (Figure 4a).

**FIGURE 4:**
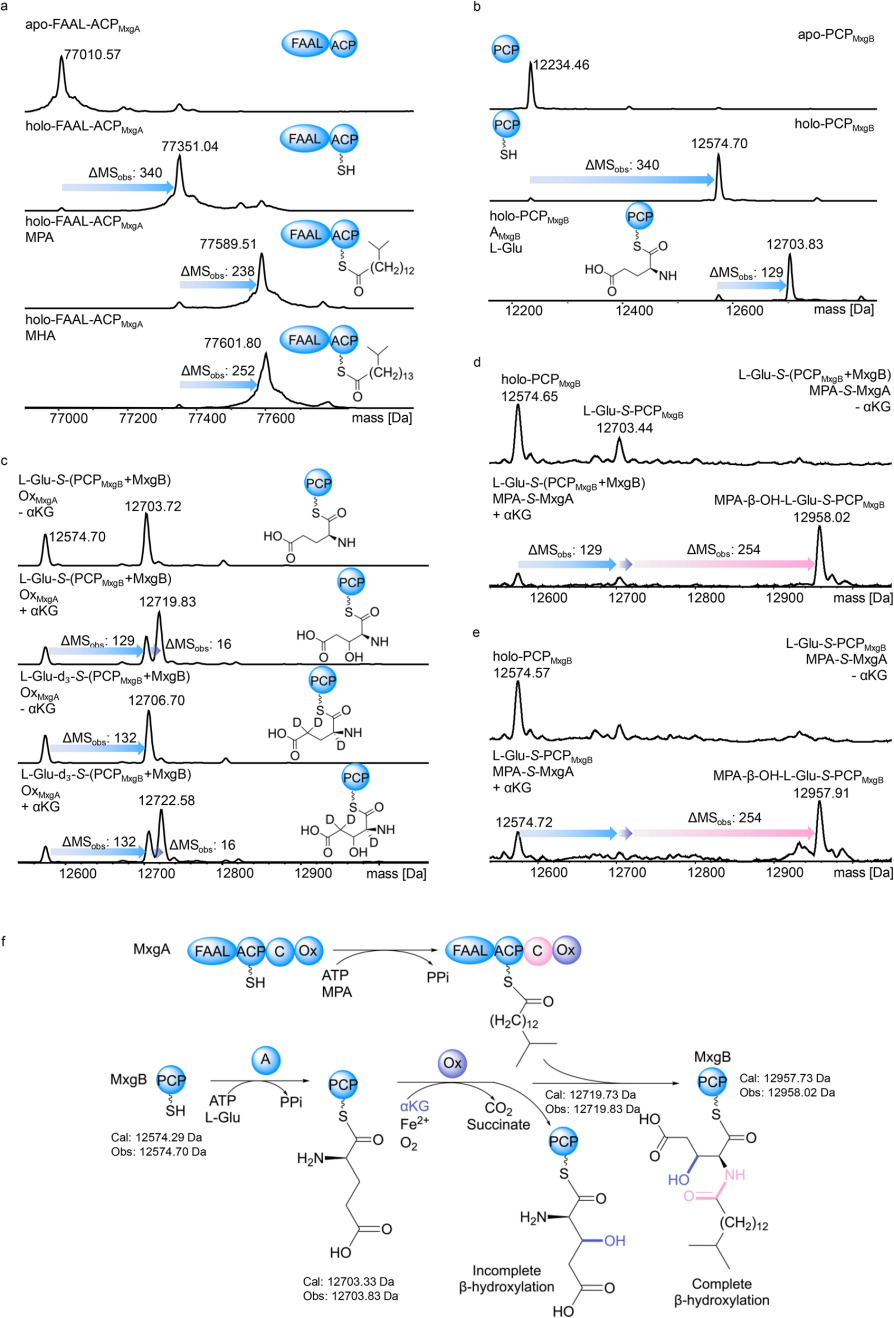
Biochemical characterization of MxgA and MxgB on the intact protein level. Full‐length proteins and split protein domains were used in the biochemical assays. Deconvoluted spectra of FAAL‐ACP_MxgA_ or PCP_MxgB_ under various in vitro conditions are shown. (a) MPA or MHA was loaded onto holo form of FAAL‐ACP_MxgA_. (b) L‐Glu was loaded onto PCP_MxgB_ by A_MxgB_. (c) β‐Hydroxylation of Glu on PCP_MxgB_ catalyzed by Ox_MxgA_. L‐Glu or 2,4,4‐D_3_‐L‐Glu was loaded onto PCP_MxgB_‐MxgB mixture and then incubated with Ox_MxgA_ with or without αKG. (d) The formation of MPA‐β‐OH‐L‐Glu‐*S*‐PCP_MxgB_ (12958.02 Da) only happened in the presence of αKG. (e) The formation of MPA‐β‐OH‐L‐Glu‐*S*‐PCP_MxgB_ happened in the presence of αKG when I_MxgB_ was absent. (f) Schematic representation of the formation of MPA‐β‐OH‐L‐Glu‐*S*‐PCP_MxgB_.

### 2.6 l‐Glutamate Serves as a Cryptic Precursor

The second building block of myxoglucamide scaffold is the unprecedented vinyl‐substituted α‐keto/hydroxyl‐γ‐amino acid Aoe/Ahe. Bioinformatic analysis, however, predicts that the adenylation (A) domain of the NRPS MxgB, which follows MxgA, activates l‐glutamate (l‐Glu; Table S19)—a residue not present in the final myxoglucamide structures. This prediction was supported by an altered isotope pattern upon supplementation of the production strain with ^13^C‐labeled glutamic acid (Figure S83). Using the malachite green assay for recombinant A domain of MxgB (A_MxgB_), we determined l‐Glu as the preferred substrate among the proteinogenic amino acids, suggesting its activation as the precursor for Aoe/Ahe (Figure S83). For further biochemical characterization, the peptidyl carrier protein (PCP) domain of MxgB (PCP_MxgB_) was expressed and purified. Incubation of holo‐PCP_MxgB_ with A_MxgB_, l‐Glu, and ATP resulted in an almost quantitative 129 Da mass increase, consistent with l‐Glu loading onto holo‐PCP_MxgB_ (Figure 4b). In contrast, alternative amino acids showed minimal or no loading, corroborating l‐Glu as the authentic substrate of MxgB (Figure S83).

### 2.7 Incomplete β‐Hydroxylation of PCP‐Bound l‐Glutamate by Ox_MxgA_

The confirmed activation and loading of l‐Glu by A_MxgB_ prompted comparison with the only well‐characterized biosynthesis of vinyl‐substituted amino acids, exemplified by the oxyvinylglycine pathway producing l‐2‐amino‐4‐methoxy‐*trans*‐3‐butenoic acid (AMB) [22]. Whereas the AMB BGC encodes two discrete α‐KG‐dependent dioxygenases, the *mxg* cluster contains only a single α‐KG‐dependent dioxygenase domain embedded at the C‐terminus of MxgA (hereafter Ox_MxgA_).

In addition, two C domains are present: one located between the ACP and Ox domains of MxgA, and another one at the N‐terminus of MxgB (Figure 1a). Phylogenetic analysis (Figure 1b) revealed that C_MxgA_ retains the canonical HHxxxDG motif and clusters with ^L^C_L_ domains, supporting a role in fatty‐acyl chain extension. In contrast, C_MxgB_ lacks the second histidine and groups with recently described interface (I) domains, which have been proposed to modulate β‐hydroxylation by α‐KG‐dependent oxygenases in certain NRPS systems [21] (hereafter designated as I_MxgB_).

To probe the role of Ox_MxgA_ in glutamyl β‐hydroxylation, we reconstituted the relevant steps in vitro using purified split individual domain and multidomain NRPS proteins (Figure S82). l‐Glu was first loaded onto PCP_MxgB_ using full‐length MxgB (Figure 4c). Subsequent incubation of l‐Glu‐bound PCP_MxgB_ with Ox_MxgA_ yielded a modified protein species displaying a +16 Da mass shift (Figure 4c), consistent with hydroxylation catalyzed by α‐KG oxygenases [30]. Regioselectivity was assessed using 2,4,4‐D_3_‐l‐Glu in the Ox_MxgA_ assay, which yielded the same +16 Da mass increase with complete retention of deuterium labels, unambiguously demonstrating β‐hydroxylation at C3 of l‐Glu tethered to holo‐PCP_MxgB_ (Figure 4c).

Notably, under all conditions tested, hydroxylation of PCP‐bound l‐Glu remained incomplete, with a substantial fraction of unmodified l‐Glu‐PCP_MxgB_ persisting (Figure 4c). While analogous β‐hydroxylation reactions have been reported in AMB [22] and glidomide [21] biosynthesis, previous studies primarily relied on analysis of offloaded small‐molecule intermediates. Therefore, the extent of β‐hydroxylation at the PCP‐bound stage has remained largely unexplored. Here, direct detection of carrier protein‐tethered species reveals that Ox_MxgA_ catalyzes β‐hydroxylation of PCP‐bound l‐Glu, but only to a partial extent under all conditions tested, indicating that efficient conversion likely requires additional assembly‐line context.

### 2.8 Completing β‐Hydroxylation and Subsequent Chain Extension Are Mutually Dependent

To determine how l‐Glu tethered to PCP_MxgB_ becomes fully β‐hydroxylated, we reconstituted the early steps of myxoglucamide biosynthesis in vitro. Full‐length holo‐MxgA was first loaded with MPA, while in parallel l‐Glu was loaded onto holo‐PCP_MxgB_ using full‐length MxgB. The two reaction mixtures were then buffer‐exchanged, combined, and supplemented sequentially with ascorbate, (NH_4_)_2_Fe(SO_4_)_2_, and finally α‐KG to initiate catalysis, ensuring that all relevant domains of MxgA and MxgB, together with their required cofactors, were present in a single reaction (Figure 4d).

Under these conditions, we detected the fully extended and hydroxylated intermediate MPA‐β‐OH‐l‐Glu‐*S*‐PCP_MxgB_ (Figure 4d). Notably, only limited amounts of holo‐PCP_MxgB_ and l‐Glu‐*S*‐PCP_MxgB_ were detected, indicating that β‐hydroxylation of l‐Glu‐*S*‐PCP_MxgB_ proceeded to completion concomitantly with fatty‐acyl chain extension to yield MPA‐β‐OH‐l‐Glu‐*S*‐PCP_MxgB_. In contrast, omission of the essential cofactor α‐KG completely abolished both β‐hydroxylation and chain extension. Instead, most of the Glu‐loaded PCP_MxgB_ reverted to the free holo‐PCP_MxgB_, likely due to nucleophilic cleavage of the thioester linkage by ascorbate supplemented in the assays [31].

Together with the observation that Ox_MxgA_ catalyzes only partial β‐hydroxylation of l‐Glu‐*S*‐PCP_MxgB_ in the absence of C_MxgA_, these results demonstrate that completion of β‐hydroxylation occurs only when fatty‐acyl chain extension is permitted (Figure 4f). Conversely, productive chain extension requires the oxidative step, establishing a tight functional coupling between these two transformations. Thus, although β‐hydroxylation can be initiated independently, its completion is strictly linked to condensation, and condensation does not proceed in the absence of concomitant β‐hydroxylation.

### 2.9 The C‐domain‐Like Interface Domain is Dispensable for Coupled β‐Hydroxylation and Fatty‐acyl Chain Extension

As two C‐domain‐like elements, C_MxgA_ and I_MxgB_ are present in the reaction system, we next examined whether the I domain I_MxgB_ is required for completion of glutamyl β‐hydroxylation during the coupled fatty‐acyl chain extension reaction. To this end, the adenylation domain of MxgB (A_MxgB_) was used instead of the full‐length MxgB to load l‐Glu onto holo‐PCP_MxgB_, thereby functionally excluding I_MxgB_ and eliminating any potential contribution from I_MxgB_. The resulting l‐Glu‐loaded holo‐PCP_MxgB_ was then incubated with MPA‐loaded full‐length holo‐MxgA under the same reaction conditions as in the Figure 4d assay.

Unexpectedly, removal of the I domain neither abolished fatty acyl chain extension nor β‐hydroxylation. Instead, substantial formation of the fully extended and hydroxylated intermediate MPA‐β‐OH‐l‐Glu‐*S*‐PCP_MxgB_ was observed (Figure 4e). In contrast to the near‐quantitative conversion observed in the presence of I_MxgB_, slightly more free holo‐PCP_MxgB_ was detected in the reaction mixture. This likely reflects incomplete turnover, with residual l‐Glu‐loaded PCP reverting to the holo form, potentially due to reduction by excess ascorbate, as observed in early assays lacking α‐KG. However, we cannot exclude the possibility that removal of the I domain also influences reaction kinetics.

This behavior differs from previously characterized I‐domain‐dependent systems, such as glidomide biosynthesis, in which the I domain is indispensable for β‐hydroxylation of PCP‐bound histidine by the standalone α‐KG‐dependent dioxygenase GlmF [21]. In the myxoglucamide pathway, by contrast, I_MxgB_ is not strictly required for completion of glutamyl β‐hydroxylation when condensation is enabled by C_MxgA_. Our data indicate that C_MxgA_ alone is sufficient to support both condensation and oxidative modification.

Together, these findings reveal that C‐domain subtypes, particularly I domains can fulfill distinct and context‐dependent roles across NRPS assembly lines. Rather than serving as essential recruitment or activation components for β‐hydroxylation [21, 32], I domains may perform alternative functions, such as modulating catalytic efficiency or recruiting additional catalytic enzymes. Further biochemical studies will be needed to clarify the functional diversity of this emerging subclass of C‐domain‐like proteins.

### 2.10 Biosynthetic Insights into Aoe/Ahe Formation Reveal Cryptic β,γ‐Double‐Bond Intermediates en Route to Unusual α‐Oxidation

To construct the γ‐amino acid scaffold, MxgC, which harbors a canonical PKS module is proposed to extend the PCP_MxgB_‐tethered intermediate by incorporating a malonyl‐CoA‐derived extender unit, analogous to the formation of other NRPS/PKS‐derived γ‐amino acid residues, such as statine biosynthesis in the pepstatin biosynthetic pathway [33]. The ketoreductase (KR) domain of MxgC subsequently reduces the β‐keto group derived from glutamate to the corresponding β‐hydroxyl group, which according to canonical PKS logic, would be dehydrated by the dehydratase (DH) domain to generate an α,β‐unsaturated intermediate.

Although DH_MxgC_ retains the conserved catalytic residues H1396 and D1558, we unexpectedly found that the tyrosine/phenylalanine residue of the canonical “YGP/FGP” motif, typically implicated in proton abstraction during dehydration [34], is replaced by leucine in DH_MxgC_ as LGP (Figure S84). To assess whether this atypical substitution affects DH_MxgC_ function, we performed site‐directed mutagenesis at this position. Myxoglucamide production was reduced to ∼10% in the L1516A mutant and nearly abolished in the L1516Y mutant, demonstrating that DH_MxgC_ is catalytically essential despite its noncanonical active‐site motif (Figure S84).

The epimerase (E) domain of MxgC is proposed to invert the stereochemistry of the newly formed γ‐aminoacyl intermediate generated from l‐Glu and malonyl‐CoA, consistent with the absolute configuration determined by ozonolysis and Marfey's analysis (Figure 2). This assignment is further supported by the presence of a downstream ^D^C_L_‐type condensation domain (catalyzing condensation between a d‐aminoacyl/peptidyl‐PCP donor and a l‐aminoacyl‐PCP acceptor) in MxgD, which elongates the scaffold by incorporation of l‐2‐aminobutyric acid (Abu). Finally, the C‐terminal thioesterase (TE) domain of MxgD releases the assembled NRP/PK chain, yielding mature myxoglucamides.

Despite this framework, the enzymatic basis for formation of the vinylic moiety, requiring dehydration and decarboxylation, remained unresolved. Among genes in the *mxg* lacking clear functional annotation, *mxgE* emerged as a prime candidate, as it encodes a protein comprising an N‐terminal C domain fused to a C‐terminal FMN‐dependent monooxygenase domain. Deletion of *mxgE* completely abolished myxoglucamide production, establishing its essential role in the pathway (Figure S80).

In the AMB biosynthetic system, an unusual C domain (C_modAA_) was implicated in dehydration and proposed to catalyze subsequent decarboxylation and isomerization, although the precise mechanism remains unclear [22]. However, phylogenetic analysis revealed that none of the C domains within the *mxg* cluster with C_modAA_. Instead, C_MxgE_ groups within the dual C domain clade (Figure 1b). Moreover, sequence analysis showed that C_MxgE_ harbors a proline residue in place of the second histidine within the canonical HHxxxDG motif, further distinguishing it from known dehydration‐associated C domains.

To assess the role of C_MxgE_, we generated H138A, P139A, and D143A point mutants. All three mutants abolished myxoglucamide production upon heterologous expression (Figure S85), indicating that C_MxgE_ functions as an integral component of the NRPS assembly line rather than as a late‐stage tailoring enzyme. We next interrogated the role of the FMN‐dependent monooxygenase domain (Ox_MxgE_) by site‐directed mutagenesis. Both F511A and H548A mutations eliminated production of **1–4** but instead led to the accumulation of new metabolites with [M+H]^+^ = 643.42 (Figures 5a and S86). One major product (**8**) was isolated and structurally characterized (Figure 5b). NMR analysis revealed that **8** shares the core scaffold of **3** and retains the vinyl moiety but lacks both the α‐keto and α‐hydroxyl functionalities characteristic of **1–7**. Instead, a previously unobserved double bond between C19 and C20 was detected (Figures S87–S92 and Table S20).

**FIGURE 5:**
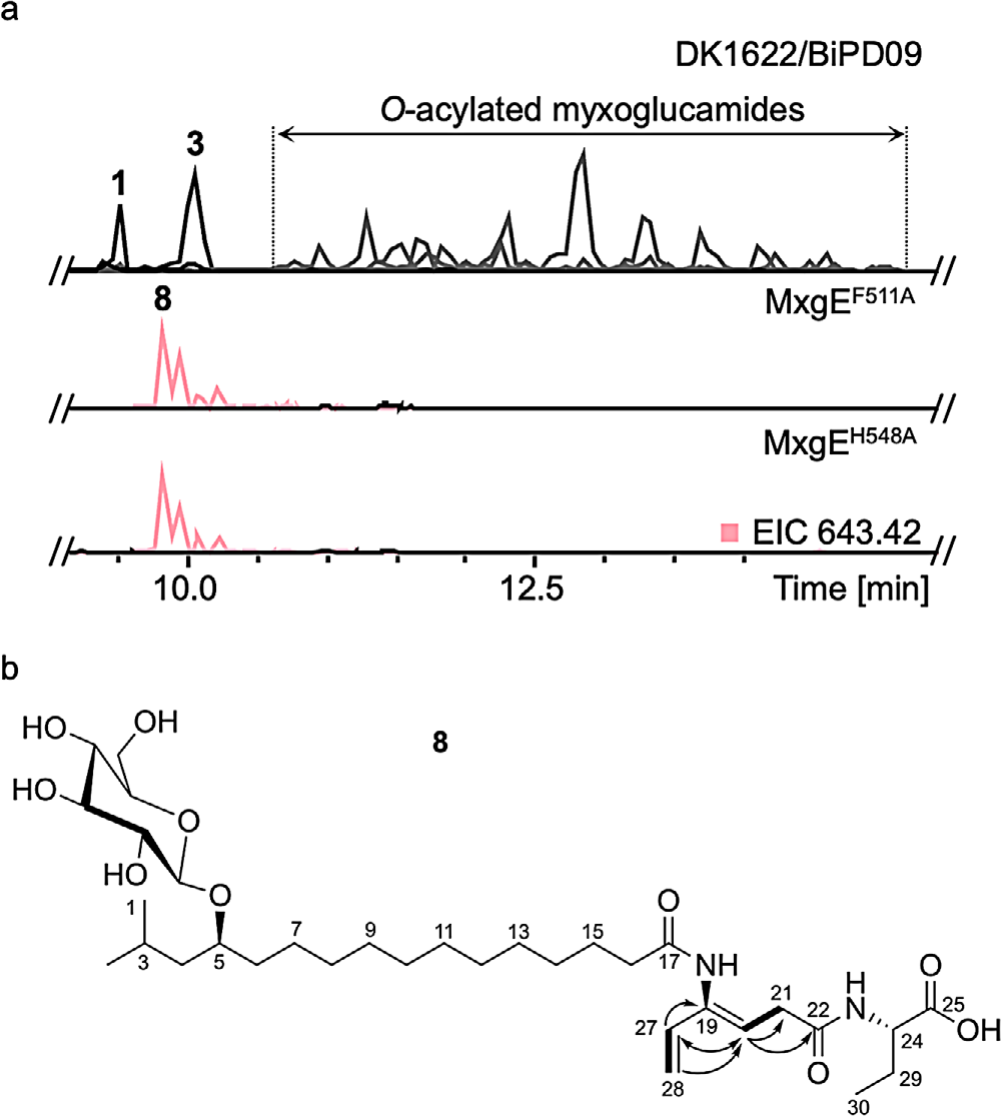
Identification and characterization of myxoglucamide intermediates with a cryptic β,γ‐double bond. (a) Site‐directed mutagenesis of two FMN‐binding residues (F511 and H548, Figure S86) in Ox_MxgE_ resulted in the accumulation of intermediates with EIC [M+H]^+^ = 643.42. (b) Chemical structure of intermediate **8** elucidated by NMR. COSY (bold line) and HMBC (arrows) correlations of **8** are indicated.

Notably, deletion of *mxgE* and point mutations in C_MxgE_ abolished myxoglucamide production, whereas point mutations in Ox_MxgE_ resulted in accumulation of **8**, suggesting that MxgE is essential for myxoglucamide biosynthesis, while the precise timing of Ox_MxgE_ activity relative to scaffold completion remains unclear.

Collectively, these data suggest that C_MxgE_ participates in a decarboxylative dehydration step required for formation of the vinylic moiety, whereas Ox_MxgE_ catalyzes α‐hydroxylation followed by oxidation to the α‐keto group, thereby completing the Aoe/Ahe motif. We therefore propose that C_MxgE_ mediates decarboxylative dehydration to generate the vinyl moiety once the myxoglucamide backbone is fully assembled on PCP_MxgD_. The presence of a β,γ‐double bond is particularly striking, as canonical DH domains typically generate α,β‐unsaturation. Two mechanistic scenarios could account for this feature: either DH_MxgC_ forms an α,β‐enoyl intermediate and subsequent double‐bond migration to the β,γ‐position, or DH_MxgC_ functions as a noncanonical DH capable of directly installing the β,γ‐double bond, which is difficult mechanistically. The precise mechanism remains to be elucidated, as no additional DH domain is present in the *mxg* pathway, distinguishing it from β,γ‐double‐bond formation in *trans*‐AT PKS systems [35, 36, 37]. Finally, the absence of α‐oxidation in **8** provides direct genetic and chemical evidence that Ox_MxgE_ installs the α‐hydroxyl group and further oxidizes it to the α‐keto functionality, thereby completing formation of the Aoe/Ahe motif in myxoglucamides **1–4**.

### 2.11 Function Assignment of the Remaining *mxg* Genes

To further understand the pathway, we examined the functions of the remaining *mxg* genes. Deletion of the P450‐encoding gene *mxgG* abolished myxoglucamide production in the heterologous expression host, indicating that *mxgG* plays an essential role in the pathway and suggesting its involvement in hydroxylation of the *iso*‐fatty acyl chain (Figure S80). In contrast, inactivation of the glycosyltransferase gene *orf10* had no detectable effect. However, deletion of the second glycosyltransferase gene, *mxgF*, completely eliminated myxoglucamide biosynthesis, indicating that MxgF catalyzes an essential *O*‐glucosylation step (Figure S80).

Notably, deletion of either *mxgF* or *mxgG* abolished metabolite production entirely, suggesting that fatty‐acyl hydroxylation and subsequent glycosylation occur early during biosynthesis and are integral to assembly‐line processing rather than late‐stage tailoring.

To further delineate the functional boundaries of the mxg biosynthetic gene cluster, four additional genes (*orf4*‐*orf7*) were individually inactivated and analyzed by heterologous expression (Figure S80). None of these deletions affected myxoglucamide production, indicating that these genes are dispensable for biosynthesis. Collectively, these results define *mxgF* as the 5′ boundary and *mxgE* as the 3′ boundary of the functional *mxg* biosynthetic pathway.

## 3 Conclusion

Noncanonical functions of C domains in NRPS assembly lines are emerging as powerful drivers of structural innovation in natural product biosynthesis. Activation of a cryptic hybrid NRPS/PKS BGC enriched in atypical C‐domain elements from the myxobacterial strain MCy9003 enabled the discovery of the myxoglucamides, a family of glycolipopeptides featuring an unprecedented γ‐amino acid bearing both a vinyl substituent and an α‐hydroxy/α‐keto amide functionality.

Heterologous expression of the *mxg* pathway via bidirectional promoter cassette exchange not only enabled myxoglucamide production but also revealed a diverse series of *O*‐acylated congeners. Genetic and biochemical analyses identified the standalone C‐domain‐like enzyme MxgH as the catalyst responsible for this diversification. MxgH accepts short‐chain fatty acyl‐CoAs (C2–C8) and catalyzes mono‐, di‐, and tri‐*O*‐acylation of hydroxyl groups on the glucose moiety of myxoglucamides, with site‐directed mutagenesis confirming that the conserved second histidine of the HHxxxDG motif is essential for activity.

Mechanistic dissection of the core assembly line revealed that the unusual vinyl‐substituted α‐hydroxy/α‐keto‐γ‐amino acid motif originates from l‐Glu through a series of noncanonical transformations (Figure 6). β‐Hydroxylation of PCP‐tethered l‐Glu by the α‐KG‐dependent dioxygenase Ox_MxgA_ is tightly coupled to upstream condensation, while condensation itself requires prior β‐hydroxylation, establishing a bidirectional checkpoint that enforces substrate fidelity during chain extension. Notably, the I domain I_MxgB_, previously proposed to be essential for analogous oxidative transformations in related systems is dispensable in the myxoglucamide pathway, underscoring the context‐dependent roles of C‐domain subtypes.

**FIGURE 6:**
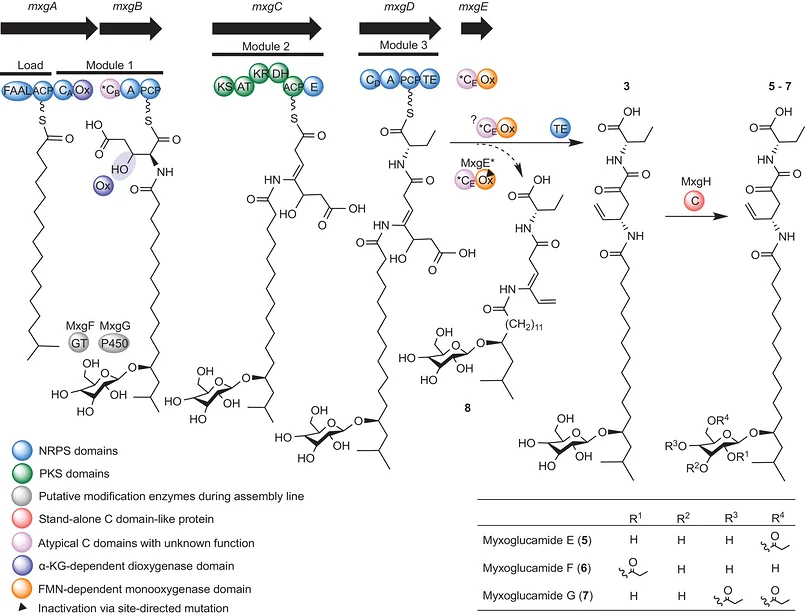
Proposed biosynthetic pathway of myxoglucamides. The structural differences among myxoglucamides E–G are summarized in the embedded table. MxgE* represents the Ox‐inactivated MxgE variant, resulting in the accumulation of intermediate **8**. The C domain of MxgE (C_MxgE_) is proposed to mediate decarboxylative dehydration to generate the vinyl moiety after myxoglucamide backbone is fully assembled on PCP_MxgD_.

Subsequent scaffold maturation is governed by the bifunctional enzyme MxgE. Genetic and chemical analyses demonstrate that the condensation domain C_MxgE_ is essential for decarboxylative dehydration, thereby generating the vinyl substituent characteristic of the Aoe/Ahe motif. Formation of the unusual β,γ‐unsaturation is enabled by the upstream DH domain of MxgC, which also introduces the additional two carbons to the backbone, distinguishing this pathway from canonical PKS logic that typically yields α,β‐unsaturated intermediates. The Ox_MxgE_ subsequently installs the α‐hydroxyl group and further oxidizes it to the α‐keto functionality, as evidenced by accumulation of a shunt metabolite lacking α‐oxidation. Together, these steps complete construction of the distinctive γ‐amino acid scaffold found in myxoglucamides.

Collectively, this work uncovers an unrecognized biosynthetic logic for the assembly of vinyl‐substituted, α‐oxidized γ‐amino acids and expands the functional repertoire of NRPS/PKS hybrid systems. More broadly, it highlights how atypical C‐domain‐like enzymes can function as pathway‐integrated proteins that enable and coordinate diverse functions to generate chemical complexity beyond canonical NRPS paradigms (Figure 6).

## Author Contributions

**Tingting Wang**: methodology, investigation, writing – original draft, writing – review and editing, visualization, data curation. **Alexander Popoff**: investigation, writing – original draft, methodology, visualization, data curation. **Maja Hunter**: investigation, writing – original draft, methodology, data curation, visualization. **Guangzhi Dai**: investigation, methodology, data curation. **Ronald Garcia**: investigation, methodology. **Huixin Xu**: methodology, investigation. **Haowen Zhao**: validation, data curation. **Asfandyar Sikandar**: validation, data curation, investigation. **Liujie Huo**: supervision, funding acquisition. **Rolf Müller**: conceptualization, funding acquisition, writing – review and editing, resources, supervision, project administration. **Chengzhang Fu**: conceptualization, investigation, funding acquisition, writing – review and editing, resources, supervision, project administration.

## Conflicts of Interest

The authors declare no conflicts of interest.

## Supporting information



The authors have cited additional references within the Supporting Information [38, 39, 40, 41, 42, 43, 44, 45, 46, 47]. **Supporting File**: anie73881‐sup‐0001‐SuppMat.pdf.

## Data Availability

The data that supports the findings of this study are available in the supplementary material of this article.
