# Supplementary material for: Atypical Condensation Domains Guide Discovery and Illuminate Biosynthesis of Myxoglucamides Featuring Vinyl‐Substituted, α‐Oxidized γ‐Amino Acids
Source: Angew Chem Int Ed Engl. 2026 Aug 7;65(41):e6995139. doi: 10.1002/anie.6995139 (PMC13638107; doi:10.1002/anie.6995139)
Supplement: Supplementary file 1 [file ANIE-65-e6995139-s001.pdf]

Supporting Information

Atypical Condensation Domains Guide Discovery and Illuminate Biosynthesis of Myxoglucamides Featuring Vinyl-Substituted, α-Oxidized γ-Amino Acids

Tingting Wang[a,b,d,+], Alexander Popoff[a,e,+], Maja Hunter[a,+], Guangzhi Dai[a,f], Ronald Garcia[a,b], Huixin Xu[a,b], Haowen Zhao[a,b], Asfandyar Sikandar[a], Liujie Huo[d], Rolf Müller*[a,b,c], and Chengzhang Fu*[a,b]

[a] Helmholtz-Institute for Pharmaceutical Research Saarland (HIPS), Helmholtz Centre for Infection Research (HZI) and Department of Pharmacy, Saarland University, Campus E8 1, 66123 Saarbrücken, Germany
E-mails: chengzhang.fu@helmholtz-hips.de, rolf.mueller@helmholtz-hips.de

[b] Helmholtz International Lab for Anti-Infectives, Helmholtz Centre for Infection Research, 38124 Braunschweig, Germany

[c] German Centre for Infection Research (DZIF), Partner Site Hannover - Braunschweig, Germany

[d] Helmholtz International Lab for Anti-Infectives, State Key Laboratory of Microbial Technology, Shandong University, 266237 Qingdao, China

[e] Departement of Pharmaceutical and Medicinal Chemistry, Institute of Pharmacy, Paracelsus Medical University, Strubergasse 21, 5020 Salzburg, Austria (Current address)

[f] School of Pharmaceutical Sciences and Institute of Materia Medica, Shandong Firest Medical University, 250117 Jinan, P. R. China (Current address)

[+] These authors contributed equally to this work.

*Corresponding Authors

Table of Contents

Materials and Methods 8

Cultivation of bacterial strains 8
Genetic manipulation of MCy9003 and mutant generation 8
Genetic manipulation of cosmids 9
Transformation of *M. xanthus* DK1622 and mutant verification 9
Sample preparation, UPLC-HRMS and UPLC-MS analysis 10
Gene cloning and protein purification 10
Substrate specificity measurement using a malachite green phosphate assay 11
In vitro loading experiments 11
Direct intact protein UPLC-ESI-MS analysis 12
Enzymatic assays for MxgH and variants 12
Phylogenetic tree 13
Isolation and structure elucidation of myxoglucamides 13
Myxoglucamide A-D 13
Myxoglucamide E-G 14
Intermediate **8** 15
Stereochemistry analysis 15
Marfey´s analysis 15
Ozonolysis 16
J-based analysis of myxoglucamides 16
Acid hydrolysis and Mosher analysis 16
Acid hydrolysis of myxoglucamide A 16
Determination of the stereogenic center in HMPA 16
Acid hydrolysis of myxoglucamide C 17
Determination of the stereogenic center of HMHA 17
The general information about the NMR spectrometers, probes and pulse sequences 17

Supporting Figures 18

Figure S1. HRMS^2 spectrum and observed fragments of myxoglucamide A 18
Figure S2. HRMS^2 spectrum and observed fragments of myxoglucamide B 19
Figure S3. HRMS^2 spectrum and observed fragments of myxoglucamide C 20
Figure S4. HRMS^2 spectrum and observed fragments of myxoglucamide D 21
Figure S5. ^{1}H spectrum of myxoglucamide A acquired in CD_3OD at 500 MHz 22
Figure S6. ^{13}C spectrum of myxoglucamide A acquired in CD_3OD at 125 MHz 22
Figure S7. H,H COSY spectrum of myxoglucamide A acquired in CD_3OD at 500 MHz 23
Figure S8. Enlarged H,H COSY spectrum of myxoglucamide A acquired in CD_3OD at 500 MHz 24
Figure S9. HSQC spectrum of myxoglucamide A acquired in CD_3OD at 500/125 MHz 25
Figure S10. HMBC spectrum of myxoglucamide A acquired in CD_3OD at 500/125 MHz 26
Figure S11. Enlarged HMBC spectrum of myxoglucamide A acquired in CD_3OD at 500/125 MHz 27
Figure S12. ^{1}H spectrum of myxoglucamide B acquired in CD_3OD at 700 MHz 28
Figure S13. H,H COSY spectrum of myxoglucamide B acquired in CD_3OD at 700 MHz 29
Figure S14. Enlarged H,H COSY spectrum of myxoglucamide B acquired in CD_3OD at 700 MHz 30
Figure S15. HSQC spectrum of myxoglucamide B acquired in CD_3OD at 700/175 MHz 31
Figure S16. HMBC spectrum of myxoglucamide B acquired in CD_3OD at 700/175 MHz 32
Figure S17. ^{1}H spectrum of myxoglucamide C acquired in CD_3OD at 500 MHz.. 33
Figure S18. ^{13}C spectrum of myxoglucamide C acquired in CD_3OD at 125 MHz 33
Figure S19. H,H COSY spectrum of myxoglucamide C acquired in CD_3OD at 500 MHz 34
Figure S20. Enlarged H,H COSY spectrum of myxoglucamide C acquired in CD_3OD at 500 MHz 35
Figure S21. HSQC spectrum of myxoglucamide C acquired in CD_3OD at 500/125 MHz 36
Figure S22. HMBC spectrum of myxoglucamide C acquired in CD_3OD at 500/125 MHz 37
Figure S23. ^{1}H spectrum of myxoglucamide D acquired in CD_3OD at 700 MHz.. 38
Figure S24. ^{13}C spectrum of myxoglucamide D acquired in CD_3OD at 175 MHz 38
Figure S25. H,H COSY spectrum of myxoglucamide D acquired in CD_3OD at 700 MHz 39
Figure S26. Enlarged H,H COSY spectrum of myxoglucamide D acquired in CD_3OD at 700 MHz 40
Figure S27. HSQC spectrum of myxoglucamide D acquired in CD_3OD at 700/175 MHz 41
Figure S28. HMBC spectrum of myxoglucamide D acquired in CD_3OD at 700/175 MHz 42
Figure S29. Marfey's analysis (L-FDLA) for amino-butyric acid standards and four isolated myxoglucamides after acid hydrolysis 43
Figure S30. Marfey's analysis (D-FDLA) for amino-butyric acid standards and four isolated myxoglucamides after acid hydrolysis 43
Figure S31. J resolved COSY spectrum of myxoglucamide D showing coupling pattern of H-19 44
Figure S32. J resolved COSY spectrum of myxoglucamide D showing coupling pattern of Ha-20 and Hb-20 44
Figure S33. J resolved COSY spectrum of myxoglucamide D showing coupling pattern of H-21 45

Figure S34. HC coupling constants for the J based analysis, extracted from a dipsi2 NMR experiment...... 45
Figure S35. HC coupling constants for the J based analysis, extracted from a HSQMBC NMR experiment 46
Figure S36. ^1H spectrum of HMHA acquired in $CDCl_3$ at 500 MHz isolated after acid hydrolysis of myxoglucamide C 47
Figure S37. HSQC spectrum of HMHA acquired in $CDCl_3$ at 500 MHz isolated after acid hydrolysis of myxoglucamide C 48
Figure S38. HMBC spectrum of HMHA acquired in $CDCl_3$ at 500 MHz isolated after acid hydrolysis of myxoglucamide C 49
Figure S39. ^1H spectrum of aqueous extract of acid hydrolysis of myxoglucamide C acquired in D_2O at 500 MHz 50
Figure S40. ^{13}C spectrum of aqueous extract of acid hydrolysis of myxoglucamide C acquired in D_2O at 125 MHz 51
Figure S41. ^1H spectrum of HMPA acquired in $CDCl_3$ at 700 MHz isolated after acid hydrolysis of myxoglucamide A 52
Figure S42. HSQC spectrum of HMPA acquired in $CDCl_3$ at 700 MHz isolated after acid hydrolysis of myxoglucamide A 53
Figure S43. HMBC spectrum of HMPA acquired in $CDCl_3$ at 700 MHz isolated after acid hydrolysis of myxoglucamide A 54
Figure S44. ^1H spectrum of aqueous extract of acid hydrolysis of myxoglucamide A acquired in D_2O at 500 MHz 55
Figure S45. ^{13}C spectrum of aqueous extract of acid hydrolysis of myxoglucamide A acquired in D_2O at 125 MHz 56
Figure S46. ^1H spectrum of the (R)-MTPA-HMHA ester reaction mixture acquired pyridine-d_5 at 700 MHz 57
Figure S47. ^1H spectrum of the (S)-MTPA-HMHA ester reaction mixture acquired pyridine-d_5 at 700 MHz 57
Figure S48. HSQC spectrum of the (S)-MTPA-HMHA ester reaction mixture acquired in pyridine-d_5 at 700/175 MHz 58
Figure S49. HSQC spectrum of the (R)-MTPA-HMHA Ester reaction mixture acquired in pyridine-d_5 at 700/175 MHz 58
Figure S50. Overlapped HSQC spectra of (R)-MTPA-HMHA ester with CH_2 groups displayed in red, CH/CH_3 groups displayed in pink, and the (S)-MTPA-HMHA ester with CH_2 groups displayed in blue and CH/CH_3 groups displayed in green 59
Figure S51. ^1H spectrum of the (S)-MTPA-HMPA Ester reaction mixture acquired pyridine-d_5 at 700 MHz 60
Figure S52. ^1H spectrum of the (R)-MTPA-HMPA Ester reaction mixture acquired pyridine-d_5 at 700 MHz 60
Figure S53. HSQC spectrum of the (S)-MTPA-HMPA Ester reaction mixture acquired in pyridine-d_5 at 700/175 MHz 61
Figure S54. HSQC spectrum of the (R)-MTPA-HMPA ester reaction mixture acquired in pyridine-d5 at 700/175 MHz 61

Figure S55. Overlapped HSQC spectra of (S)-MTPA-HMPA ester with CH_2 groups displayed in red, CH/CH_3 groups displayed in pink and (R)-MTPA-HMPA ester with CH_2 groups displayed in blue, CH/CH_3 groups displayed in green 62
Figure S56. Modification of cosmid D09........ 63
Figure S57. MS^2 fragmentation of myxoglucamides and their congeners from crude extracts........ 64
Figure S58. $HRMS^2$ spectrum and observed fragments of myxoglucamide E........ 65
Figure S59. $HRMS^2$ spectrum and observed fragments of myxoglucamide F 66
Figure S60. $HRMS^2$ spectrum and observed fragments of myxoglucamide G........ 67
Figure S61. ^1H spectrum of myxoglucamide E acquired in CD_3OD at 700 MHz........ 68
Figure S62. COSY spectrum of myxoglucamide E acquired in CD_3OD at 700 MHz........ 69
Figure S63. Enlarged COSY spectrum of myxoglucamide E acquired in CD_3OD at 700 MHz 70
Figure S64. HSQC spectrum of myxoglucamide E acquired in CD_3OD at 700/ 175 MHz 71
Figure S65. HMBC spectrum of myxoglucamide E acquired in CD_3OD at 700/ 175 MHz 72
Figure S66. ^1H spectrum of myxoglucamide F acquired in CD_3OD at 700 MHz........ 73
Figure S67. COSY spectrum of myxoglucamide F acquired in CD_3OD at 500 MHz........ 74
Figure S68. Enlarged COSY spectrum of myxoglucamide F acquired in CD_3OD at 500 MHz 75
Figure S69. HSQC spectrum of myxoglucamide F acquired in CD_3OD at 500/125 MHz........ 76
Figure S70. HMBC spectrum of myxoglucamide F acquired in CD_3OD at 500/ 125 MHz 77
Figure S71. ^1H spectrum of myxoglucamide G acquired in CD_3OD at 700 MHz 78
Figure S72. COSY spectrum of myxoglucamide G acquired in CD_3OD at 700 MHz 79
Figure S73. Enlarged COSY spectrum of myxoglucamide G acquired in CD_3OD at 700 MHz........ 80
Figure S74. HSQC spectrum of myxoglucamide G acquired in CD_3OD at 700/ 175 MHz........ 81
Figure S75. HMBC spectrum of myxoglucamide G acquired in CD_3OD at 700/ 175 MHz........ 82
Figure S76. Phylogenetic analysis of MxgH together with representative acyltransferases from the CL0149 family 83
Figure S77. MxgH catalyzed O-acylation of myxoglucamide C with fatty acyl-CoAs of different chain lengths 84
Figure S78. MS^2 fragmentation of products from MxgH-CoAs assays........ 85
Figure S79. Mutational analysis of MxgH........ 86
Figure S80. Gene knockouts of mxg in M. xanthus DK1622........ 87
Figure S81. Verification of gene knock-out in modified cosmids and DK1622 mutants 88
Figure S82. SDS-PAGE analysis of proteins used in **Figure 4**........ 89
Figure S83. Incorporation of glutamate and substrate specificity of A_{mxgB} for 20 amino acids........ 90
Figure S84. Sequence alignment and functional analysis of catalytic residues in DH_{MxgC}........ 91
Figure S85. Site-directed mutagenesis of C_{MxgE}........ 92
Figure S86. Sequence alignment of Ox_{MxgE} with other luciferase-like monooxygenase class flavin-dependent oxidoreductases and gel verification of Ox_{MxgE} mutants........ 93
Figure S87. ^1H spectrum of intermediate **8** acquired in CD_3OD at 700 MHz 94

Figure S88. ^{13}C spectrum of intermediate **8** acquired in CD_3OD at 175 MHz 95
Figure S89. HSQC spectrum of intermediate **8** acquired in CD_3OD at 700/175 MHz 96
Figure S90. HMBC spectrum of intermediate **8** acquired in CD_3OD at 700/175 MHz 97
Figure S91. COSY spectrum of intermediate **8** acquired in CD_3OD at 700 MHz 98
Figure S92. TOCSY spectrum of intermediate **8** acquired in CD_3OD at 700 MHz 99

Supporting Tables 100

Table S1. Gene annotation of open reading frames from mxg gene cluster 100
Table S2. Spectroscopic data of myxoglucamide A acquired in CD_3OD 102
Table S3. Spectroscopic data of myxoglucamide B acquired in CD_3OD 104
Table S4. Spectroscopic data of myxoglucamide C acquired in CD_3OD 106
Table S5. Spectroscopic data of myxoglucamide D acquired in CD_3OD 108
Table S6. Marfey´s analysis of myxoglucamides compared to the L-Abu/D-Abu standards 109
Table S7. Marfey's analysis of myxoglucamides after ozonolysis compared to the L-Asp standards 109
Table S8. Obtained coupling constants for the J-based analysis 110
Table S9. Spectroscopic data of n-hexane extract from hydrolysis mixture of myxoglucamide A in $CDCl_3$ containing HMPA 110
Table S10. Chemical shifts of the β-Glucose in the methanol extract from the acidic hydrolysis of myxoglucamide A obtained in D_2O 111
Table S11. Spectroscopic data of both Mosher esters of the HMPA chain in pyridine-d_5 111
Table S12. Spectroscopic data of n-hexane extract from hydrolysis mixture of myxoglucamide C in $CDCl_3$ containing HMHA 112
Table S13. Chemical shifts of the β-Glucose in the methanol extract from the acidic hydrolysis of myxoglucamide C obtained in D_2O 112
Table S14. Spectroscopic data of both Mosher esters of the HMHA chain in pyridine-d_5 113
Table S15. Spectroscopic data myxoglucamide E acquired in CD_3OD 114
Table S16. Spectroscopic data myxoglucamide F acquired in CD_3OD 116
Table S17. Spectroscopic data myxoglucamide G acquired in CD_3OD 117
Table S18. Proposed acylated product species from in vitro O-acylation assays using fatty acyl-CoAs of different chain lengths or their mixtures 118
Table S19. A domain substrate specificity prediction 120
Table S20. Spectroscopic data of intermediate **8** acquired in CD_3OD 121
Table S21. Primers used in this study 122

Reference 128

Materials and Methods

Cultivation of bacterial strains

Escherichia coli DH10β was used for cloning, and *E. coli* BL21(DE3) for recombinant protein expression. *E. coli* strains were cultivated in LB medium (10 g/L tryptone, 5 g/L NaCl, 5 g/L yeast extract) or 2TY medium (1.6 % tryptone, 1 % yeast extract, 0.5 % NaCl, supplemented with 1.8% agar for solid medium) at 37 °C, or at 30 °C for maintenance of plasmids larger than 20 kb. Antibiotics were supplemented at the following concentrations: ampicillin (100 µg/mL), chloramphenicol (12.5 µg/mL), kanamycin (50 µg/mL), apramycin (50 µg/mL), spectinomycin (50 µg/mL), and zeocin (50 µg/mL).

Cystobacterineae sp. MCy9003 was cultivated at 30 °C in CyHv3 medium (2 g/L Bacto™ soytone, 3 g/L Bacto™ casitone, 2 g/L glucose, 3 g/L soluble starch, 1.5 g/L Bacto™ yeast extract, 0.5 g/L $CaCl_2 \times 2H_2O$, 1 g/L $MgSO_4 \times 7H_2O$, 50 mM HEPES, 8 mg/L Fe-EDTA, pH 7.2) or CyS medium (2.5 g/L Bacto™ casitone, 1 g/L Bacto™ yeast extract, 1 g/L $CaCl_2 \times 2H_2O$, 2.5 g/L soluble starch, 10 mM HEPES, pH 7.0). Kanamycin (75 µg/mL) was added for cultivation of mutant strains. Liquid cultures were grown in 300 mL Erlenmeyer flasks containing 50 mL CyHv3 medium at 30 °C and 180 rpm for 1-2 days. Seed cultures (2%, v/v) were then transferred to CyS medium and cultivated for 10-12 days in baffled Erlenmeyer flasks for fermentation. XAD-16 resin (2 %, v/v) was added to the culture broth after fermentation.

Myxococcus xanthus DK1622 was used as the heterologous expression host and cultivated in CTT medium (10 g/L casitone, 1.21 g/L TRIS, 8 mM $MgSO_4$, 1 mM KH_2PO_4, pH 7.6) or CyS medium for genomic DNA isolation, transformation, production screening and fermentation. Strains harboring modified cosmids were recovered from cryostocks on CTT agar plates and incubated at 30 °C for 3-4 days, followed by inoculation into CTT liquid medium and cultivation for 2 days as seed cultures. 10-15% (v/v) seed cultures were used for screening or fermentation. After one day, vanillate (1 mM) and XAD-16 (2%) were added. The cultures and resins were harvested after an additional six days. All liquid cultivations were performed at 30 °C and 180 rpm. Kanamycin (50 µg/mL), apramycin (50 µg/mL) or zeocin (50 µg/mL) were used for selection of *M. xanthus* mutants.

Genetic manipulation of MCy9003 and mutant generation

Detailed procedures for the genetic manipulation of MCy9003 and generation of mutants have been described previously[1] and are briefly summarized below.

The *tn5* promoter was PCR amplified from pSBtn5Kan plasmid[2], which contains a pCR2.1 vector backbone from the TOPO-TA cloning kit (Thermo Scientific, USA) and a *tn5* promoter, using primers tn5-HindIII-F and tn5-R. The 182-bp DNA fragment and pSBtn5Kan plasmid were digested with *Hind*III and *Eco*RI. The plasmid was dephosphorylated prior to ligation, resulting in pSB2tn5Kan. The homology region for overexpression of *mxg* was PCR-amplified from MCy9003 genomic DNA using primers 001bgc18-NdeI-F and 001bgc18-EcoRI-R, digested with *Nde*I and *EcoR*I, and ligated into dephosphorylated pSB2tn5Kan, yielding pSB2tn5Kan001bgc18[1]. Plasmid sequences were confirmed using M13-21F and M13-24R primers.

The constructs were electroporated into MCy9003 wild-type (650 V, 25 µF, 400 Ω), plated on CyHv3 agar containing 75 µg/mL kanamycin, and incubated at 30 °C for 6-10 days. Transformants were replated onto fresh CyHv3 agar with kanamycin and incubated at 30 °C for 2-4 days. Cell mass was collected, resuspended in 300 µL ddH_2O, and genomic DNA was extracted using the Gentra Puregene Cell Kit. Integration of the pSBtn5Kan-based construct at both the left and right homologous regions was confirmed by DNA amplification with one primer on either left (g001-bgc18-F) or right (g001-bgc18-R) side of the homology region and the other primer on the plasmid (M13-24R or M13-21F), respectively (Table S21).

Genetic manipulation of cosmids

Cosmid D09 harbors the putative *mxg* gene cluster along with eight additional genes. To enable heterologous expression, the Mx8 phage integrase gene was introduced into the D09 backbone using the Redαβ recombination system[3]. A bidirectional promoter cassette, consisting of a constitutive *tn5* promoter, an inducible *van* promoter, and a kanamycin resistance gene, was introduced between *mxgH* and *mxgA*.

Briefly, the ampicillin resistance gene *ampR* was PCR-amplified from pUC18 using primers containing *Spe*I restriction sites and 40-bp homologous arms corresponding to *mxgH* or *mxgA*. The purified PCR product was inserted between *mxgH* and *mxgA* via Redαβ recombineering[4]. The resulting cosmid (amp-Mx8D09) was digested with *Spe*I, and the larger fragment was purified and assembled with the bidirectional promoter cassette by Gibson assembly[5], yielding the bidirectional promoter cosmid BiPD09.

For gene knockout in the heterologous expression system, an apramycin resistance gene was amplified and used to replace target genes on the cosmid via Redαβ recombination. For recombineering, 1% overnight culture of *E. coli* GB08Red was inoculated into LB medium and grown at 37 °C to an OD_{600} of 0.4-0.6. Expression of the Redαβ system was induced by adding 10% (w/v) L-arabinose to a final concentration of 0.25% (w/v), followed by incubation for 40 min at 37 °C. Cells were harvested, washed twice with ice-cold ddH_2O, and resuspended in 30 µL ddH_2O. Approximately 100-200 ng of insert fragment and corresponding cosmid were co-electroporated into *E. coli* GB08Red (1350 V, 3-6 ms). After overnight cultivation on LB agar plates containing appropriate antibiotics, positive colonies were cultured overnight, and cosmids were isolated by alkaline lysis.

For site-directed mutagenesis of MxgC and MxgE, the ccdB-ampicillin resistance cassette flanked by *Pme*I sites was similarly amplified with gene-specific homologous regions. Each target gene was divided into two fragments, and the desired mutations were introduced at the junction using primers listed in Table S21. The resulting mutant fragments were assembled into *Pme*I-digested cosmid BiPD09 via Gibson assembly.

Modified cosmids were verified by Sanger sequencing (LGC Genomics GmbH) or restriction enzyme digestion before being electroporated into *M. xanthus* DK1622.

Transformation of *M. xanthus* DK1622 and mutant verification

Overnight DK1622 cultures (2 mL) were harvested, washed twice with 1 mL ddH_2O, and resuspended in 35 µL ddH_2O. Then 10 µL of cosmid (1-3 µg) was added to the cell suspension. Electroporation was performed using 1 mm cuvettes under the following conditions: 650 V, 25 µF,

400 Ω. Transformed cells were resuspended in 1 mL CTT medium and incubated at 30 °C with constant shaking for 6 h. Afterward, cells were mixed with 3 mL of CTT soft agar (CTT with 7.5 g/L agar) and overlaid onto CTT agar plates containing the corresponding antibiotics. Plates were incubated at 30 °C for 5 days. Single colonies were isolated onto fresh antibiotic-containing CTT agar plates and incubated at 30 °C for 5 days. Integration of the constructs into the chromosome occurred via site-specific recombination at the Mx8 attachment site. Integration was verified by PCR using different combinations of the primers Mx8-attB-up2, Mx8-attB-down, Mx8-attP-up2, Mx8-attP-down[6], Mxg-veri-4F/4R, Mxg-veri-5F/5R and MxgH-Tn5-F/MxgA-van-R. For colony PCR, genomic DNA was prepared by scraping ~5 mm^2 of cell material from CTT agar, resuspending in 50 µL ddH_2O, and lysing at 95 °C for 30 min prior to PCR verification.

Sample preparation, UPLC-HRMS and UPLC-MS analysis

Cells and XAD-16 resin from screening or fermentation cultures were harvested by centrifugation (6,000 rpm, 10 min, 4 °C) and extracted with 50 mL MeOH/acetone (1:1) under agitation at room temperature for 3 h. Extracts were filtered using folded filter paper (8-12 µm pore size) and evaporated to dryness *in vacuo*. The dried extract was dissolved in 1 mL methanol for analysis.

UPLC-HRMS was performed on an UltiMate 3000 LC System (Dionex) equipped with an Acquity UPLC BEH C18 column (1.7 µm, 100 × 2 mm; Waters) and VanGuard BEH C18 guard column (1.7 µm; Waters), coupled to an Apollo II ESI source (Bruker) and a maXis 4G ToF mass spectrometer (Bruker). Separation was conducted at 0.6 mL/min using eluent A: deionized water + 0.1% formic acid (FA) and eluent B: acetonitrile + 0.1% FA at 45 °C with the following gradient: 5% B for 30 s, linear to 95% B in 18.5 min, maintained at 95% B for 2 min. The gradient was adjusted by returning to 5% B within 30 s, followed by re-equilibration at 5% B for 1.5 min. The LC flow was split to 75 µL/min prior to entering the mass spectrometer. Mass spectra were acquired in centroid mode ranging from 150 to 2500 *m/z* at a 2 Hz full scan rate. Mass spectrometry source parameters were set to 500 V as endplate offset, 4000 V as capillary voltage, 1 bar nebulizer gas pressure, 5 L/min dry gas flow, and 200 °C dry temperature. For MS^2, CID (collision-induced dissociation) energy was ramped from 35 eV for 500 *m/z* to 45 eV for 1000 *m/z*. MS full scan acquisition rate was set to 2 Hz and MS^2 spectra acquisition rates were ramped from 1 to 4 Hz for precursor ion intensities of 10 kcts to 1000 kcts.

UPLC-MS was conducted on an UltiMate 3000 RSLC system coupled to an amaZon speed 3D ion trap mass spectrometer (Bruker) equipped with an Apollo II ESI source in positive mode. Samples were separated on a BEH C18 column (1.7 µm, 100 × 2.1 mm; Waters) at 0.6 mL/min and 45 °C using eluent A: deionized water + 0.1% FA and eluent B: methanol + 0.1% FA. The gradient was as follows: 5% B for 30 s, linear gradient up to 20% B in 1 min, linear gradient up to 30% B in 13 min, linear gradient up to 95% B in 3 min, maintained at 95% B for 3 min and re-equilibrated at 5% B in 30 s. The LC flow was split to 75 µL/min before entering the mass spectrometer. Mass spectra were acquired in centroid mode over a range of 150-1500 *m/z* with source parameters identical to HRMS.

Gene cloning and protein purification

DNA fragments encoding full-length or truncated proteins involved in myxoglucamide biosynthesis (MxgA, FAAl-PCP_{MxgA}, Ox_{MxgA}, MxgB, A_{MxgB}, PCP_{MxgB}, and MxgH) were amplified from cosmid D09 using Q5 High-Fidelity DNA polymerase (NEB) and cloned into pET28b via Gibson assembly,

yielding constructs with an N-terminal 6xHis-tag and TEV protease cleavage site. For site-directed mutagenesis of MxgH (H141, D146, and G147), pET28b was digested with *Nco*I and *Hind*III, and the *mxgH* gene was split into two fragments; mutations were introduced at the junction using primers listed in Table S21. All generated constructs were verified by Sanger sequencing (LGC Genomics GmbH).

Constructs were transformed into BL21(DE3) cells. Cells were grown overnight in LB medium with appropriate antibiotics at 37 °C, diluted 1:100 into fresh LB, and incubated at 37 °C until OD_{600} reached 0.4-0.6. Cultures were rapidly cooled to 16 °C by incubation at 4 °C for 30 min, and protein expression was induced with 0.1 mM IPTG for 16-20 h at 16 °C. To obtain active NRPS proteins or adenylation domains, the MbtH-like gene was amplified from MCy9003 genome DNA and constructed into pCDF1b plasmid. MbtH-like protein was co-expressed with MxgA, FAAL-ACP_{MxgA}, MxgB and A_{MxgB}, to stabilize the adenylation domains and enable amino acid activation[7].

Cells were harvested by centrifugation (6,000 rpm, 4 °C, 15 min) and resuspended in lysis buffer (20 mM Tris pH 8.0, 200 mM NaCl, 10% glycerol, 20 mM imidazole) supplemented with cOmplete EDTA-free protease inhibitor tablets (Roche) and lysed via two passages through a cell disrupter (30 kpsi, Microfluidics Corp.). Lysates were clarified by centrifugation (23,500 rpm, 30 min, 4 °C). Affinity chromatography and size-exclusion chromatography (SEC) were performed on an Äkta Go system. The supernatants were applied to a 5 mL Ni-NTA column (Qiagen) pre-equilibrated with lysis buffer. Columns were washed with 30 column volumes (CV) of lysis buffer and eluted with 5 CV elution buffer (lysis buffer containing 250 mM imidazole). The protein fractions were concentrated and buffer-exchanged into lysis buffer to a final volume of less than 5 mL, and further purified by SEC on a HiLoad 16/600 Superdex 200 pg column (GE Healthcare) equilibrated in running buffer (25 mM HEPES, pH 7.5, 150 mM NaCl, 1 mM DTT). Protein purity was assessed by SDS-PAGE and the concentration was determined by UV absorbance at 280 nm using the molar extinction coefficient calculated by ExPASy ProtParam (https://web.expasy.org/protparam/). Proteins were aliquoted, rapid-frozen, and stored at −80 °C until use.

Substrate specificity measurement using a malachite green phosphate assay

Substrate specificity of A_{MxgB} was assessed using a malachite green-based phosphate detection assay (Sigma-Aldrich, MAK307). Reactions (80 µL) were performed in phosphate-free buffer containing 50 mM Tris-HCl (pH 7.5), 100 mM NaCl, 10 mM $MgCl_2$, 0.05 U inorganic pyrophosphatase (Thermo, 0.1 U/µL), 2 µM purified His-tagged A_{MxgB}, and 10 µM individual proteinogenic amino acids. Reactions were initiated by the addition of ATP (0.25 mM) and incubated at 30 °C with shaking at 300 rpm for 1 h. Subsequently, 20 µL malachite green working reagent (Reagent A/Reagent B, 100:1, v/v) was added, and samples were incubated at room temperature for 30 min. Released inorganic phosphate was quantified by measuring absorbance at 620 nm according to the manufacturer's instructions.

***In vitro* loading experiments**

All substrate loading experiments were performed in reaction buffer containing 50 mM Tris-HCl (pH 8.0) and 10 mM $MgCl_2$. Conversion of apo proteins (MxgA, FAAL-ACP_{MxgA}, MxgB and PCP_{MxgB}, or PCP_{MxgB} alone) to their holo forms was carried out by incubating 25-50 µM protein with 2 µM MtaA and 1 mM CoA at 30 °C with shaking at 300 rpm for 30 min. Where further loading steps were

required, buffer exchange was performed using Amicon centrifugal filters (Merck Millipore) into 20 mM Tris-HCl (pH 8.0) and 150 mM NaCl.

For substrate loading, 4 µM holo-form carrier protein was incubated with 2 µM cognate adenylation domain (or full-length protein when indicated), 1 mM ATP, and 1 mM substrate at 30 °C for 3 h. Reactions were quenched by rapid freezing in liquid N_2 or, where indicated, buffer-exchanged into 50 mM Tris-HCl (pH 8.0) and 25 mM NaCl for subsequent assays.

Hydroxylation of PCP_{MxgB}-tethered L-glutamate by Ox_{MxgA} was performed by supplementing the reaction with 1 mM $(NH_4)_2Fe(SO_4)_2$, 1 mM ascorbate, and 3 µM MxgA or Ox_{MxgA} protein, depending on the experimental setup, and initiated by the addition of 1 mM α-KG, incubating at 30 °C for 1.5 h.

To test the transfer of MPA or MHA from MxgA to β-OH-L-Glu-*S*-PCP_{MxgB}, 10 µM L-Glu-*S*-PCP_{MxgB} or 10 µM L-Glu-*S*-(PCP_{MxgB} and MxgB) was incubated with 10 µM MPA/MHA-*S*-MxgA, supplemented with 1 mM $(NH_4)_2Fe(SO_4)_2$, 1 mM ascorbate and 1 mM α-KG, and incubated at 30 °C for 1.5 h. Reaction products were analyzed by direct intact protein UPLC-ESI-MS analysis as described below.

Direct intact protein UPLC-ESI-MS analysis

Intact proteins were analyzed using an UltiMate 3000 UPLC system coupled to a maXis4G Q-ToF mass spectrometer with an Apollo II ESI source in positive mode. Samples were separated on an Aeris Widepore XB-C8 column (3.6 µm, 150 × 2.1 mm; Phenomenex) at 0.3 mL/min and 45 °C using eluent A: deionized water + 0.1% FA and eluent B: acetonitrile + 0.1% FA. The gradient was as follows: 2% B for 30 s, linear gradient up to 75% B over 10 min, held at 75% B for 3 min, followed by a return to 2% B within 30 s and re-equilibration at 2% B for 3 min. The LC flow was split to 75 µL/min before entering the mass spectrometer. Mass spectra were acquired in centroid mode over an *m/z* range of 150–2500 at a 2 Hz full scan rate. Mass spectrometry source parameters were set to 500 V as endplate offset, 4000 V as capillary voltage, 1.1 bar nebulizer gas pressure, 6 L/min dry gas flow, and 180 °C dry temperature. Protein masses were deconvoluted using the Maximum Entropy deconvolution algorithm in Compass DataAnalysis (Bruker).

Enzymatic assays for MxgH and variants

Enzymatic assays were carried out in 50 µL reaction mixtures containing 10 µM acceptor substrate myxoglucamide C, 200 µM donor substrate(s) (acetyl CoA, *n*-propionyl CoA, *n*-butyryl CoA, *n*-hexanoyl CoA, *n*-heptanoyl CoA, *n*-octanoyl CoA, or *n*-decanoyl CoA for single CoA assays; combinations of acetyl CoA, *n*-propionyl CoA, *n*-butyryl CoA at a total concentration of 200 µM with molar ratios of 1:1, or 1:1:1), 50 mM Tris buffer (pH 8.0), and 25 mM NaCl. The reactions were initiated by the addition of 1 µM MxgH and quenched by the addition of the same volume of methanol after incubating overnight at 30 °C, and mixed thoroughly. After centrifugation, the supernatants were analyzed by UPLC-HRMS or UPLC-HRMS^2.

Activity assays were carried out in 30 µL of reaction mixture containing 10 µM acceptor substrate myxoglucamide C and 200 µM donor substrate fatty acyl CoAs, 50 mM Tris buffer (pH 8.0), 25 mM NaCl and initiated by the addition of 15 nM MxgH or MxgH variants. The reactions were quenched with the same volume of methanol after incubation at 30 °C for 30 min, and well-mixed. After centrifugation, the supernatants were analyzed using an amaZon speed 3D ion trap MS system

(Bruker) with an Apollo II ESI source. The experiments were performed in duplicate in three independent replicates.

Phylogenetic tree

Besides the C and E domains from the *mxg* pathway, the remaining 255 C and E domains for building the phylogenetic tree were retrieved from the NaPDoS database[8], literature[9–12] and MxgH analogs (WP_120613649, WP_120643778, WP_206726902, WP_223754787, WP_253984509). The sequences were aligned using Clustal Omega with the BLOSUM matrix. The phylogenetic tree was constructed using the Jukes-Cantor distance model and neighbor-joining method. Abbreviations for different functions of the C domains: $^{L}C_{L}$, condensation domains catalyze the condensation between a L-aminoacyl/peptidyl-PCP donor and a L-aminoacyl-PCP acceptor; $^{D}C_{L}$, condensation domains catalyze the condensation between a D-aminoacyl/peptidyl-PCP donor and a L-aminoacyl-PCP acceptor; Dual, condensation domains with both condensation and epimerization; Starter, condensation domains catalyze acylation of the first amino acid with a fatty acid, polyketide, or other molecules; Cyclization, condensation domains that perform condensation and cyclization of cysteine, serine or threonine residues; C_{modAA}, modification of the incorporated amino acid; Interface, recruitment of other functional enzymes such as oxygenases; Ester bond formation, catalysis of ester bond formation; Epimerization, epimerization activity.

Isolation and structure elucidation of myxoglucamides

Myxoglucamide A-D

All measurements to analyze the masses of myxoglucamides during purification were performed by low resolution LC-MS measurements using a 6 min or 9 min gradient.

The molecules were isolated from a 9 L culture of MCy9003/Mutant in CyS media. The cells and the adsorber resin XAD-16 were extracted three times with 800 mL acetone/MeOH 1:1. The solvents were poured through a funnel stuffed with glass wool and the solvents were evaporated. The dried residues were dissolved in 500 mL H_2O and extracted twice with 400 mL ethyl acetate in a separatory funnel. Myxoglucamides were mostly found in the organic layer. The organic layer was dried *in vacuo* and further fractionated using reverse phase flash chromatography. The following setup was used:

Biotage with a 25 g Snapfit column, Versa Flash Spherical C18 silica 45-75 µm 70 A, Solvent System: A=H_2O + 0.1 % FA; B= MeOH + 0.1 FA; A flow rate of 20 mL/min was used and 50 mL were collected per tube. The column was equilibrated with 3 column volumes (CV) 95:5 Solvent A/B. The sample was loaded on the column using iSolute beads. The gradient 4 CV 95:5 solvent A/B; 40 CV linear increases from 5-100 % solvent B; 5 CV 100 % solvent B was used.

An aliquot of every fraction (including the waste from the flushes) was taken and measured by UPLC-MS to screen for the myxoglucamides. Fractions containing myxoglucamides were dried and further purified by HPLC. We used Dionex Ultimate 3000 coupled with Bruker High Capacity Trap mass spectrometer (HCT); Column: Luna C18(2) 250 x 10 mm, 5 µm; flow rate 5 mL/min and column temperature 40 °C with H_2O + 0.1 % FA as eluent A and ACN + 0.1% FA as eluent B. The gradient, 0-2 min 5 % eluent B; 2-4 min linear increase of eluent B to 43 %; 4-24 min linear increase of eluent B to 46 %; 24-25 min linear increase of eluent B to 95 % eluent B; 25-28 min 95 % eluent

B 28-29 min linear decrease of eluent B to 5 %; 29-33 min re-equilibration with 5 % eluent B, was applied. Myxoglucamide B was collected from 10.8-11.1 min; myxoglucamide D was collected from 13.0-13.4 min; myxoglucamide A was collected from 14.4-14.85 min and myxoglucamide C was collected from 18.0-18.7 min. The collected fractions were dried and dissolved in MeOH.

Myxoglucamide A was isolated as colorless amorphous solid. *hr*Mass: 645.40 $[M+H]^+$; specific optical rotation $[\propto]_D^{20}$ -10.4 $°g^{-1}mL\ dm^{-1}$; UV max 197, 241 nm (UV data extracted from LC-MS-UV measurement using a Dionex ultimate 3000 HPLC system equipped with a DAD UV-VIS detector running with H_2O + 0.1 % FA and ACN + 0.1 % FA); IR: 921, 1017, 1074, 1158, 1366, 1533, 1653, 1723, 2854, 2924, 3296 cm^{-1}.

Myxoglucamide B was isolated as colorless amorphous solid. *hr*Mass: 647.41 $[M+H]^+$; specific optical rotation $[\propto]_D^{20}$ 0.9 $°g^{-1}mL\ dm^{-1}$; UV max: 195 nm (UV data extracted from LC-MS-UV measurement using a Dionex ultimate 3000 HPLC system equipped with a DAD UV-VIS detector running with H_2O + 0.1 % FA and ACN + 0.1 % FA) IR: 921, 1018, 1073, 1159, 1405, 1533, 1594, 1635, 2854, 2925, 3287 cm^{-1}.

Myxoglucamide C was isolated as colorless amorphous solid. *hr*Mass: 659.41 $[M+H]^+$; specific optical rotation $[\propto]_D^{20}$-3.5 $°g^{-1}mL\ dm^{-1}$; UV max: 193, 241 nm (UV data extracted from LC-MS-UV measurement using a Dionex ultimate 3000 HPLC system equipped with a DAD UV-VIS detector running with H_2O + 0.1 % FA and ACN + 0.1 % FA); IR: 920, 1018, 1074, 1160, 1366, 1457, 1533, 1652, 2855, 2925 cm^{-1}.

Myxoglucamide D was isolated as colorless amorphous solid. *hr*Mass: 661.43 $[M+H]^+$; specific optical rotation $[\propto]_D^{20}$ 6.8 $°g^{-1}mL\ dm^{-1}$; UV max: 199 nm (UV data extracted from LC-MS-UV measurement using a Dionex ultimate 3000 HPLC system equipped with a DAD UV-VIS detector running with H_2O + 0.1 % FA and ACN + 0.1 % FA) IR: 1019,1074, 1459, 1533, 1638, 2925 cm^{-1}.

Myxoglucamide E-G

The molecules were isolated from 60 x 50 mL cultures of DK1622/BiPD09 in CTT media. The cells and the adsorber resin XAD-16 were extracted three times with 600 mL acetone/MeOH 1:1. The solvents were poured through a funnel stuffed with glass wool and the solvents were evaporated. The dried residues were defatted by liquid/liquid partitioning using 2 x 400 mL n-hexane and MeOH with 10 % H_2O. The MeOH layer was dried *in vacuo* and dissolved in 500 mL H_2O, acidified to pH 3-4 and extracted twice with 400 mL ethyl acetate in a separatory funnel. Myxoglucamides were mostly present in the ethyl acetate layer. This layer was dried *in vacuo* and further fractionated using flash chromatography. The following setup was used:

Biotage with a 25 g Snapfit column filled with silica gel (Sigma, 60741). The solvent system was A=n-hexane + 0.1 % FA; B= ethyl acetate + 0.1 FA; C = MeOH + 0.1 FA. A flow rate of 25 mL/min was used and 45 mL were collected per tube. The column was equilibrated with 3 column volumes (CV) 65:35 Solvent A/B. The sample was loaded on the column using iSolute beads. The gradient was initiated with a linear increase to 100% solvent B over 20 CV, followed by 3 CV 100 solvent B and a linear increase to 50 % solvent C over 15 CV.

An aliquot of every fraction (including the waste from the flushes) was taken and measured by UPLC-MS to screen for the myxoglucamides. Fractions containing myxoglucamides were dried and further purified by HPLC. We used Dionex Ultimate 3000 coupled single quadrupole mass spectrometer manufactured by Thermo Scientific. Column: Waters Xbridge Peptide BEH C18 250

x 10 mm, 5 µm; flow rate 5 mL/min and column temperature 40 °C with H_2O + 0.1 % FA as eluent A and ACN + 0.1% FA as eluent B. The gradient: 0-2 min 5 % eluent B; 2-4 min linear increase of eluent B to 46 %; 4-24 min linear increase of eluent B to 60 %; 24-25 min linear increase of eluent B to 95 % eluent B; 25-28 min 95 % eluent B; 28-29 min linear decrease of eluent B to 5 %; 29-33 min re-equilibration with 5 % eluent B, was applied. Myxoglucamide E was collected from 17.3-18.0 min; myxoglucamide F was collected from 23.1-23.6 min; myxoglucamide G was collected from 15.2-16.1 min. The collected fractions were dried and dissolved in MeOH.
Myxoglucamide E was isolated as a colorless amorphous solid. *hr*Mass: 715.44 $[M+H]^+$
Myxoglucamide F was isolated as a colorless amorphous solid. *hr*Mass: 715.44 $[M+H]^+$
Myxoglucamide G was isolated as a colorless amorphous solid. *hr*Mass: 771.46 $[M+H]^+$

Intermediate **8**

The molecules were isolated from a 5 L culture of DK1622/Ox_{MxgE}-mutants in CyH medium. The crude extracts were treated using Biotage Isoleta One flash chromatography purification instrument with a Biotage® Sfär C18D - Duo300Å 20µm column (66 mL). Solvent system A=H_2O, B=Methanol. The flow rate of 50 mL/min was used. The column was firstly equilibrated with 3 CV of 95: 5 solvent A/B, then the samples were loaded on the column by using iSolute beads. Finally, the gradient 2 CV 95: 5 solvent A/B; 35 CV linear increases from 5-100% solvent B; 3 CVs 100% solvent B were performed to get fractions. An aliquot of each fraction was taken and measured by LC-MS analysis to screen for the target compounds. Bruker HCT ion trap with Dionex Ultimate HPLC was used to isolate target compound. The purification procedure: solvent system A=H_2O, B=Acetonitrile; The flow rate 5 mL/min; Gradient: 22% ACN to 53%ACN 22min, 53% ACN to 95%ACN 1min, 95% ACN 3min, 95% ACN to 22% ACN 1min, 22% ACN 3min. The compound was collected from 23.2-23.5 min.

Stereochemistry analysis

Marfey´s analysis

Between 50 and 200 µg of compound were placed in a 2 mL glass vial. 100 µL of 6N HCl were added and the sample was heated to 110 °C for 45 min under nitrogen atmosphere. In the next step, the sample was dried and dissolved in 110 µL H_2O. 50 µL of the sample were transferred into two 1.5 mL plastic tubes. 20 µL 1N $NaHCO_3$ solution (pH 9) was added to each tube followed by addition of 1 % Marfey´s reagent in acetone (D-1-Fluoro-2,4-dinitrophenyl-5-leucine amide (D-FDLA) and L-FDLA). The reaction mixture was incubated for 2 h under constant shaking at 700 rpm. The reaction was stopped by addition of 10 µL 2 N HCl. After the reaction was stopped, 300 µL ACN were added, the sample was centrifuged and an aliquot of the supernatant was analyzed by *hr*-LC-MS using a maXis4G analytical setup (Table S6). Standards were handled in a similar manner skipping the hydrolysis with HCl. The following gradient was used: Column: ACQUITY BEH C18 (100 x 2.1 mm ,1.7 µm, 130 Å), flow rate 0.6 mL/min, and column temperature 45°C with H_2O + 0.1 % FA as eluent A and ACN + 0.1% FA as eluent B. Gradient: 0-1 min linear increase of eluent B to 10 %; 1-15 min linear increase of eluent B to 35%; 15-22 min linear increase of eluent B to 55%. The detection was performed in positive ion mode.

Ozonolysis

100-200 µg of each myxoglucamide derivative was dissolved separately in 5-10 mL MeOH and placed in a two-necked flask and cooled to -70 °C using an acetone bath with dry ice. Ozone was introduced into the solution for 5 min and 1 mL 35 % H_2O_2 were added to the mixture afterwards. The MeOH was removed *in vacuo*, the reaction mixture was dried under nitrogen flow and analyzed using Marfey's method as described above. L-aspartate was used as the standard amino acid (Table S7).

J-based analysis of myxoglucamides

To determine the configuration of the hydroxyl group at position 21 in myxoglucamide D, a *J*-based analysis was conducted according to Bifulco et al.[13] The ^3J H,H coupling constants were extracted from the proton spectrum and from a *J*-resolved COSY. H,C coupling constants were obtained from dipsi 2 and HSQMBC 2D NMR experiments (Figures S31-S35). Analogous to Bifulco et al.,[13] the molecule of interest was divided into two segments. Based on the obtained values and neglecting the ^3J H-20b,C-22 coupling constants, the relative configuration between C-19 and C-21 in myxoglucamide D can be assigned as *syn* (Table S8).

Acid hydrolysis and Mosher analysis

Acid hydrolysis of myxoglucamide A

3.7 mg of myxoglucamide A were dissolved in 1 N HCl and was incubated at 100 °C for 26 h. The hydrolysis mixture was cooled to room temperature and extracted first with *n*-hexane followed by ethyl acetate. The remaining aqueous layer was mixed with MeOH and dried under nitrogen flow**.** 0.6 mg of n-hexane extract was obtained. NMR analysis showed that the n-hexane layer contained 13-hydroxy-14-methylpentadecanoic acid (HMPA) as hydrolysis product (Table S9). The aqueous MeOH extract (yield: 1.2 mg) contained the sugar moiety and traces of amino-butyric acid as confirmed by NMR analysis. The optical rotation of the MeOH extract was $[\alpha]_D^{20}$17 ° g $^{-1}$ mL dm^{-1}. The positive optical rotation indicates that the glucose moiety is D-glucose.

Determination of the stereogenic center in HMPA

The (*S)*-MTPA ester was prepared as follows:

100 µg of HMPA (0.37 µM) was dissolved in 120 µL pyridine-d_5 and 1.5 µL *(R)*-MTPA-Cl (9.6 µM) were added to the reaction mixture. The reaction progress was observed *via* LC-MS. The expected product mass of 487.30 [M-H]$^-$ was observed in ESI negative mode. After 3.5 h, 450 µL pyridine-d_5 were added and the reaction mixture was transferred into a NMR tube.

The *(R)*-MTPA ester was prepared as follows:

120 µg of HMPA (0.44 µM) was dissolved in 120 µL pyridine-d_5 and 1.5 µL *(S)*-MTPA-Cl (9.6 µM) were added to the reaction mixture. The reaction progress was observed *via* LC-MS. The expected product mass of 487.30 [M-H]$^-$ was observed in ESI negative mode. After 5 h, 450 µL pyridine-d_5 were added and the reaction mixture was transferred into a NMR tube. The reaction mixture was analyzed without further purification by 2D NMR experiments (HSQC). Analysis of the shift

difference Δδ(S-R) yielded a certain difference distribution. An analysis according to Hoye et al.[14] suggested an (*S)*-configuration of the hydroxyl group at C-4.

Acid hydrolysis of myxoglucamide C

6.4 mg of myxoglucamide C was dissolved in 1 N HCl and incubated at 100 °C for 24 h. The hydrolysis mixture was cooled to room temperature and extracted with *n*-hexane followed by ethyl acetate. The remaining aqueous layer was mixed with MeOH and dried under nitrogen flow. All extracts were analyzed by NMR to monitor hydrolysis products. The *n*-hexane layer contained 14-hydroxy-15-methylhexadecanoic acid (HMHA) as hydrolysis product (Table S12).

The aqueous MeOH extract (yield: 2.4 mg) contained the sugar moiety and traces of amino-butyric acid as confirmed by NMR analysis (Table S13). The optical rotation of the MeOH extract was $[\propto]_D^{20}$ 19.6 ° g $^{-1}$ mL dm^{-1}. The positive optical rotation indicates that the glucose moiety is D-glucose.

Determination of the stereogenic center of HMHA

The (*S)*-MTPA ester was prepared as follows:

358 µg of HMHA (1.26 µM) was dissolved in 250 µL pyridine-d_5 and 1.5 µL *(R)*-MTPA-Cl (9.6 µM) were added to the reaction mixture. The reaction progress was observed *via* LC-MS. The expected product mass of 501.20 $[M-H]^-$ was observed in ESI negative mode. After 10 min 400 µL pyridine-d_5 were added and the reaction mixture was transferred into a NMR tube.

The *(R)*-MTPA ester was prepared as follows:

397 µg of HMHA (1.39 µM) was dissolved in 250 µL pyridine-d_5 and 1.3 µL *(S)*-MTPA-Cl (8.3 µM) were added to the reaction mixture. The reaction progress was observed *via* LC-MS. The expected product mass of 501.20 $[M-H]^-$ was observed in ESI negative mode. After 10 min, 400 µL pyridine-d_5 were added and the reaction mixture was transferred into a NMR tube. Both reaction mixtures were analyzed without further purification by 2D NMR experiments (HSQC). Analysis of the shift difference Δδ(S-R) yielded a certain difference distribution. According to Hoye et al.,[14] the hydroxyl group at C-5 was assigned an (*S)*-configuration.

The general information about the NMR spectrometers, probes and pulse sequences

1D and 2D NMR spectra were acquired in CD_3OD using Bruker Ascend 700 (^{1}H NMR data at 700 MHz, ^{13}C NMR data at 175 MHz) and Bruker UltraShield 500 (^{1}H NMR data at 500 MHz, ^{13}C at 125 MHz) spectrometers equipped with TCI 5-mm probe heads. The chemical shift values are reported in ppm and coupling constant values (*J*) are reported in Hertz (Hz). Standard pulse programs were used to collect the HSQC and HMBC NMR data. HSQMBC NMR experiments were optimized for $^{2,3}J_{H,C}$ value of 6 Hz. To increase sensitivity, the NMR data were collected using 5-mm Shigemi tubes (Shigemi Inc., Allison Park, PA 15101, USA). The $J_{H,C}$ values were determined based on the HSQMBC data collected with a resolution of 8k data points for F2 axis and 512 data points for F1 axis. The data were analyzed using Bruker TopSpin 3.6 software.

Supporting Figures

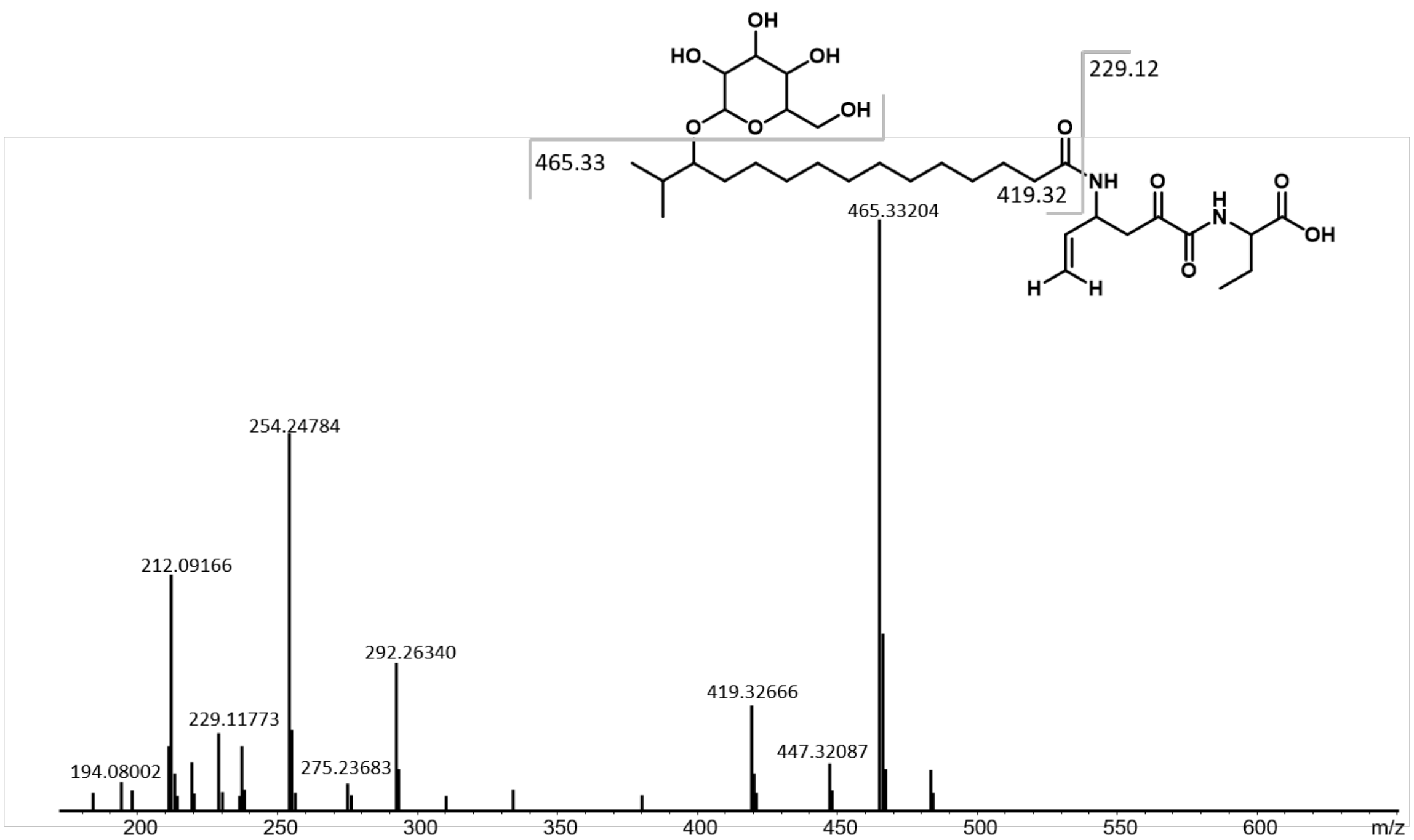

Figure S1. HRMS² spectrum and observed fragments of myxoglucamide A

MS² spectrum is extracted from UPLC-HRMS measurement of an extract of MCy9003/Mutant.

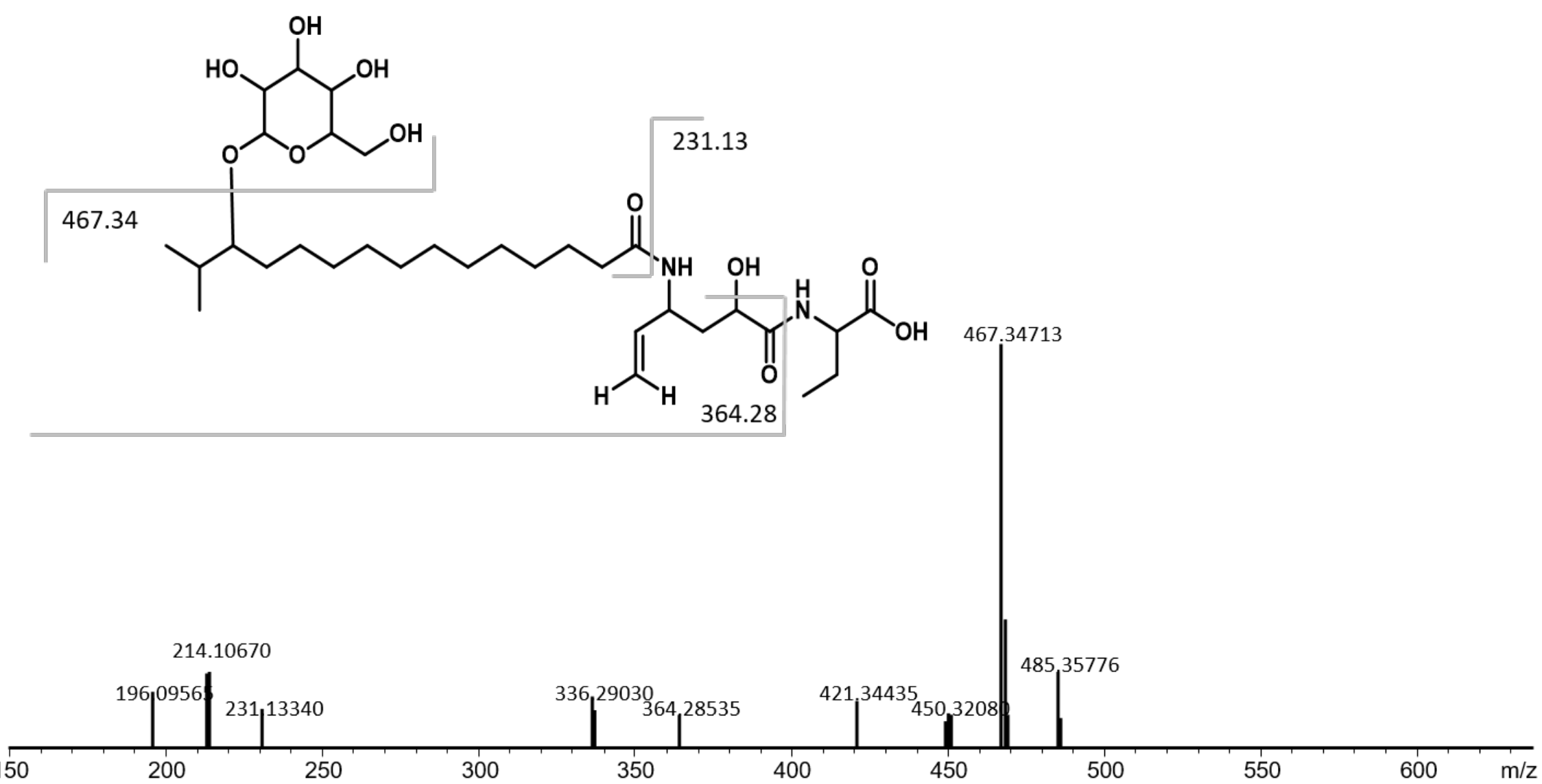

Figure S2. HRMS^2 spectrum and observed fragments of myxoglucamide B

MS^2 spectrum is extracted from UPLC-HRMS measurement of an extract of MCy9003/Mutant.

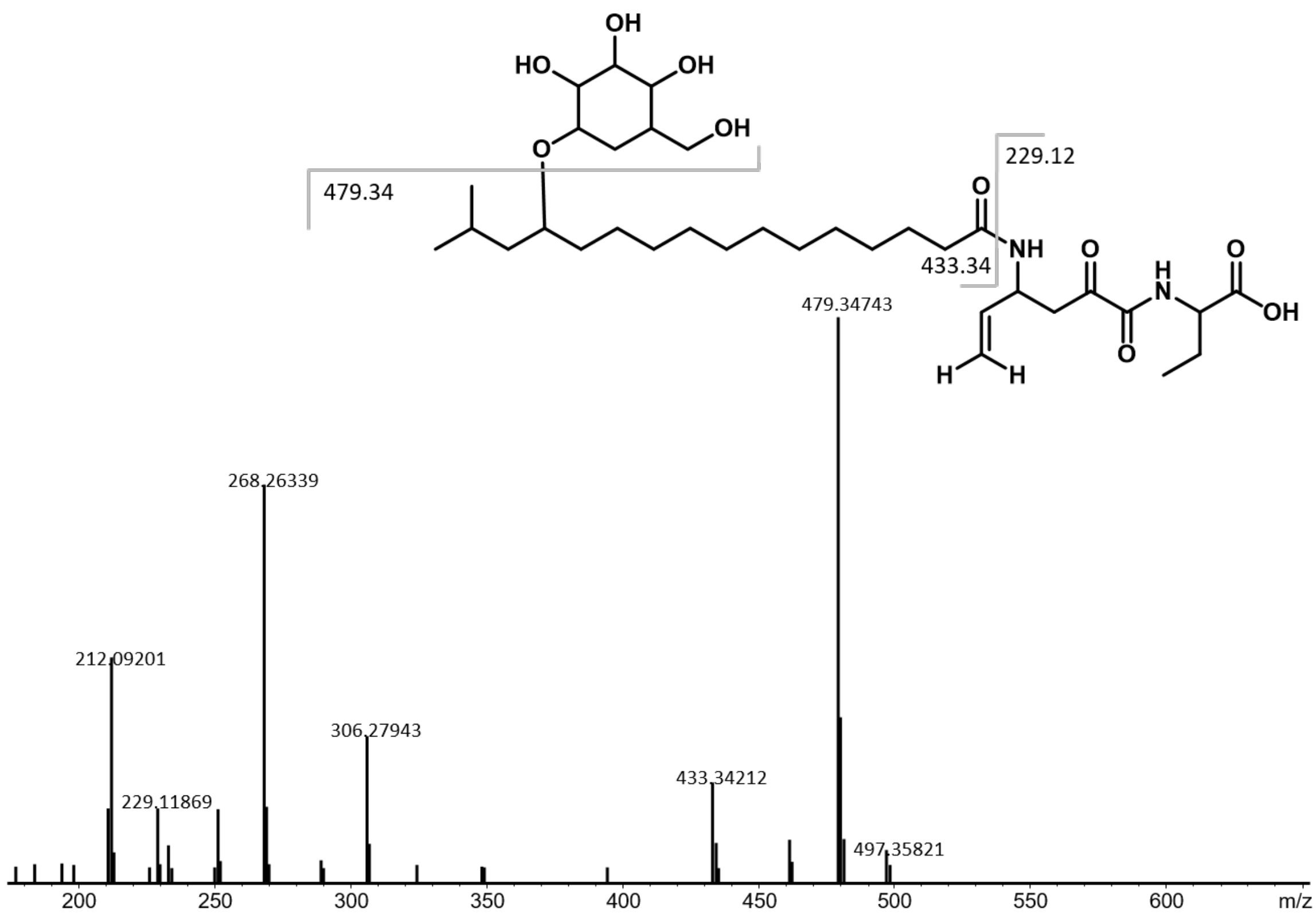

Figure S3. HRMS^2 spectrum and observed fragments of myxoglucamide C

MS^2 spectrum is extracted from UPLC-HRMS measurement of an extract of MCy9003/Mutant.

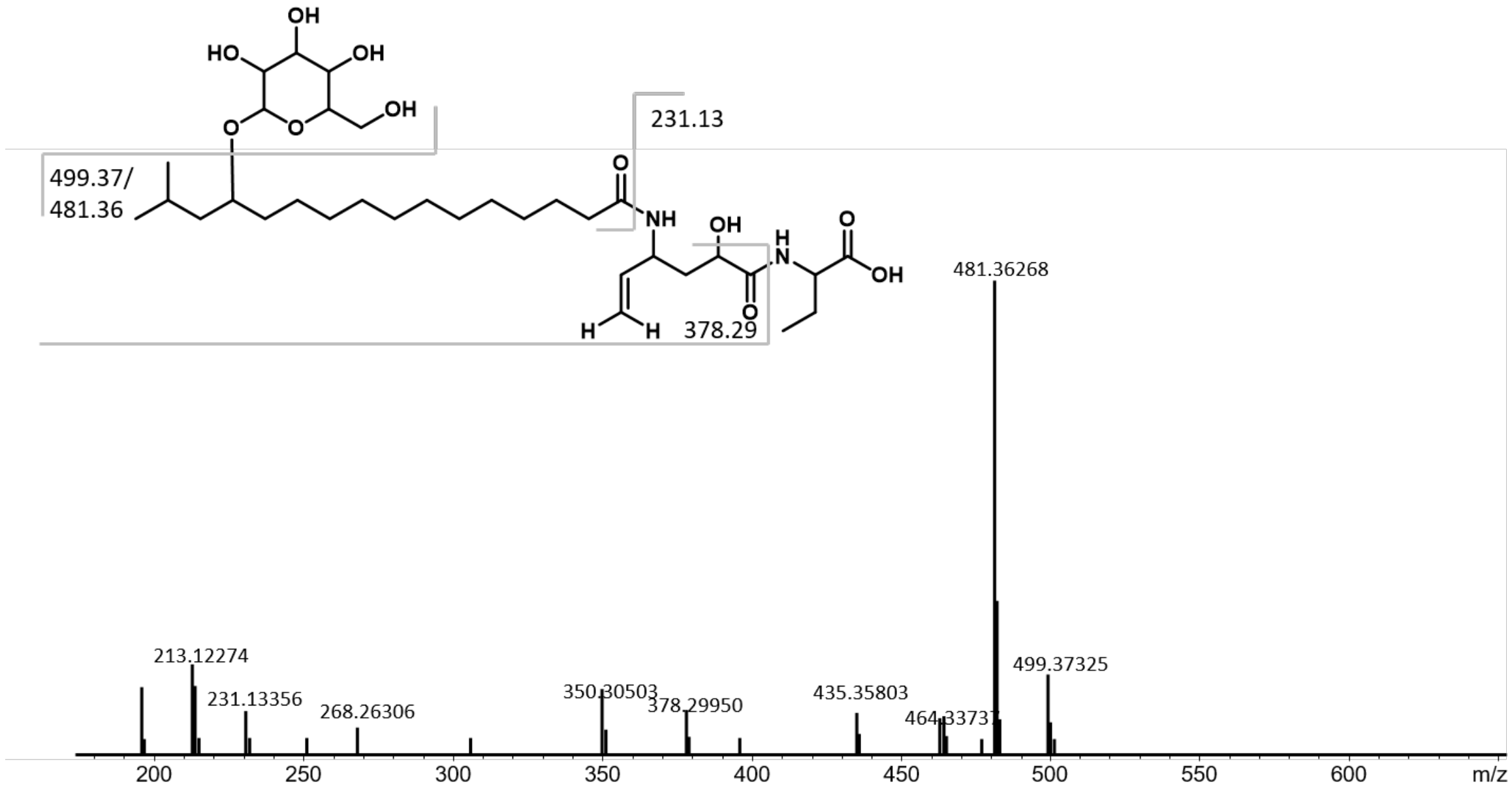

Figure S4. HRMS^2 spectrum and observed fragments of myxoglucamide D

MS^2 spectrum is extracted from UPLC-HRMS measurement of an extract of MCy9003/Mutant.

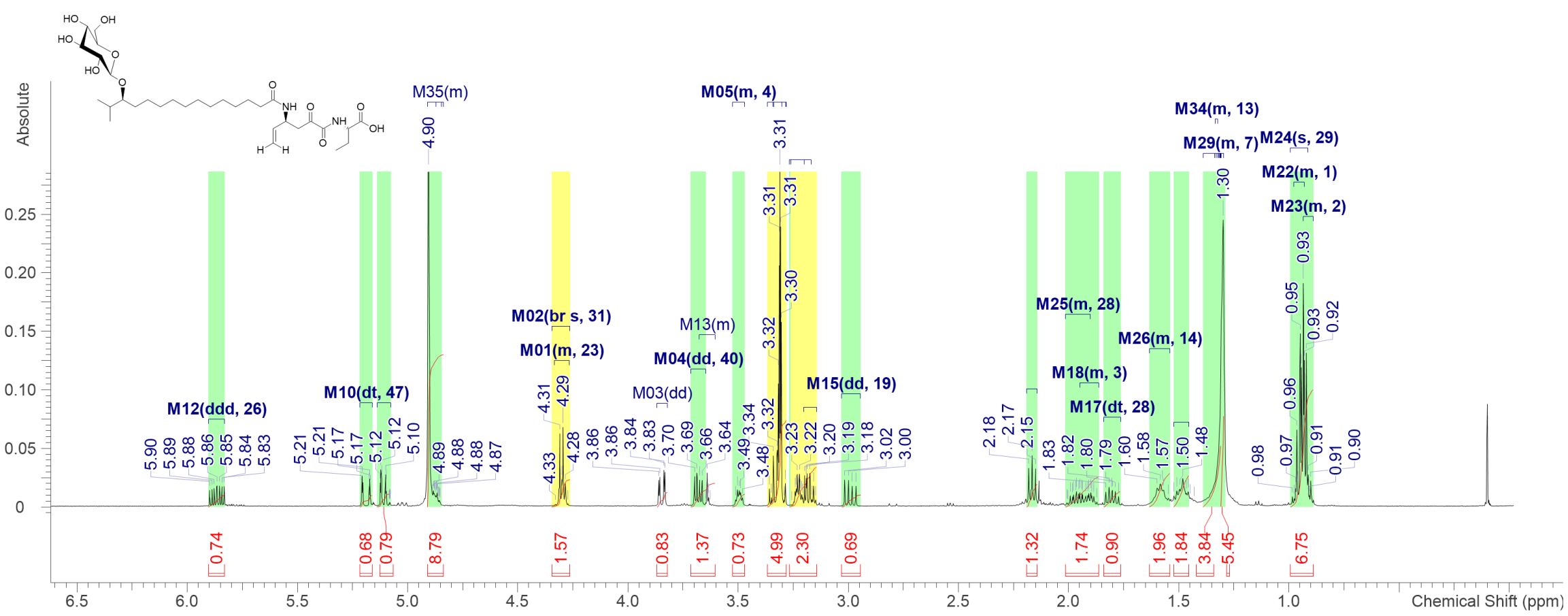

Figure S5. ^{1}H spectrum of myxoglucamide A acquired in CD_3OD at 500 MHz

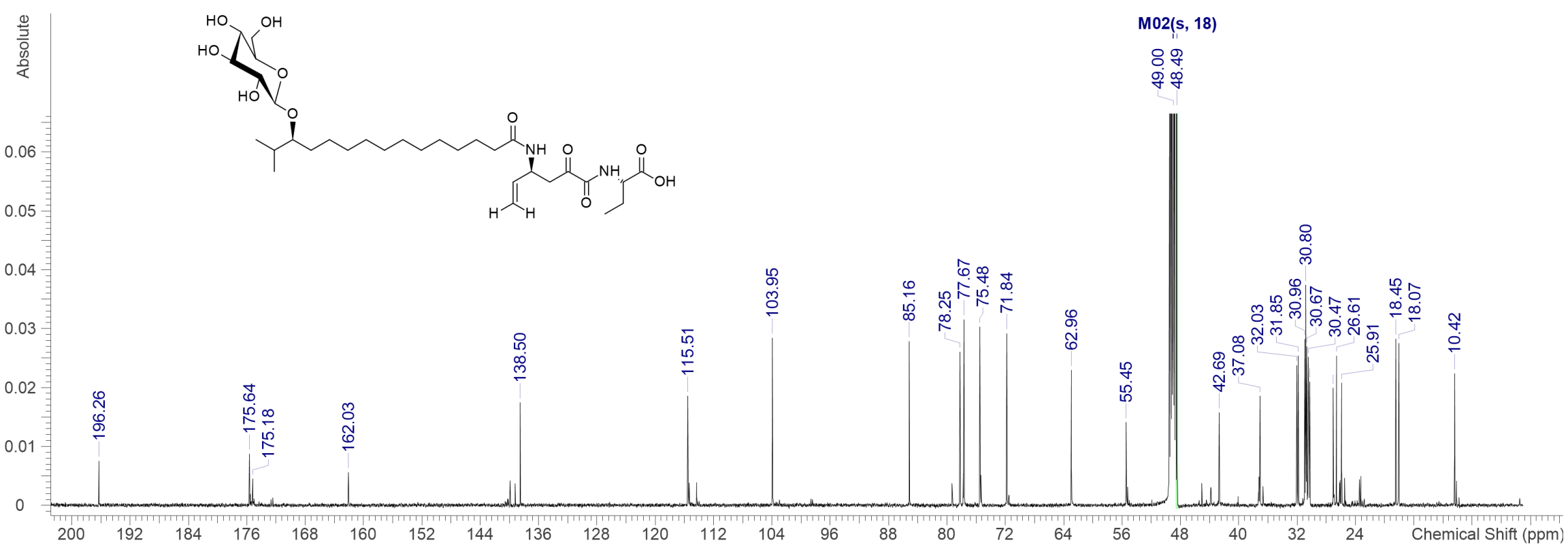

Figure S6. ^{13}C spectrum of myxoglucamide A acquired in CD_3OD at 125 MHz

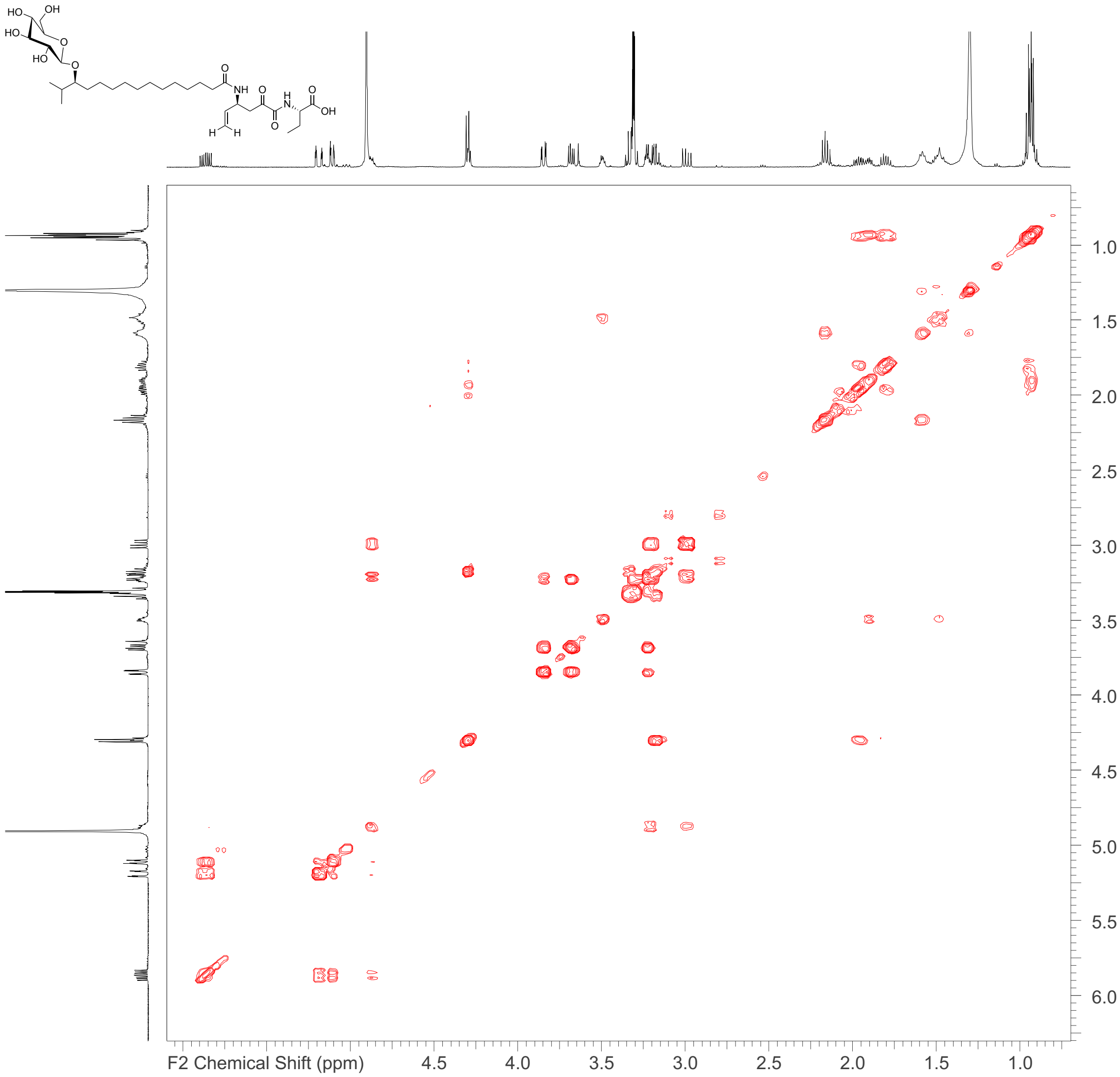

Figure S7. H,H COSY spectrum of myxoglucamide A acquired in CD_3OD at 500 MHz

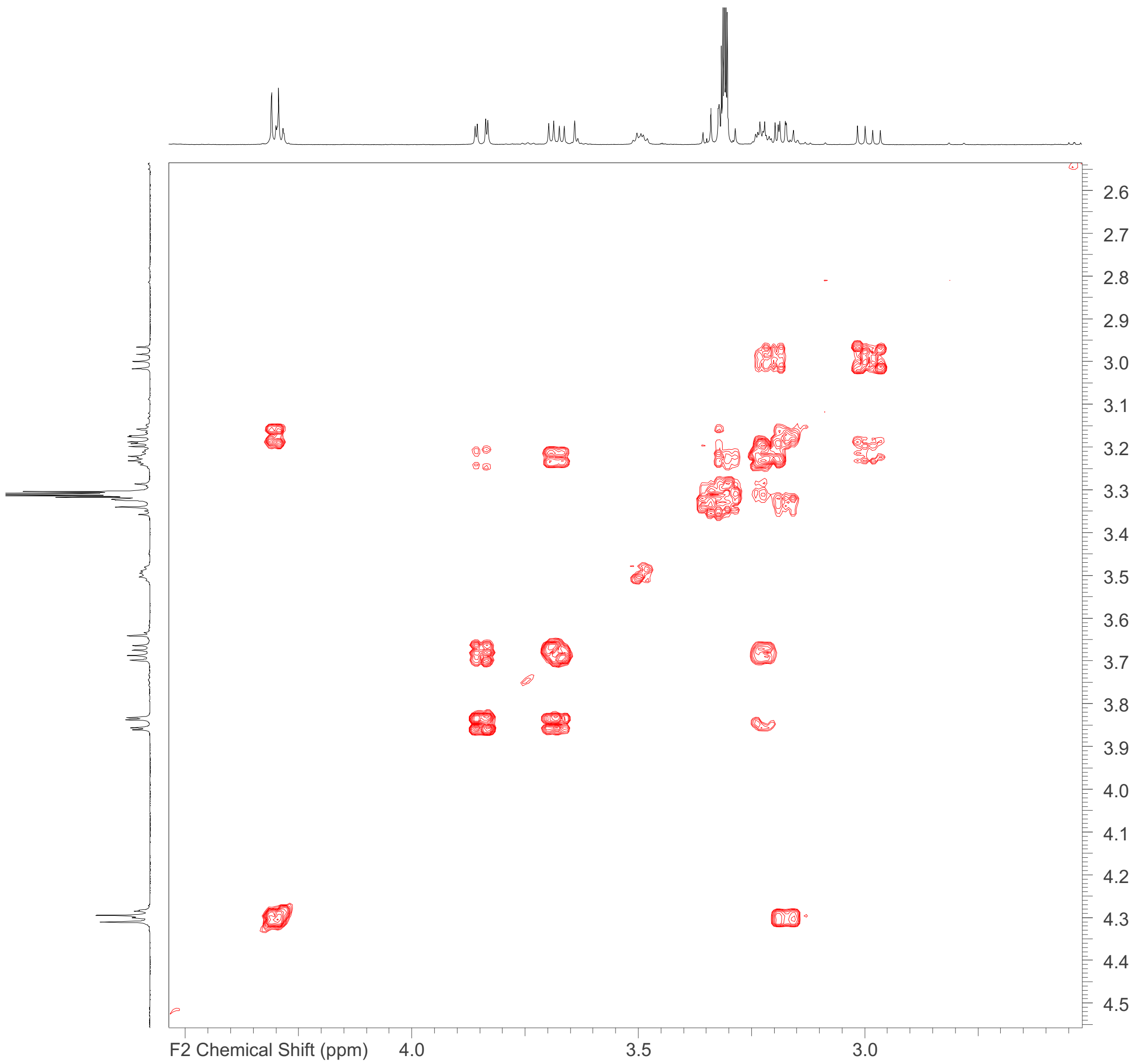

Figure S8. Enlarged H,H COSY spectrum of myxoglucamide A acquired in CD_3OD at 500 MHz

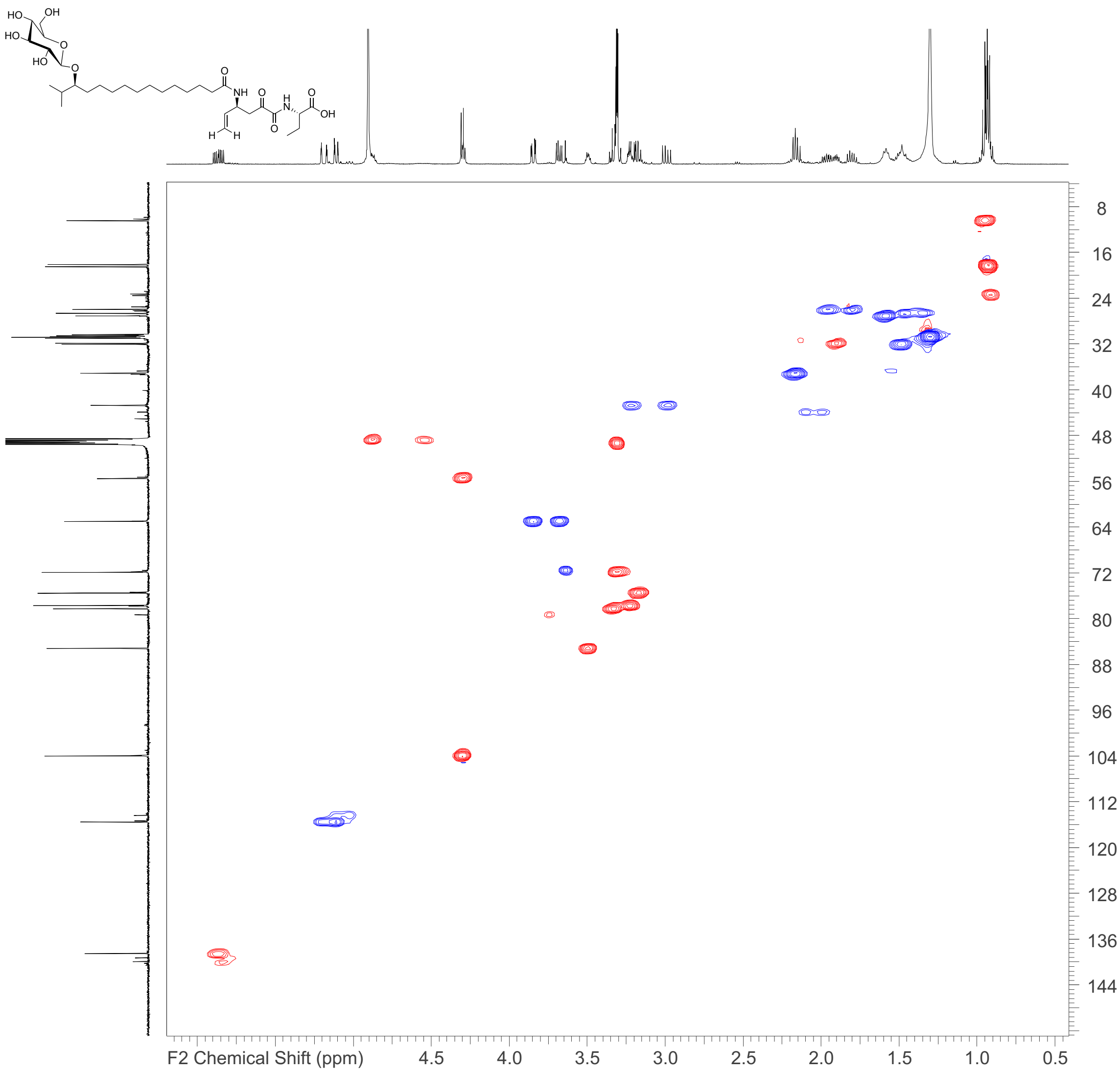

Figure S9. HSQC spectrum of myxoglucamide A acquired in CD_3OD at 500/125 MHz

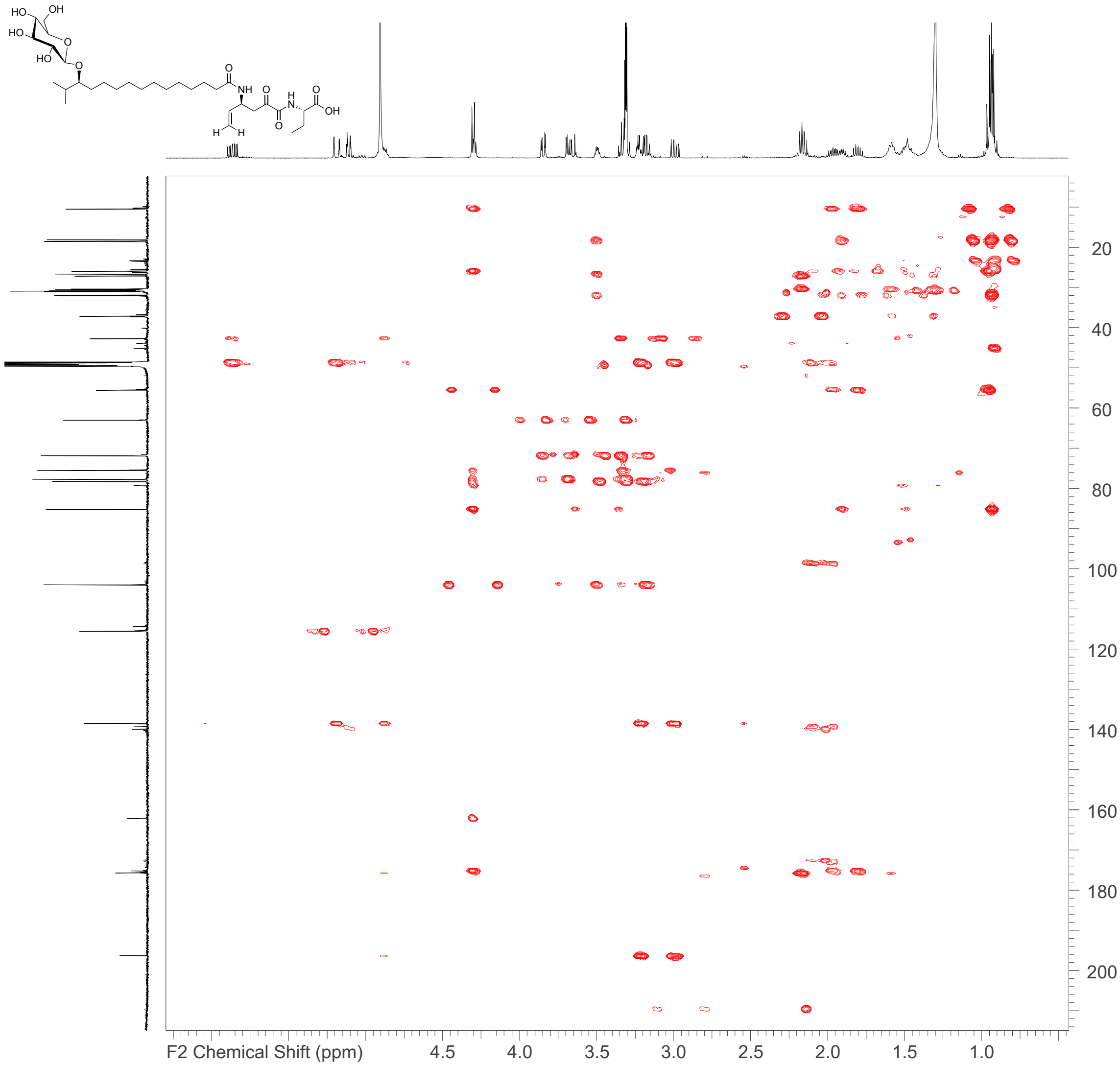

Figure S10. HMBC spectrum of myxoglucamide A acquired in CD_3OD at 500/125 MHz

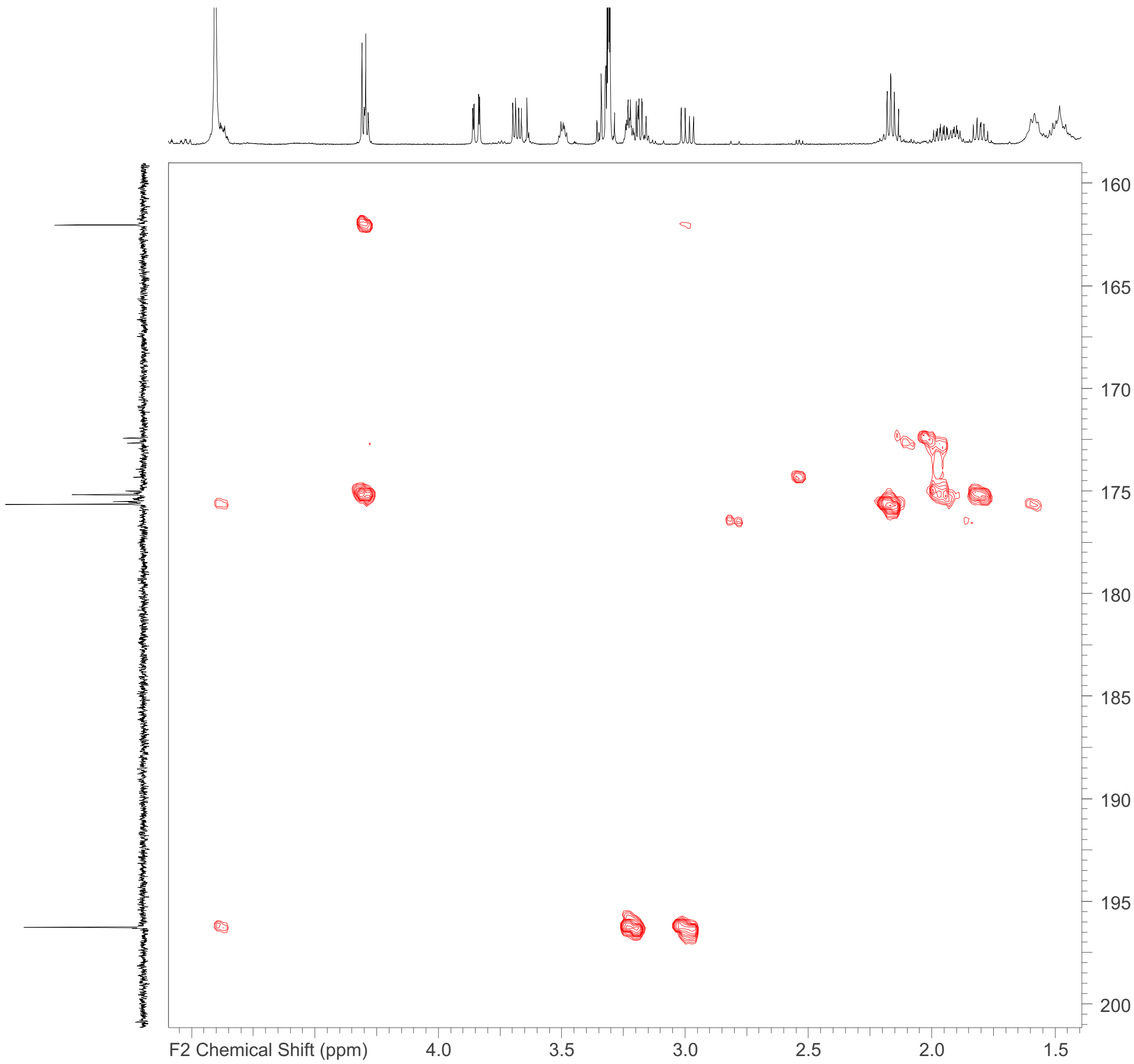

Figure S11. Enlarged HMBC spectrum of myxoglucamide A acquired in CD_3OD at 500/125 MHz

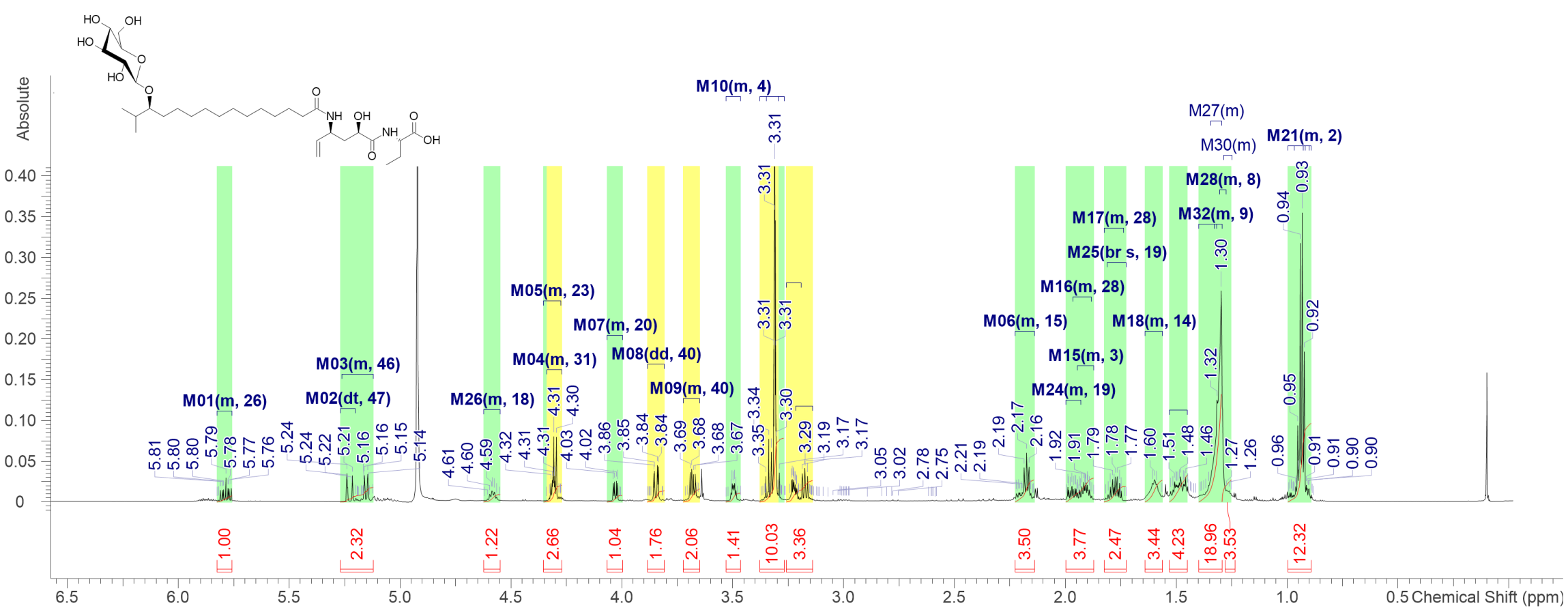

Figure S12. ^{1}H spectrum of myxoglucamide B acquired in CD_3OD at 700 MHz

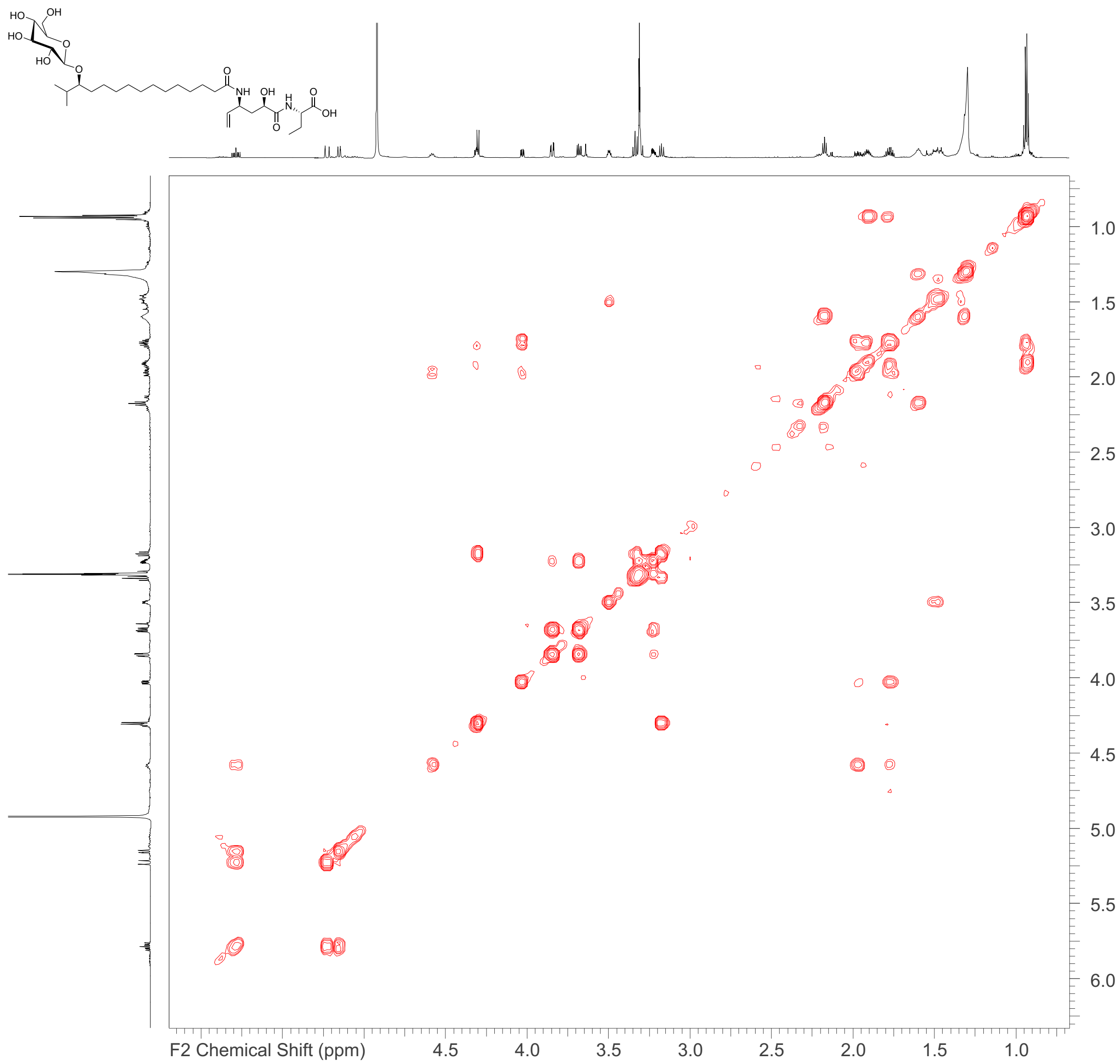

Figure S13. H,H COSY spectrum of myxoglucamide B acquired in CD_3OD at 700 MHz

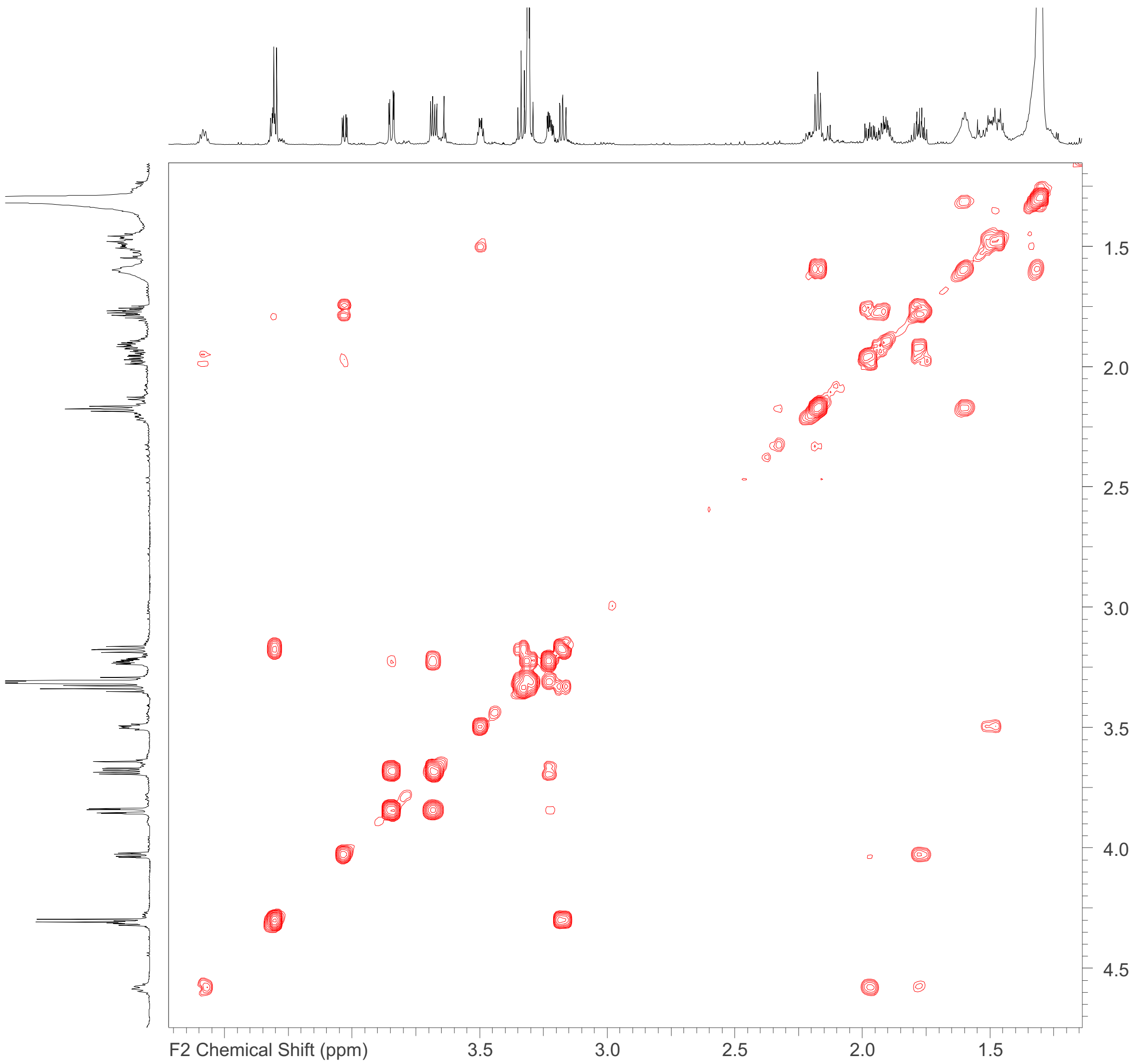

Figure S14. Enlarged H,H COSY spectrum of myxoglucamide B acquired in CD_3OD at 700 MHz

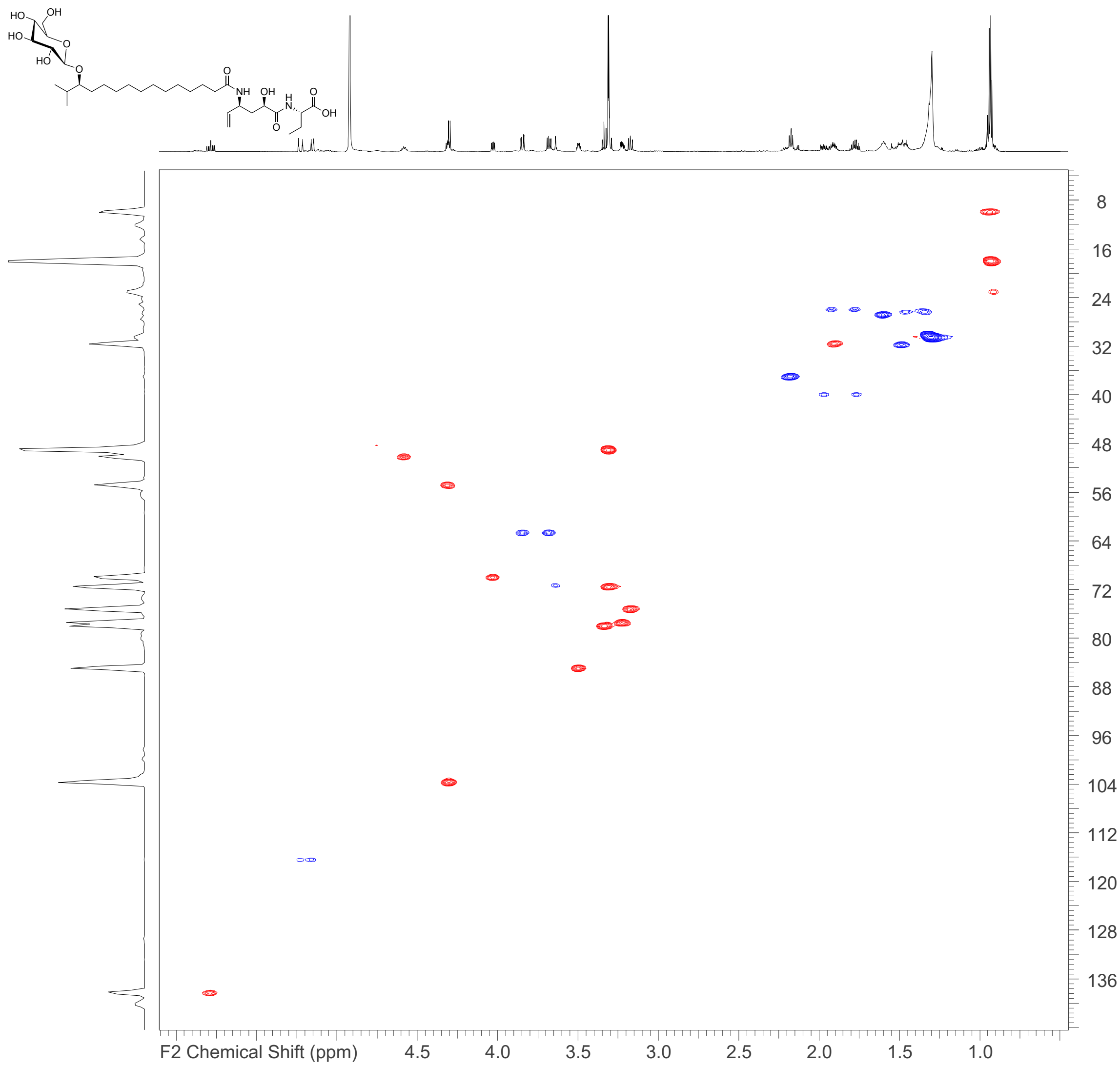

Figure S15. HSQC spectrum of myxoglucamide B acquired in CD_3OD at 700/175 MHz

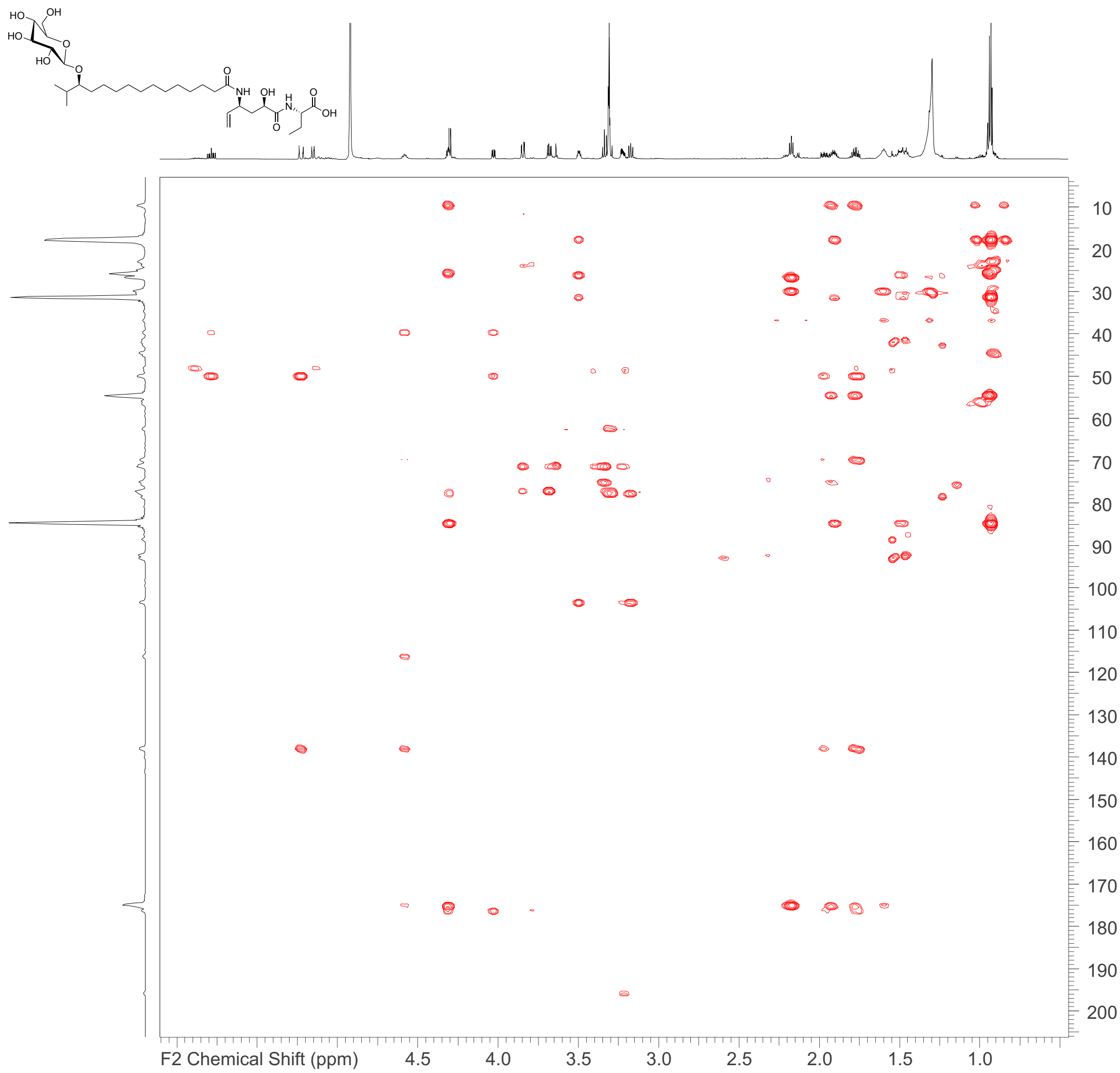

Figure S16. HMBC spectrum of myxoglucamide B acquired in CD_3OD at 700/175 MHz

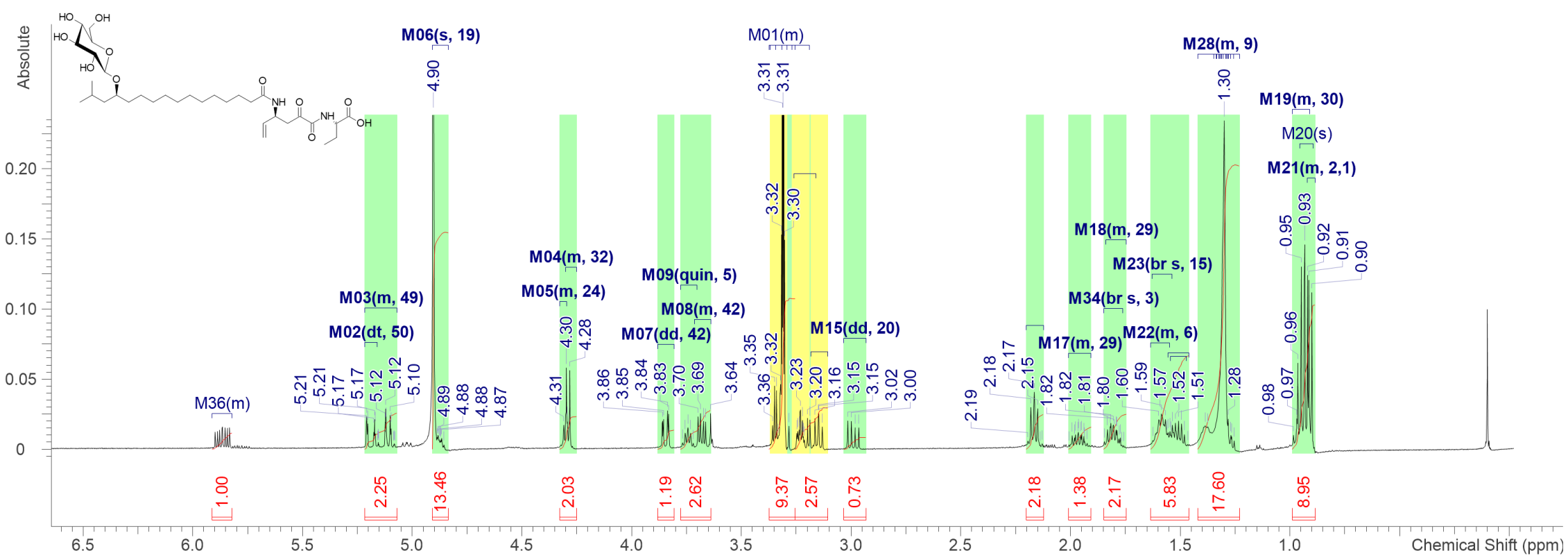

Figure S17. ^{1}H spectrum of myxoglucamide C acquired in CD_3OD at 500 MHz

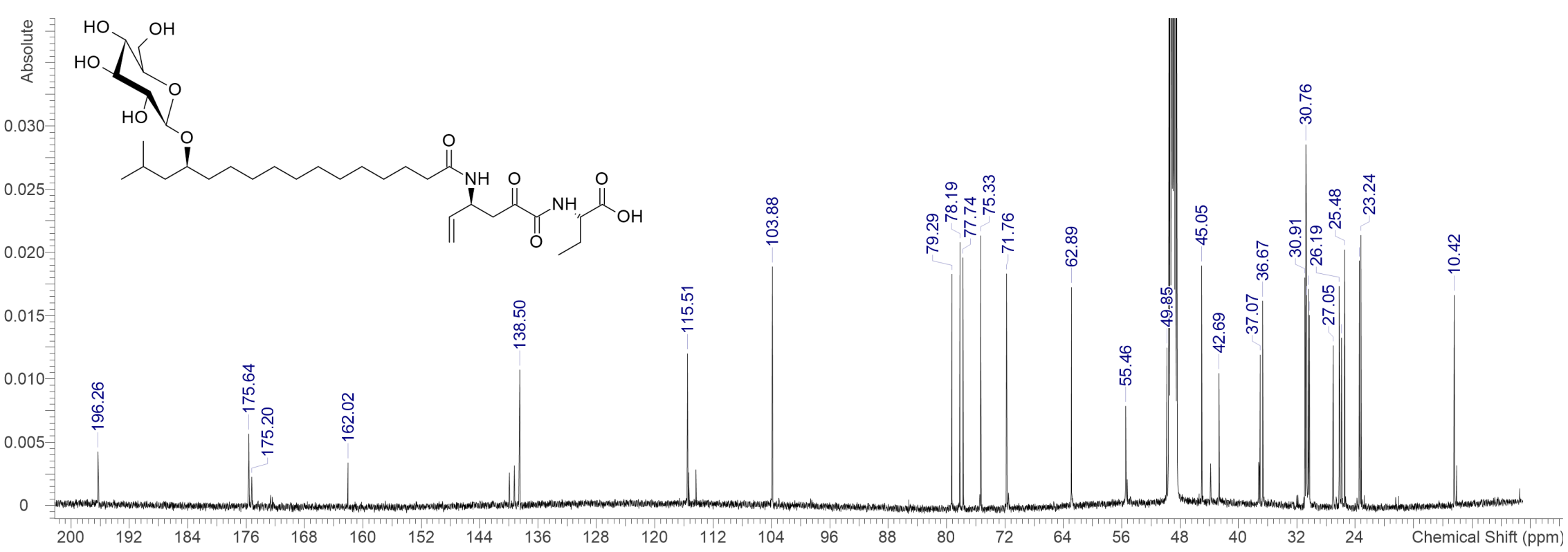

Figure S18. ^{13}C spectrum of myxoglucamide C acquired in CD_3OD at 125 MHz

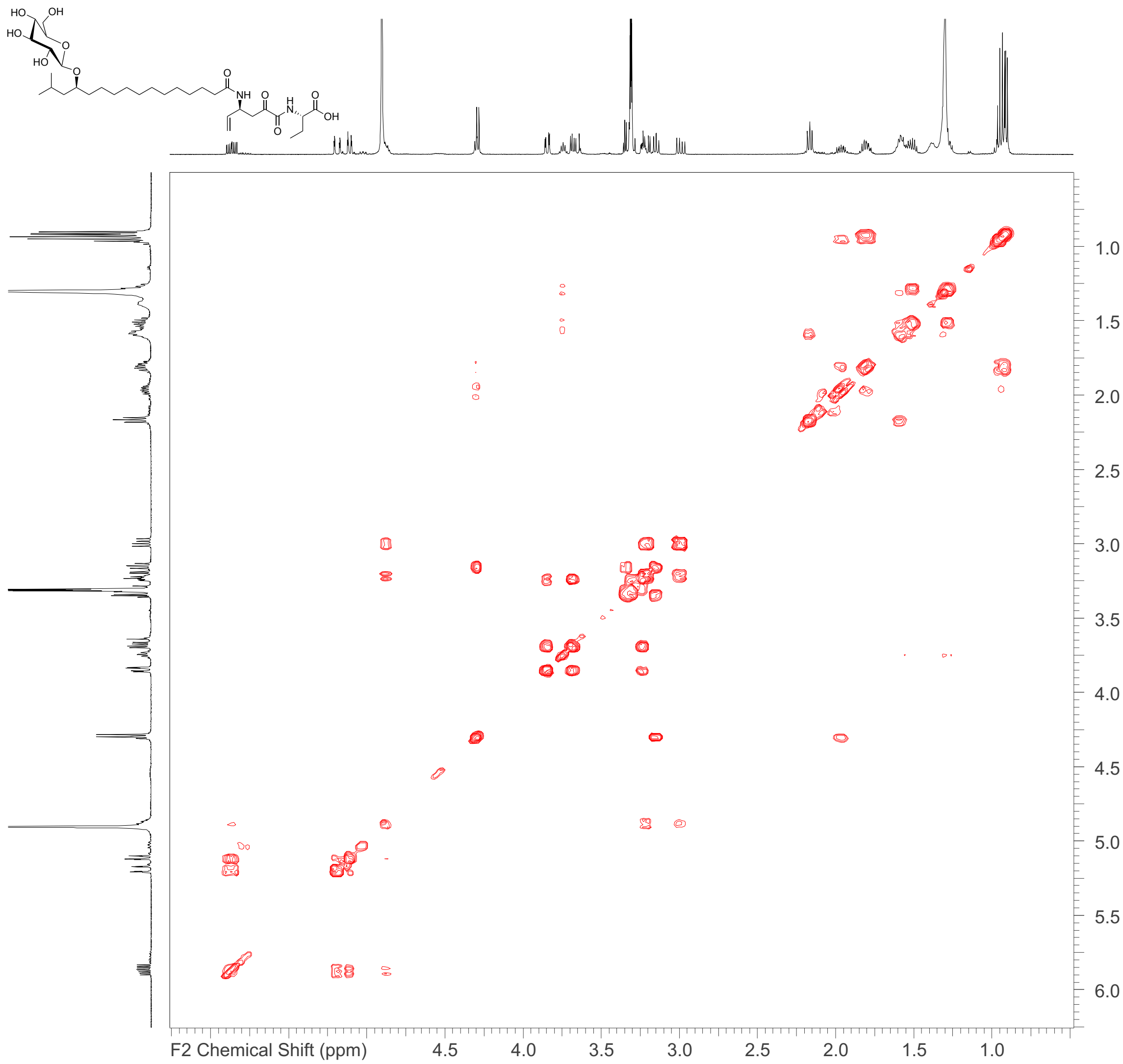

Figure S19. H,H COSY spectrum of myxoglucamide C acquired in CD_3OD at 500 MHz

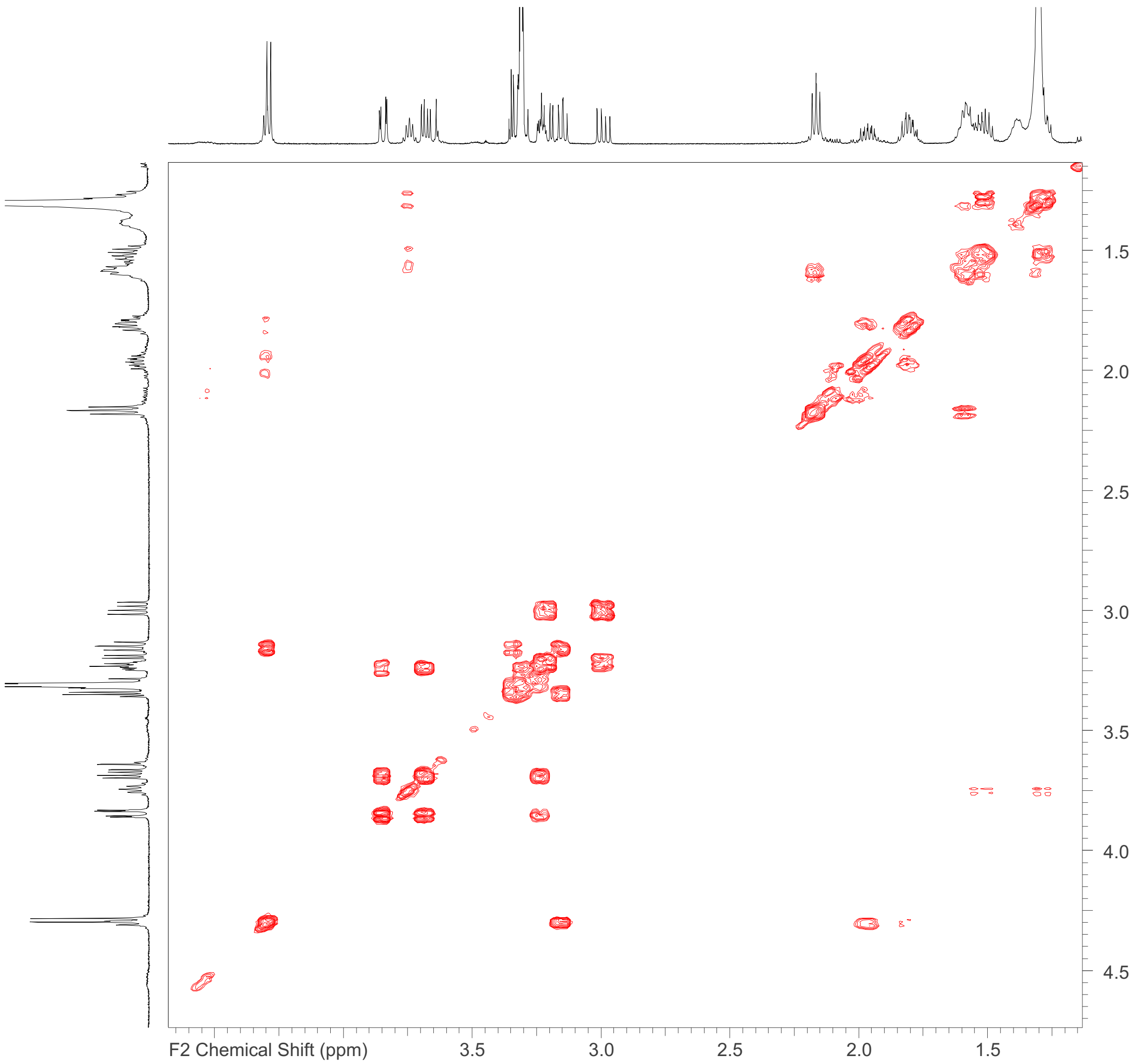

Figure S20. Enlarged H,H COSY spectrum of myxoglucamide C acquired in CD_3OD at 500 MHz

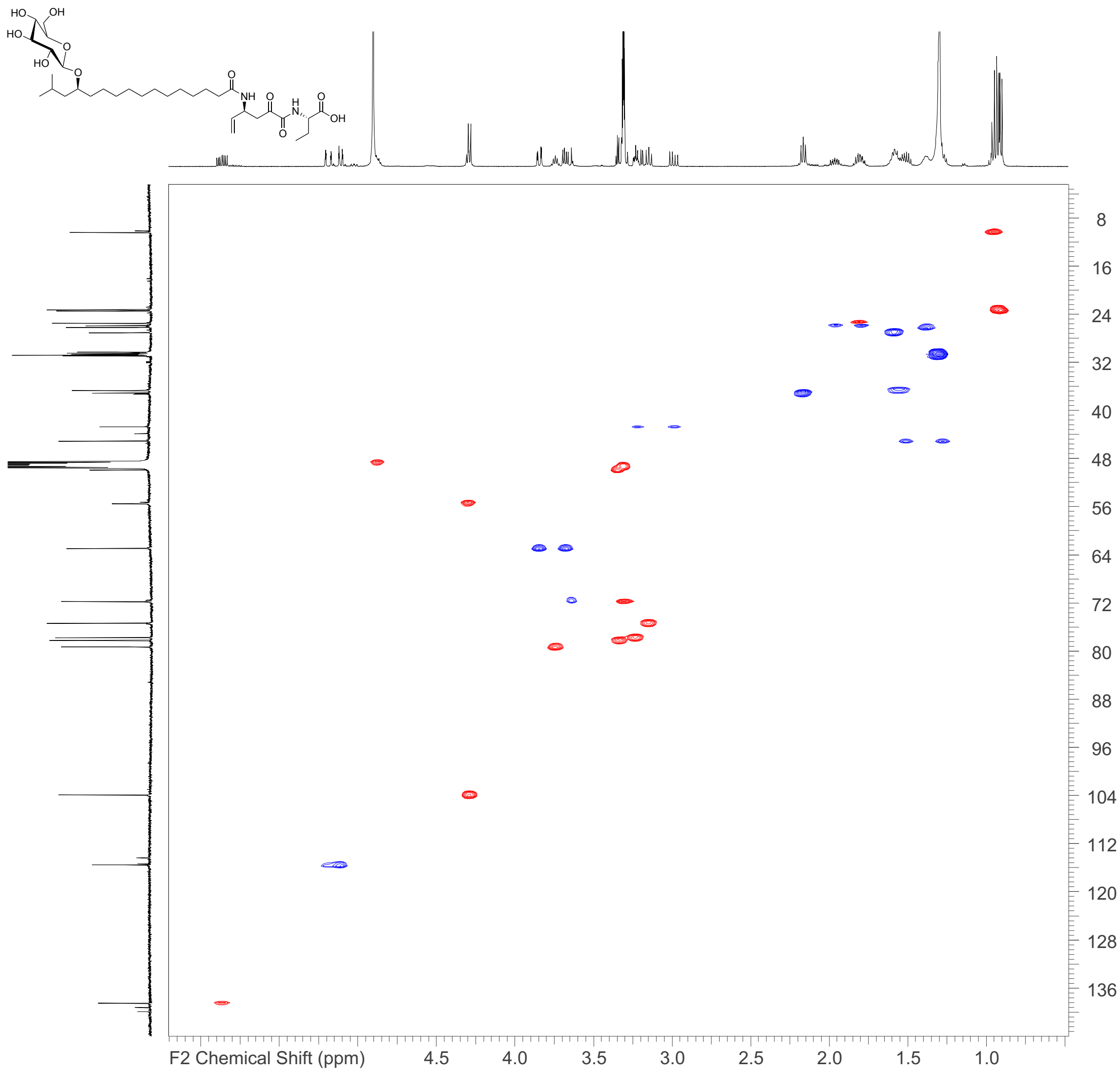

Figure S21. HSQC spectrum of myxoglucamide C acquired in CD_3OD at 500/125 MHz

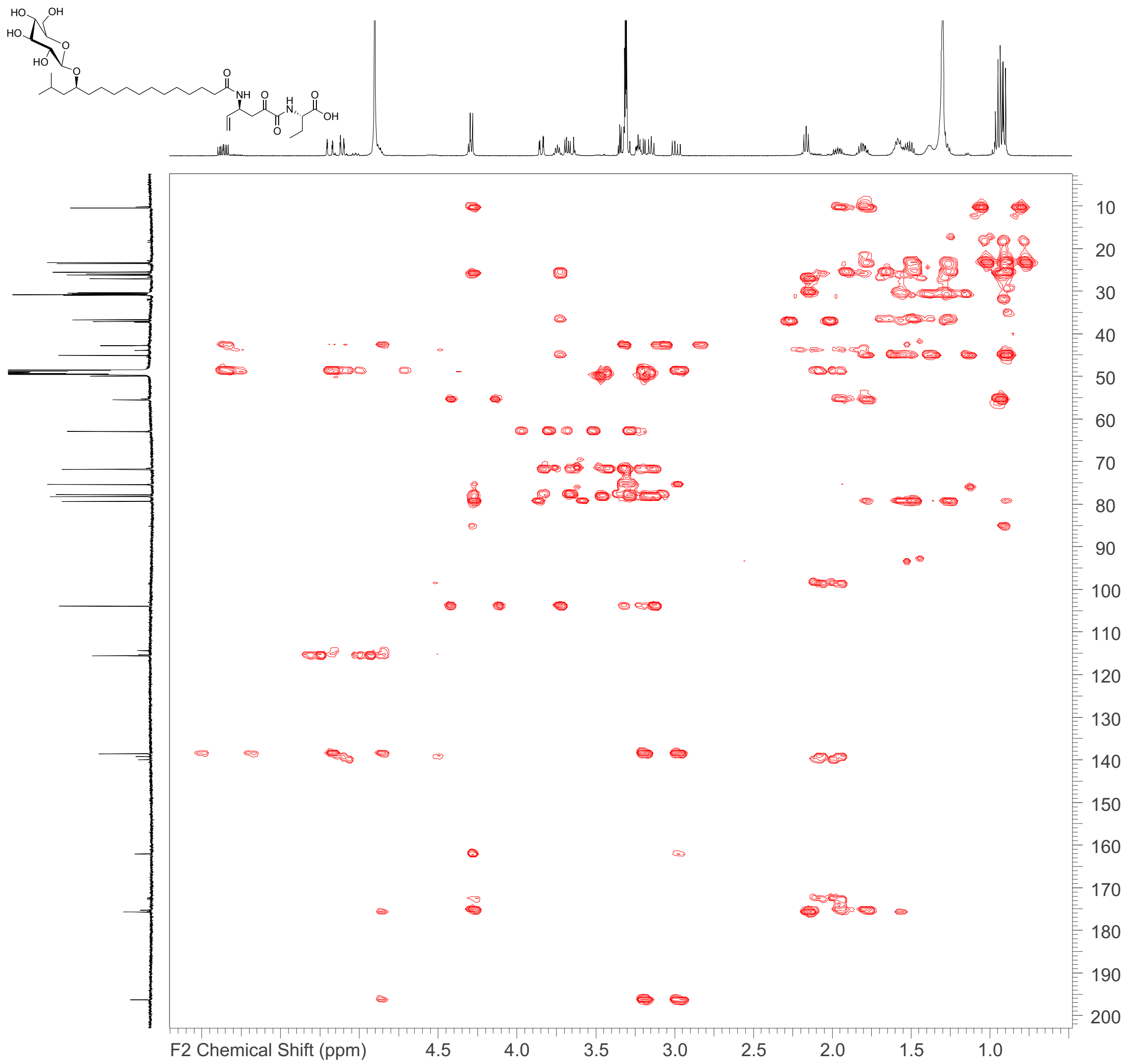

Figure S22. HMBC spectrum of myxoglucamide C acquired in CD_3OD at 500/125 MHz

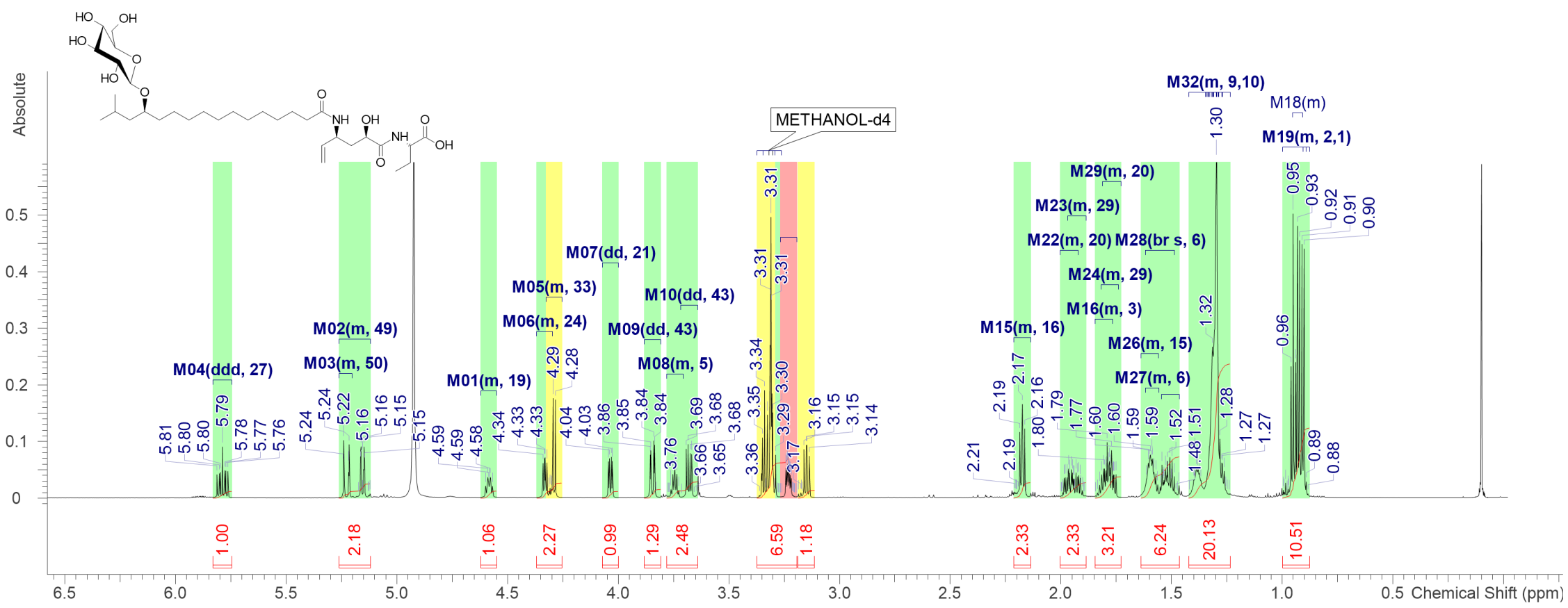

Figure S23. ^{1}H spectrum of myxoglucamide D acquired in CD_3OD at 700 MHz

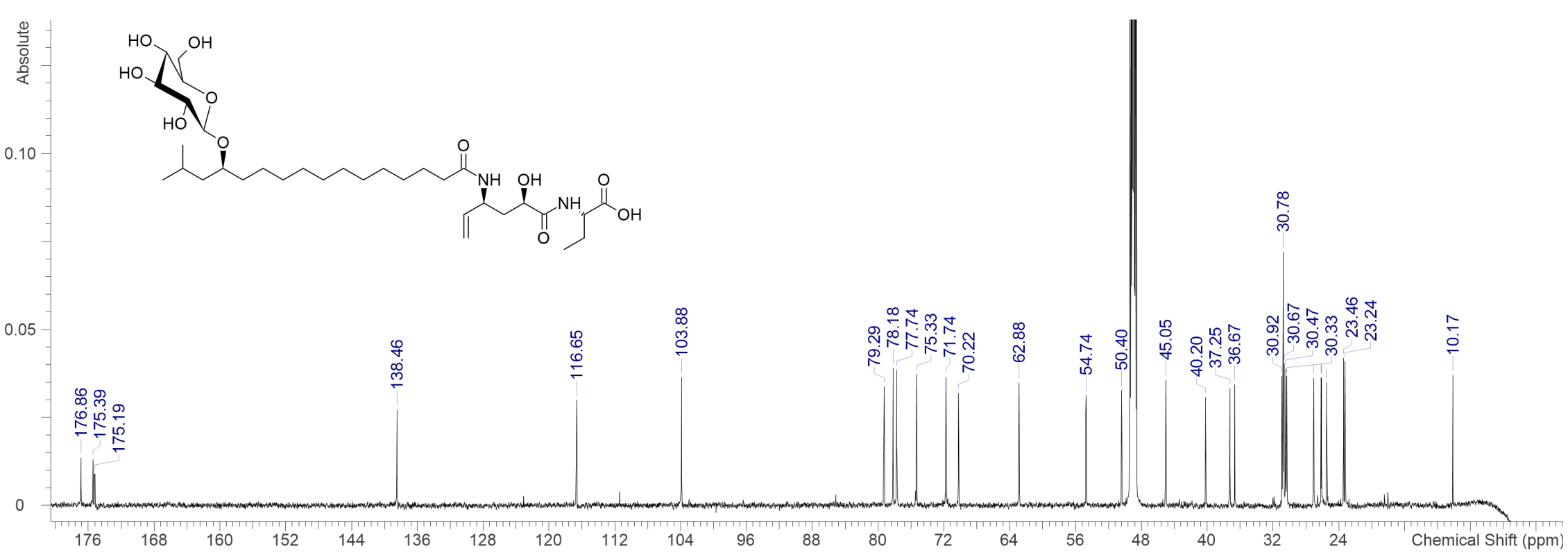

Figure S24. ^{13}C spectrum of myxoglucamide D acquired in CD_3OD at 175 MHz

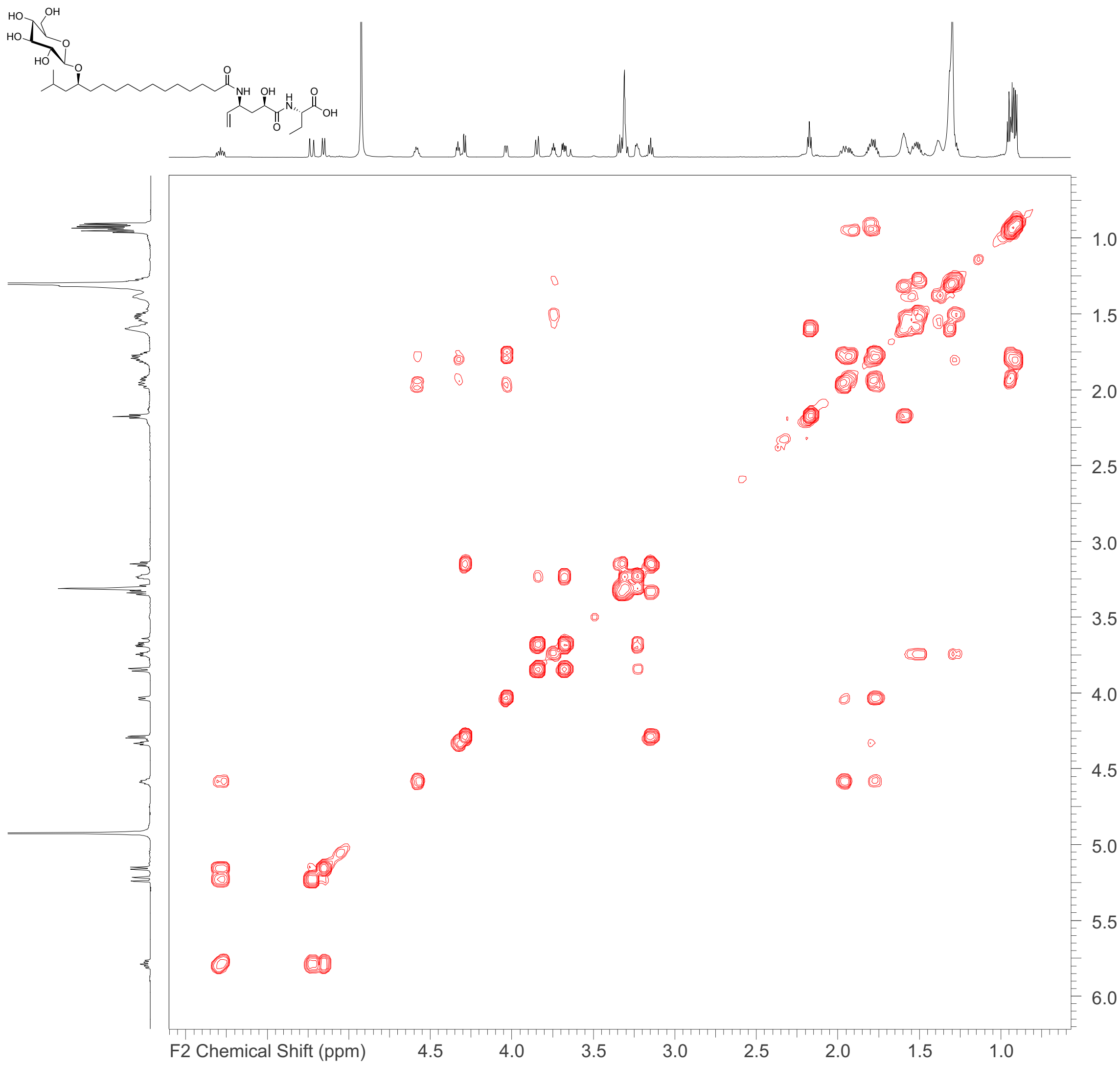

Figure S25. H,H COSY spectrum of myxoglucamide D acquired in CD_3OD at 700 MHz

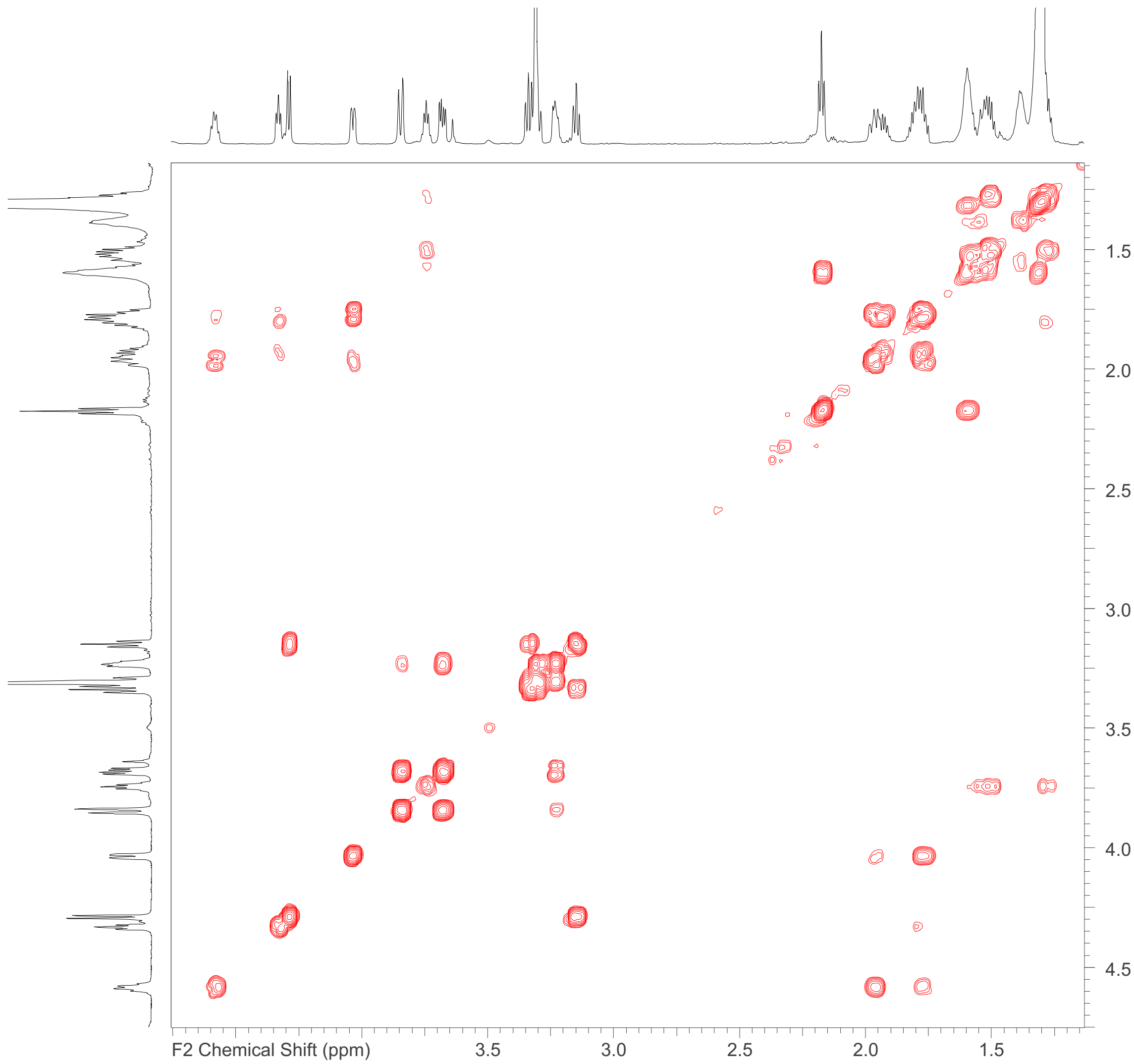

Figure S26. Enlarged H,H COSY spectrum of myxoglucamide D acquired in CD_3OD at 700 MHz

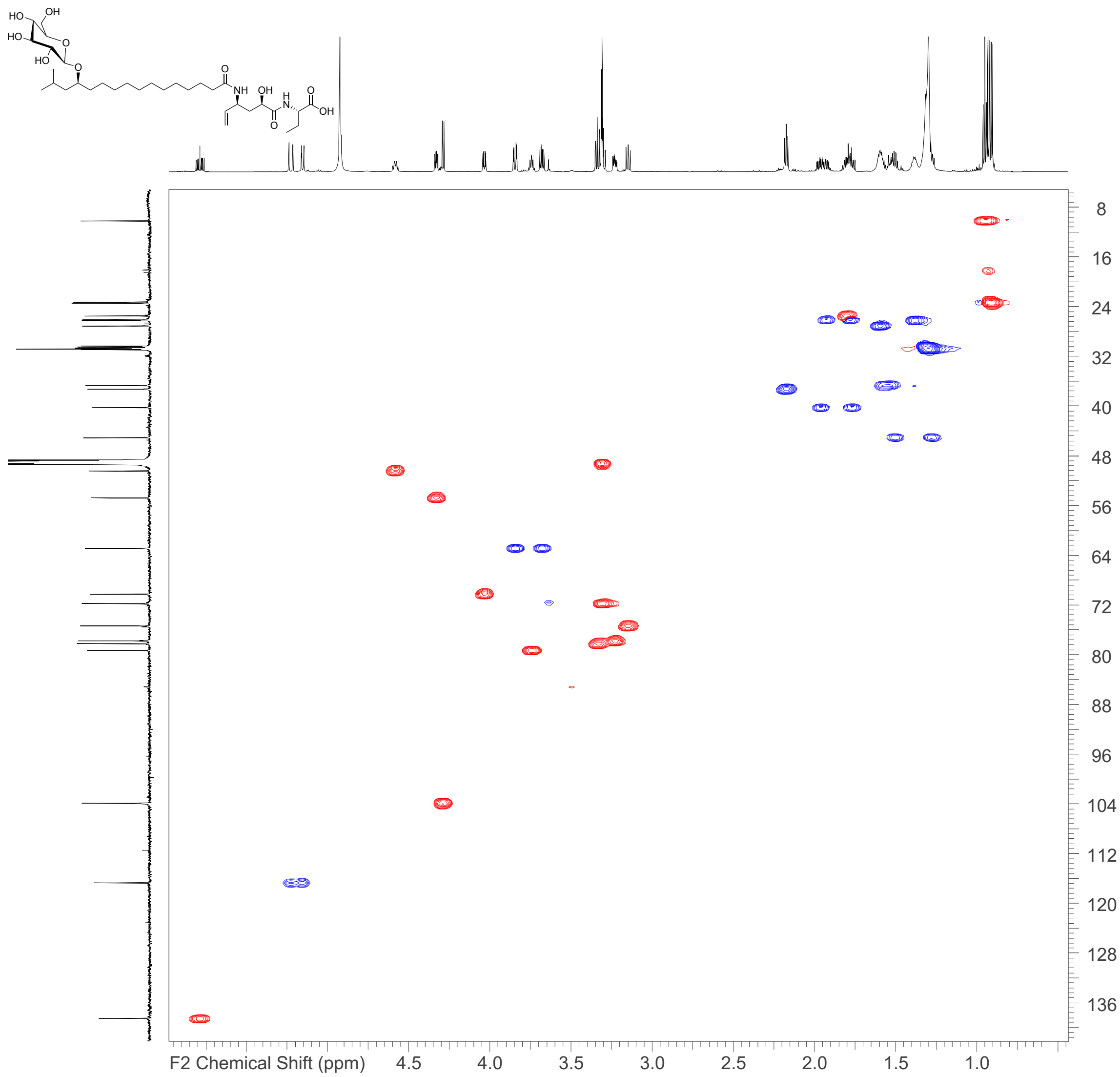

Figure S27. HSQC spectrum of myxoglucamide D acquired in CD_3OD at 700/175 MHz

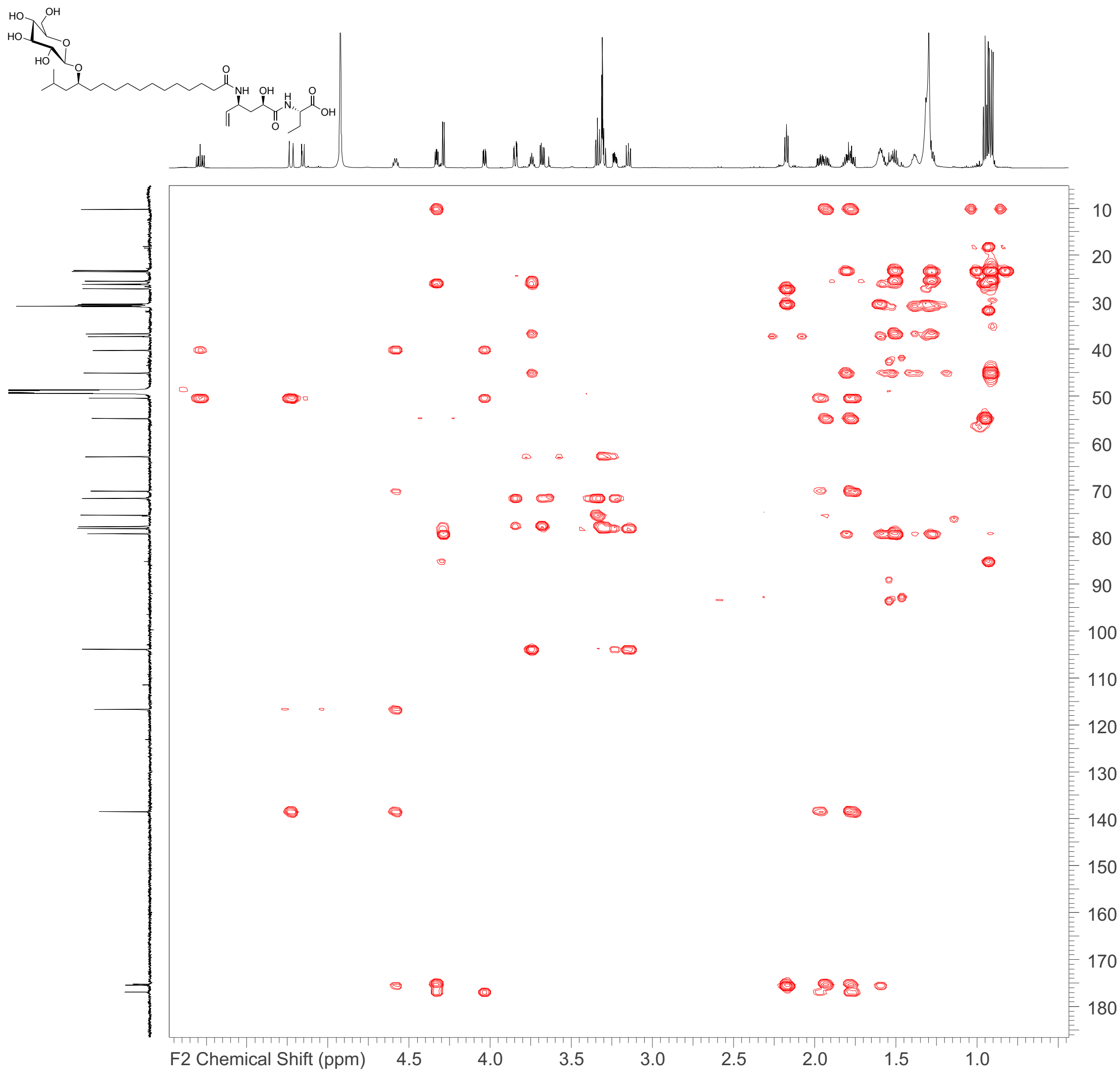

Figure S28. HMBC spectrum of myxoglucamide D acquired in CD_3OD at 700/175 MHz

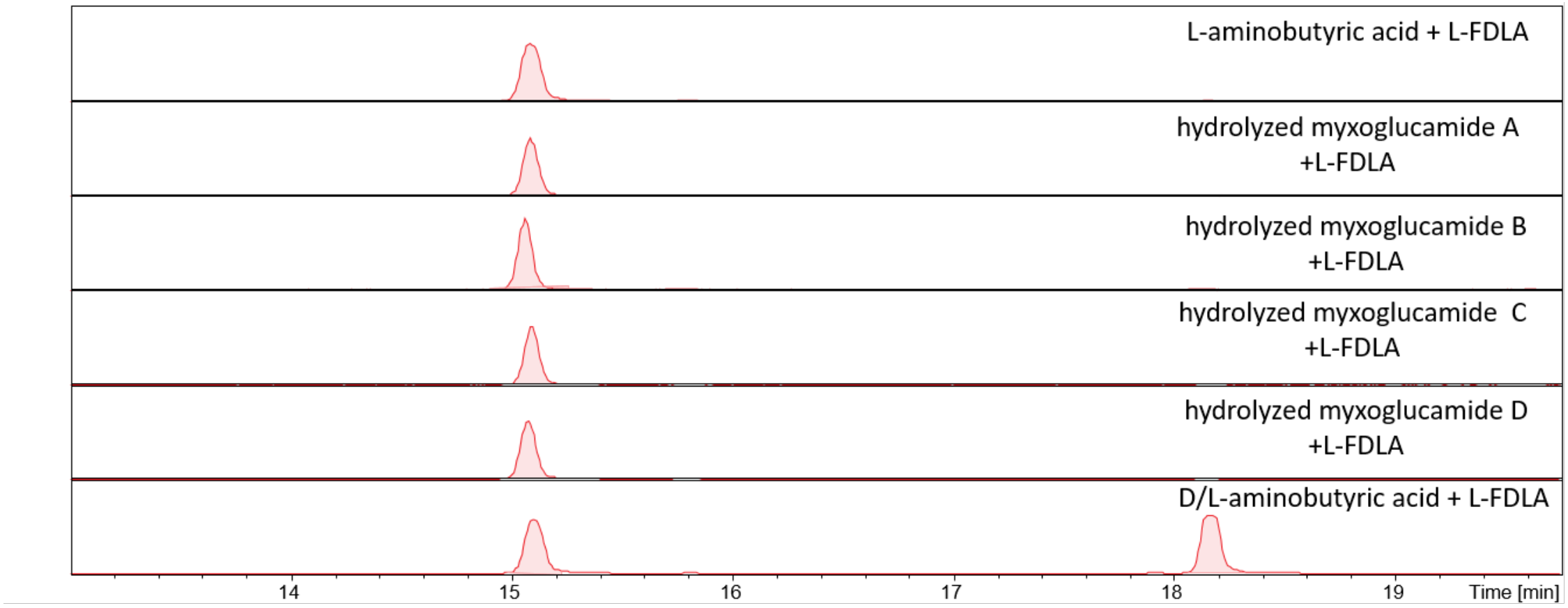

Figure S29. Marfey's analysis (L-FDLA) for amino-butyric acid standards and four isolated myxoglucamides after acid hydrolysis

Extracted Ion Chromatogram (EIC) 398.17 ± 0.1 *m/z* for L-FDLA derivatized amino butyric acid.

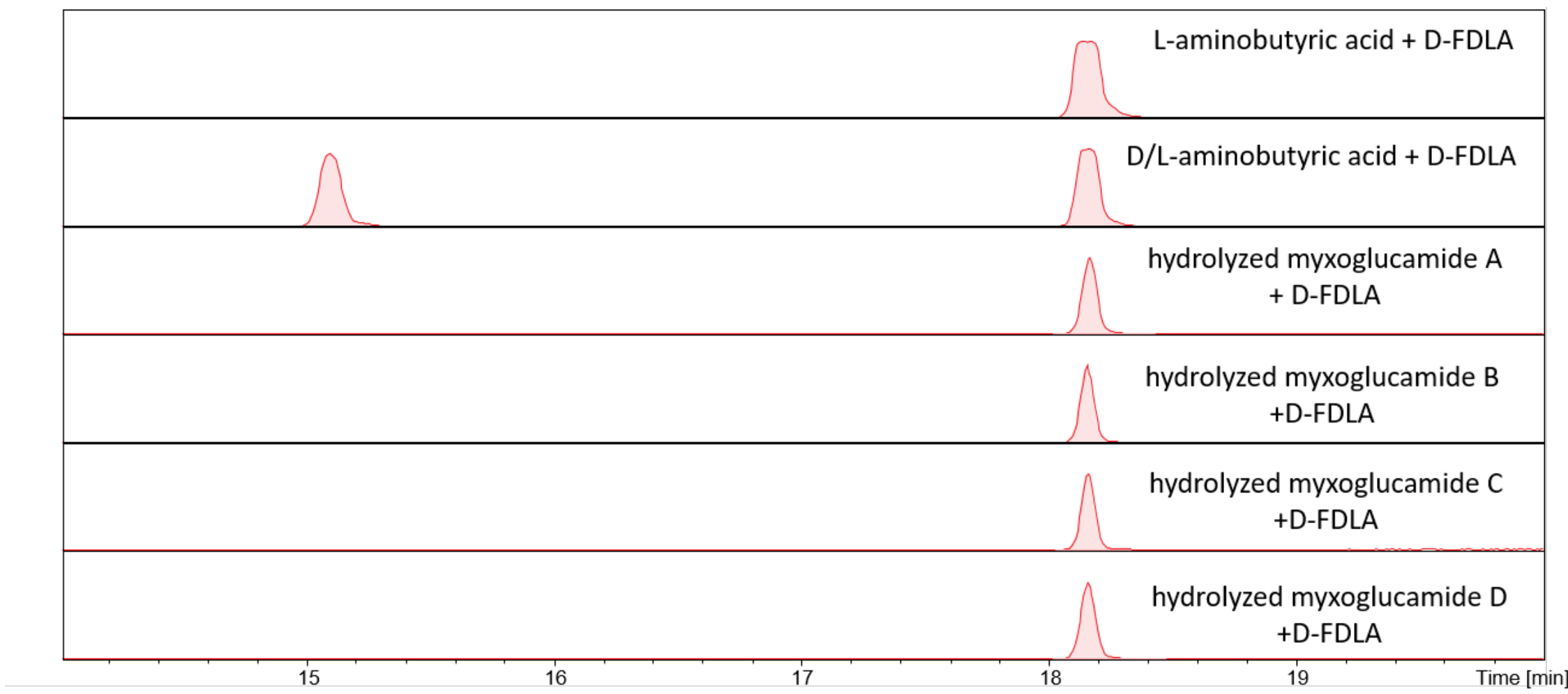

Figure S30. Marfey's analysis (D-FDLA) for amino-butyric acid standards and four isolated myxoglucamides after acid hydrolysis

Extracted Ion Chromatogram (EIC) 398.17 ± 0.1 *m/z* for D-FDLA derivatized amino butyric acid.

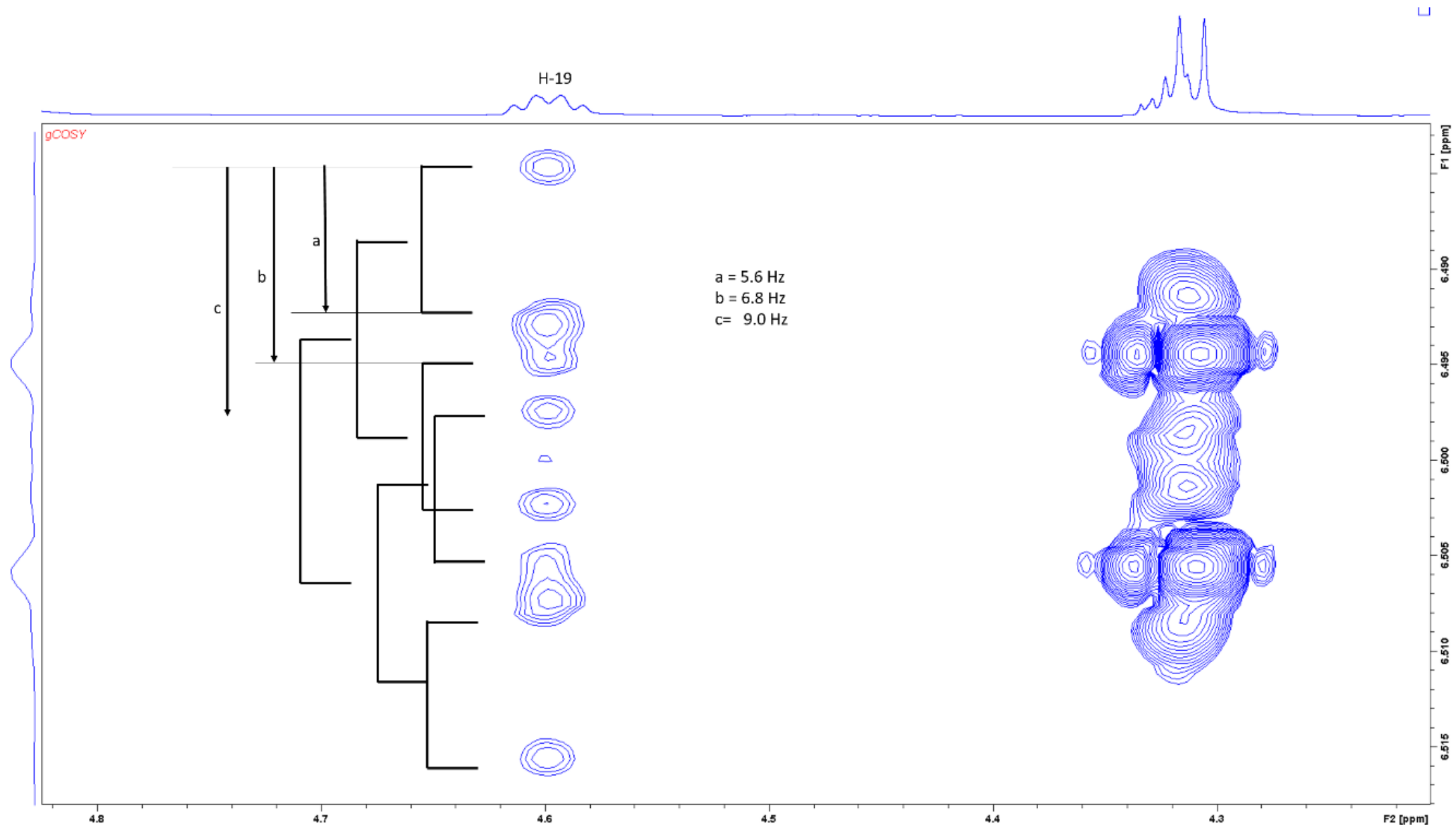

Figure S31. *J* resolved COSY spectrum of myxoglucamide D showing coupling pattern of H-19

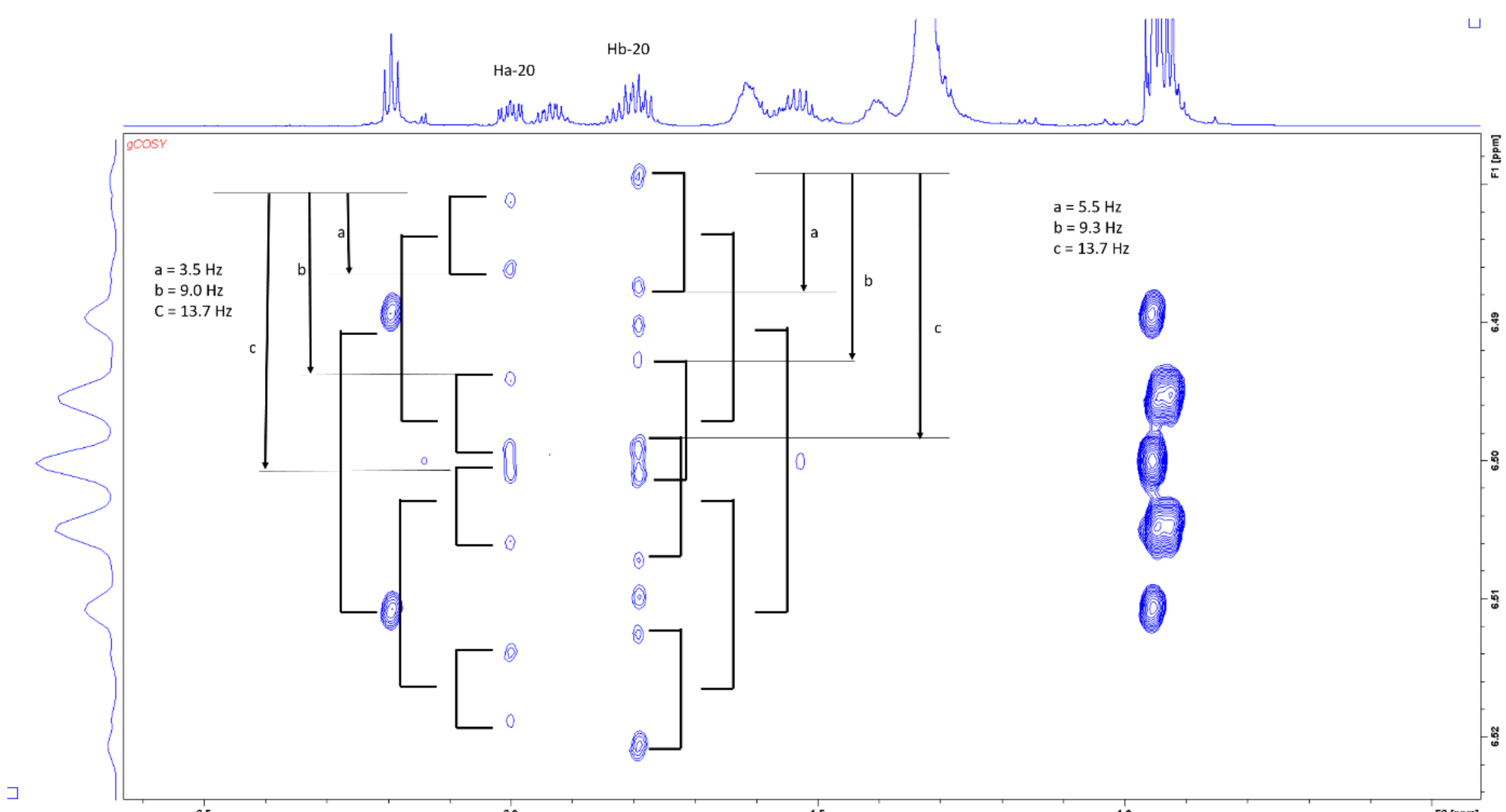

Figure S32. *J* resolved COSY spectrum of myxoglucamide D showing coupling pattern of Ha-20 and Hb-20

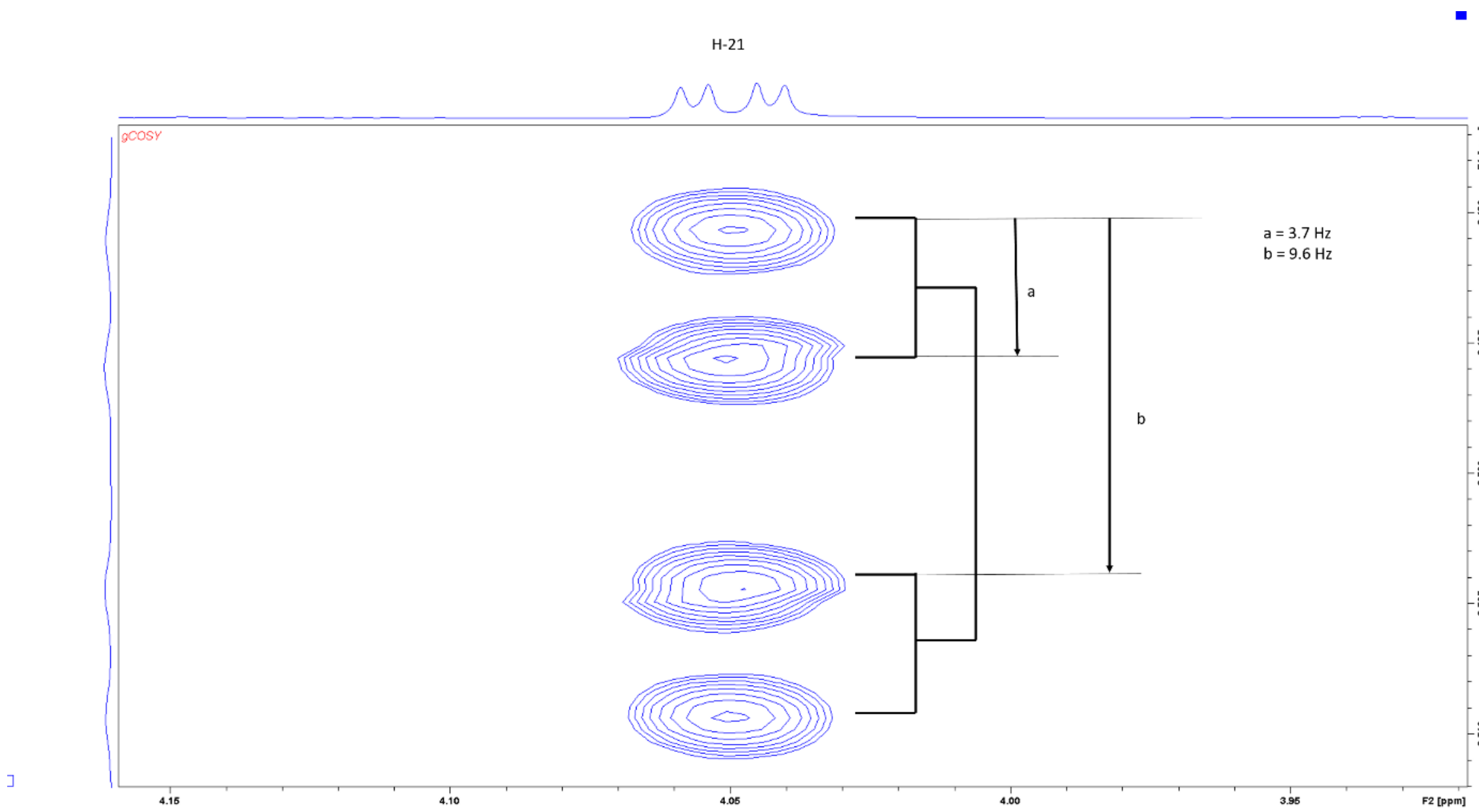

Figure S33. *J* resolved COSY spectrum of myxoglucamide D showing coupling pattern of H-21

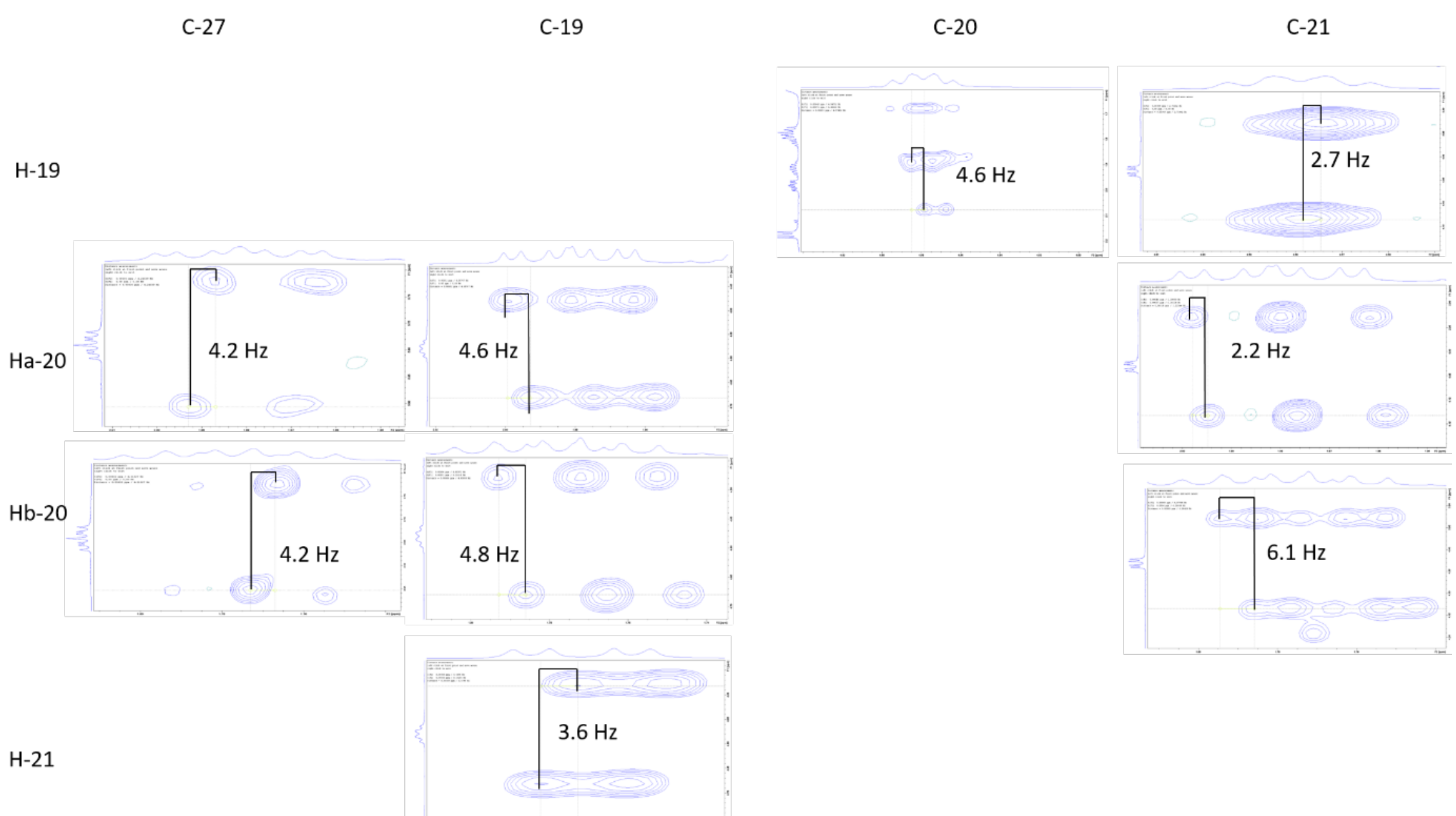

Figure S34. HC coupling constants for the *J* based analysis, extracted from a dipsi2 NMR experiment

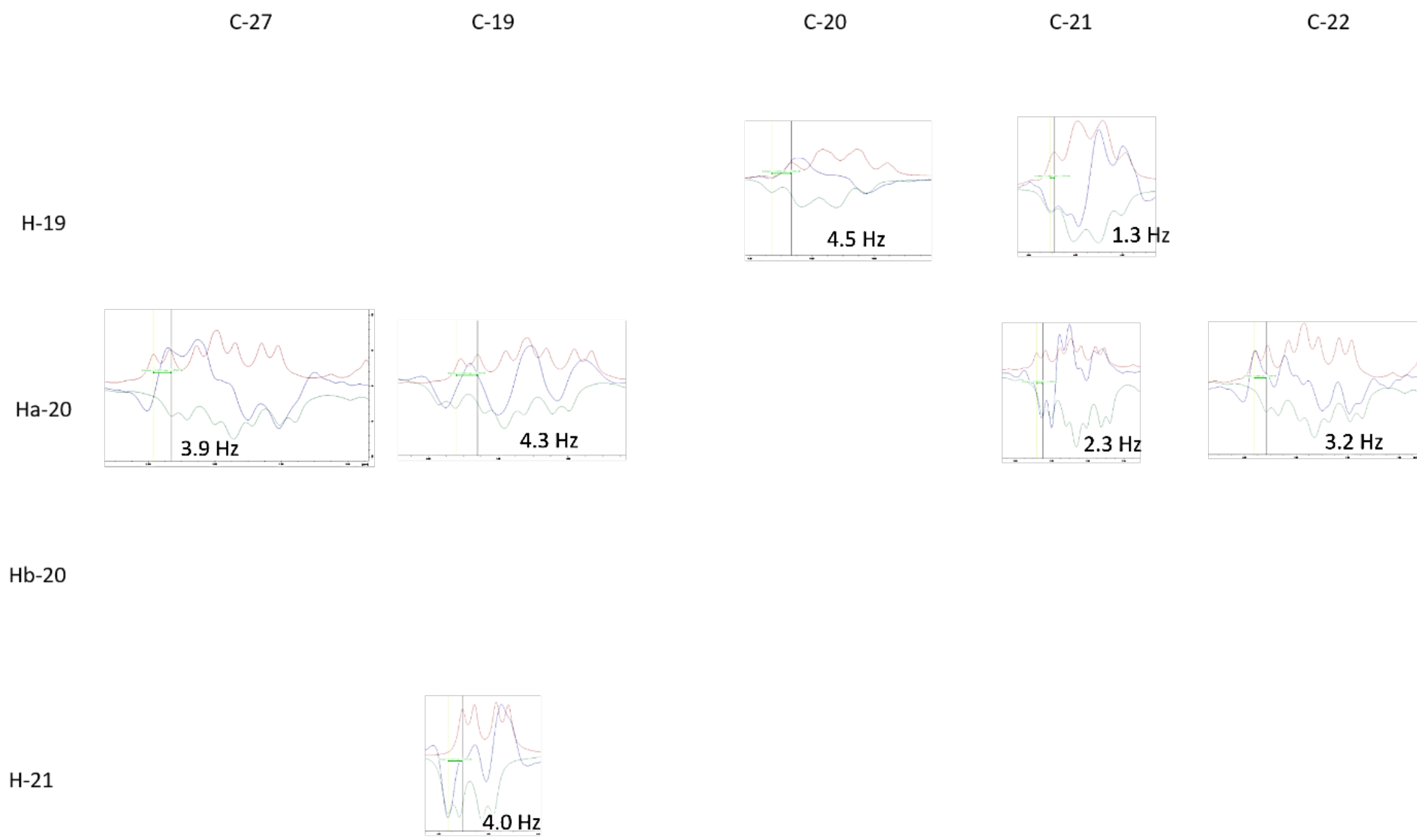

Figure S35. HC coupling constants for the *J* based analysis, extracted from a HSQMBC NMR experiment

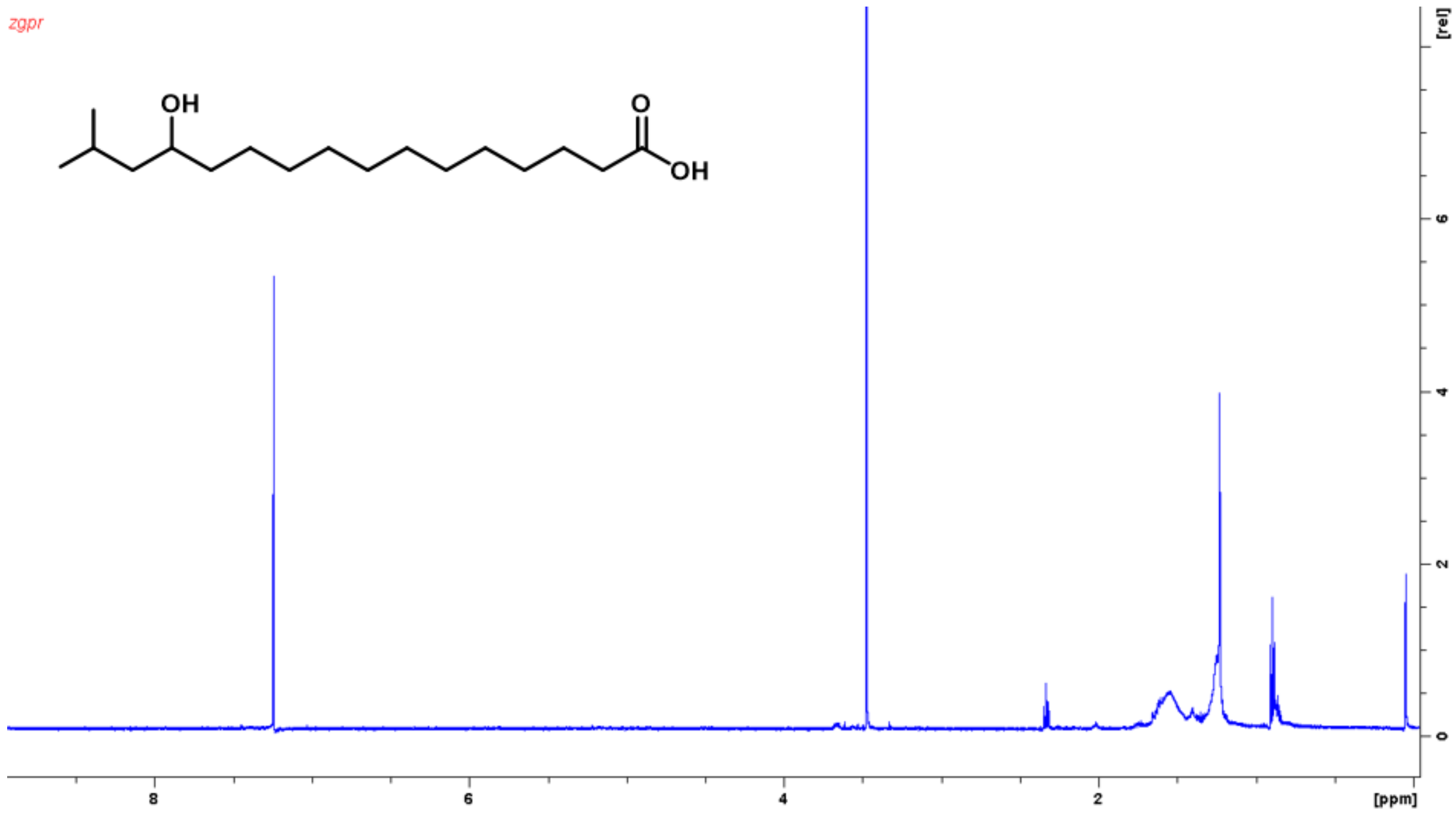

Figure S36. ^{1}H spectrum of HMHA acquired in $CDCl_3$ at 500 MHz isolated after acid hydrolysis of myxoglucamide C

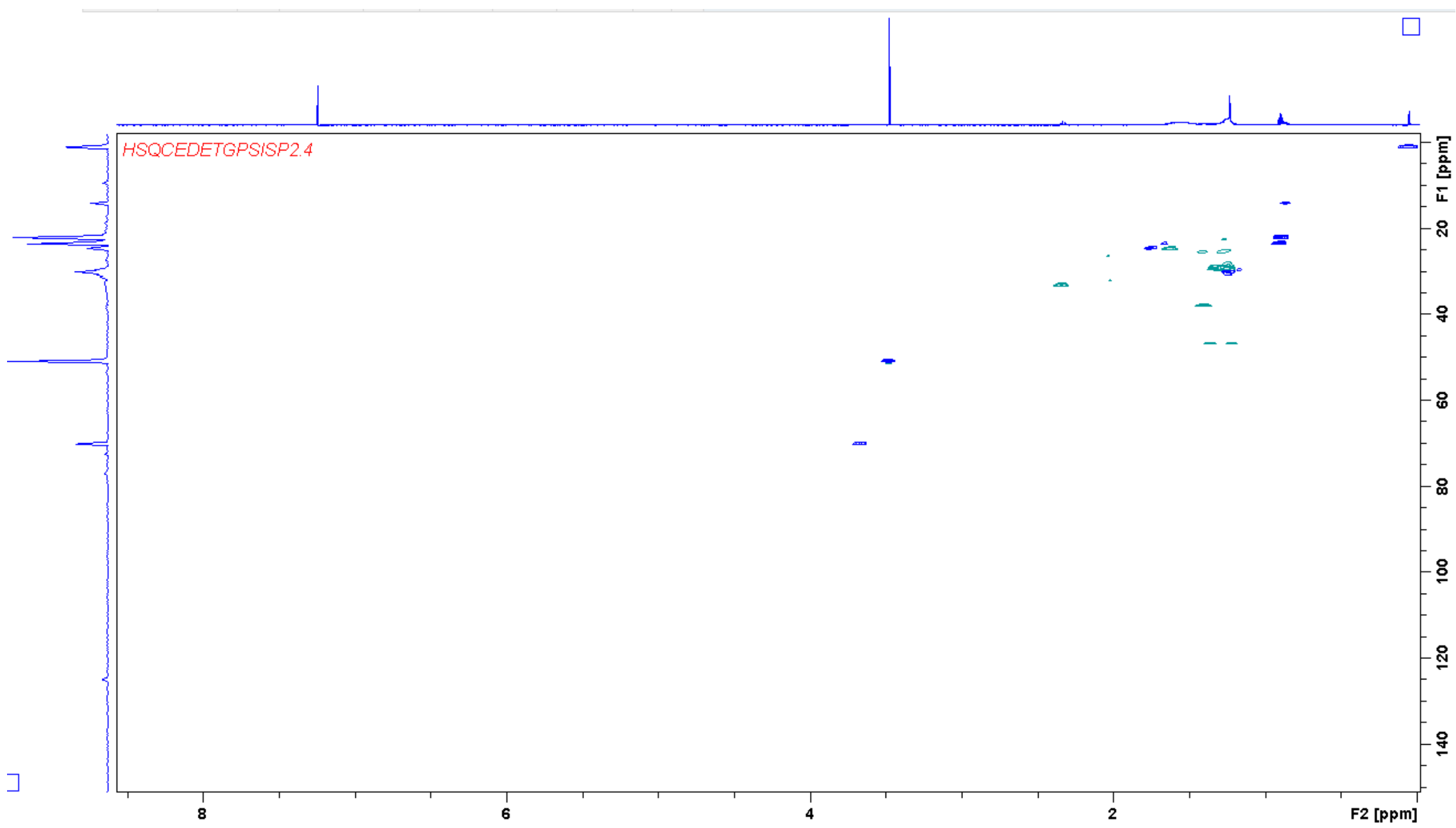

Figure S37. HSQC spectrum of HMHA acquired in $CDCl_3$ at 500 MHz isolated after acid hydrolysis of myxoglucamide C

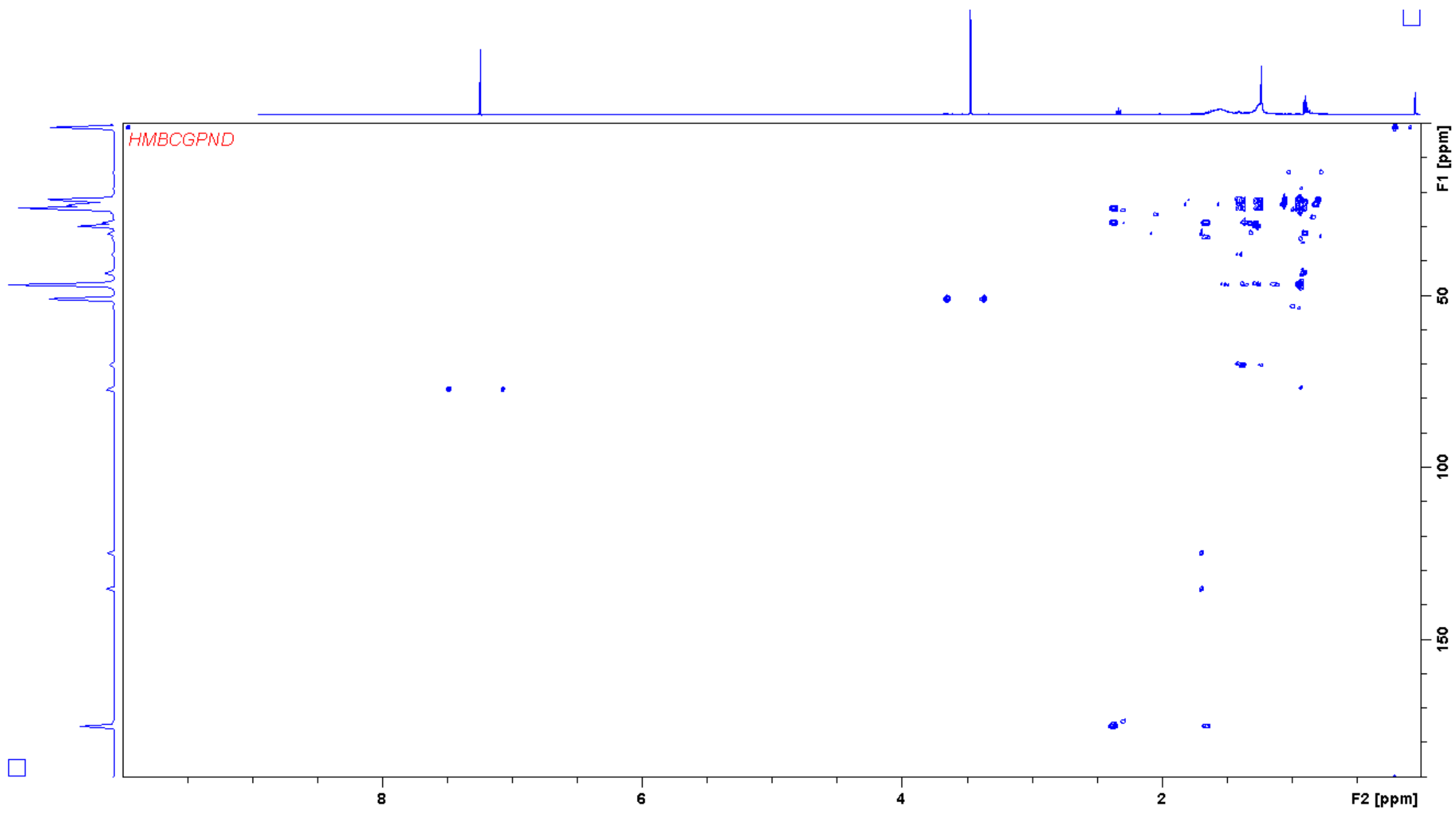

Figure S38. HMBC spectrum of HMHA acquired in $CDCl_3$ at 500 MHz isolated after acid hydrolysis of myxoglucamide C

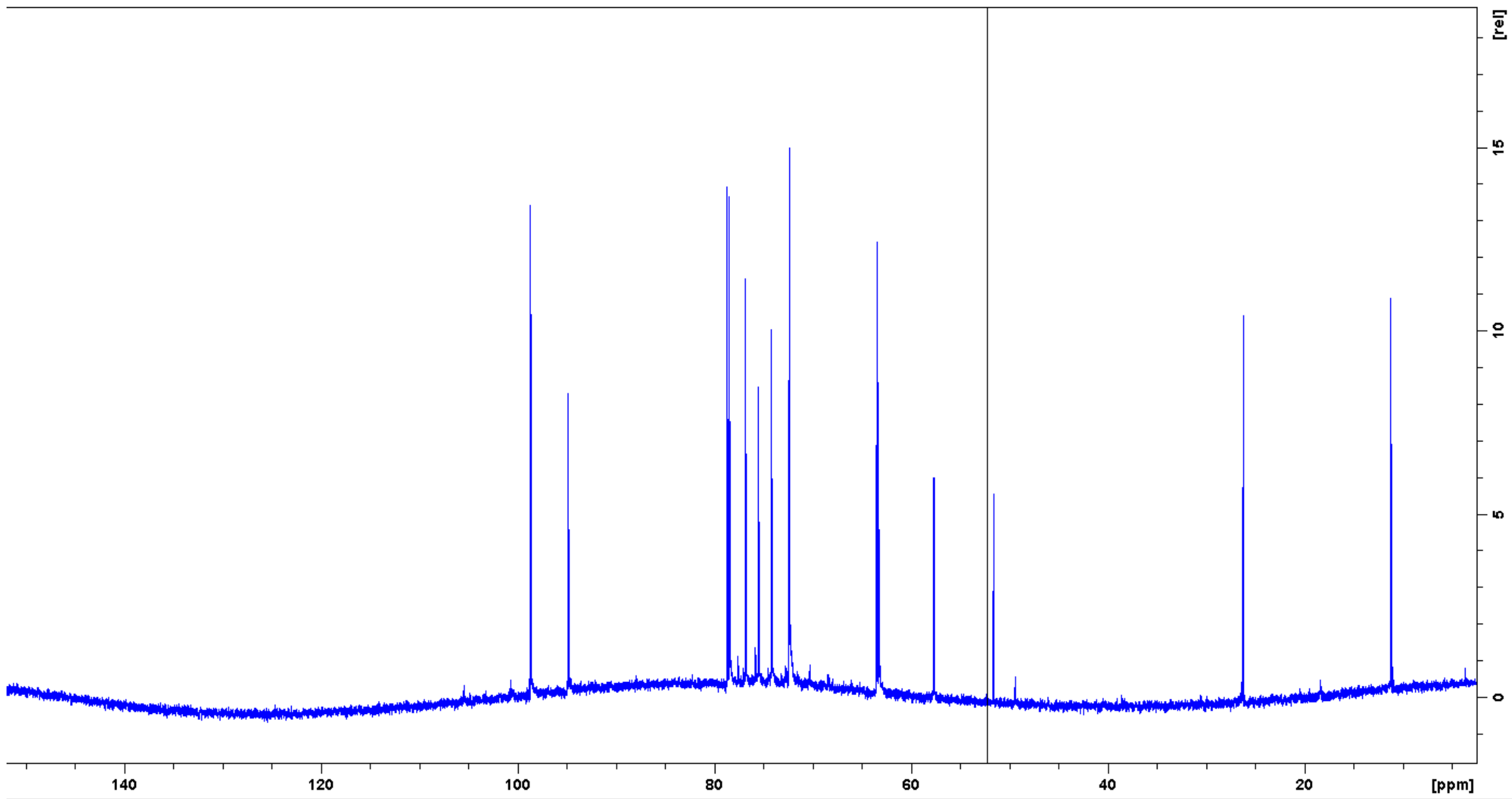

Figure S39. ^{1}H spectrum of aqueous extract of acid hydrolysis of myxoglucamide C acquired in D_2O at 500 MHz

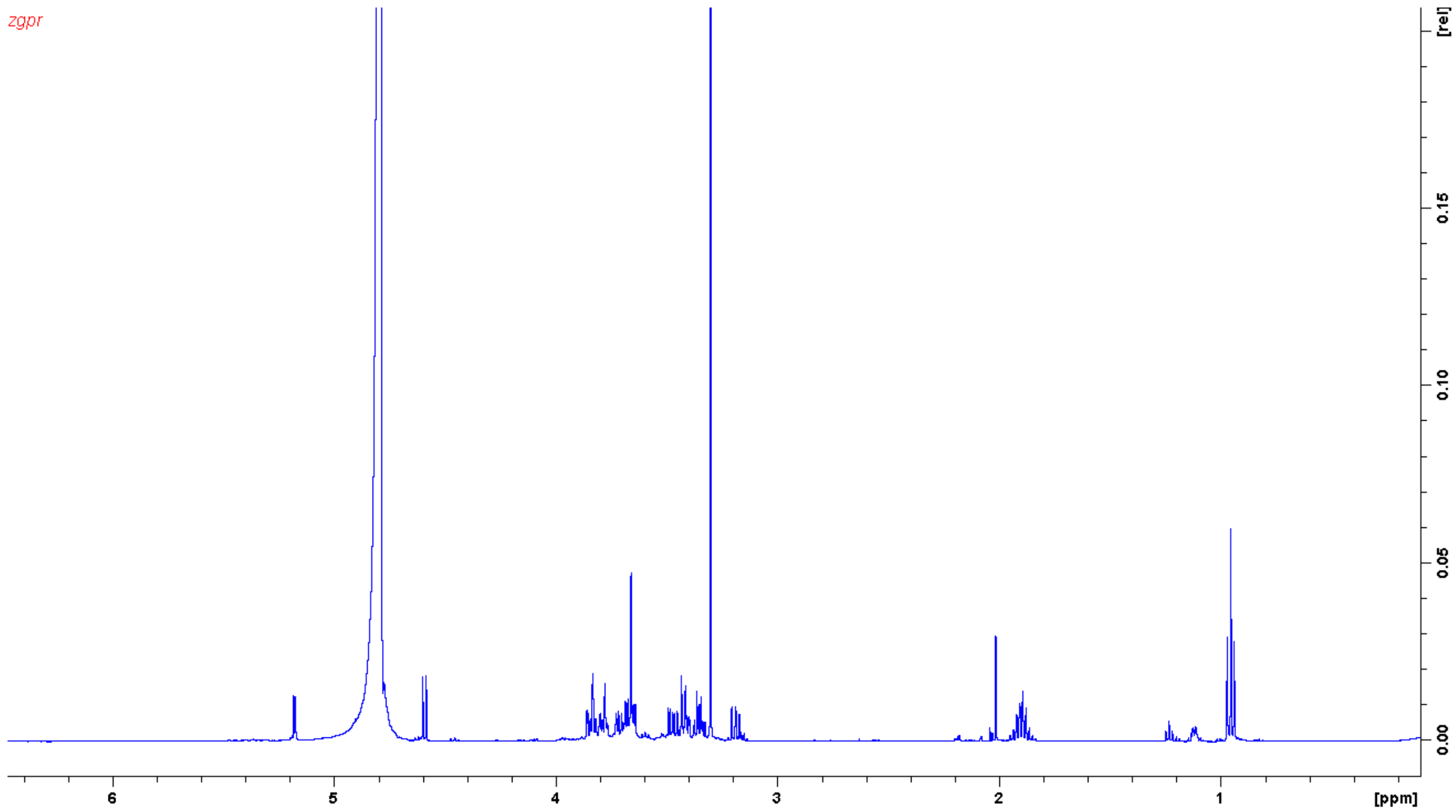

Figure S40. ^{13}C spectrum of aqueous extract of acid hydrolysis of myxoglucamide C acquired in D_2O at 125 MHz

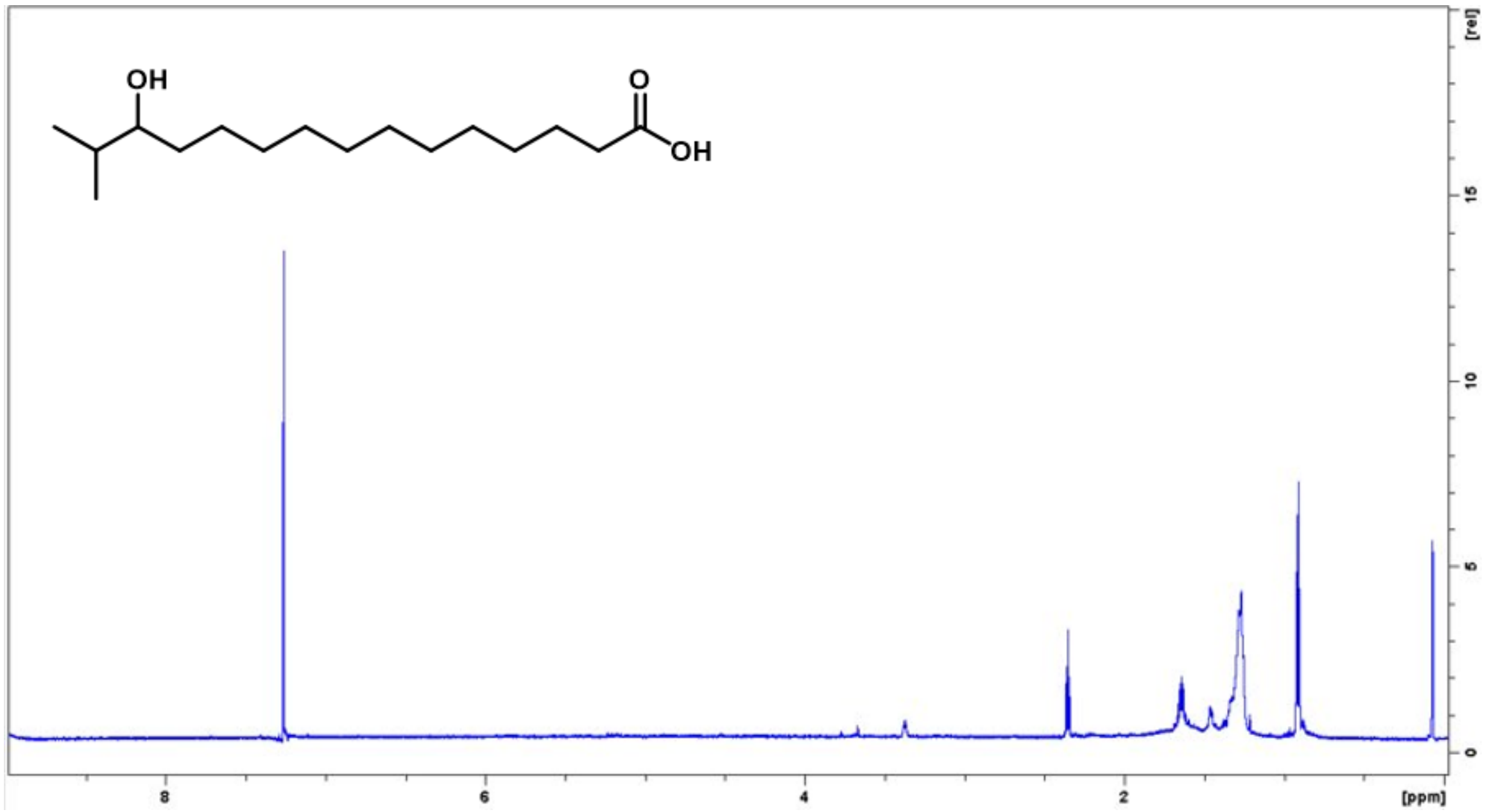

Figure S41. ^{1}H spectrum of HMPA acquired in $CDCl_3$ at 700 MHz isolated after acid hydrolysis of myxoglucamide A

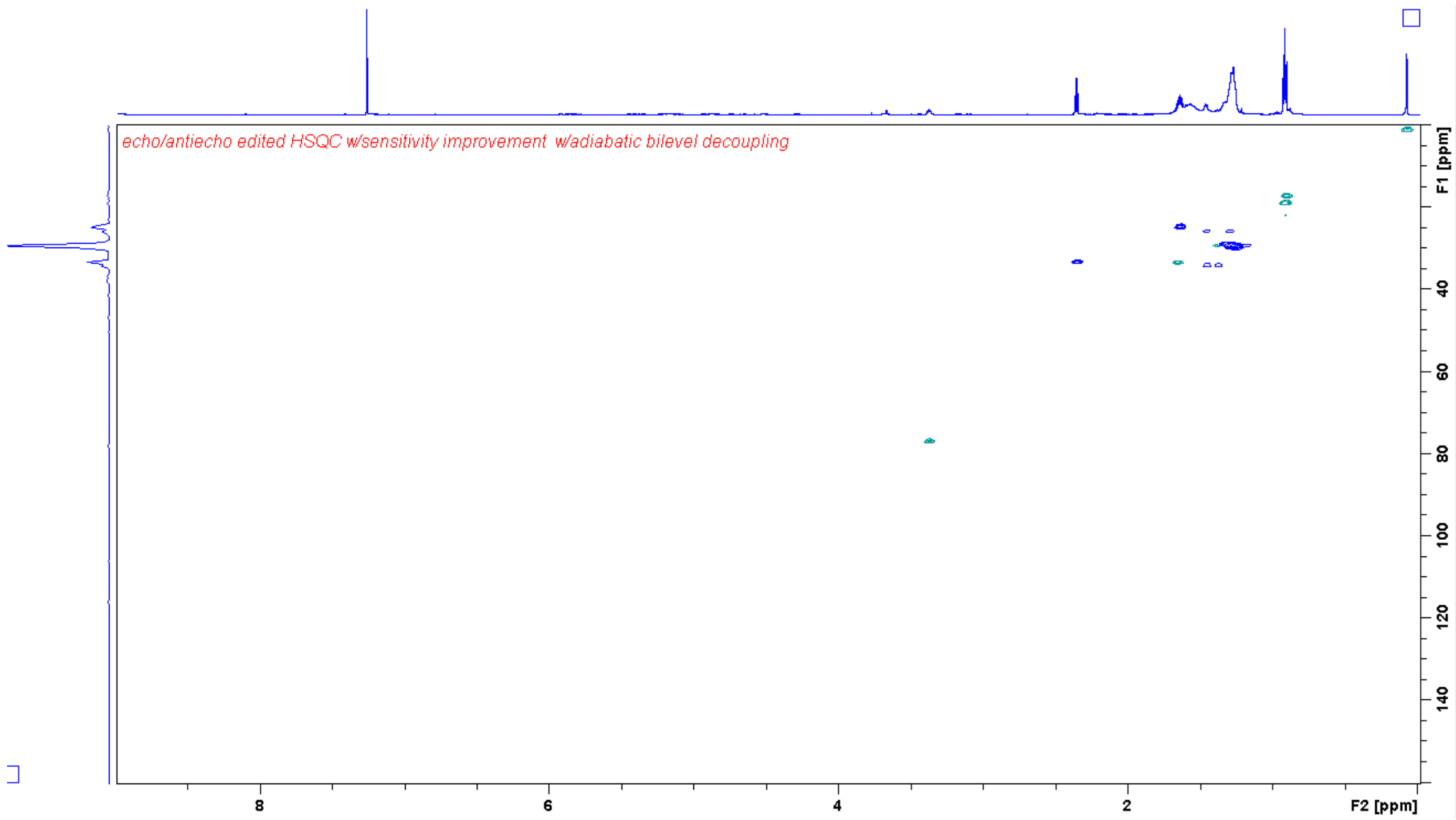

Figure S42. HSQC spectrum of HMPA acquired in $CDCl_3$ at 700 MHz isolated after acid hydrolysis of myxoglucamide A

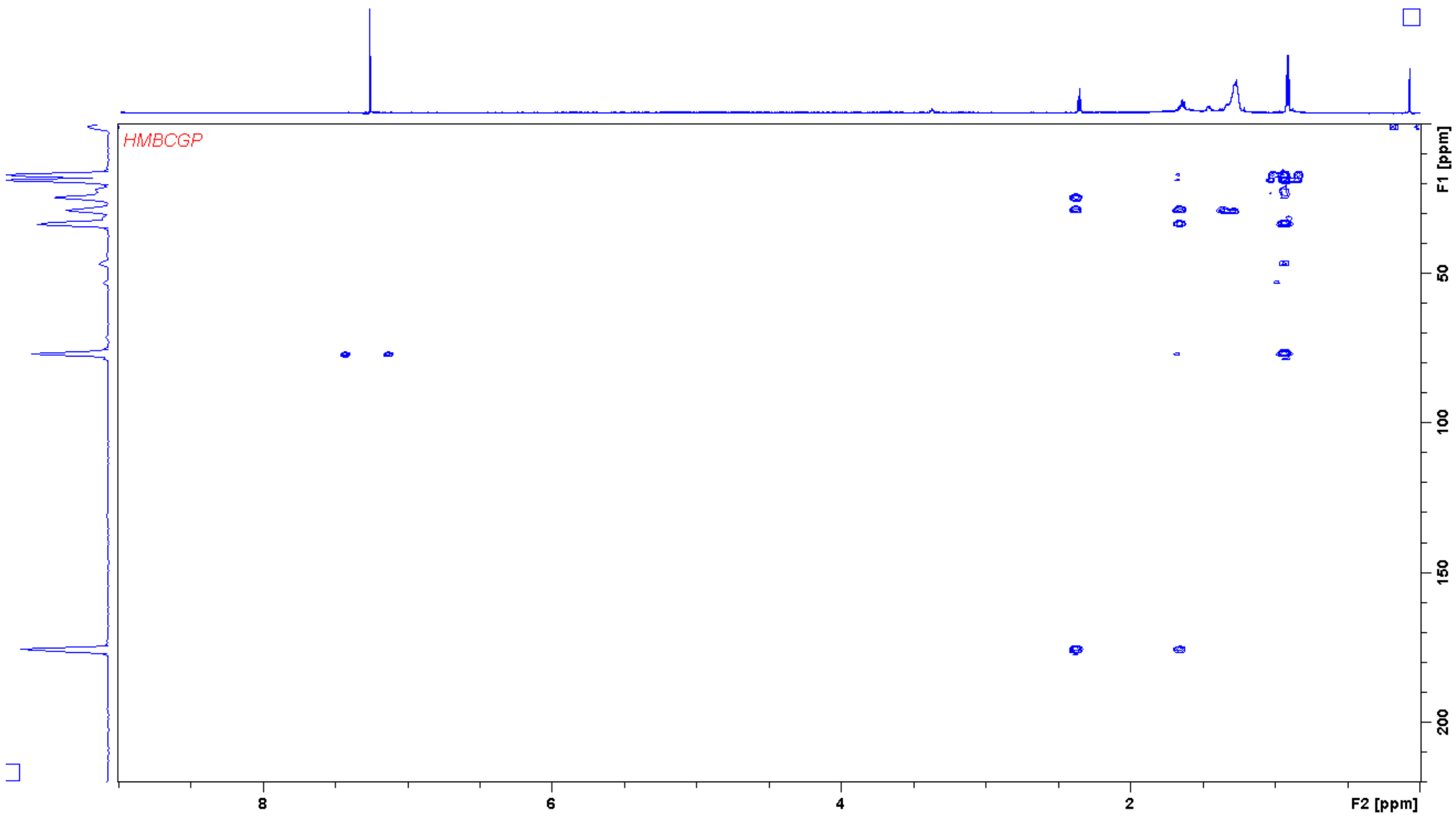

Figure S43. HMBC spectrum of HMPA acquired in $CDCl_3$ at 700 MHz isolated after acid hydrolysis of myxoglucamide A

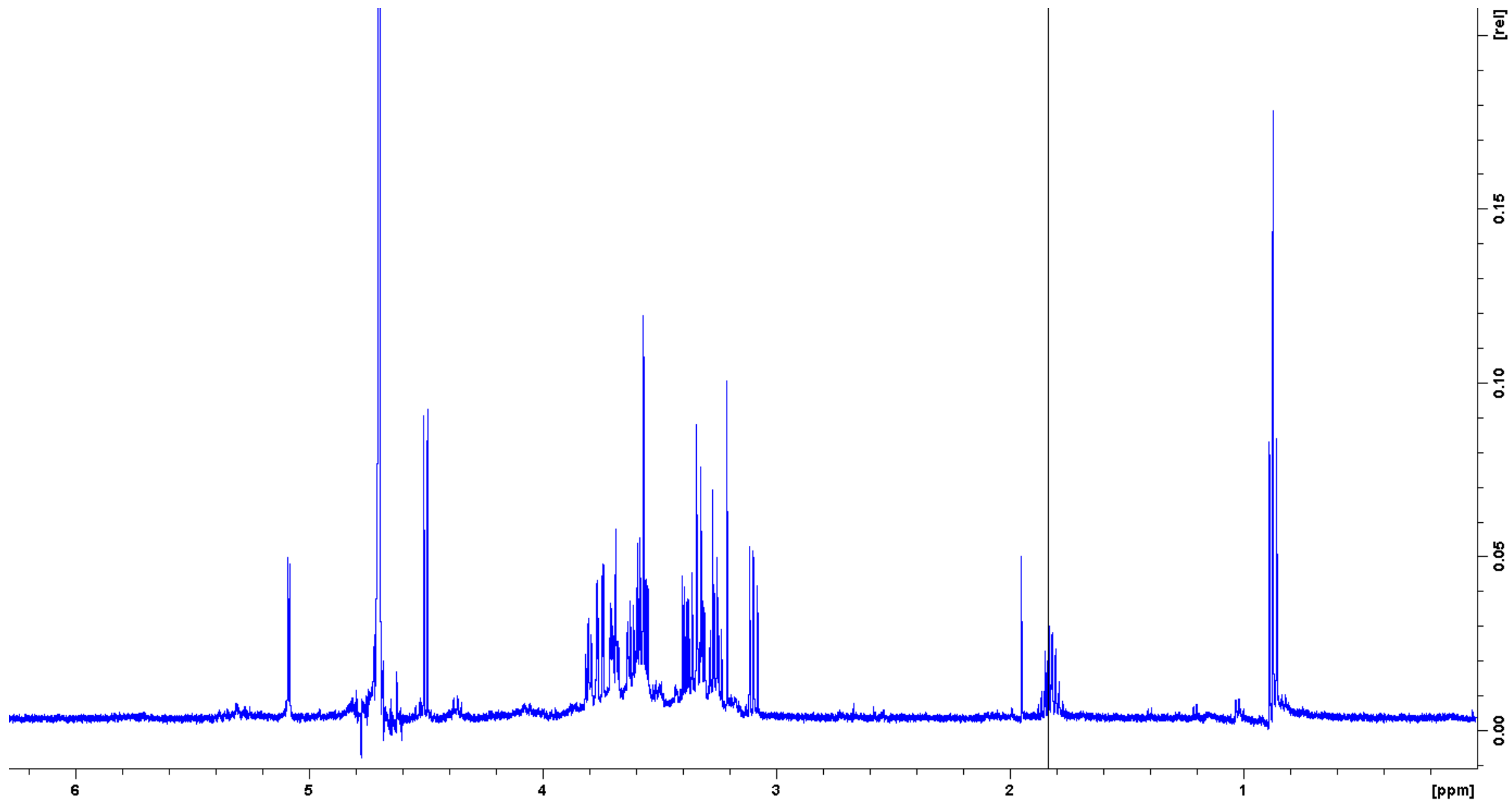

Figure S44. ^{1}H spectrum of aqueous extract of acid hydrolysis of myxoglucamide A acquired in D_2O at 500 MHz

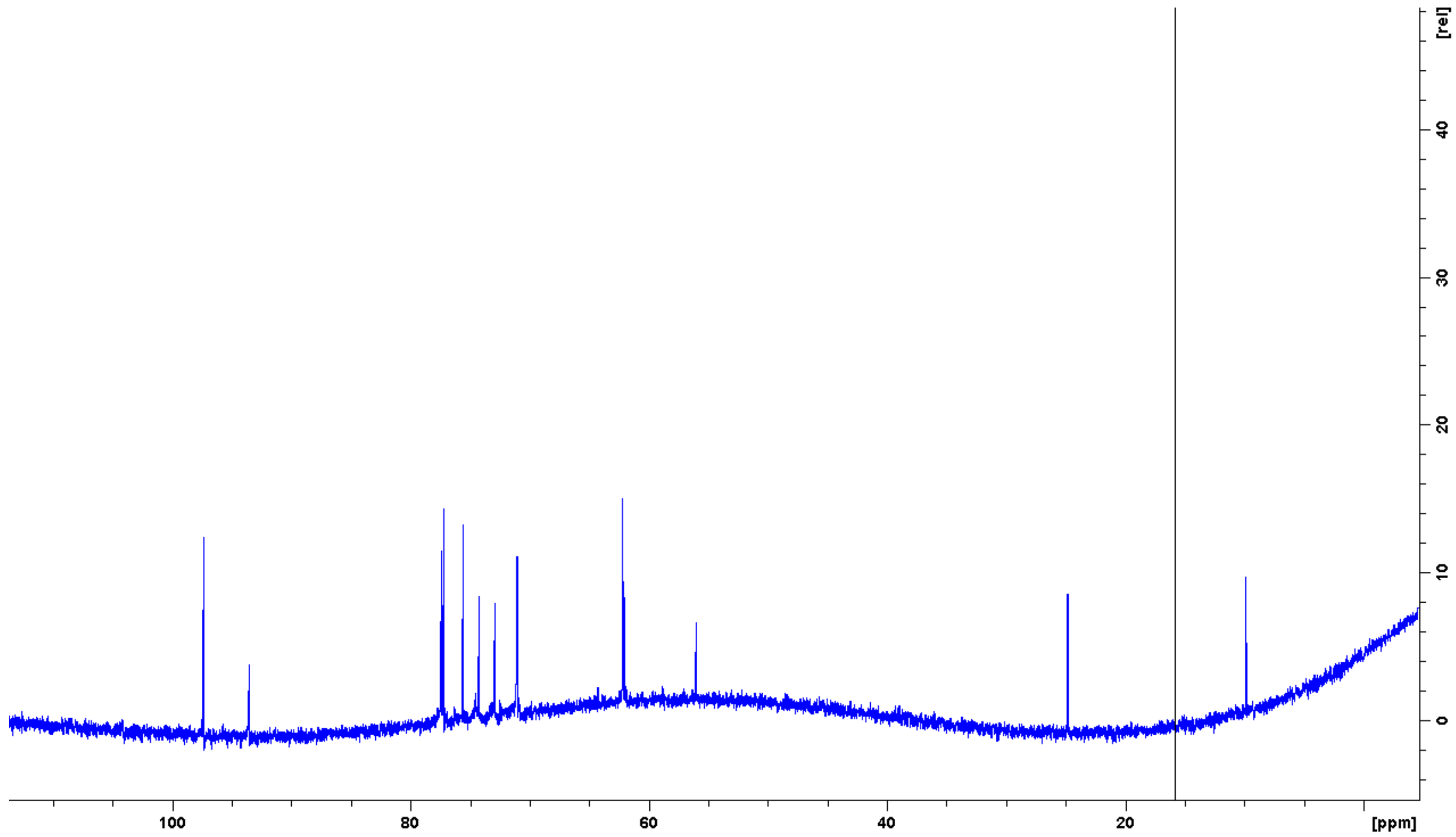

Figure S45. ^{13}C spectrum of aqueous extract of acid hydrolysis of myxoglucamide A acquired in D_2O at 125 MHz

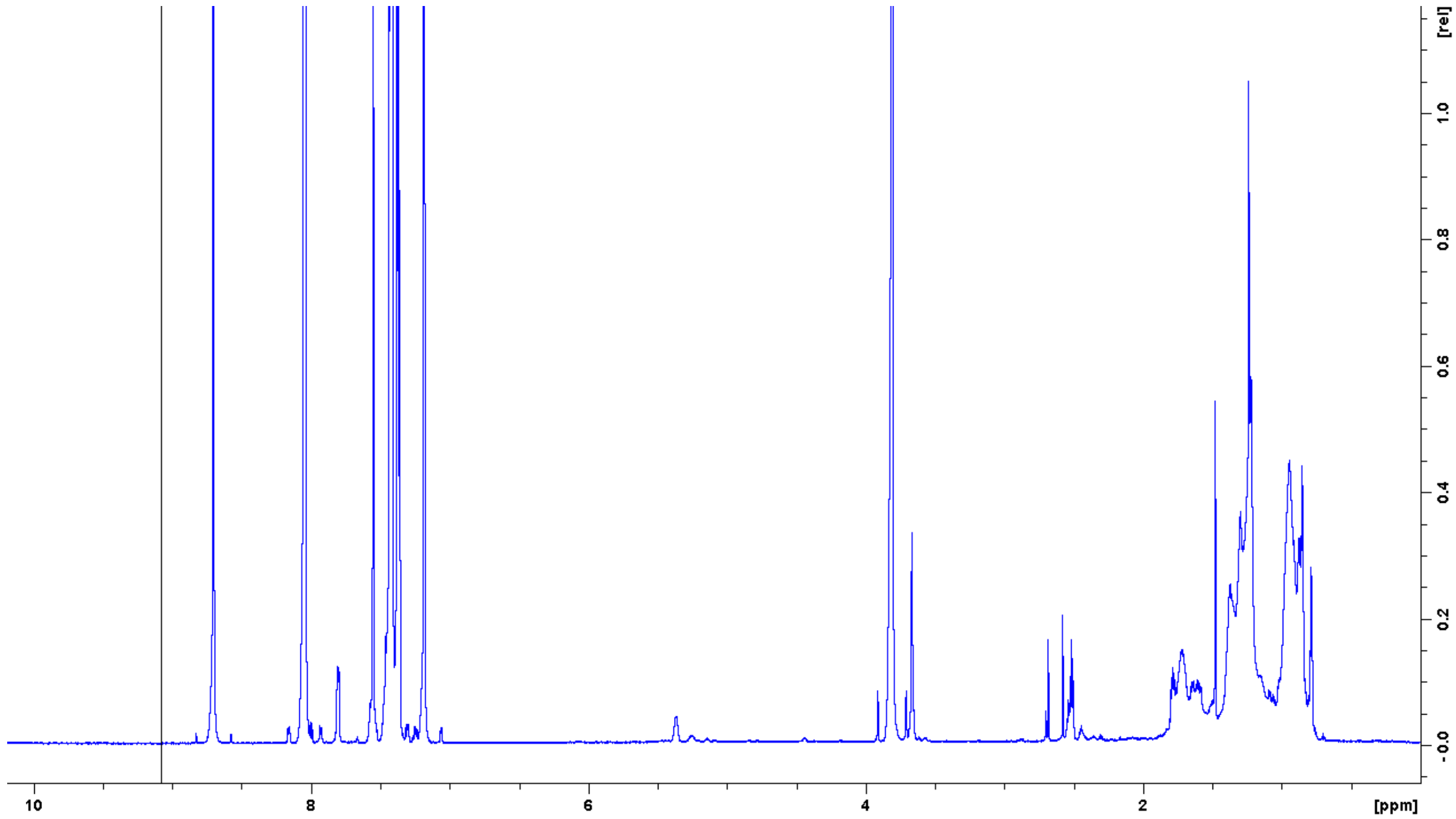

Figure S46. ^{1}H spectrum of the (*R*)-MTPA-HMHA ester reaction mixture acquired pyridine-d_5 at 700 MHz

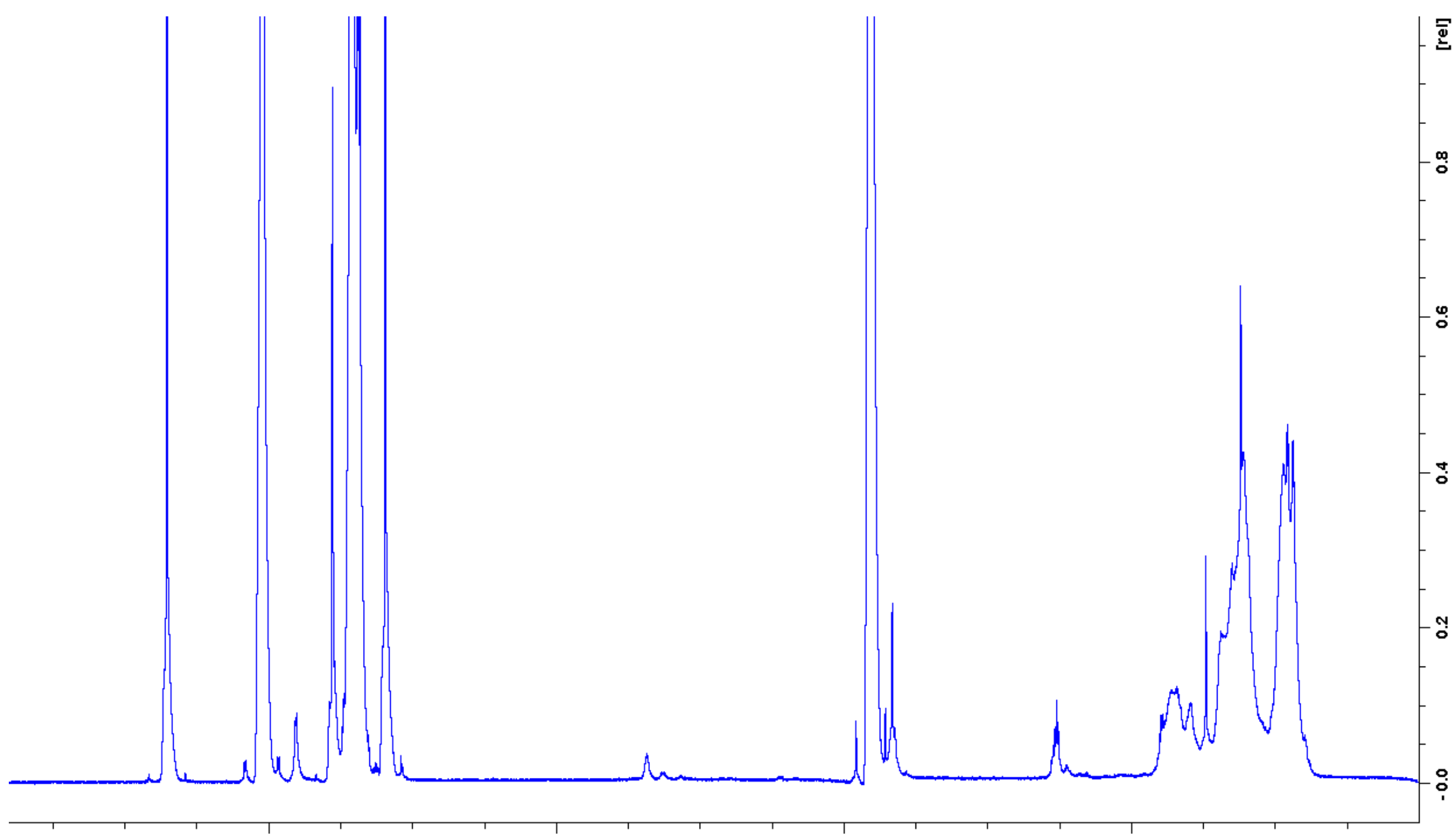

Figure S47. ^{1}H spectrum of the (*S*)-MTPA-HMHA ester reaction mixture acquired pyridine-d_5 at 700 MHz

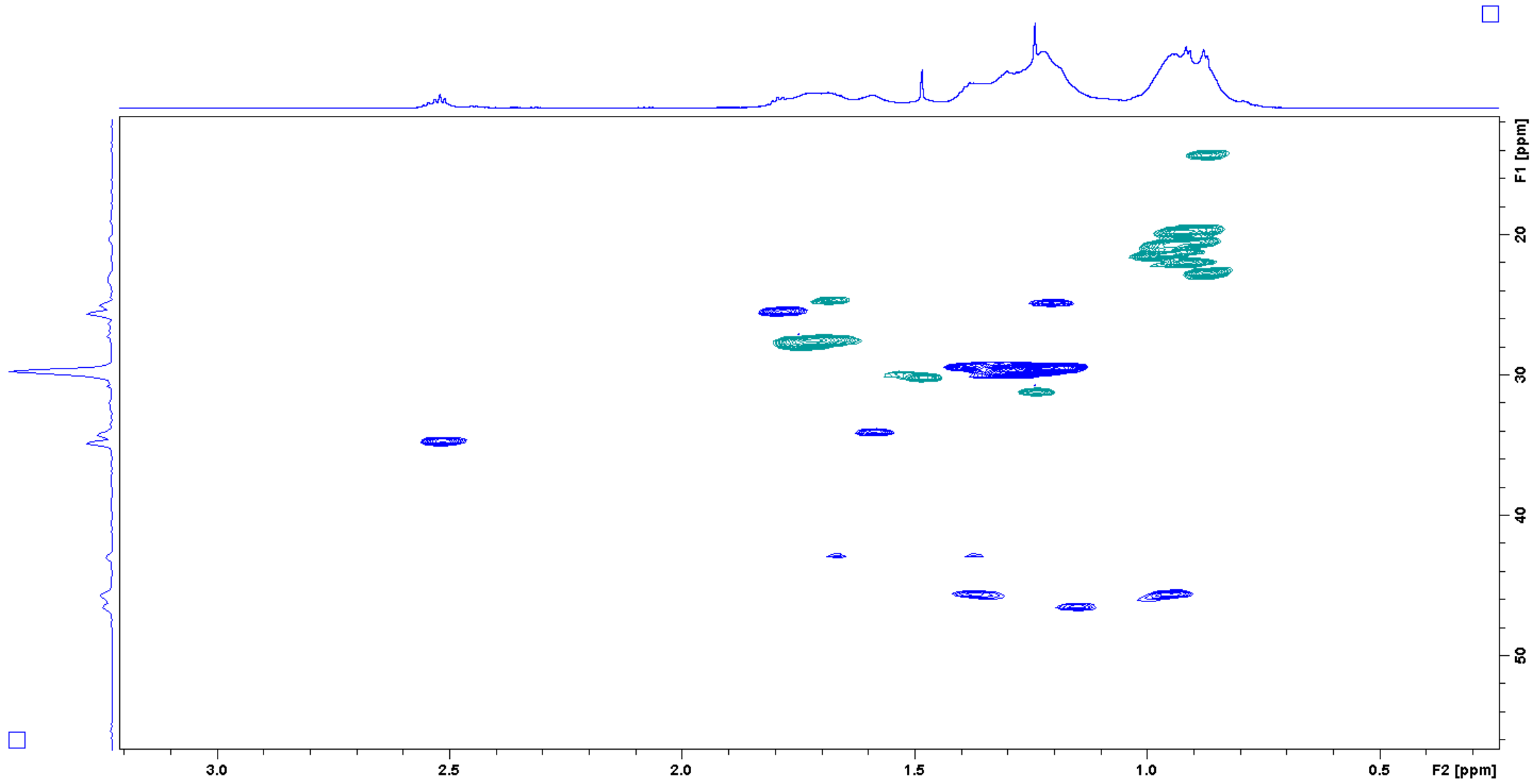

Figure S48. HSQC spectrum of the (*S*)-MTPA-HMHA ester reaction mixture acquired in pyridine-d_5 at 700/175 MHz

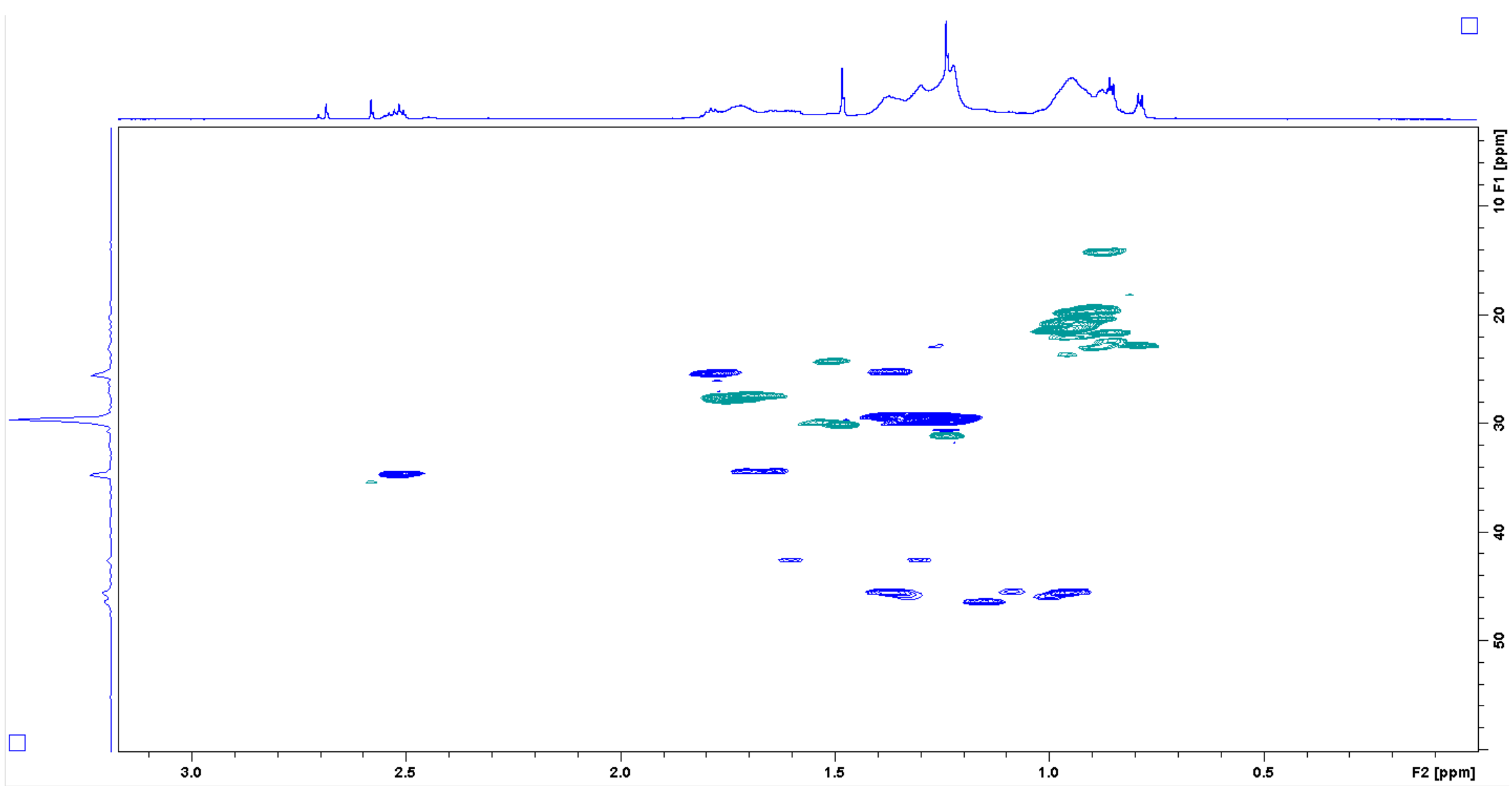

Figure S49. HSQC spectrum of the (*R*)-MTPA-HMHA Ester reaction mixture acquired in pyridine-d_5 at 700/175 MHz

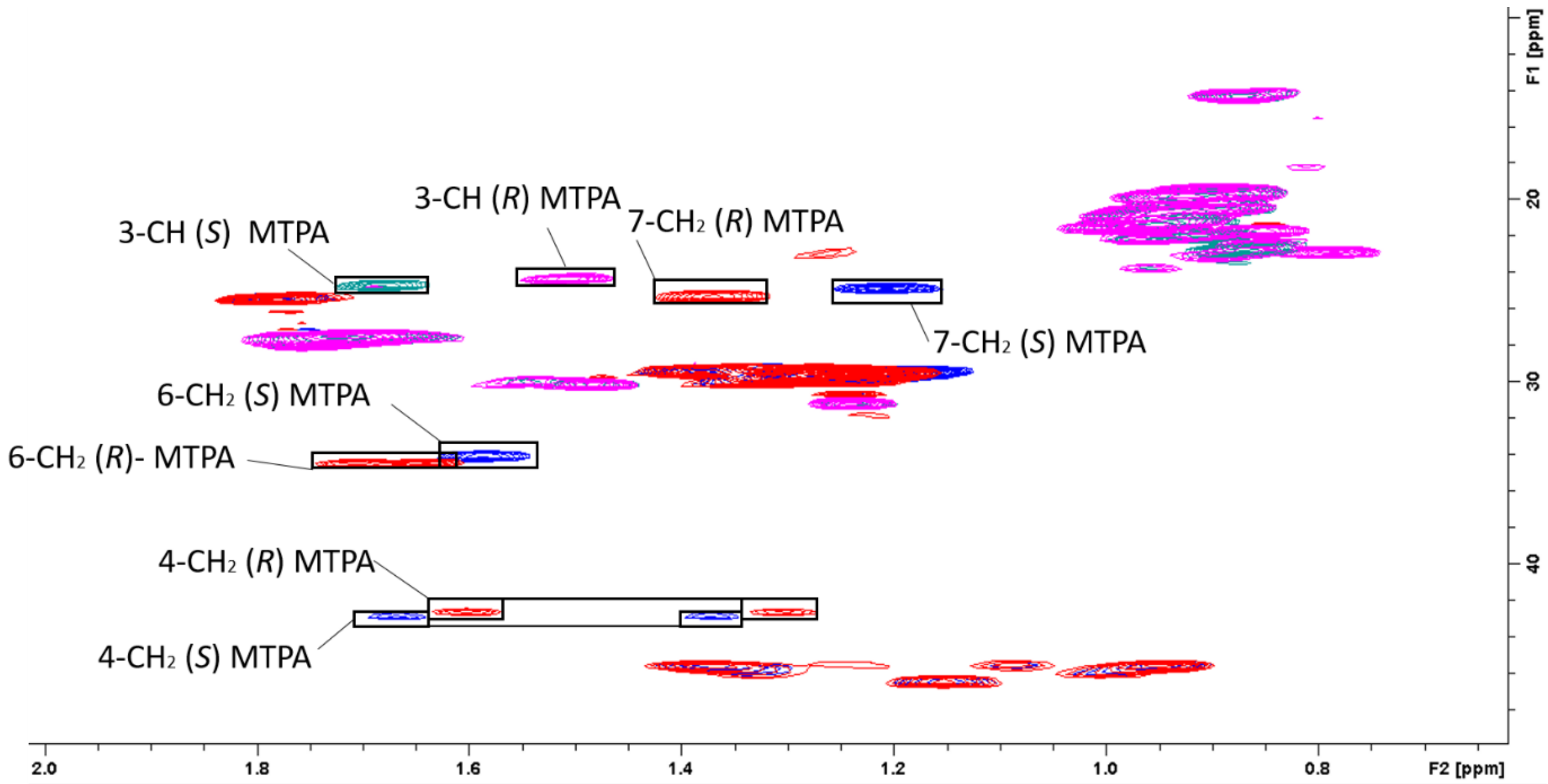

Figure S50. Overlapped HSQC spectra of (*R*)-MTPA-HMHA ester with CH_2 groups displayed in red, CH/CH_3 groups displayed in pink, and the (*S*)-MTPA-HMHA ester with CH_2 groups displayed in blue and CH/CH_3 groups displayed in green

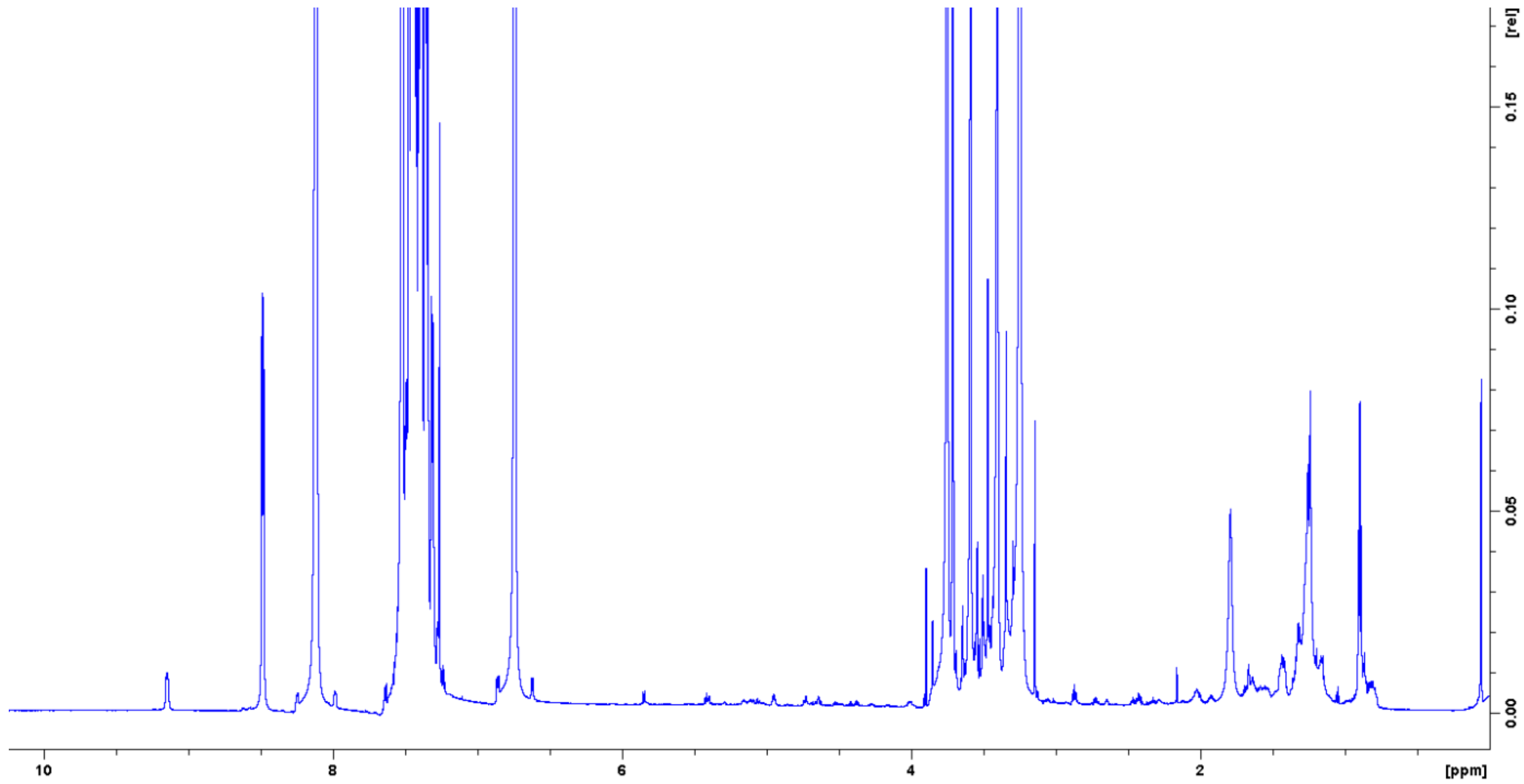

Figure S51. ^{1}H spectrum of the (*S*)-MTPA-HMPA Ester reaction mixture acquired pyridine-d_5 at 700 MHz

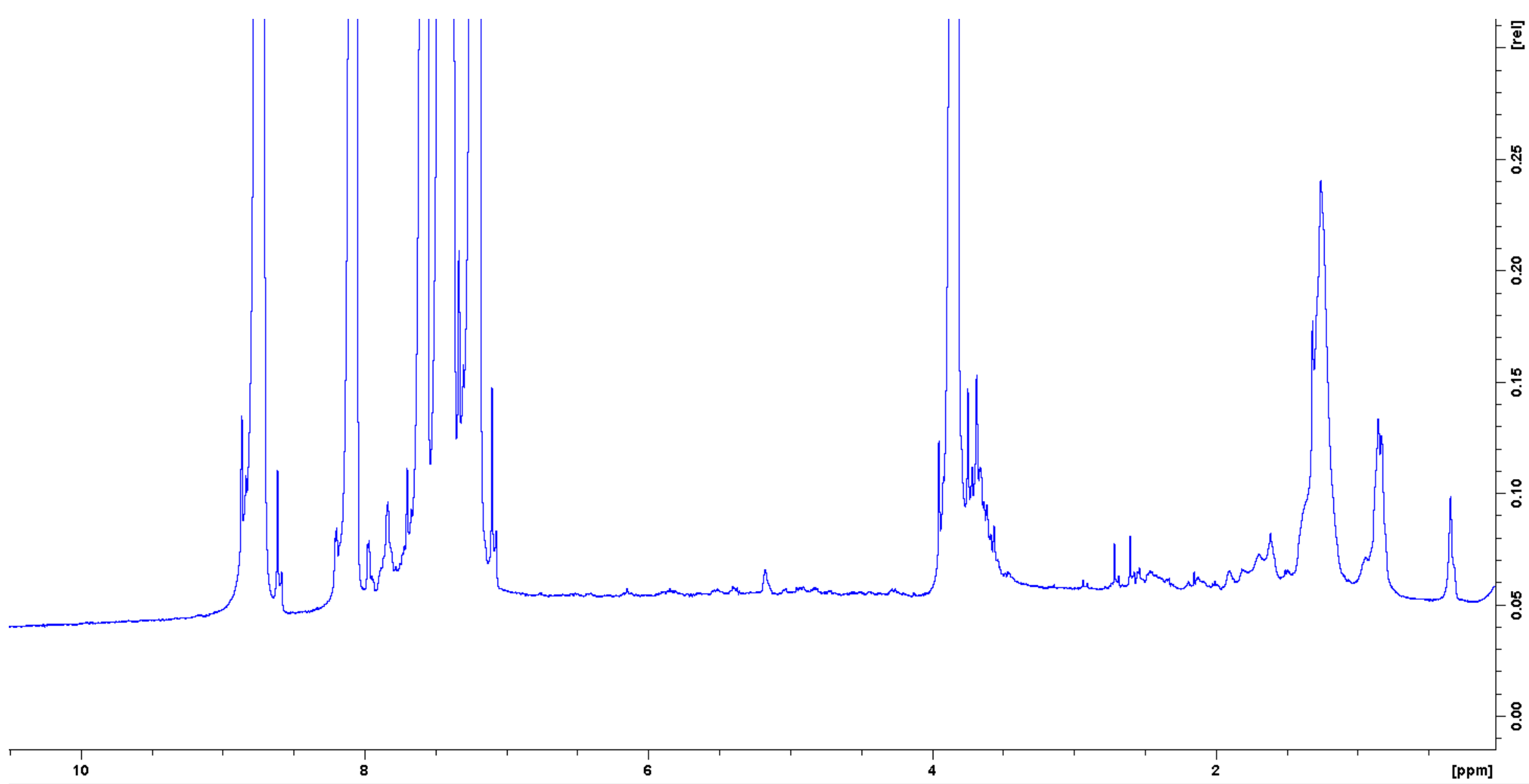

Figure S52. ^{1}H spectrum of the (*R*)-MTPA-HMPA Ester reaction mixture acquired pyridine-d_5 at 700 MHz

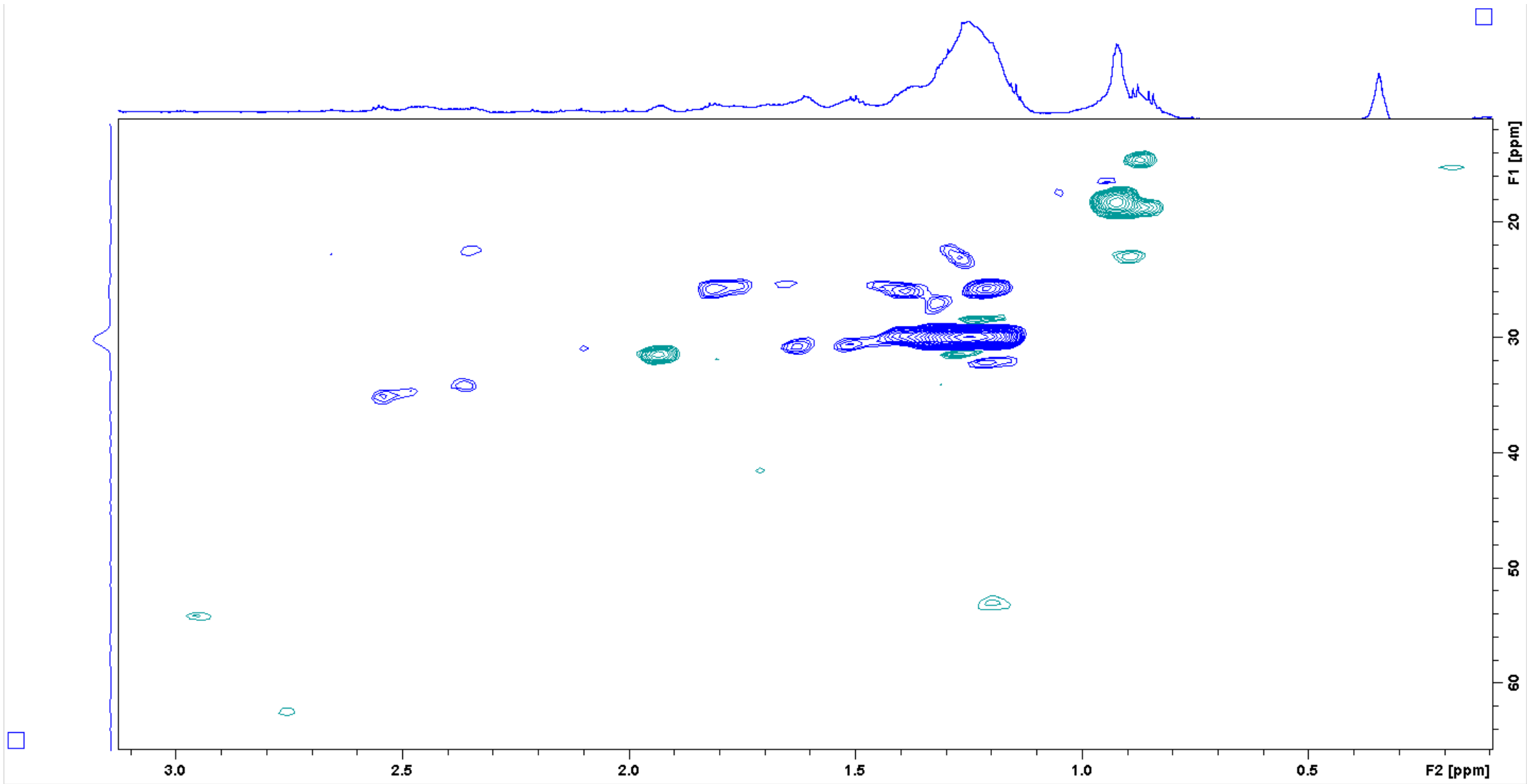

Figure S53. HSQC spectrum of the (*S*)-MTPA-HMPA Ester reaction mixture acquired in pyridine-d_5 at 700/175 MHz

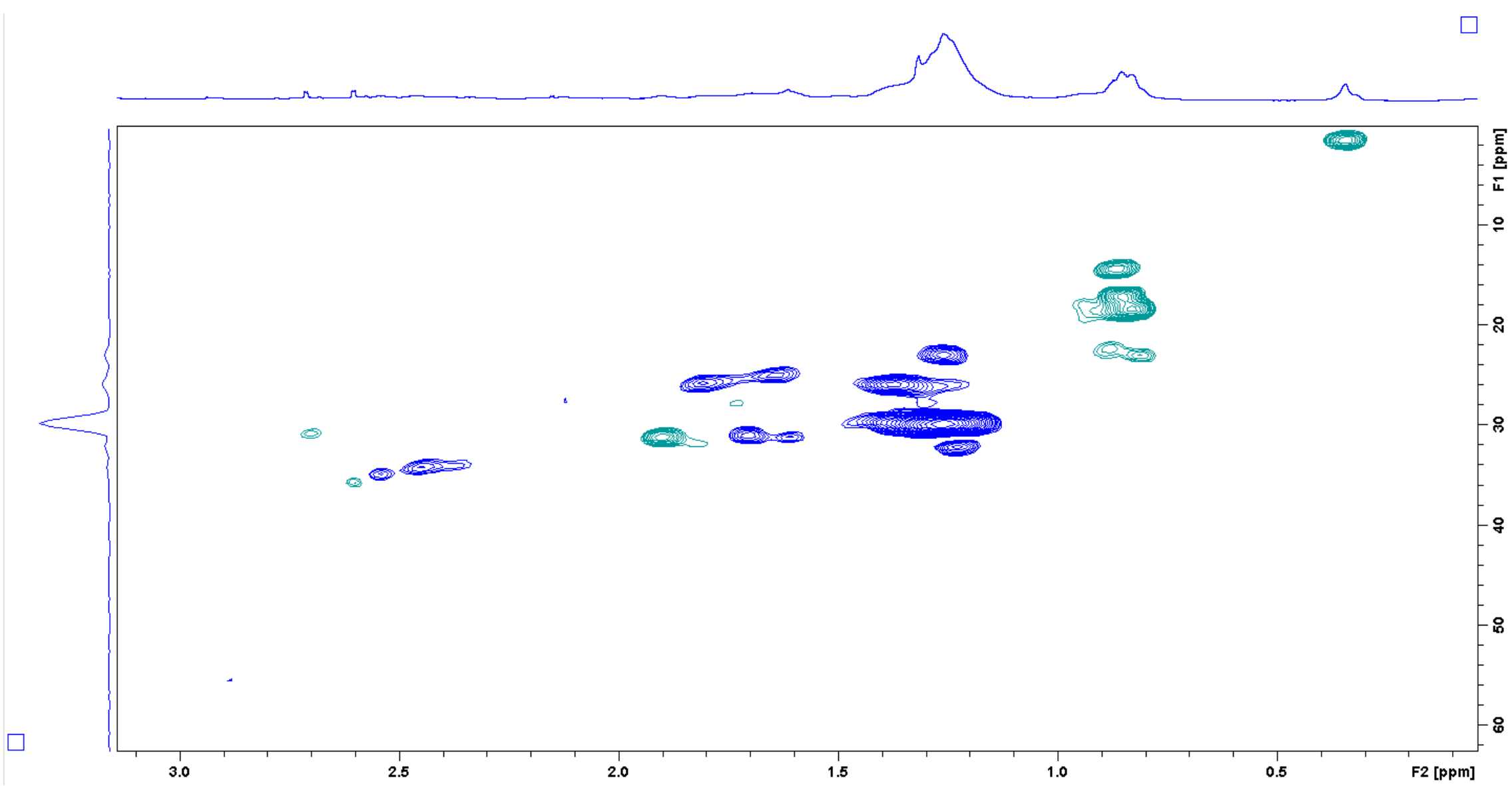

Figure S54. HSQC spectrum of the (*R*)-MTPA-HMPA ester reaction mixture acquired in pyridine-*d5* at 700/175 MHz

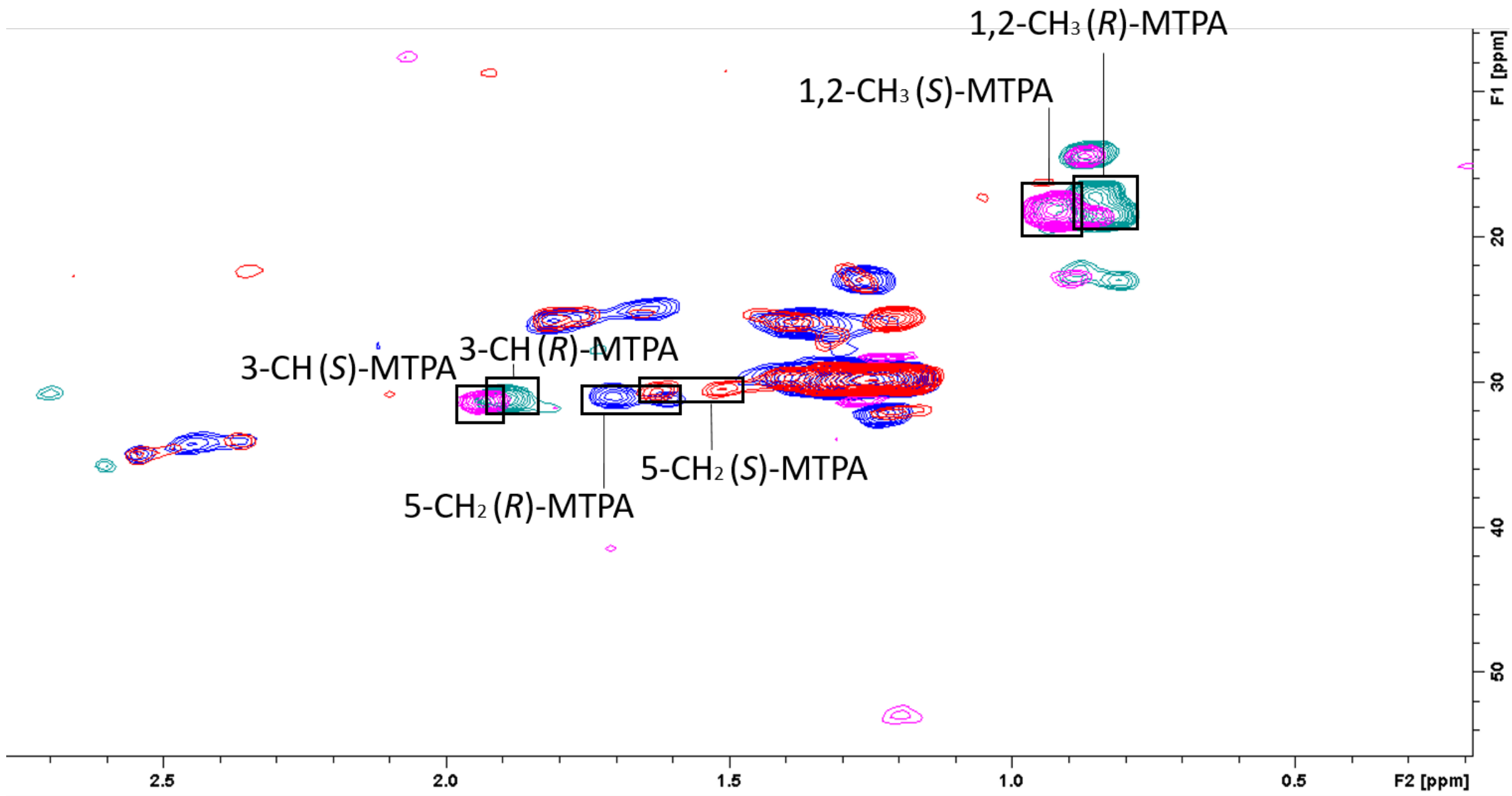

Figure S55. Overlapped HSQC spectra of (*S*)-MTPA-HMPA ester with CH_2 groups displayed in red, CH/CH_3 groups displayed in pink and (*R*)-MTPA-HMPA ester with CH_2 groups displayed in blue, CH/CH_3 groups displayed in green

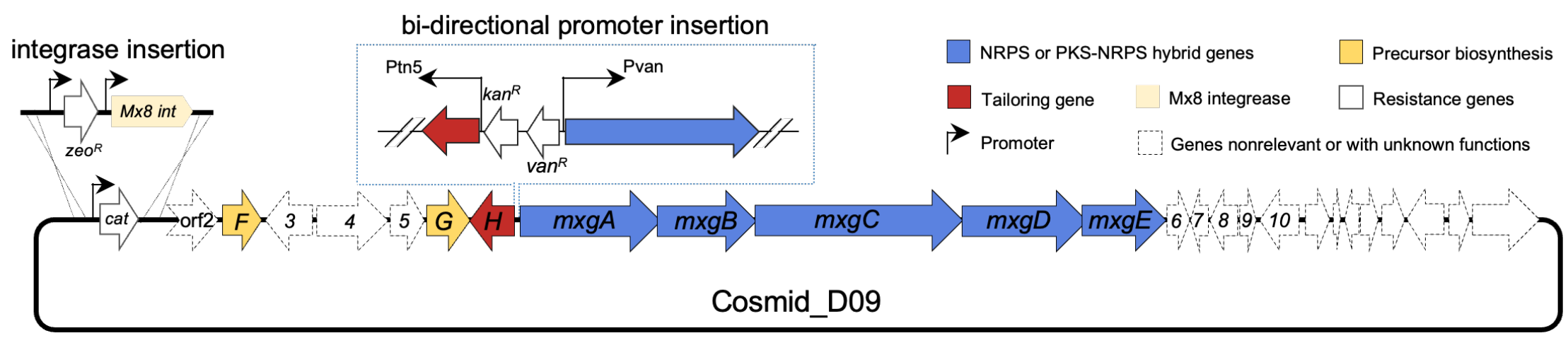

Figure S56. Modification of cosmid D09

Cosmid D09 was modified to enable heterologous expression of the myxoglucamide biosynthetic gene cluster in *M. xanthus* DK1622 by introducing an integrase cassette and a bidirectional promoter cassette. First, the Mx8 phage integrase gene[11] was introduced into cosmid D09 via Redαβ linear-circular homologous recombination. During this process, the chloramphenicol resistance gene (*cat*) was replaced with a zeocin resistance gene (*zeo*), yielding cosmid Mx8D09. Second, an ampicillin resistance gene (*ampR*) flanked by two *Spe*I restriction sites was inserted between mxgH and mxgA via Redαβ recombination. The resulting cosmid was digested with *Spe*I, and the larger fragment was purified. Third, the larger fragment was assembled with a bidirectional promoter cassette (*tn5*-*KanR*-*VanR*-*van*) using Gibson assembly, generating the final construct, designated BiPD09. BiPD09 was confirmed by restriction enzyme digestion and Sanger sequencing.

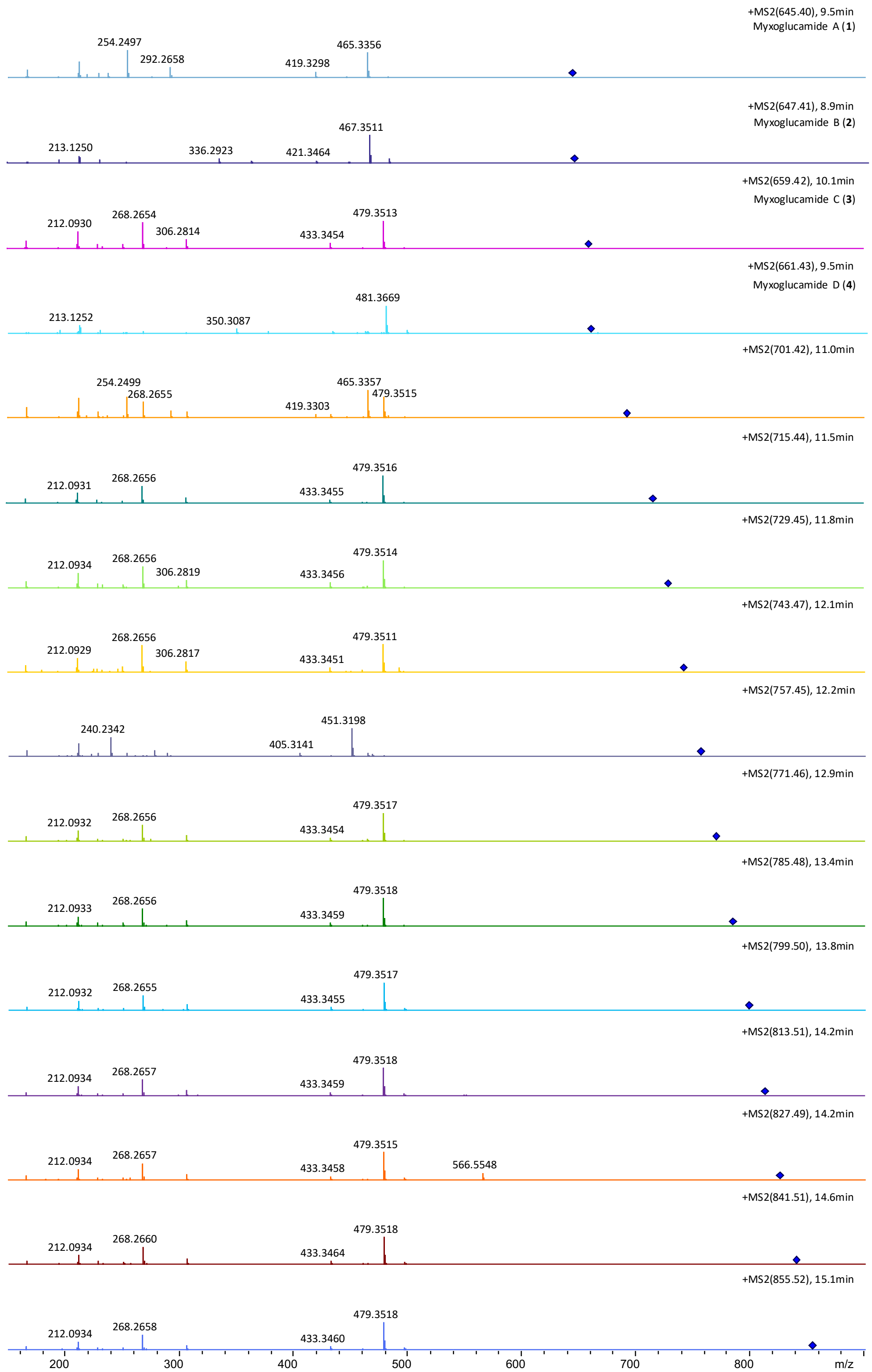

Figure S57. MS^2 fragmentation of myxoglucamides and their congeners from crude extracts

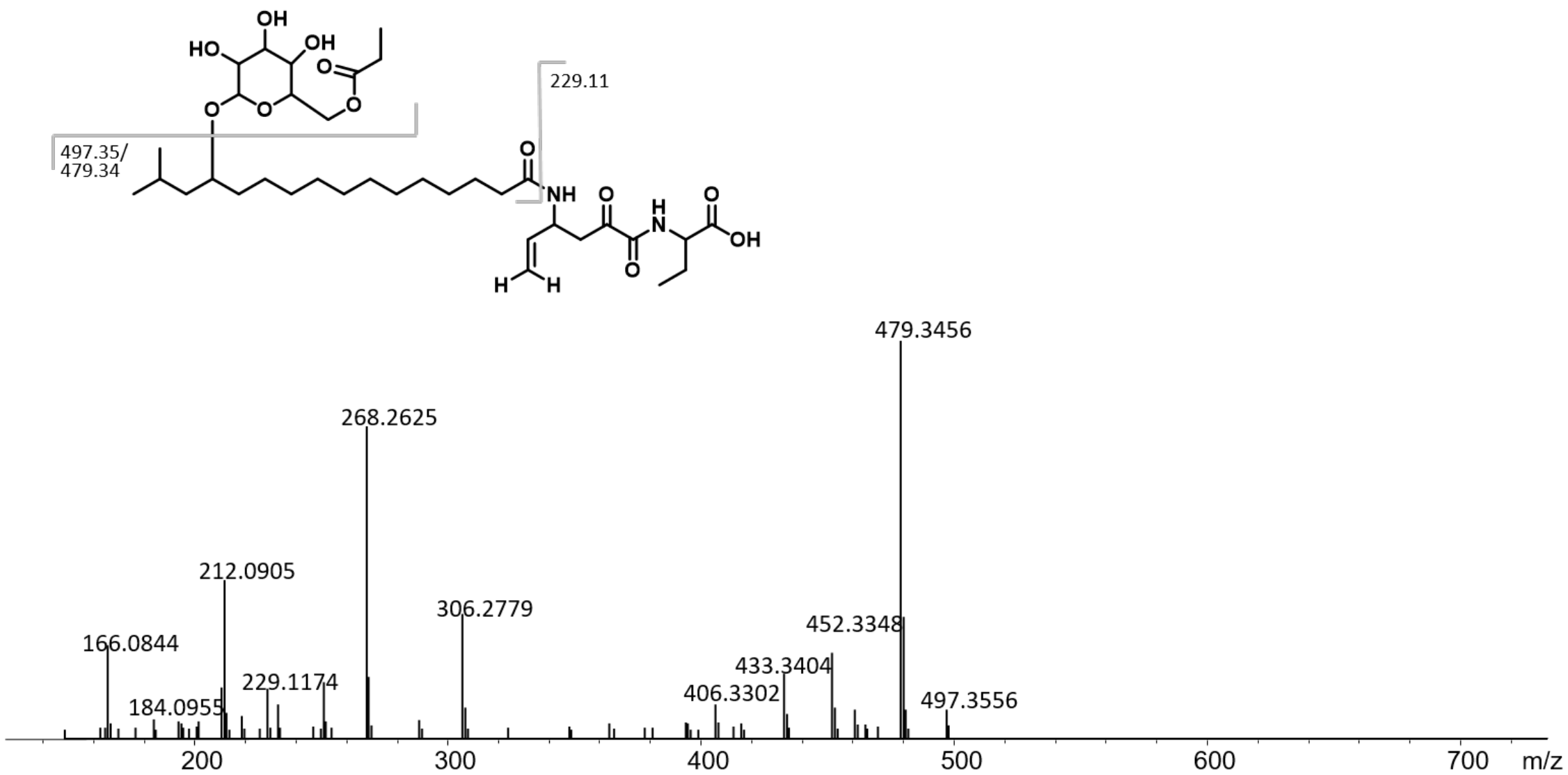

Figure S58. HRMS^2 spectrum and observed fragments of myxoglucamide E

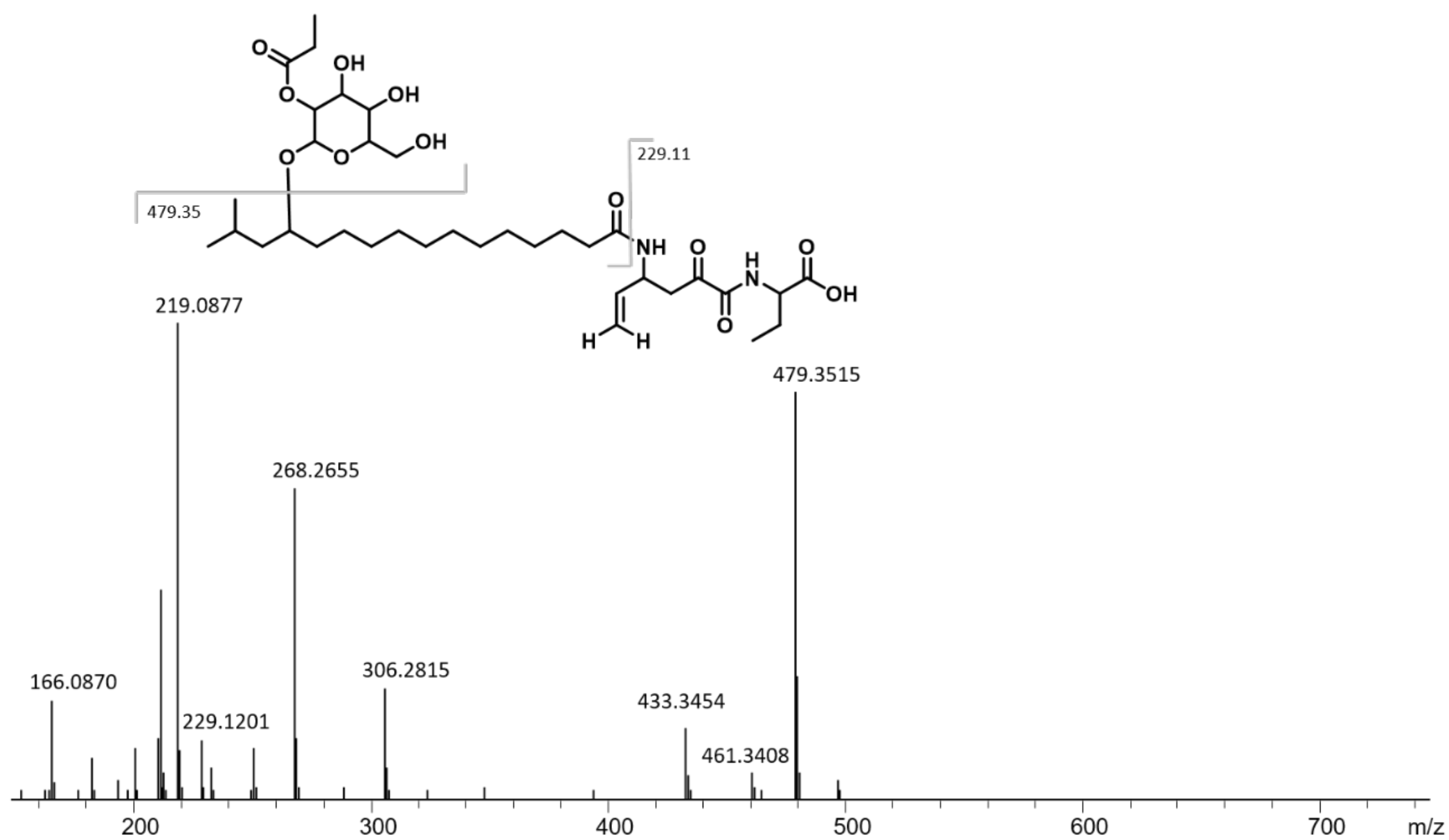

Figure S59. HRMS^2 spectrum and observed fragments of myxoglucamide F

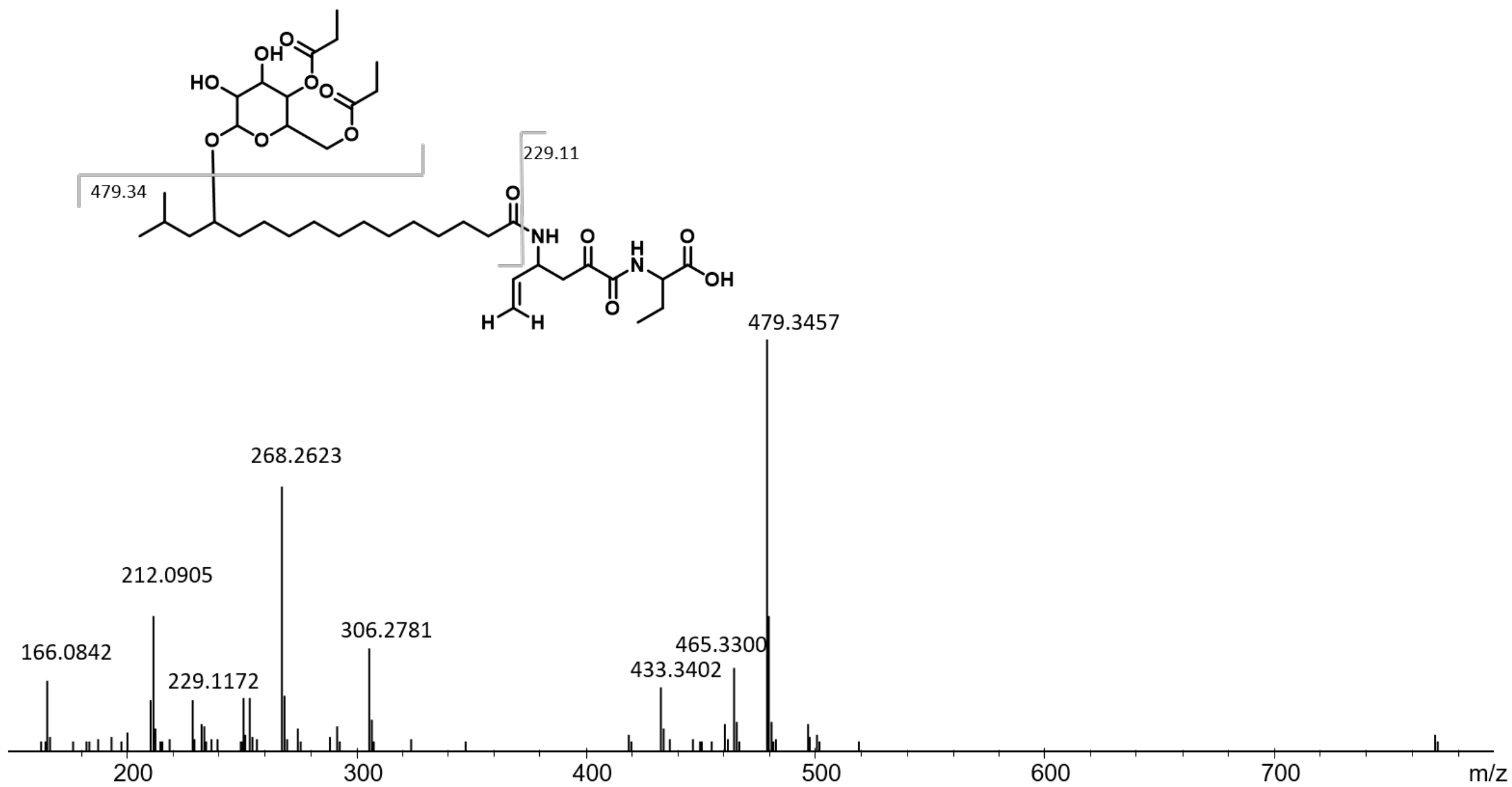

Figure S60. HRMS^2 spectrum and observed fragments of myxoglucamide G

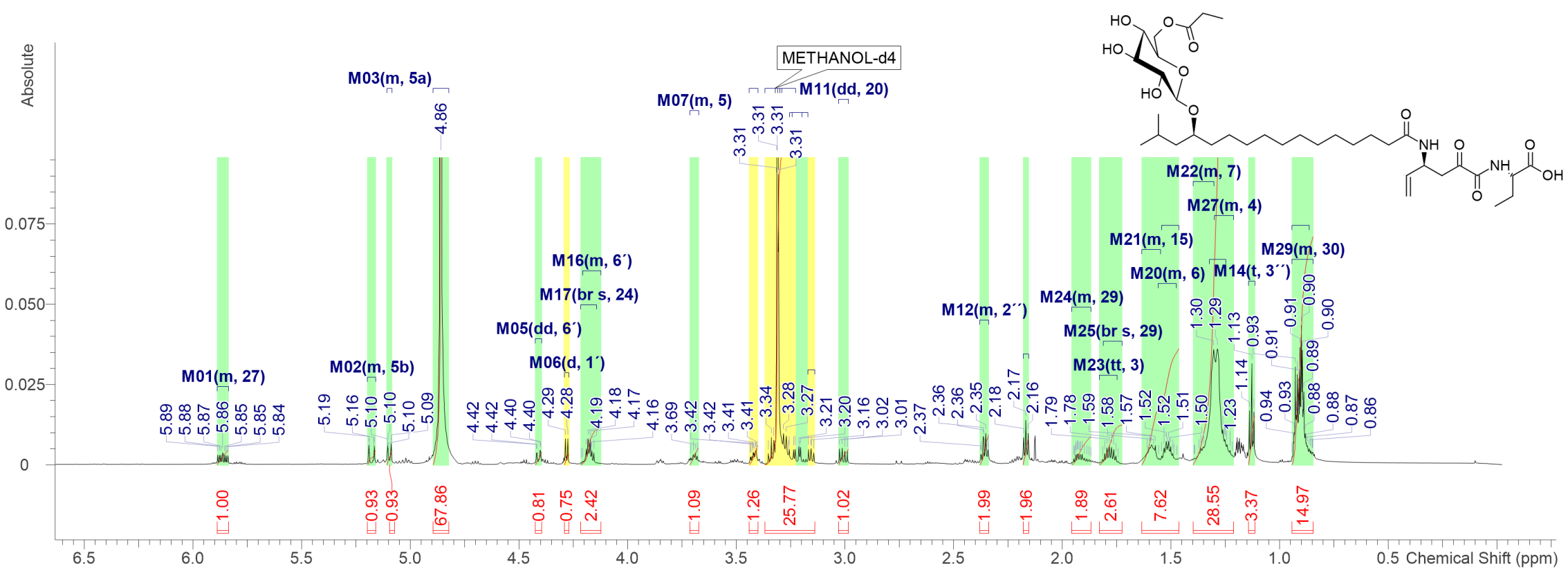

Figure S61. ^{1}H spectrum of myxoglucamide E acquired in CD_3OD at 700 MHz

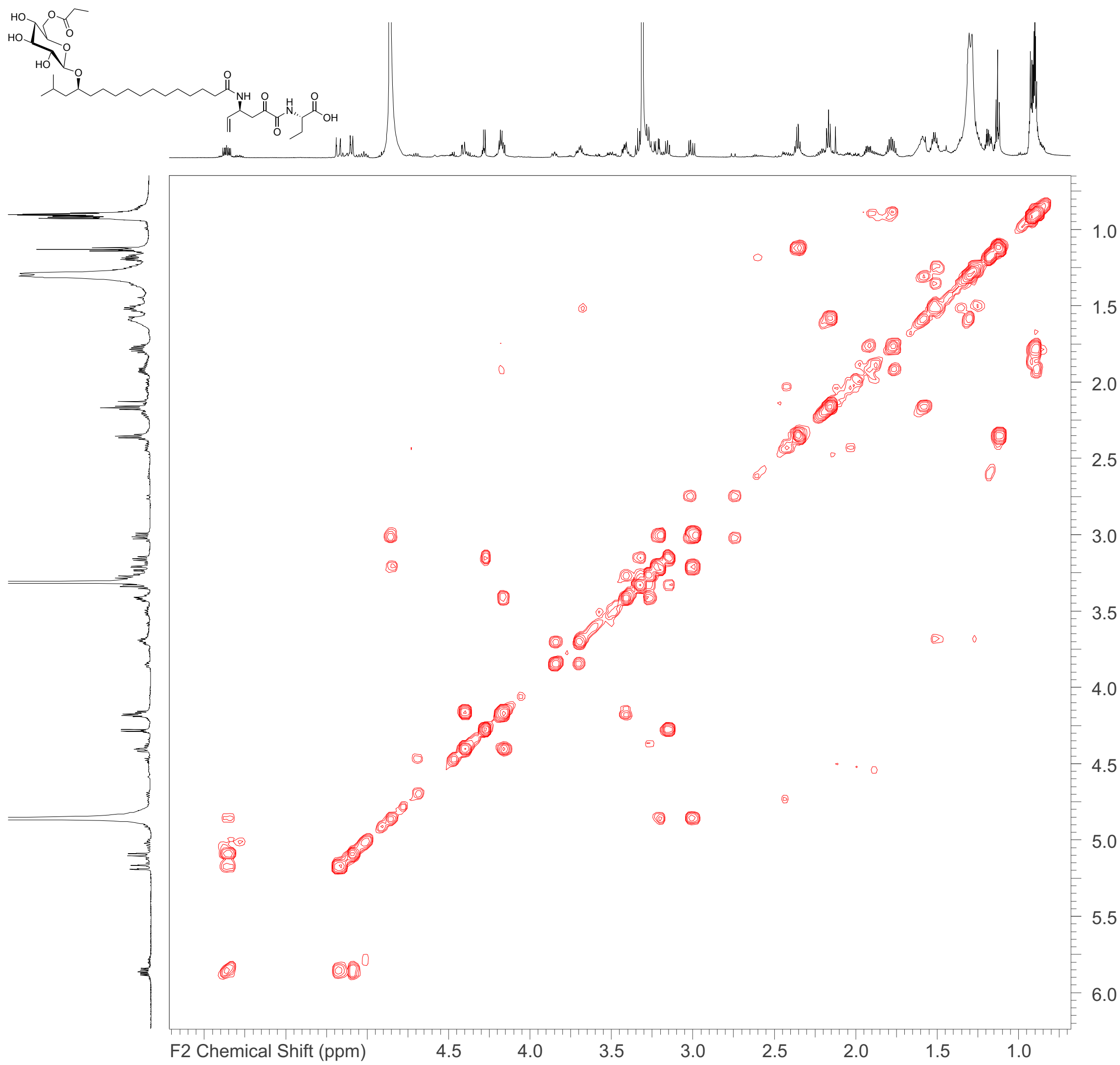

Figure S62. COSY spectrum of myxoglucamide E acquired in CD_3OD at 700 MHz

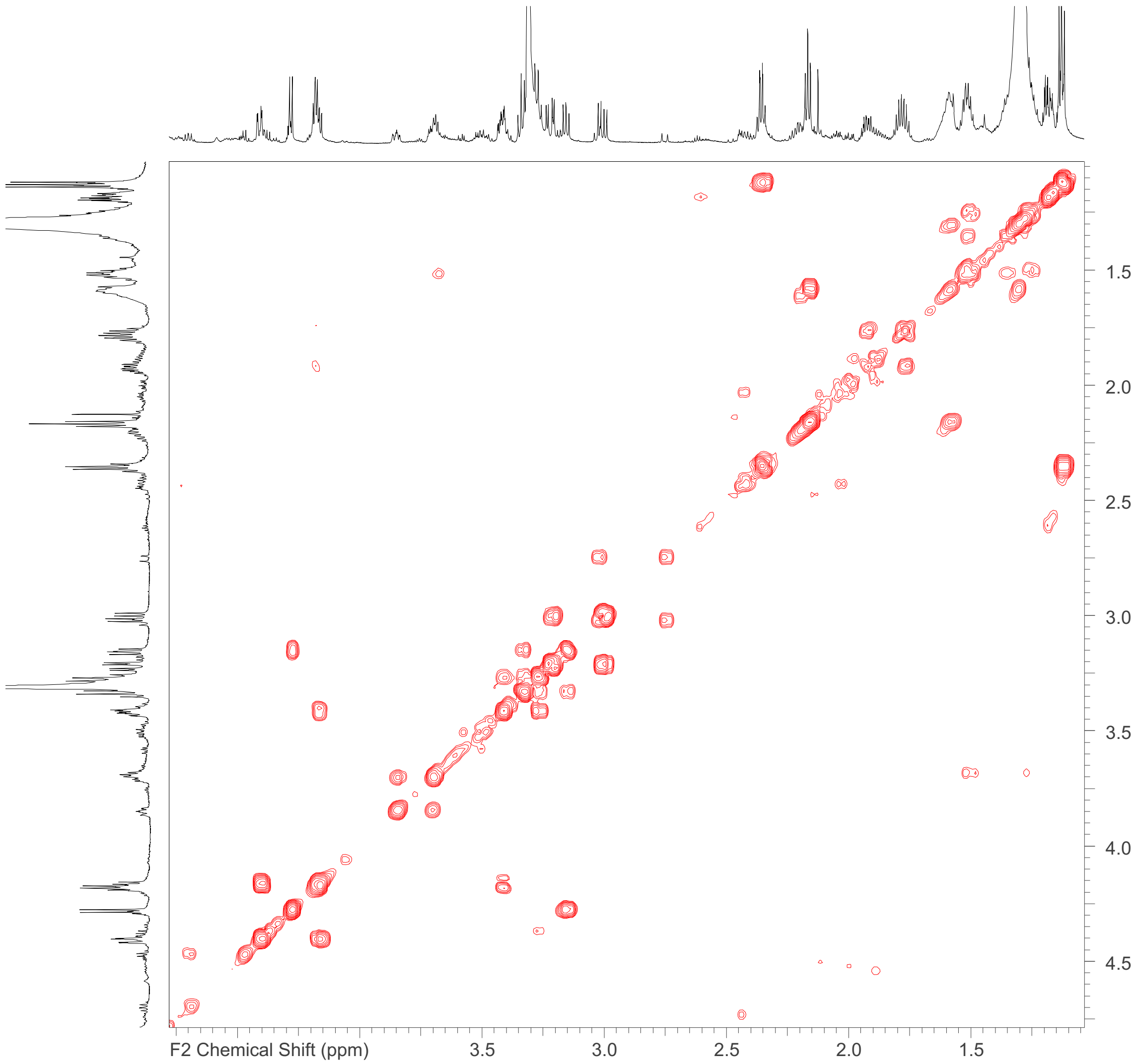

Figure S63. Enlarged COSY spectrum of myxoglucamide E acquired in CD_3OD at 700 MHz

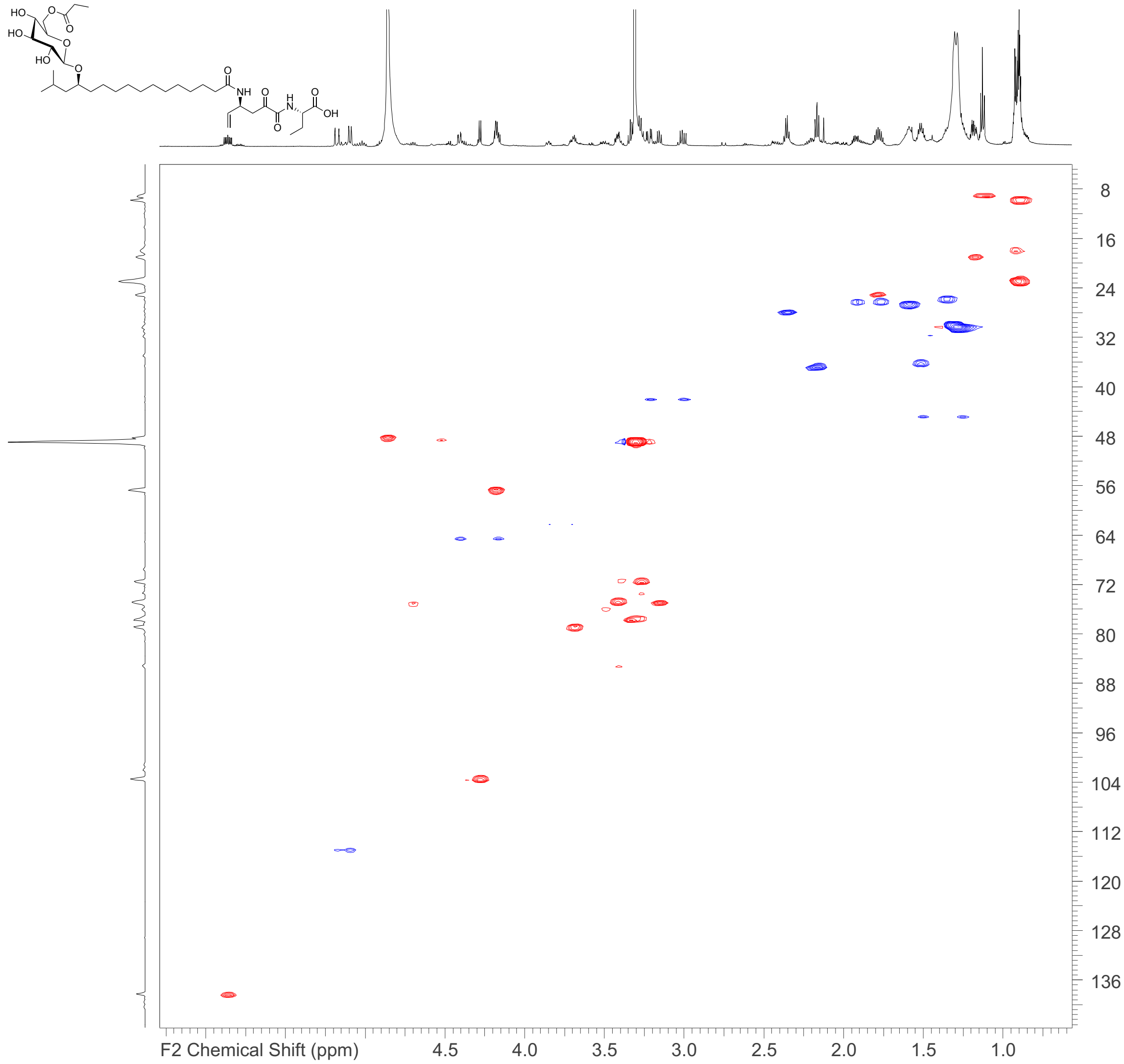

Figure S64. HSQC spectrum of myxoglucamide E acquired in CD_3OD at 700/ 175 MHz

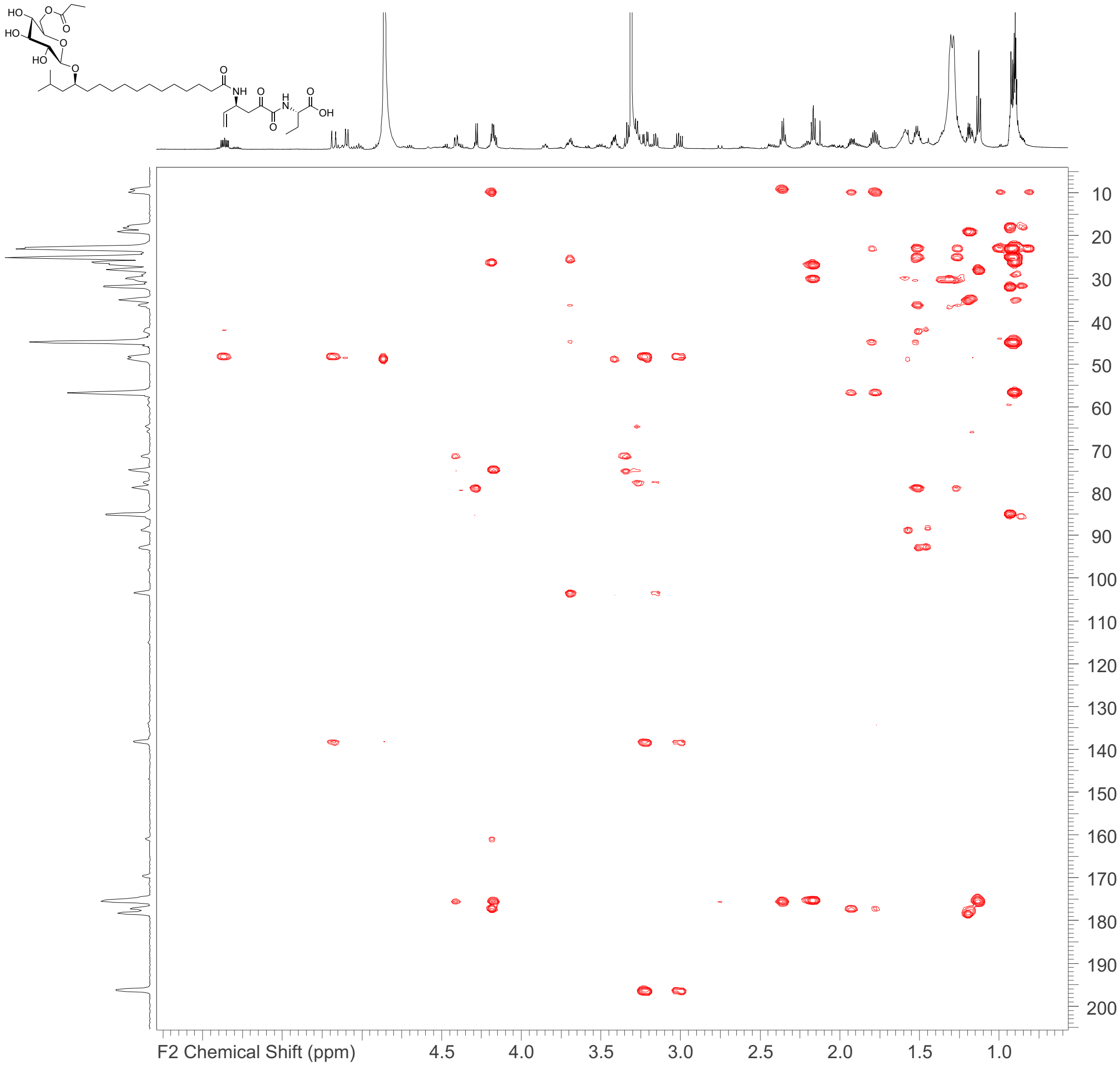

Figure S65. HMBC spectrum of myxoglucamide E acquired in CD_3OD at 700/ 175 MHz

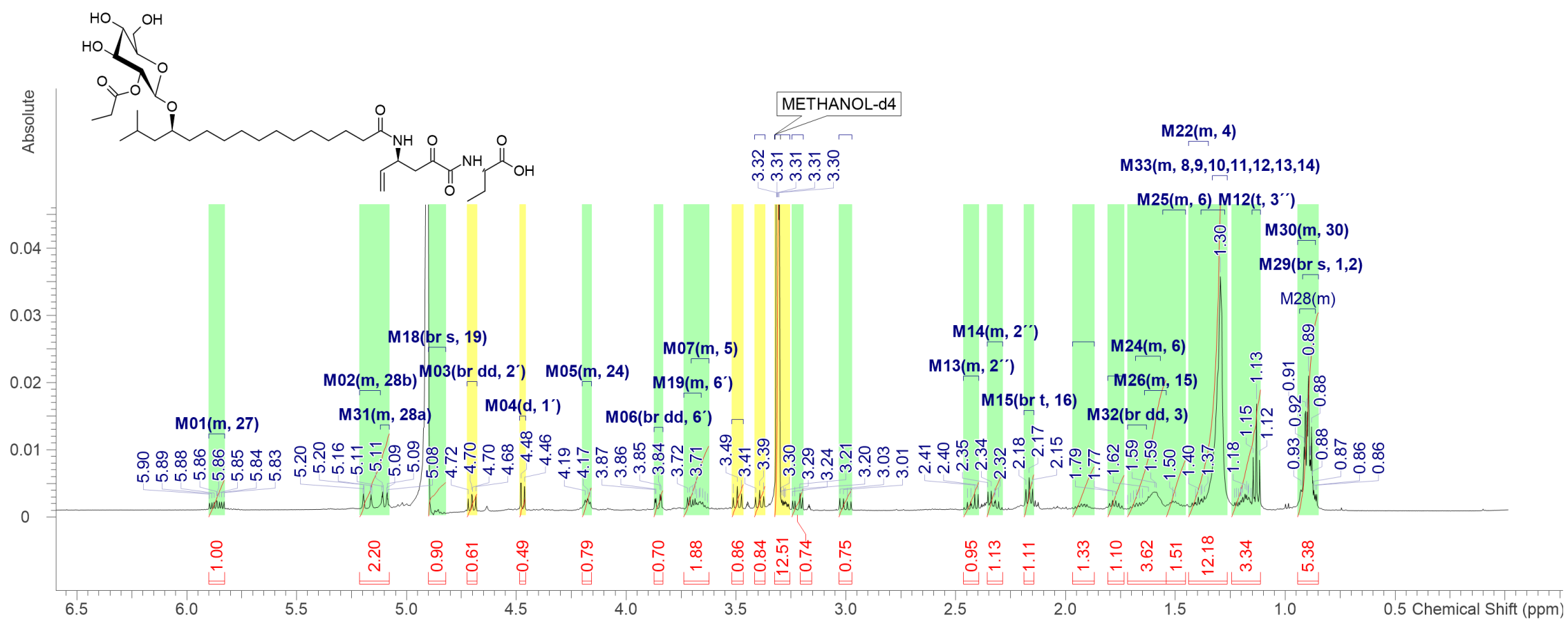

Figure S66. ^{1}H spectrum of myxoglucamide F acquired in CD_3OD at 700 MHz

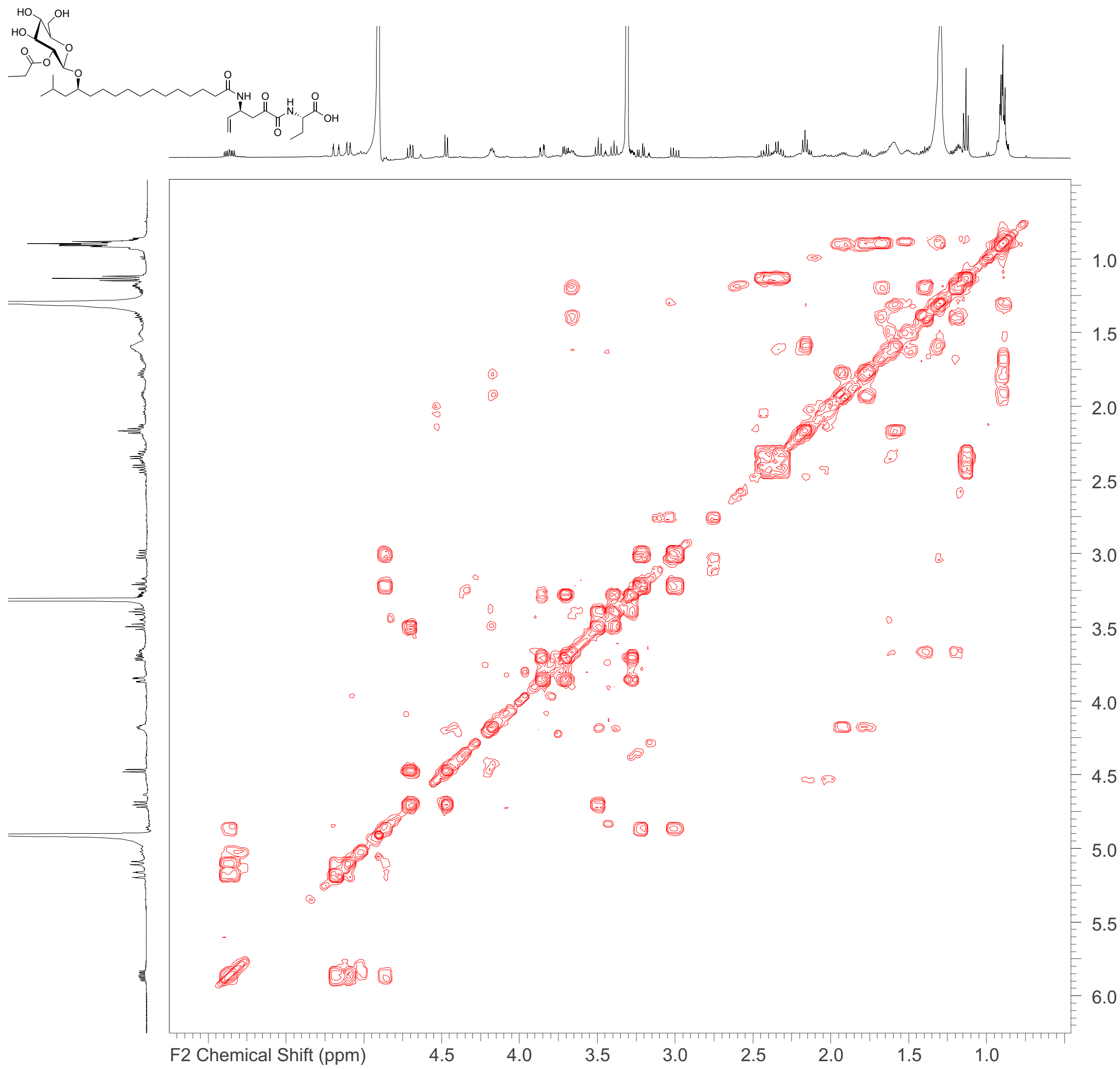

Figure S67. COSY spectrum of myxoglucamide F acquired in CD_3OD at 500 MHz

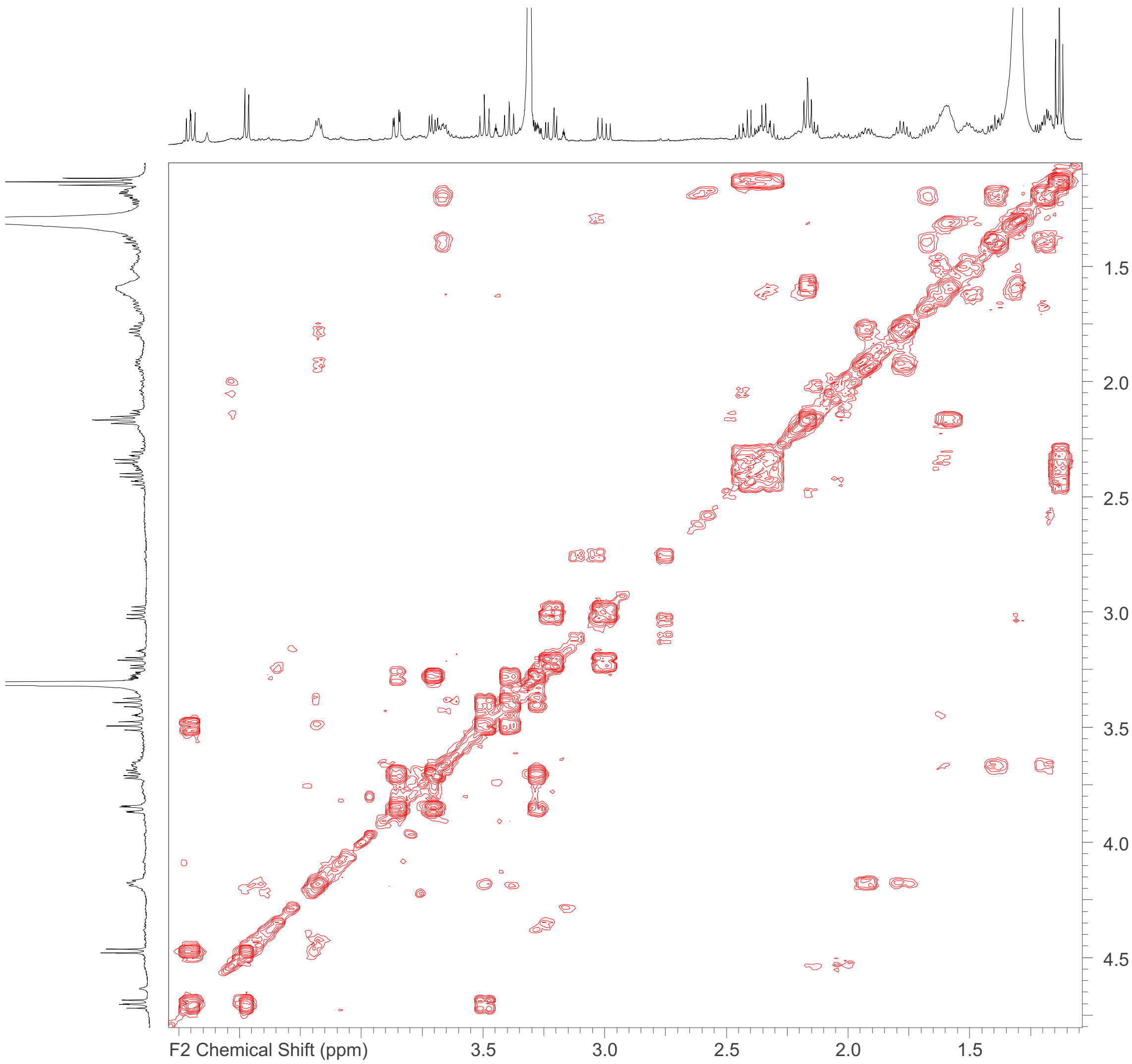

Figure S68. Enlarged COSY spectrum of myxoglucamide F acquired in CD_3OD at 500 MHz

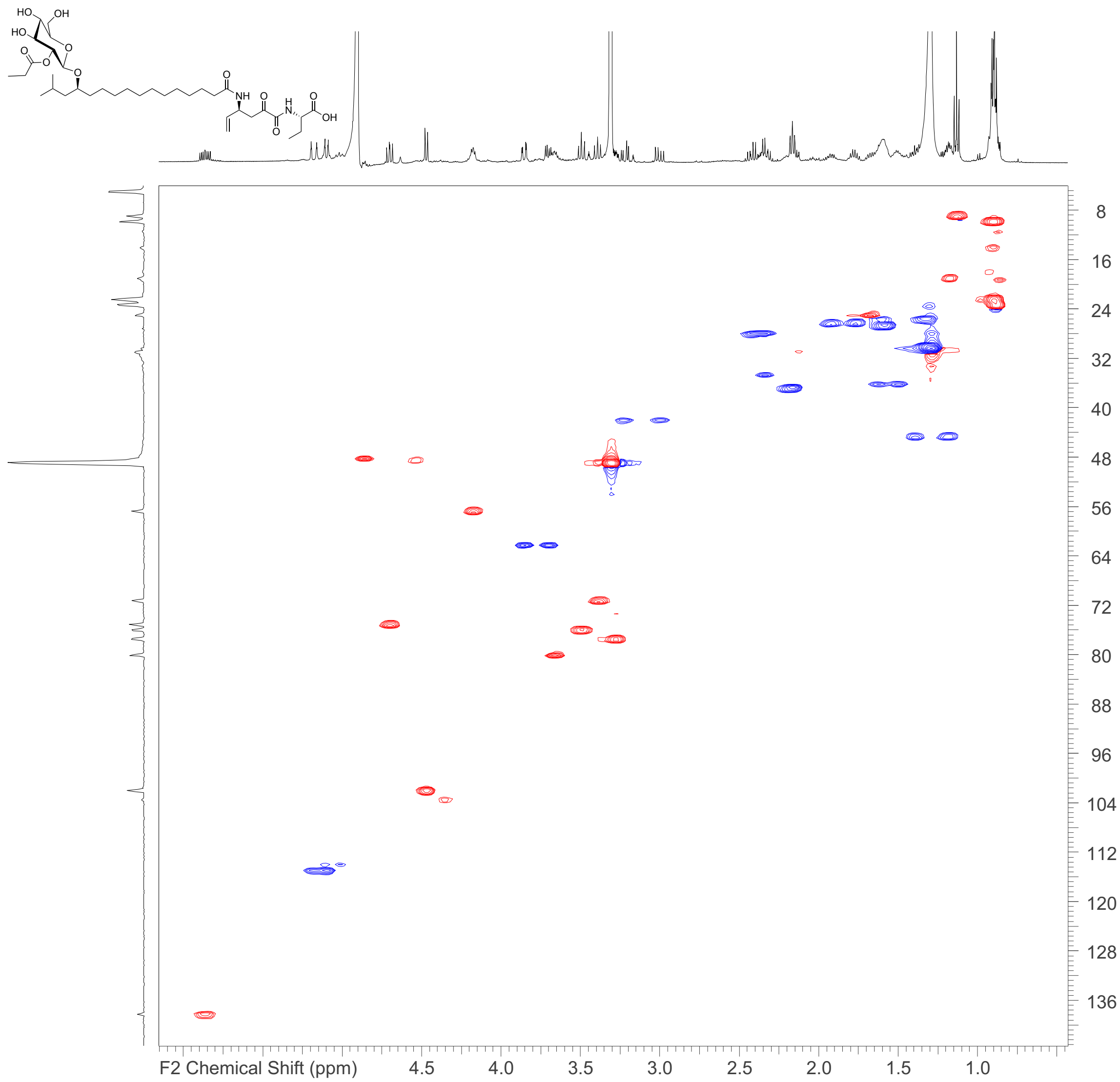

Figure S69. HSQC spectrum of myxoglucamide F acquired in CD_3OD at 500/125 MHz

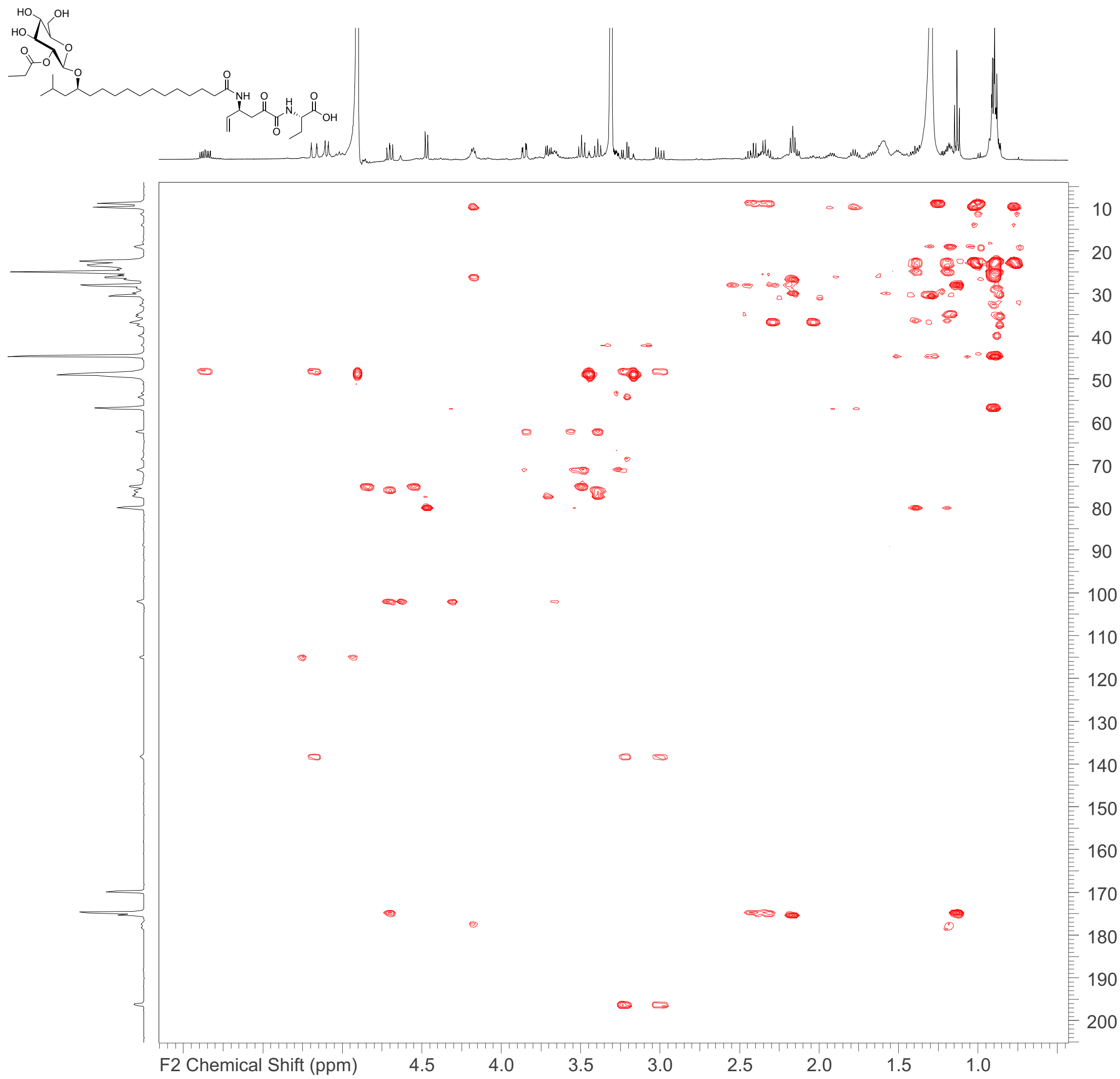

Figure S70. HMBC spectrum of myxoglucamide F acquired in CD_3OD at 500/ 125 MHz

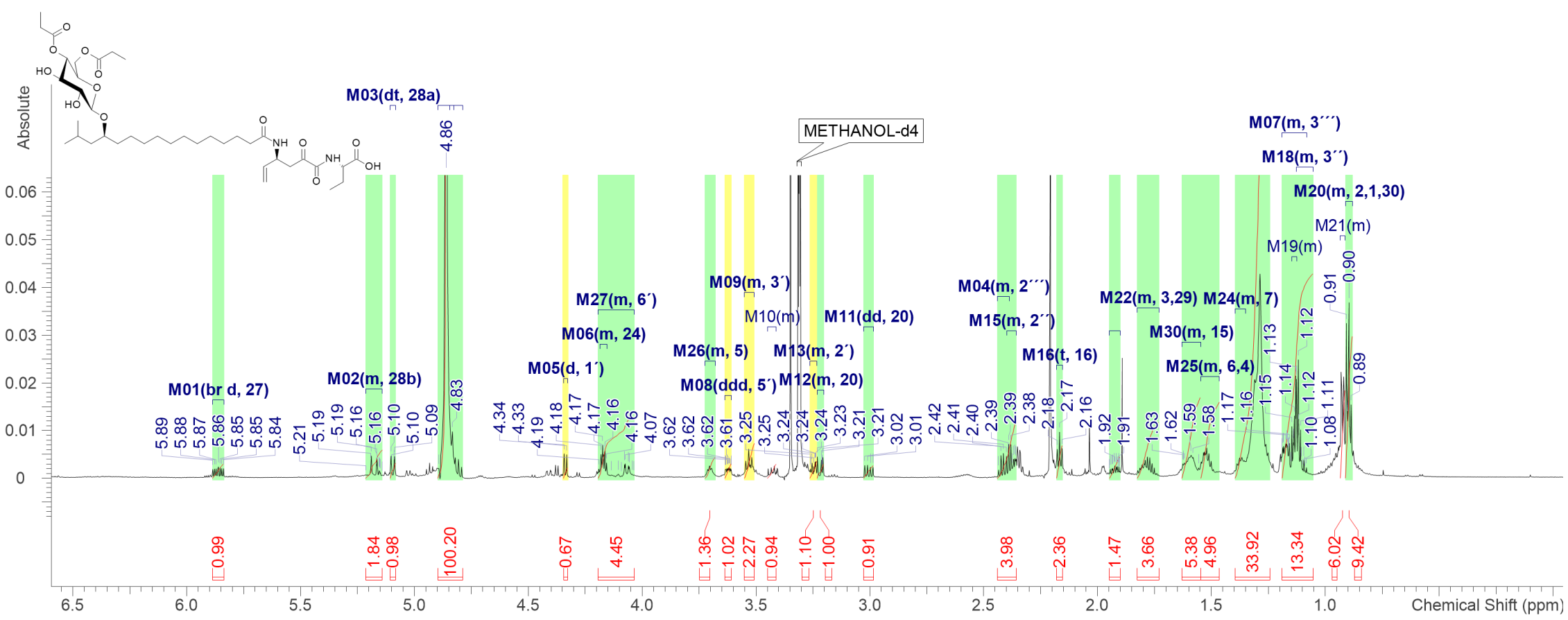

Figure S71. ^{1}H spectrum of myxoglucamide G acquired in CD_3OD at 700 MHz

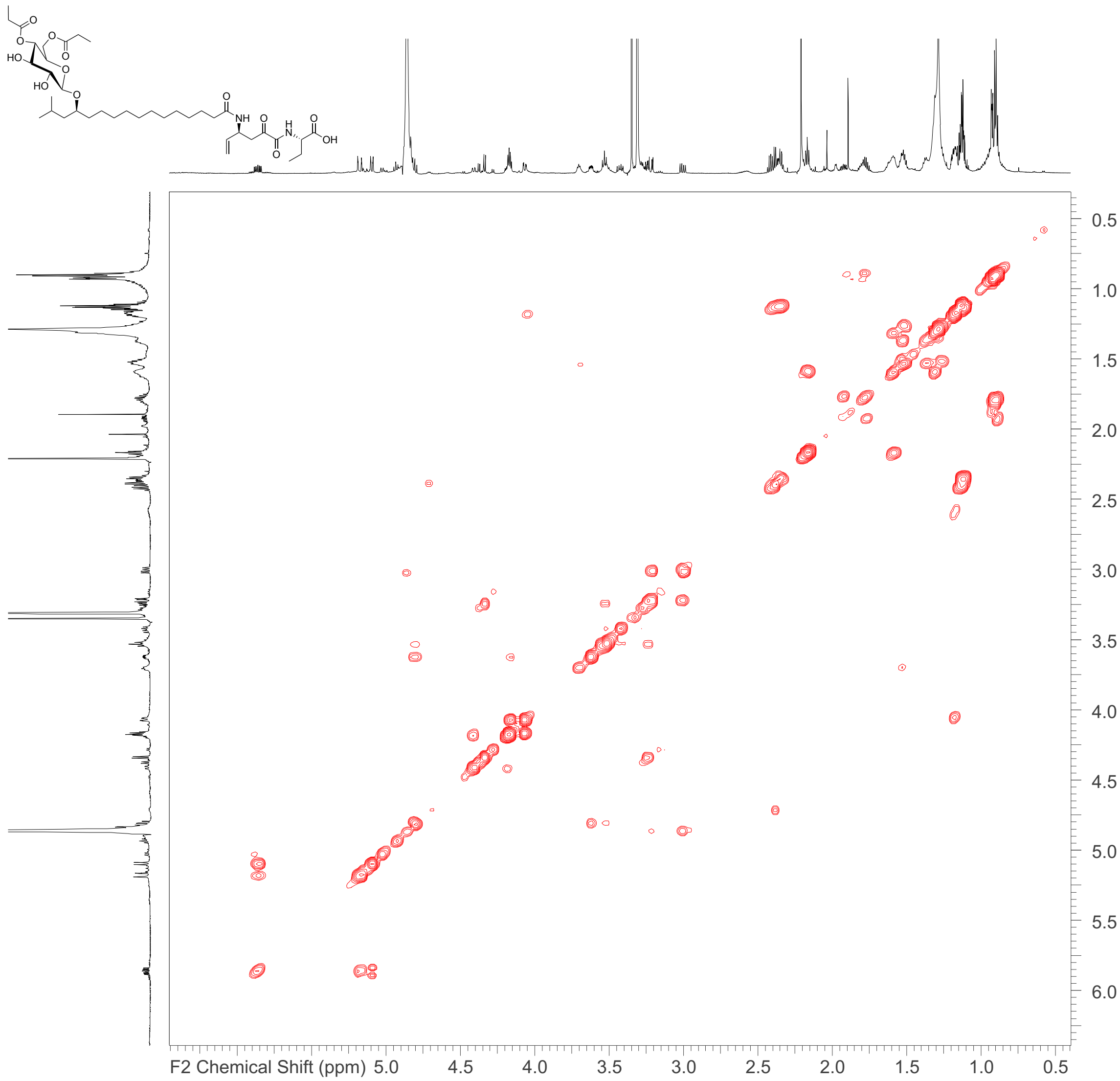

Figure S72. COSY spectrum of myxoglucamide G acquired in CD_3OD at 700 MHz

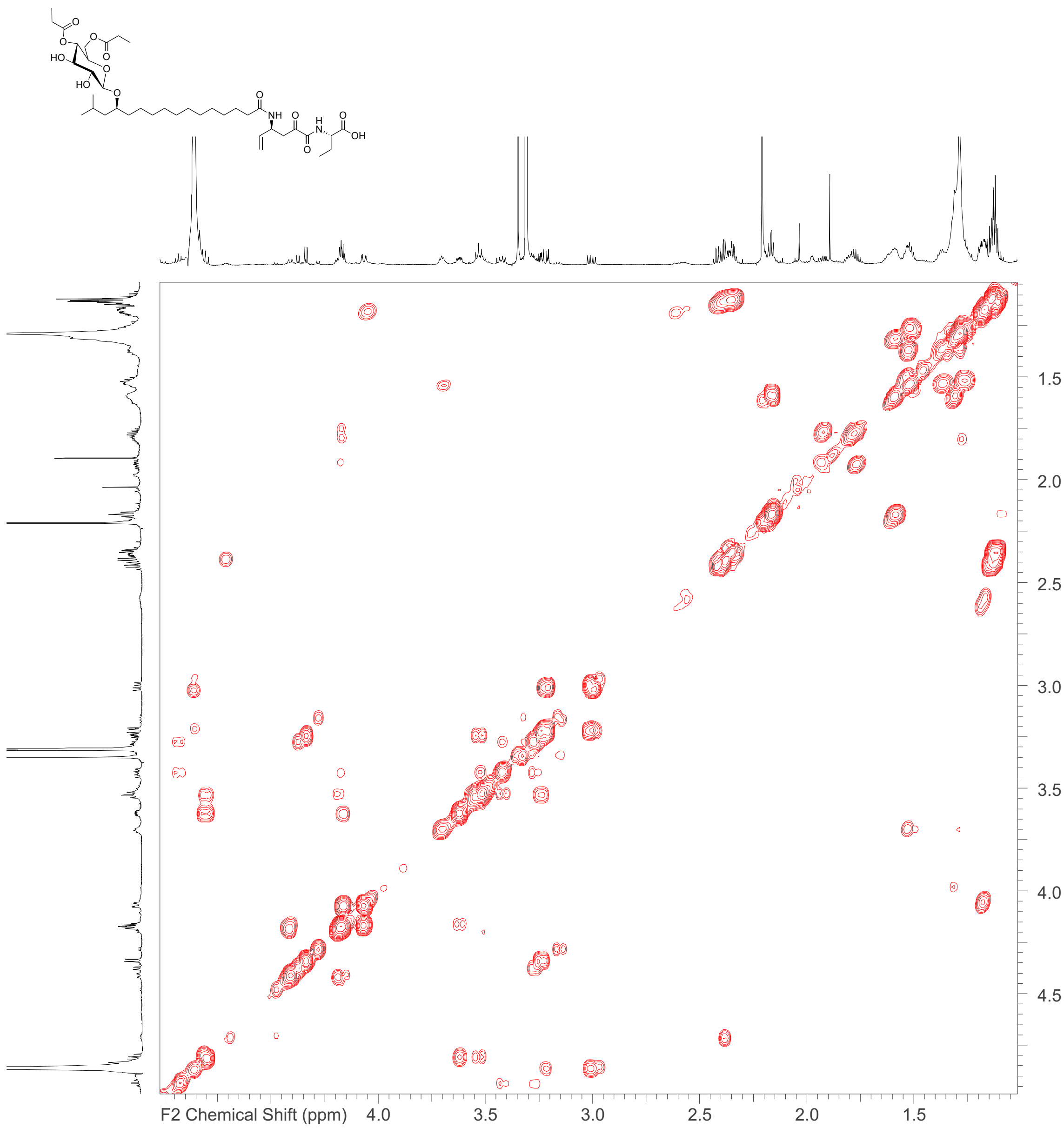

Figure S73. Enlarged COSY spectrum of myxoglucamide G acquired in CD_3OD at 700 MHz

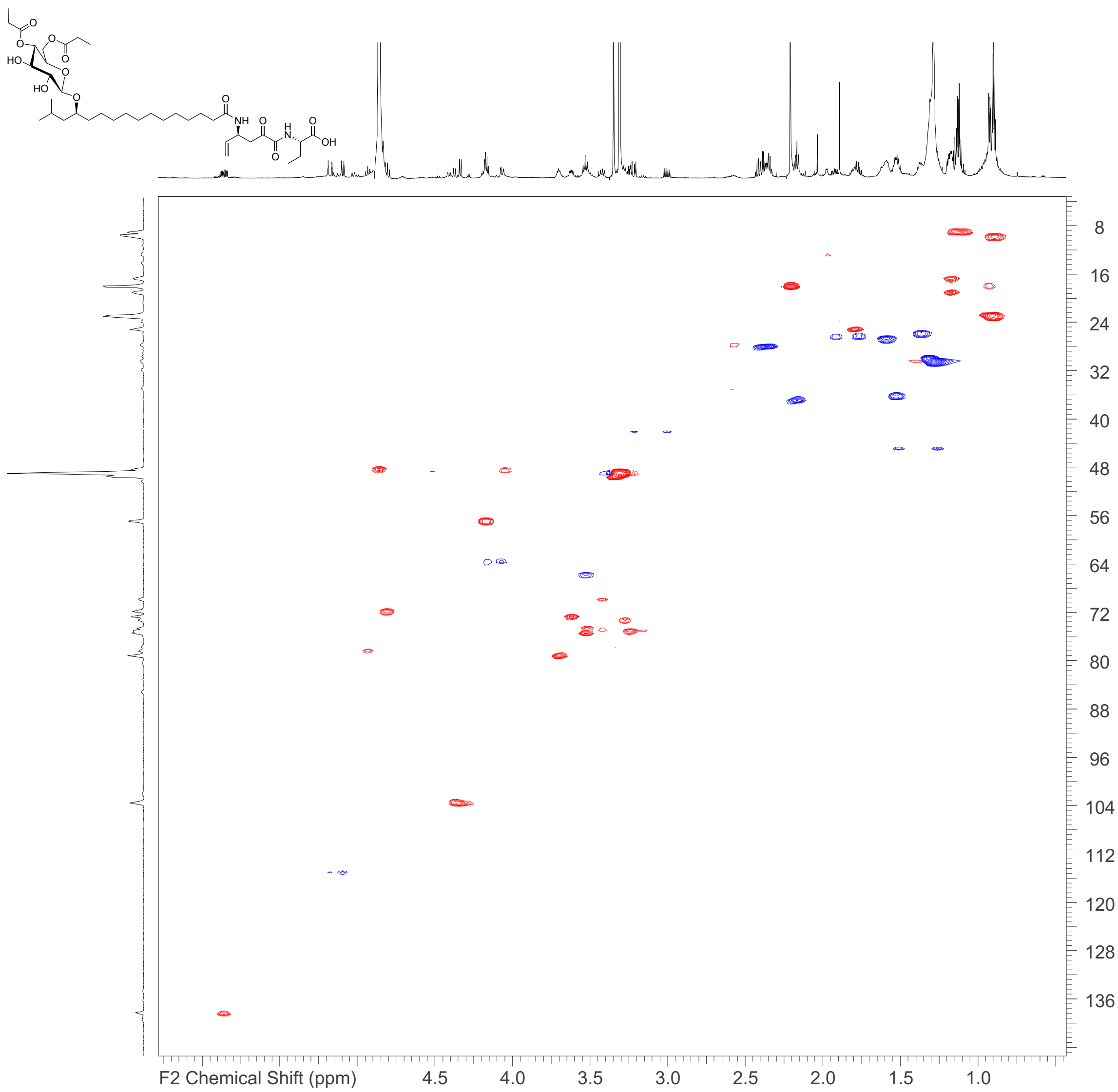

Figure S74. HSQC spectrum of myxoglucamide G acquired in CD_3OD at 700/ 175 MHz

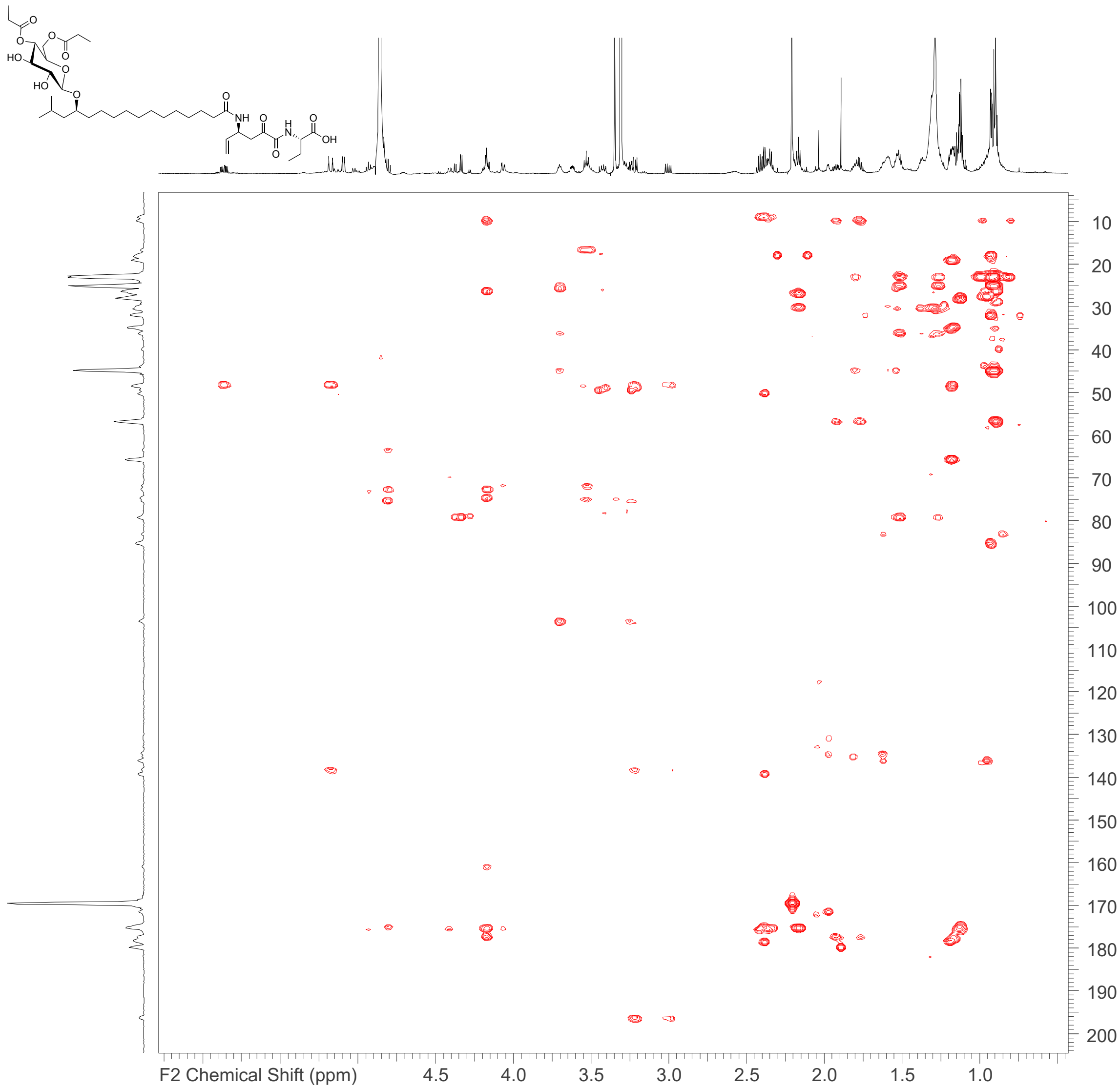

Figure S75. HMBC spectrum of myxoglucamide G acquired in CD_3OD at 700/ 175 MHz

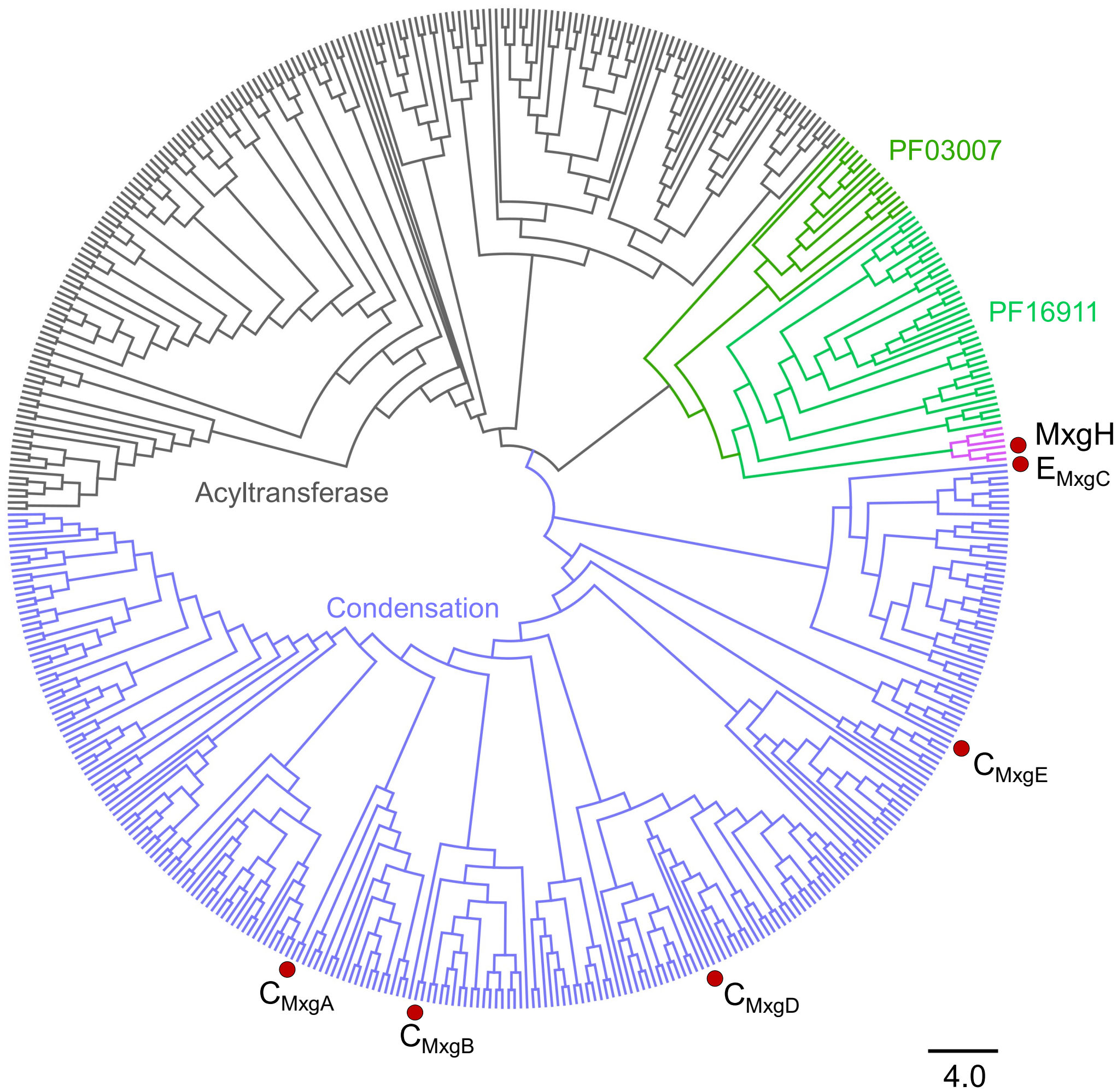

Figure S76. Phylogenetic analysis of MxgH together with representative acyltransferases from the CL0149 family

Condensation domains (PF00668) are shown in purple, acyltransferases are shown in grey, and the families that MxgH clusters with are highlighted in green (PF16911 and PF03007). MxgH and its close homologs are shown in magenta, while *mxg*-derived domains are indicated by red dots. Representative sequences were retrieved from the InterPro database by selecting members of the Pfam clan CL0149 (https://www.ebi.ac.uk/interpro/set/pfam/CL0149/) and literature[9–12,15].

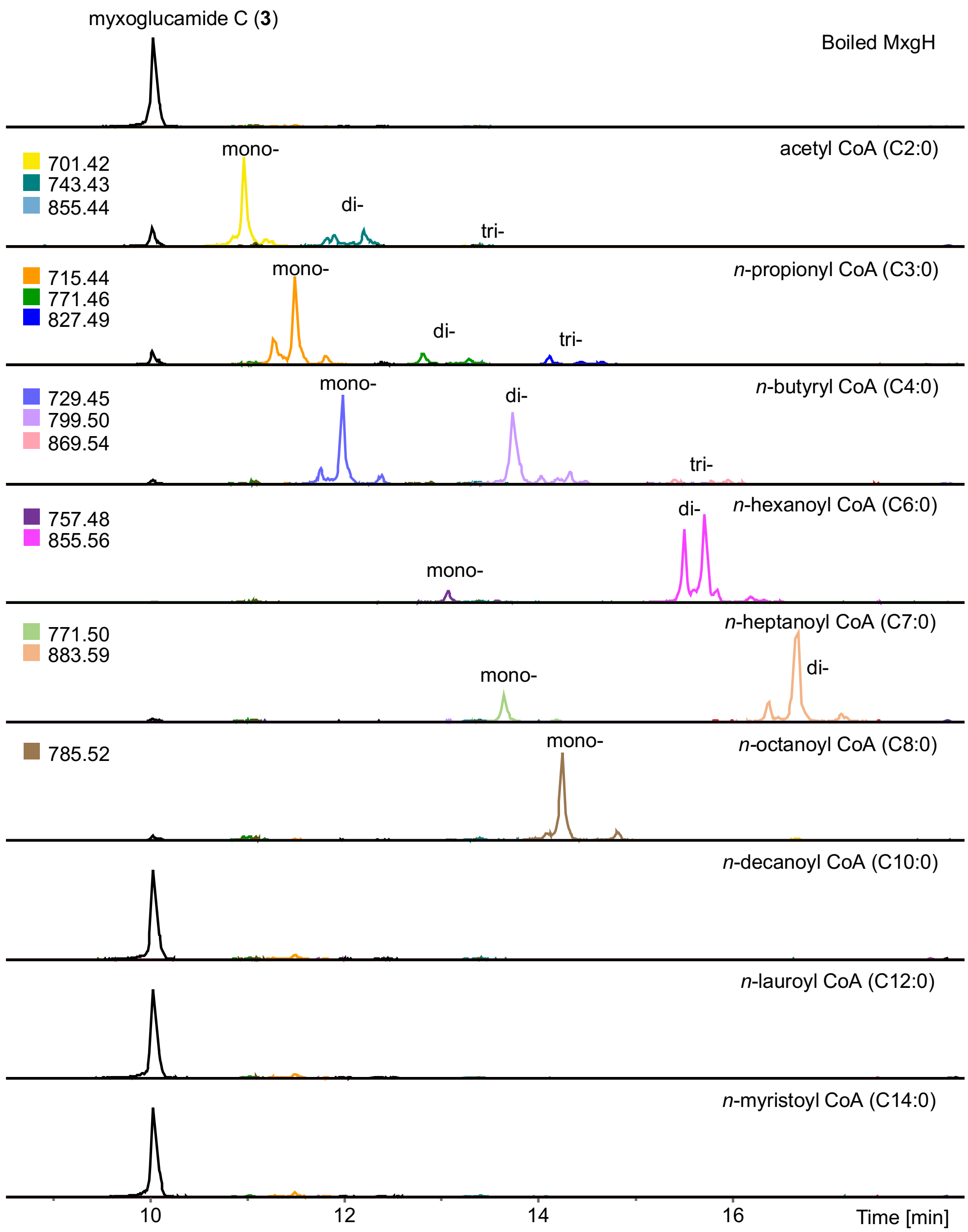

Figure S77. MxgH catalyzed *O*-acylation of myxoglucamide C with fatty acyl-CoAs of different chain lengths

In vitro O-acylation of myxoglucamide C (**3**) by recombinant MxgH using single fatty acyl-CoA substrates of varying chain lengths (C2-C14), revealing formation of mono-, di-, and/or tri-acylated products. EICs of myxoglucamide C are shown in black, whereas the reaction products are shown in different colors. Products derived from C2:0-CoA (*m/z* 701.42 and 743.43) are shown in yellow and teal, respectively; products derived from C3:0-CoA (*m/z* 715.44, 771.46, and 827.49) are shown in orange, green, and ultramarine, respectively; products derived from C4:0-CoA (*m/z* 729.45 and 799.50) are shown in blue violet and lavender, respectively; products derived from C6:0-CoA (*m/z* 757.48 and 855.56) are shown in purple and magenta, respectively; products derived from C7:0-CoA (*m/z* 771.50 and 883.59) are shown in lime and bronze, respectively; products derived from C8:0-CoA (*m/z* 785.52) are shown in tortilla**.**

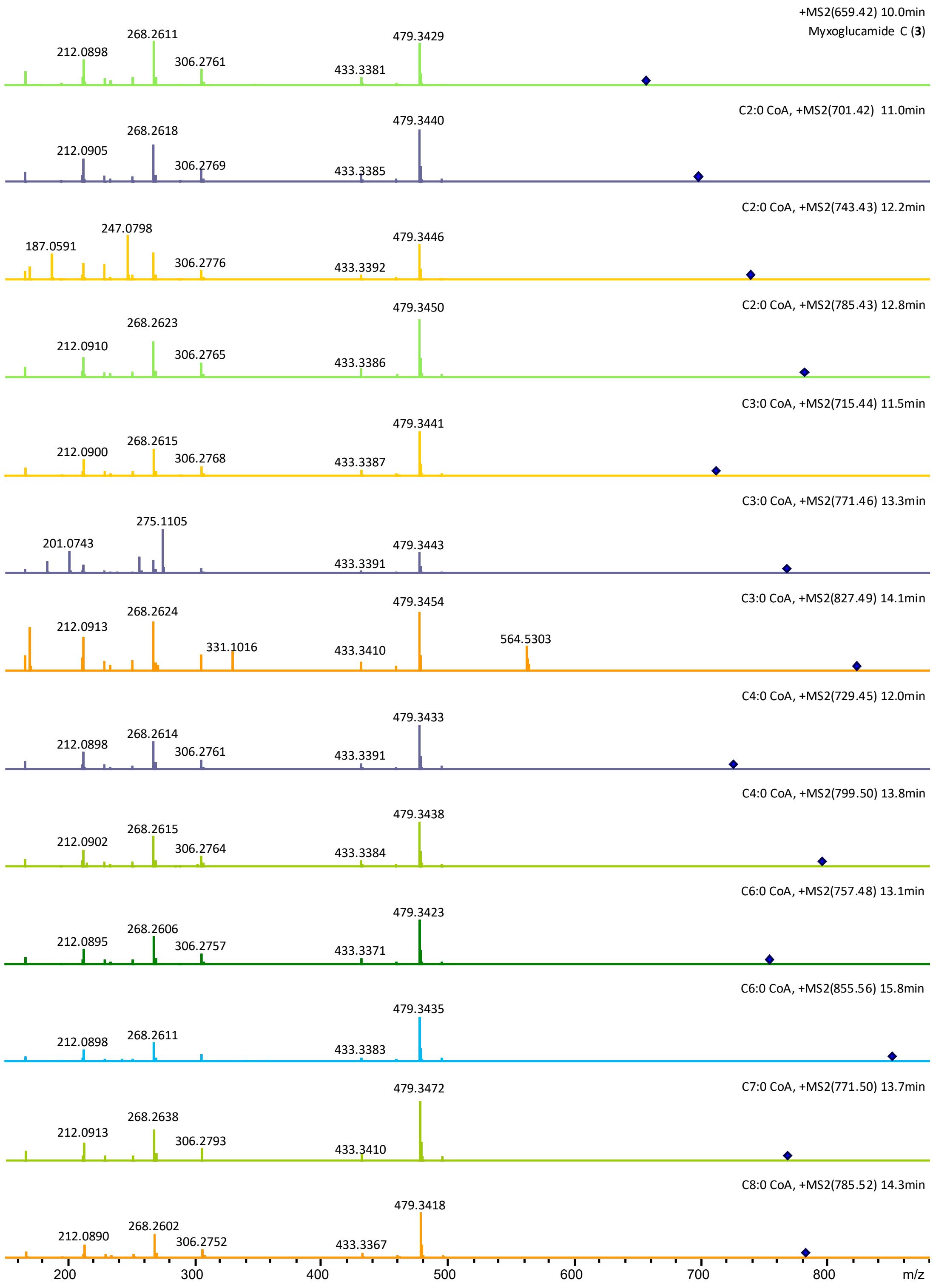

Figure S78. MS^2 fragmentation of products from MxgH-CoAs assays

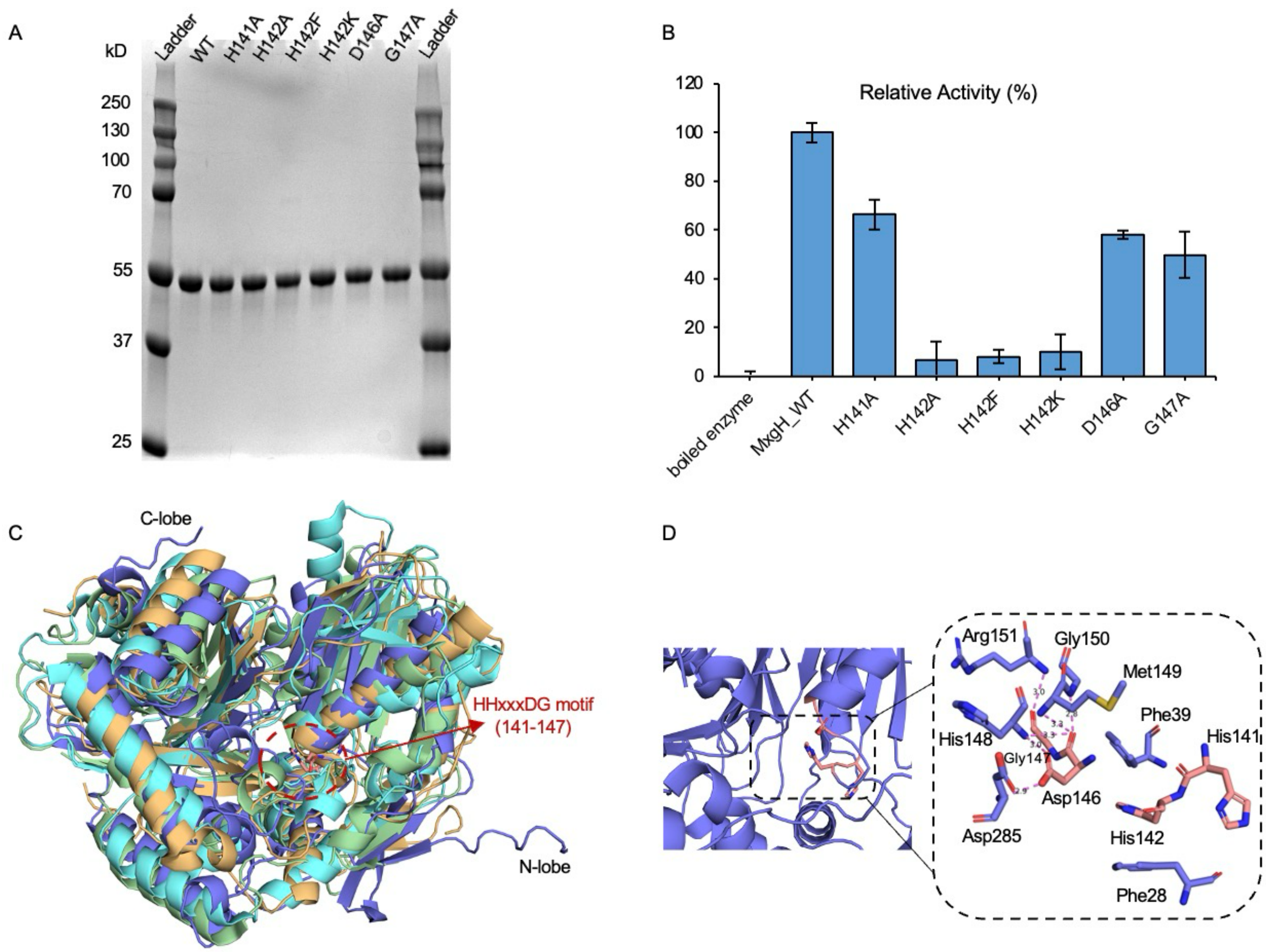

Figure S79. Mutational analysis of MxgH

A) A Bio-Rad 4-20% precast Tris-Glycine gel was loaded with each fraction. Bio-Rad precision plus protein standards were run alongside the samples for molecular weight determination, and the gel was stained with Coomassie Blue. B) Relative activities of wild-type MxgH (considered as 100% due to the complete consumption of **3**) and mutants. The experiments were repeated in duplicates three times. C) Superposition of the MxgH model (slate) predicted by AlphaFold with structural homologues identified from DALI (PDB IDs: 6n8e[16] (light orange; only C domain is shown), 4hvm (unpublished, pale green), and 4znm[10] (cyan). Cα RMSD were approx. 3.5 Å (6n8e), 4.9 Å (4hvm), and 4.5 Å (4znm) over the entire length of the protein. D) The close-up view of the MxgH active site cavity. His141 is pointing away from the active site, whereas Asp146 and Gly147 are found within H-bonding distance of Asp285, His148, Arg151, Gly150 and Met149 side chains and/or peptide backbone. Phe39 and Phe28 may help position His142 for catalysis. All distances are shown in Å.

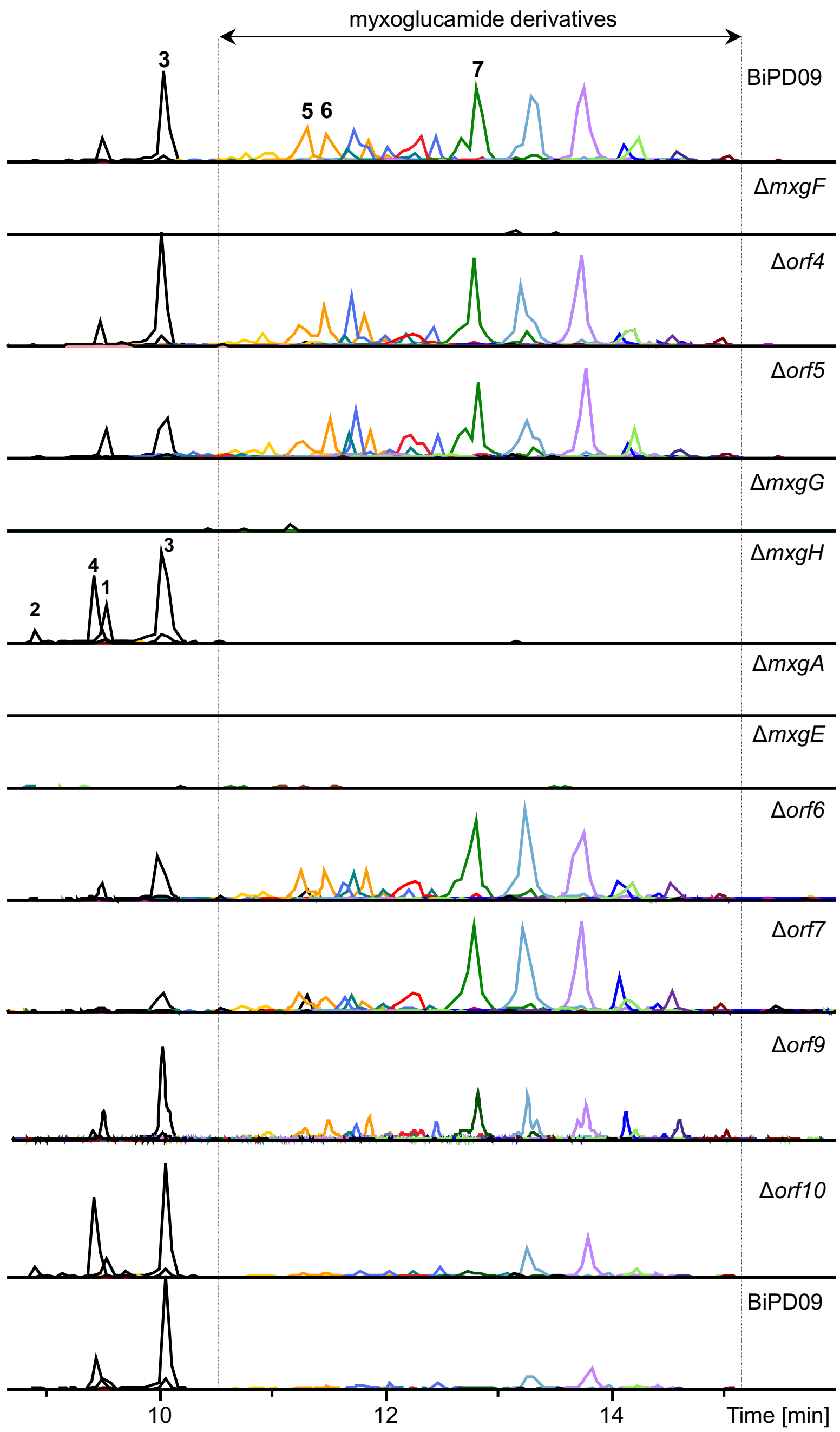

Figure S80. Gene knockouts of *mxg* in *M. xanthus* DK1622

Extracted ion chromatograms (EICs) of myxoglucamides A-D are shown in black (**1**-myxoglucamide A, **2**-myxoglucamide B, **3**-myxoglucamide C, **4**-myxoglucamide D). Myxoglucamide derivatives are shown in other colors in the range of the double-headed arrow ($[M+H]^+$ = 701.42 in yellow, $[M+H]^+$ = 715.44 in orange, $[M+H]^+$ = 729.45 in blue violet, $[M+H]^+$ = 743.43 in teal, $[M+H]^+$ = 757.45 in red, $[M+H]^+$ = 771.46 in green, $[M+H]^+$ = 785.48 in paleblue, $[M+H]^+$ = 799.50 in lavender, $[M+H]^+$ = 813.51 in lime, $[M+H]^+$ = 827.49 in ultramarine, $[M+H]^+$ = 841.51 in purple, and $[M+H]^+$ = 855.52 in brown).

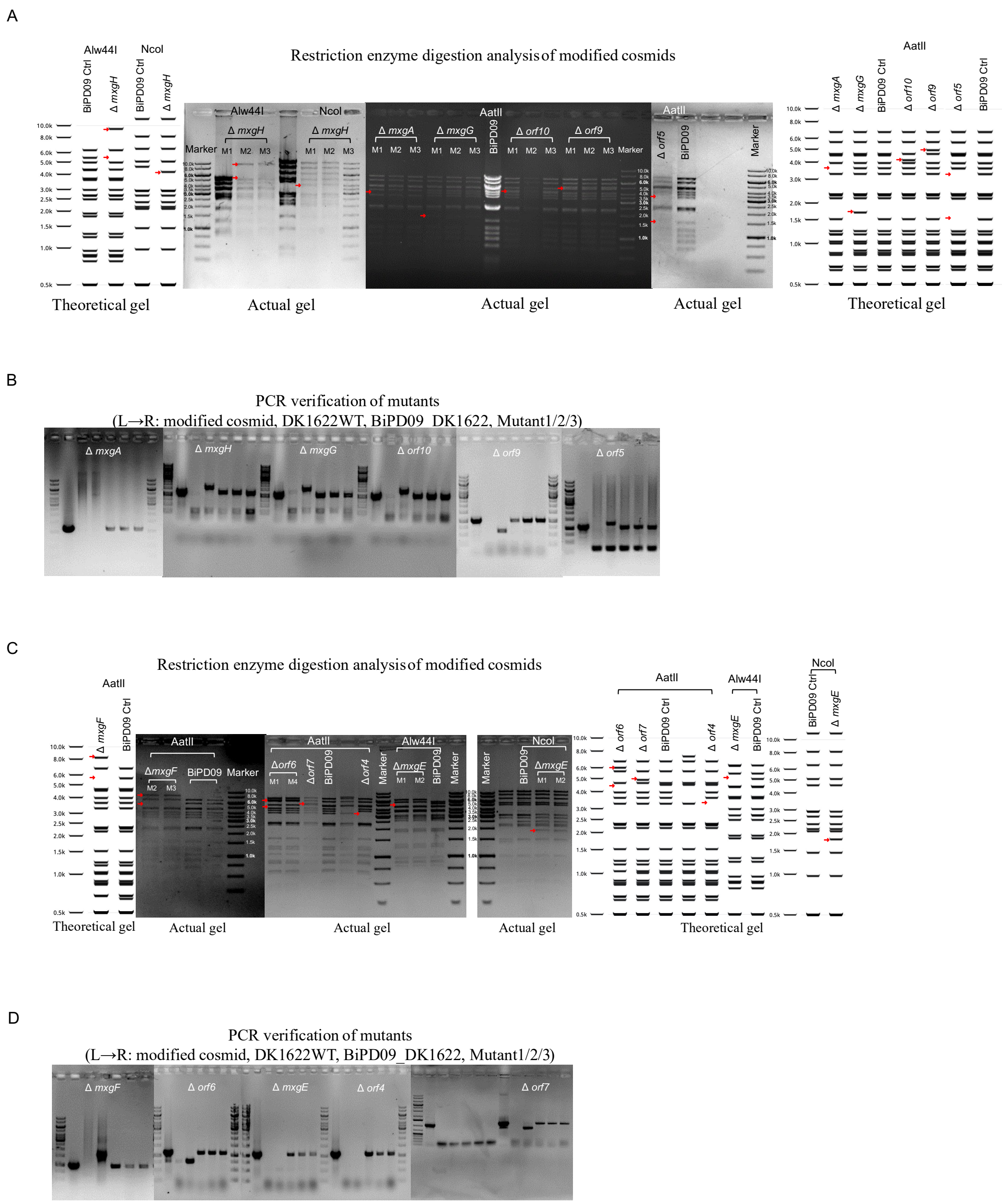

Figure S81. Verification of gene knock-out in modified cosmids and DK1622 mutants

A) Restriction enzyme digestion analysis of cosmids carrying knockouts of *mxgA*, *mxgG*, *mxgH*, *orf5*, *orf9* or *orf10*. B) PCR verification of *mxgA*, *mxgG*, *mxgH*, *orf5*, *orf9* or *orf10* knock-out DK1622 mutants. C) Restriction enzyme digestion analysis of cosmids carrying knockouts of *mxgE*, *mxgF*, *orf4*, *orf6*, or *orf7*. D) PCR verification of *mxgE*, *mxgF*, *orf4*, *orf6*, or *orf7* knock-out DK1622 mutants.

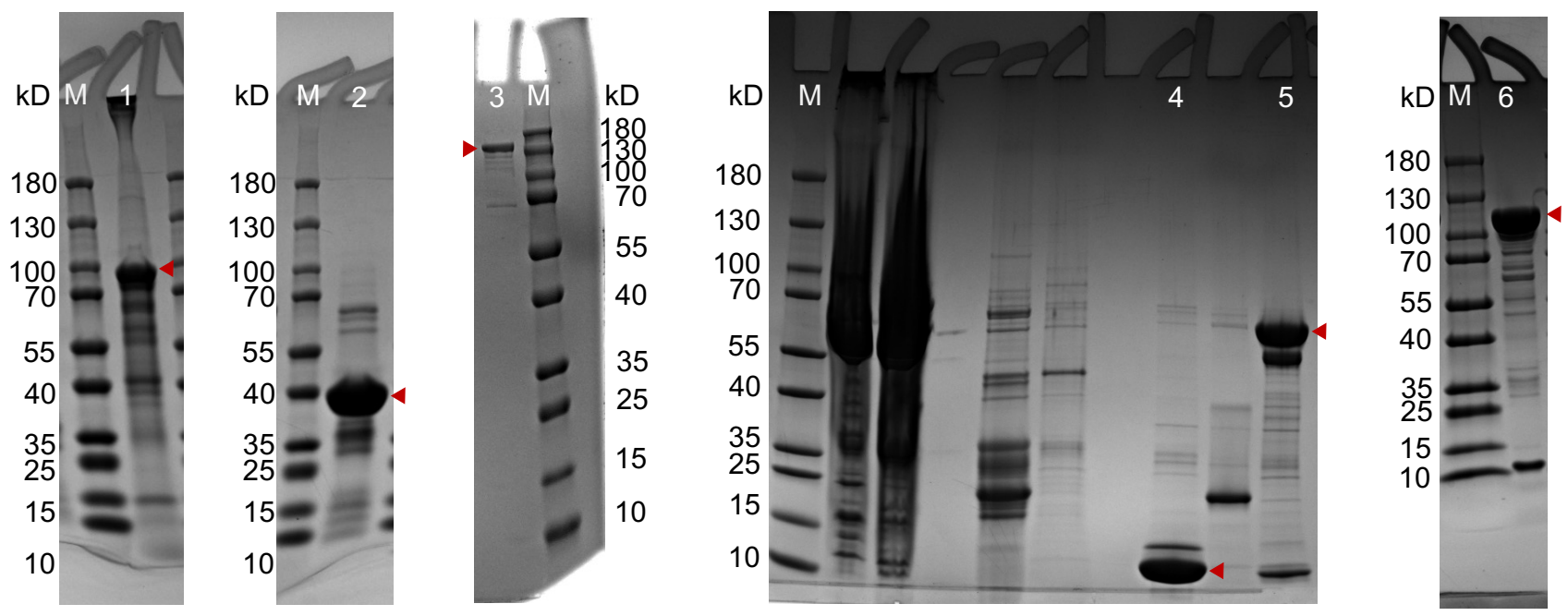

M: Protein ladder.

1: FAAL-ACP$_{MxgA}$ (His tag), 77.0 kD

2: Ox$_{MxgA}$ (His tag), 39.7 kD

3: MxgA (His tag), 167.1 kD

4: PCP$_{MxgB}$ (His tag), 12.2 kD

5: A$_{MxgB}$ (His tag), 63.8 kD

6: MxgB (His tag), 120.4 kD

Figure S82. SDS-PAGE analysis of proteins used in **Figure 4**

M, protein ladder. Lanes 1-6 correspond to FAAL-ACP$_{MxgA}$, Ox$_{MxgA}$, MxgA, PCP$_{MxgB}$, A$_{MxgB}$, and MxgB, respectively. The calculated molecular weights are indicated. The corresponding protein bands are marked with red arrowheads.

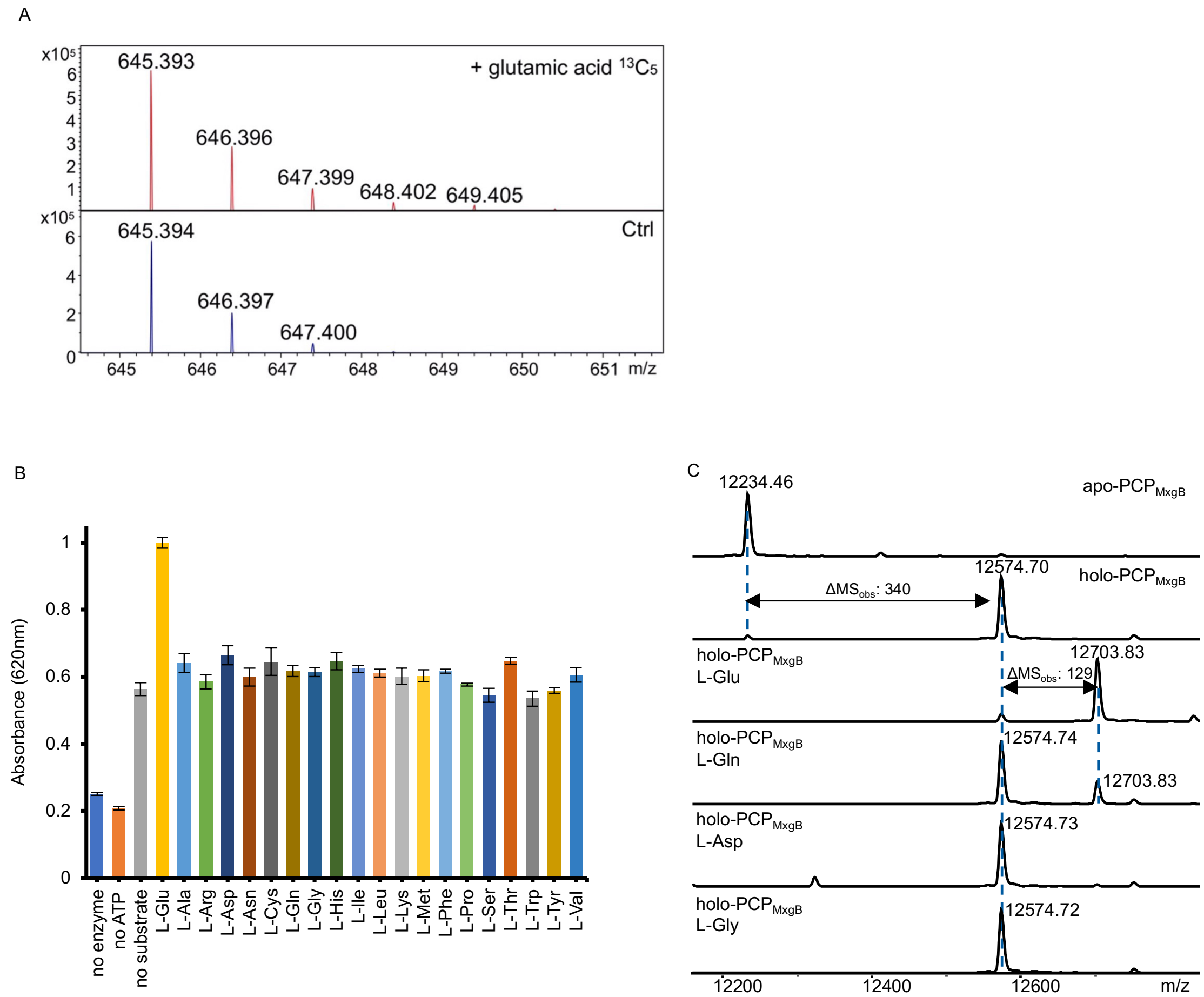

Figure S83. Incorporation of glutamate and substrate specificity of A_{mxgB} for 20 amino acids

A) Isotope pattern of myxoglucamide A showing single charged ion $[M+H]^+$ extracted from UPLC-HRMS chromatograms from a control culture (blue) and supplemented with $^{13}C_5$ glutamic acid (red). B) Substrate specificity of A_{MxgB} was assessed using a malachite green based phosphate detection assay. Individual substrate activities are presented by bars and the highest activity defined as 1. Data are shown as mean ± SD from three independent experiments. C) Amino acid loading onto PCP_{MxgB} by A_{mxgB} was further validated by intact protein mass spectrometry.

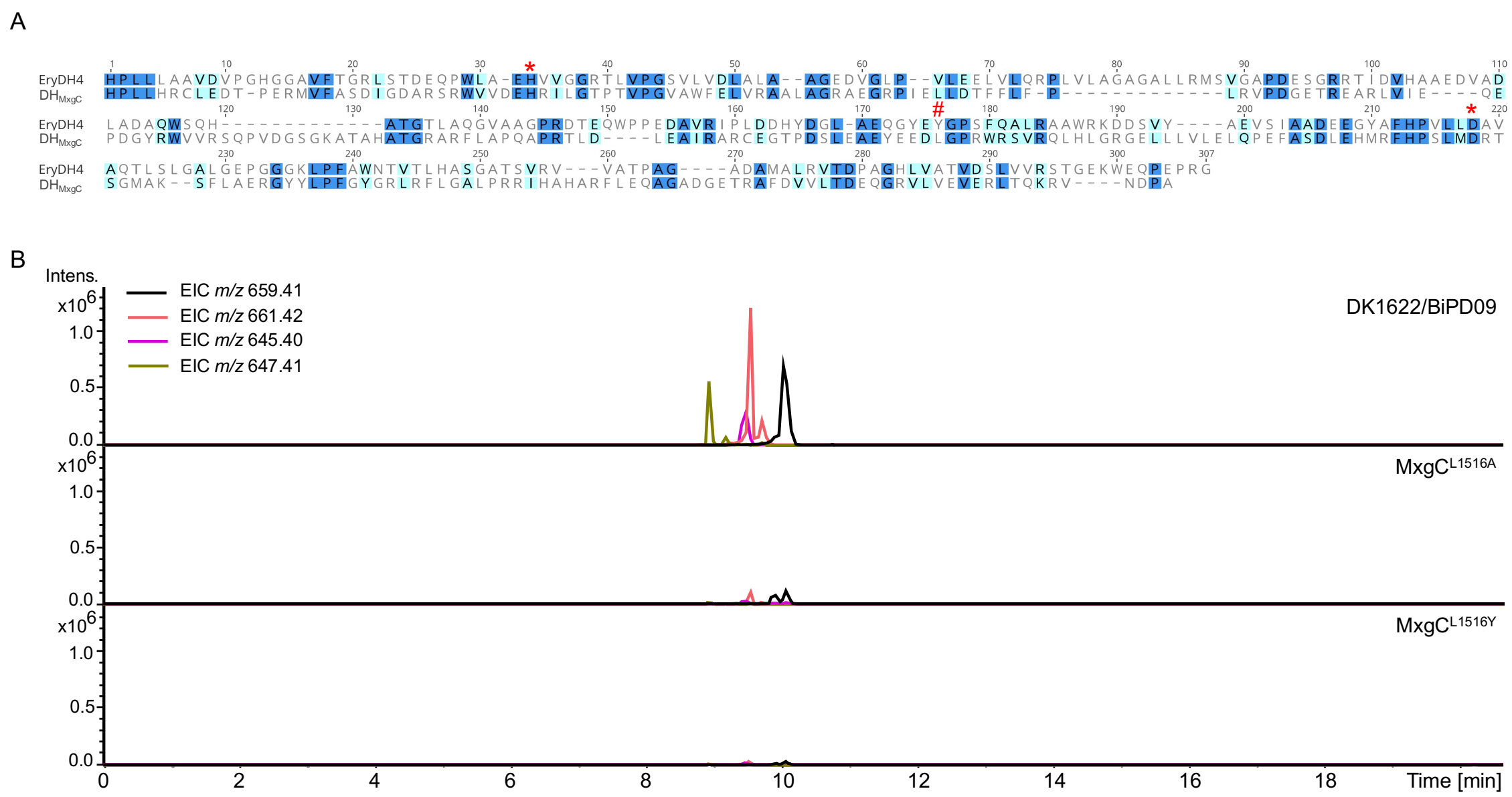

Figure S84. Sequence alignment and functional analysis of catalytic residues in DH_{MxgC}

A) Sequence alignment of DH_{MxgC} with EryDH4, a cis-AT dehydratase from the erythromycin biosynthetic gene cluster[17]. Conserved catalytic residues are indicated by red asterisks. The tyrosine highlighted with a red hashtag corresponds to the conserved "YGP" motif, typically Tyr (or occasionally Phe), which has been proposed to facilitate protonation of the β-hydroxyl group. Notably, this position is occupied by leucine in DH_{MxgC}.

B) Site-directed mutagenesis of this leucine to alanine or tyrosine demonstrated its critical role in DH_{MxgC} activity and myxoglucamide biosynthesis.

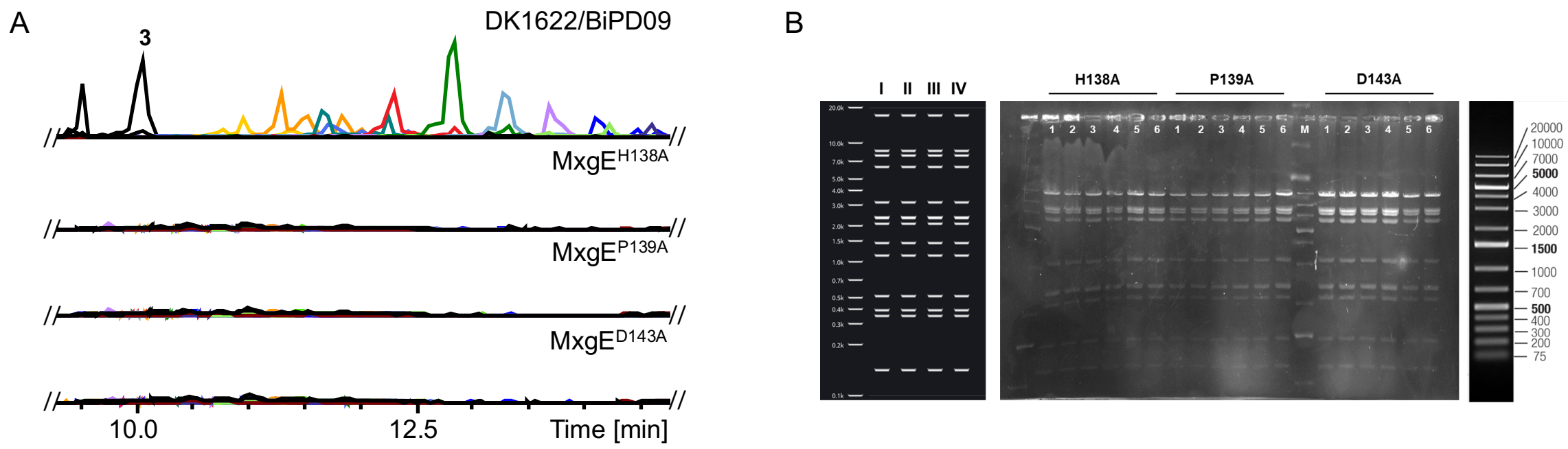

Figure S85. Site-directed mutagenesis of C_{MxgE}

A) Site-directed mutagenesis of C_{MxgE} abolished the production of all myxoglucamides. B) Agarose gel electrophoresis of C_{MxgE} mutant plasmids digested with the restriction enzyme *Sgs*I. The left panel shows the simulated digestion patterns of the corresponding plasmids. Lane I: BiPD09-WT plasmid, Lane II: MxgE^{H138A}; Lane III: MxgE^{P139A}; Lane IV: MxgE^{D143A}.

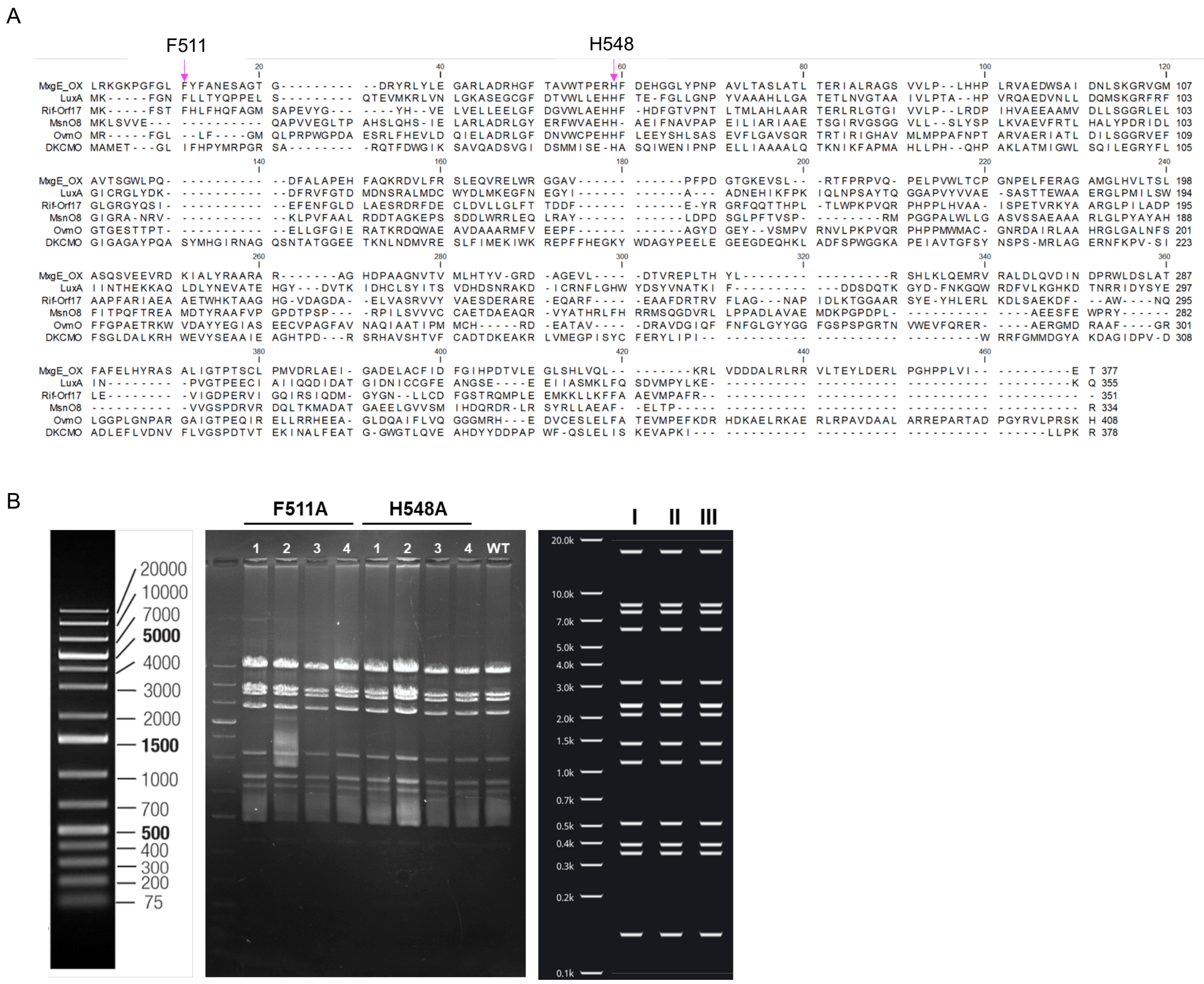

Figure S86. Sequence alignment of Ox_{MxgE} with other luciferase-like monooxygenase class flavin-dependent oxidoreductases and gel verification of Ox_{MxgE} mutants

A) Sequence alignment of Ox_{MxgE} with other luciferase-like monooxygenase class flavin-dependent oxidoreductases including LuxA (GenBank: UXP07237.1), Rif-Orf17 (GenBank: KDO09628.1), MsnO8 (GenBank: AHL46694.1), OvmO (WP_194274743.1), DKCMO (GenBank: AEZ35247.1). Two amino acids F511 and H548 in MxgE are important residues for cofactor FMN binding. B) Agarose gel electrophoresis of Ox_{MxgE} domain mutant plasmids (F511A and H548A) digested with the restriction enzyme *Sgs*I. The right panel shows the simulated agarose gel electrophoresis patterns of corresponding *Sgs*I-digested plasmids. Lane I: BiPD09-WT plasmid, Lane II: $MxgE^{F511A}$ plasmid; Lane III: $MxgE^{H548A}$ plasmid.

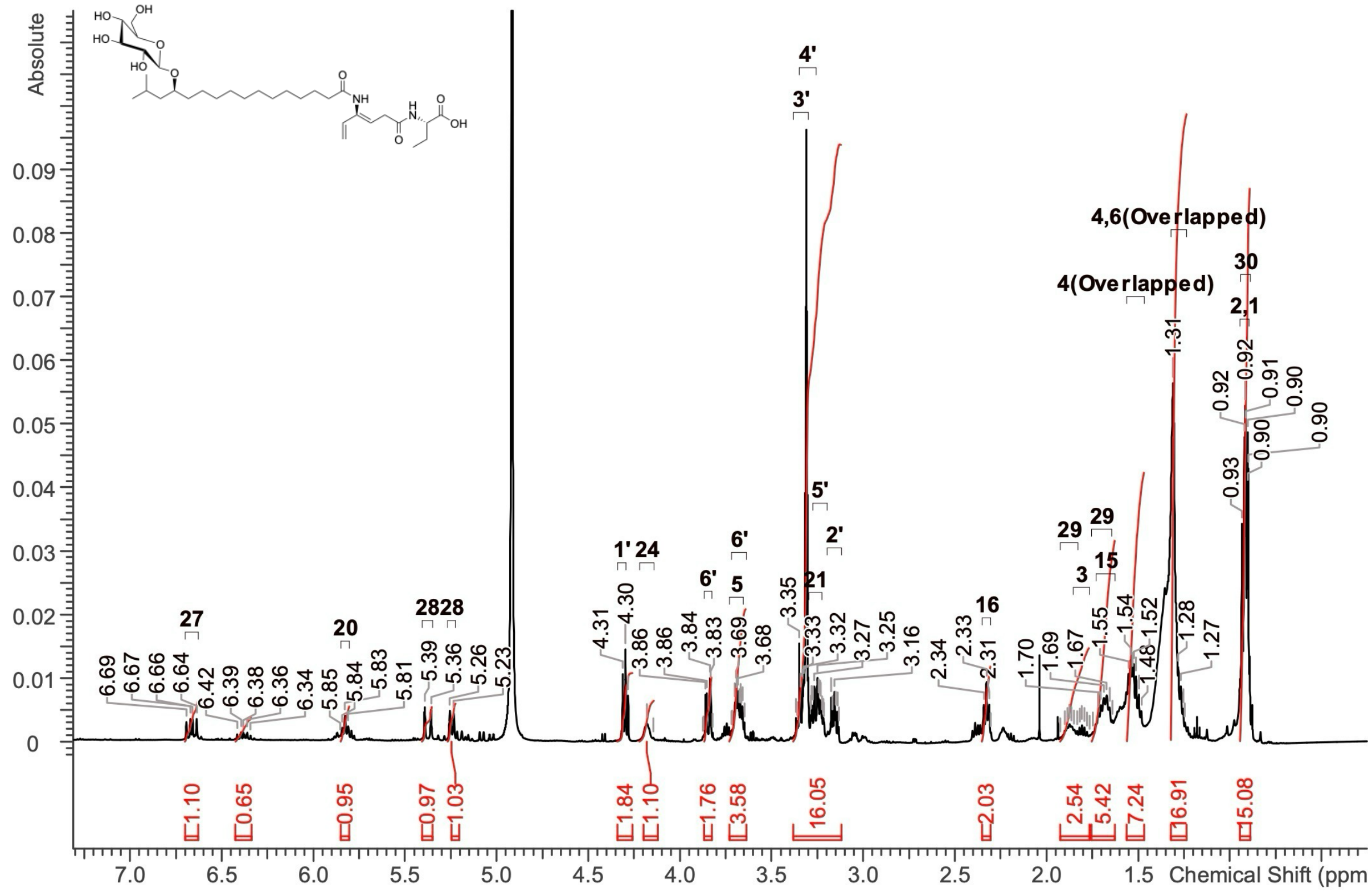

Figure S87. ^{1}H spectrum of intermediate **8** acquired in CD_3OD at 700 MHz

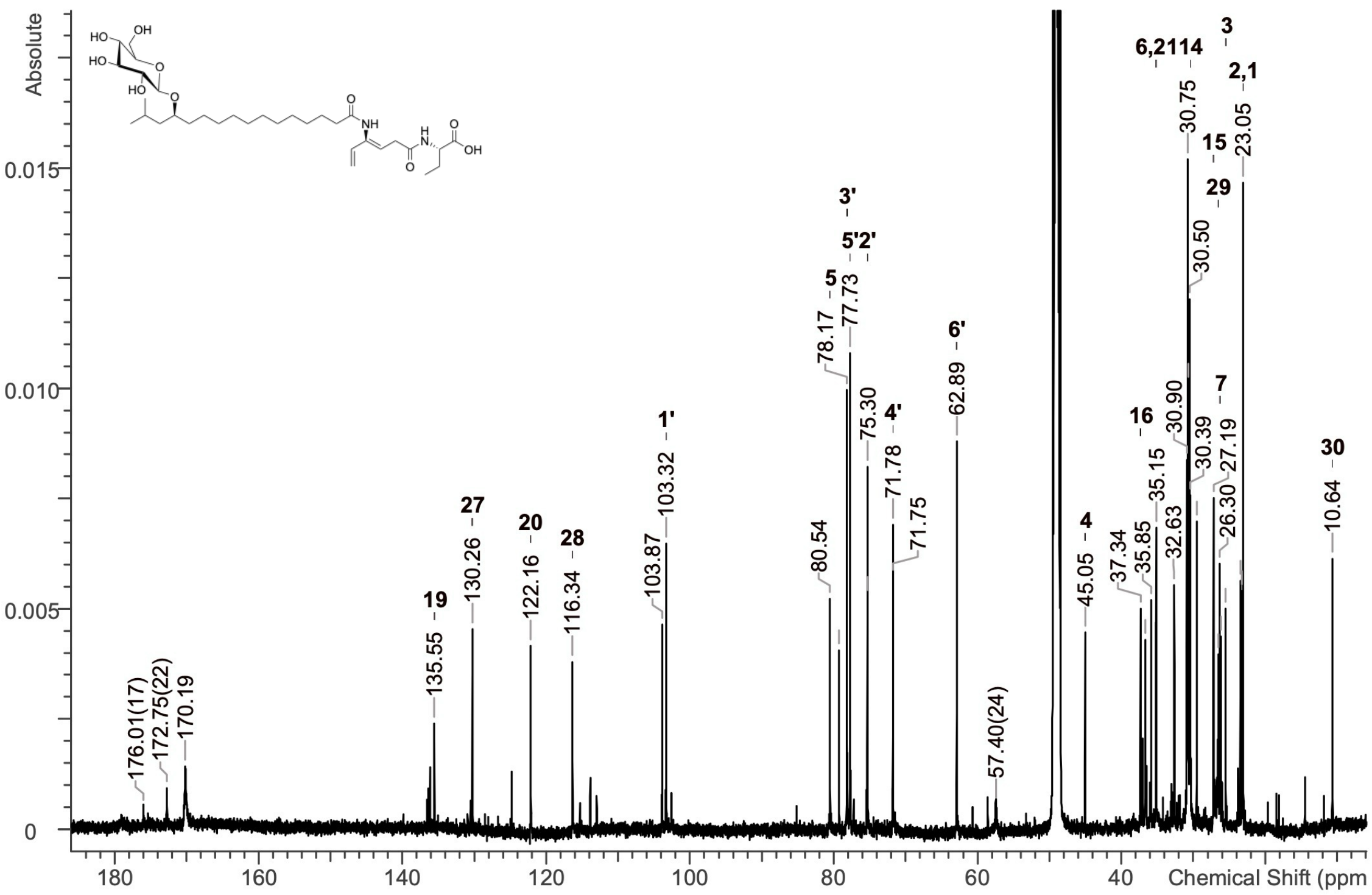

Figure S88. ^{13}C spectrum of intermediate **8** acquired in CD_3OD at 175 MHz

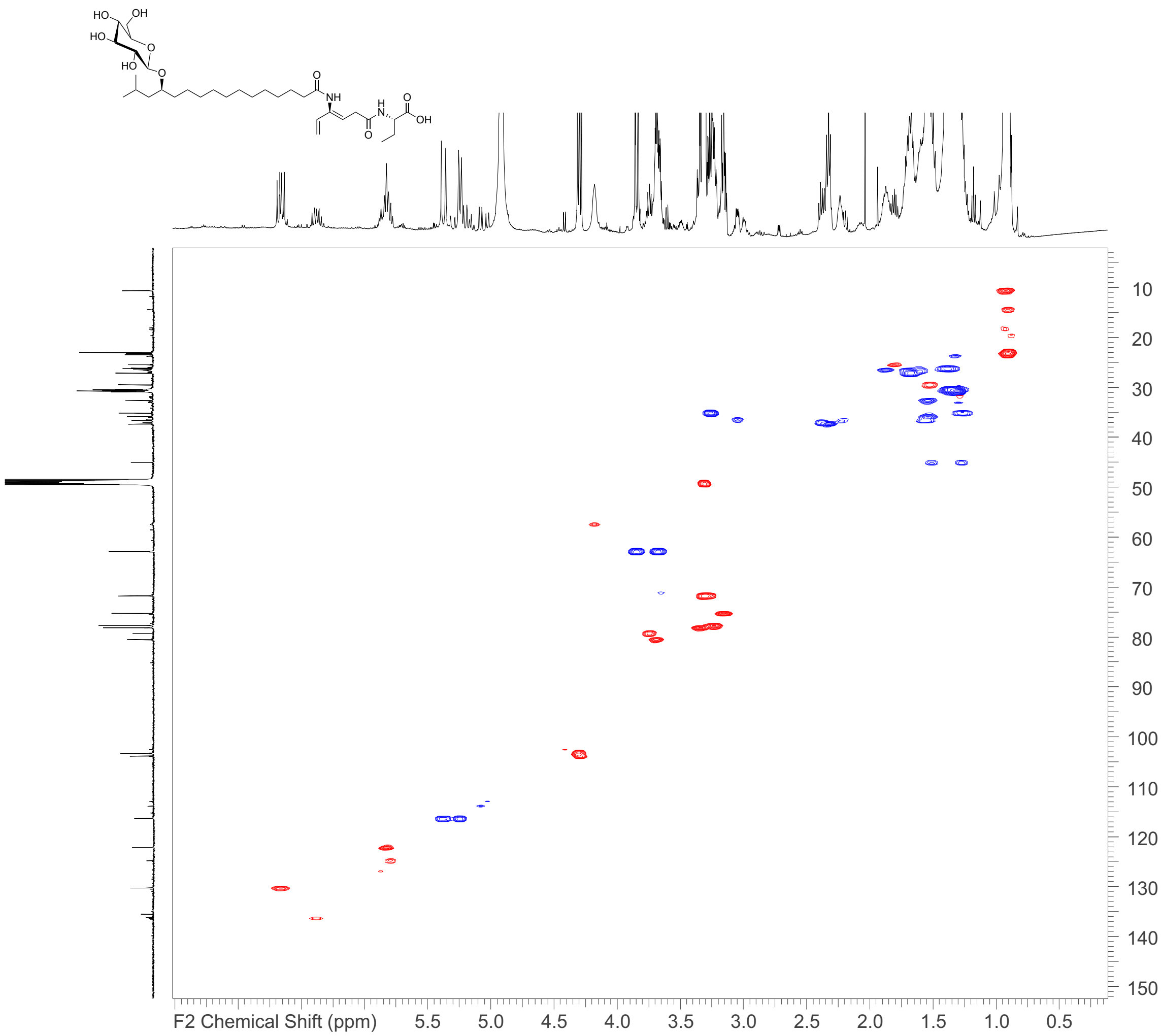

Figure S89. HSQC spectrum of intermediate **8** acquired in CD_3OD at 700/175 MHz

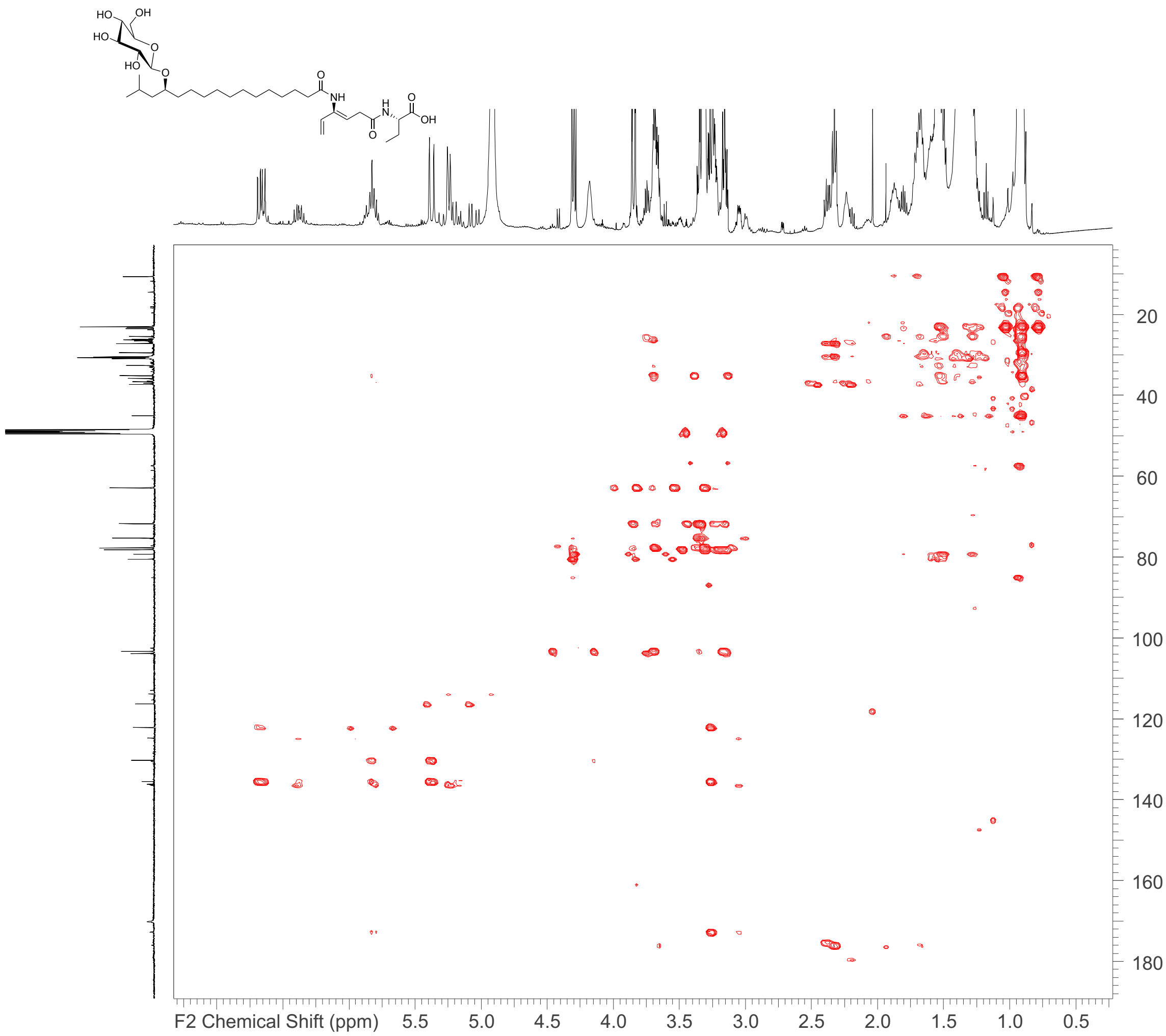

Figure S90. HMBC spectrum of intermediate **8** acquired in CD_3OD at 700/175 MHz

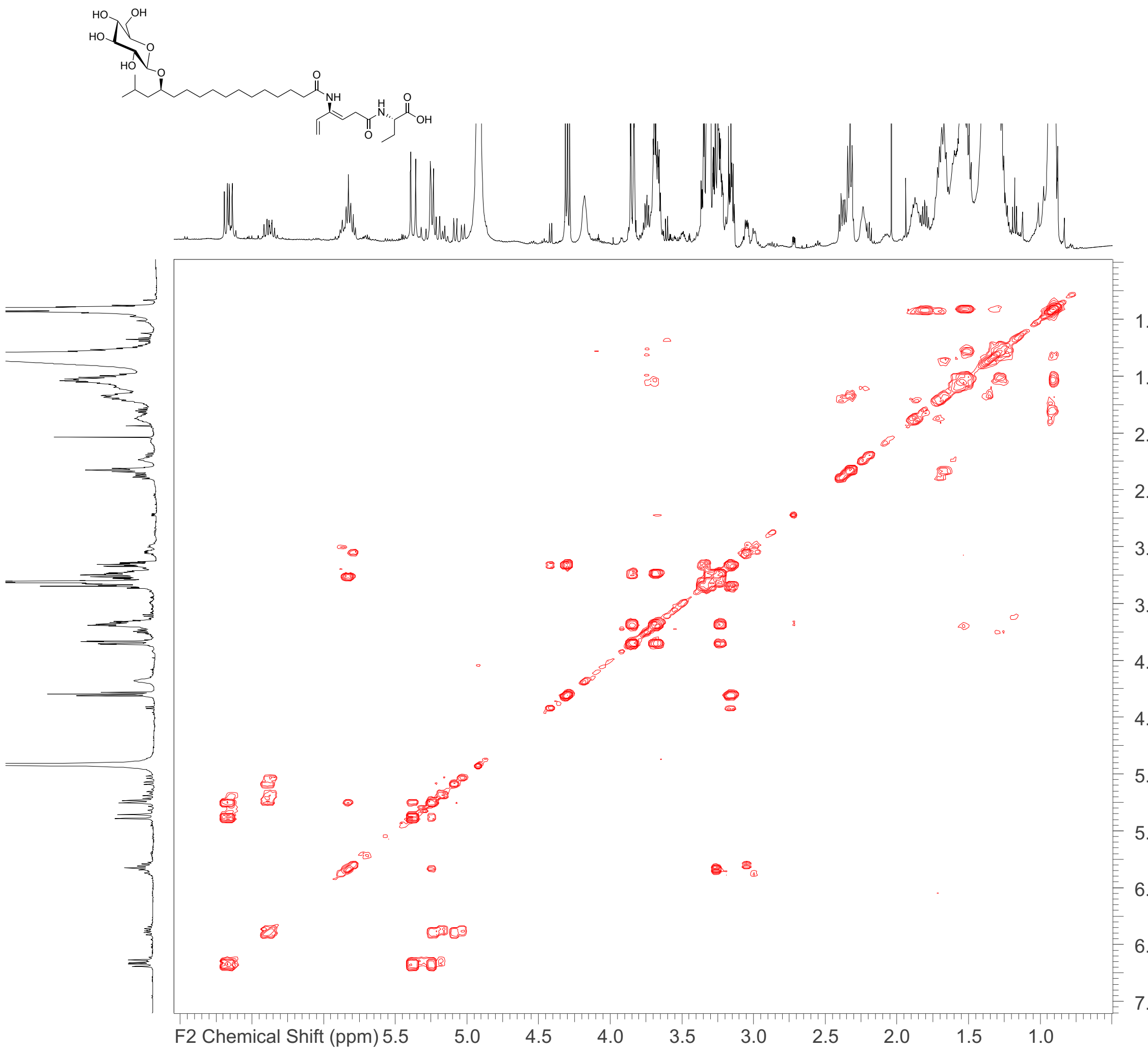

Figure S91. COSY spectrum of intermediate **8** acquired in CD_3OD at 700 MHz

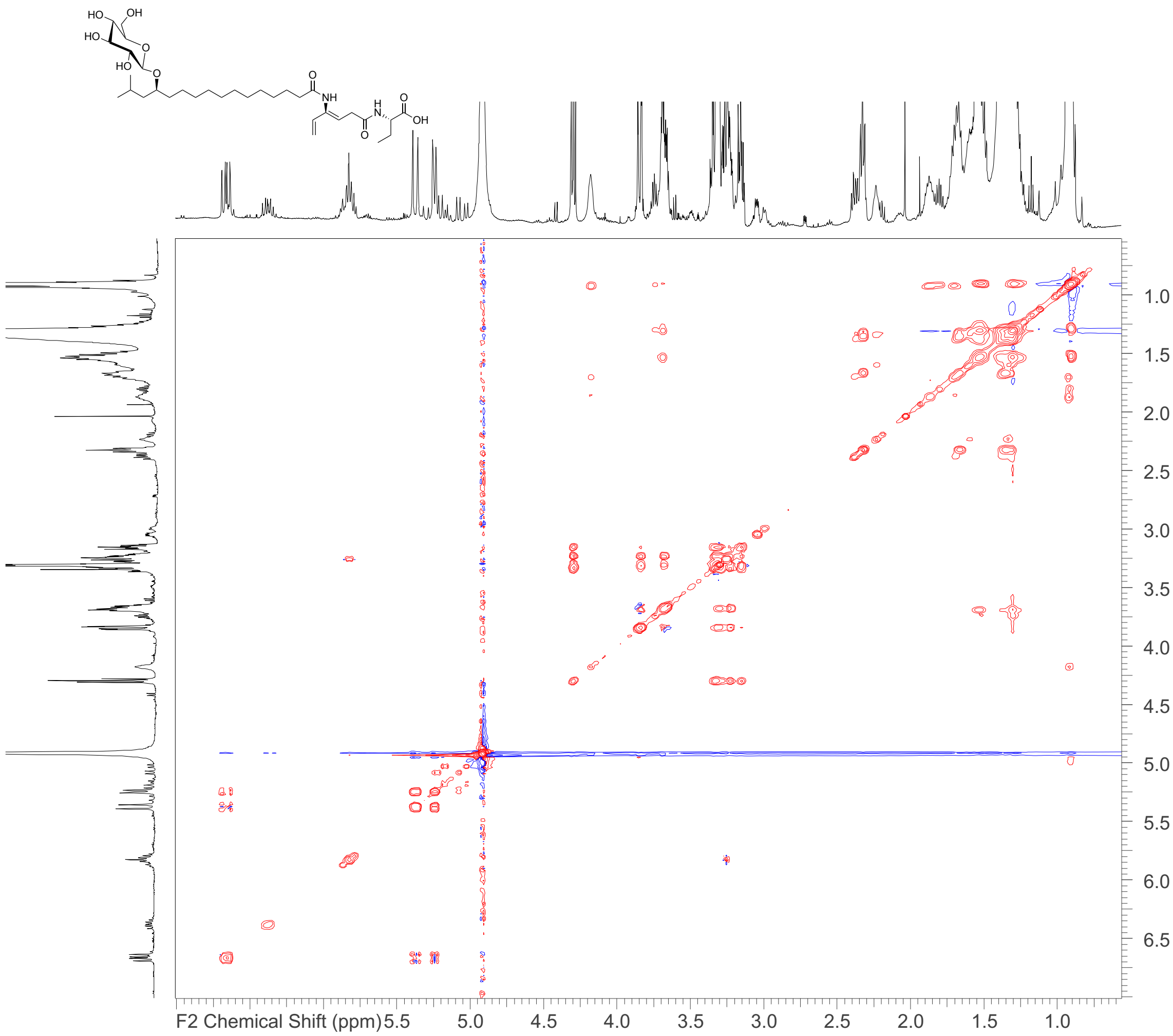

Figure S92. TOCSY spectrum of intermediate **8** acquired in CD_3OD at 700 MHz

Supporting Tables

Table S1. Gene annotation of open reading frames from *mxg* gene cluster

Gene	Size[a]	Proposed function	Highest similarity in PDB (organism)	PDB hit accession number (identity/similarity %)[b]	Catalytic domains
orf1	393	Efflux pump protein	Probable Resistance-Nodulation-Cell Division (RND) efflux membrane fusion protein (*Pseudomonas aeruginosa*)	6VEJ_P (29/44%)	
orf2	1048	Efflux pump protein	Efflux pump membrane transporter (*Klebsiella pneumoniae subsp. Pneumoniae*)	8P1I_A (34/53%)	
mxgF	420	Glycosyltransferase	Sterol 3-beta-glucosyltransferase (*Saccharomyces cerevisiae* ATCC 204508)	5XVM_A (34/51%)	GT
orf3	492	Hypothetical protein	NA	NA	
orf4	745	Hypothetical protein	Prolyl Endopeptidase (*Haliotis discus hannai*)	6JCI_A (48/67%)	
orf5	368	Aldo-keto reductase	Chain A, DepB (*Devosia*)	8HWN_A (40/54%)	
mxgG	464	Cytochrome P450	Cytochrome P450 (*Streptomyces avermitilis*)	7WZL_A (38/53%)	Ox
mxgH	455	Dimycocerosyl transferase	Phthiocerol/phthiodiolone dimycocerosyl transferase (*Pseudomonas aeruginosa* PAO1)	8S6L_A (29/39%)	acyltransferase
mxgA	1509	NRPS	Polyketide synthase (*Synechococcus sp*. PCC 7002)	7R7G_A (50/66%)	FAAL; ACP; $^{L}C_{L}$; Ox
mxgB	1082	NRPS	Microcystin synthetase B (Fragment) (*Microcystis aeruginosa*)	8HLK_A (33/51%)	$^{L}C_{L}$; A; PCP
mxgC	2242	PKS-NRPS	Polyketide synthase (*Escherichia coli*)	8XJY_A (49/64%)	KS; AT; KR; DH; ACP; E

mxgD	1301	NRPS	surfactin A synthetase C (SrfA-C) (*Bacillus subtilis*)	2VSQ_A (34/52%)	COM; $^{D}C_{L}$; A; PCP; TE
mxgE	877	NRPS	PuwE Flavin-utilizing Monooxygenase (Ox) Domain of Hybrid PKS/NRPS (*Cylindrospermum alatosporum* CCALA 988)	6KET_A (48/69%)	$^{D}C_{L}$; Ox
orf6	235	Hypothetical protein	PHB depolymerase PhaZ7 (*Paucimonas lemoignei*)	4BYM_A (24/43 %)	
orf7	209	Hypothetical protein	Peroxisomal coenzyme A diphosphatase NUDT7 (*Homo sapiens*)	5QGG_A (34/53 %)	
orf8	297	Hypothetical protein	Undecaprenyl-diphosphatase (*Escherichia coli*)	5OON_A (36/53 %)	
orf9	156	Hypothetical protein	UPF0225 protein YchJ (*Salmonella enterica subsp. enterica serovar Typhimurium* str. 14028S)	9VXG_A (47/67 %)	
orf10	415	Glycosyltransferase	Trehalose phosphorylase/synthase (*Thermoproteus uzoniensis*)	6ZJ4_AAA (46/65 %)	GT

[a]Sizes are given in amino acids; [b]Accession numbers and percentage of identity/similarity are given in parentheses. The proposed function and closest homolog according to a blastp search in PDB (Protein Data Bank) protein database of NCBI.

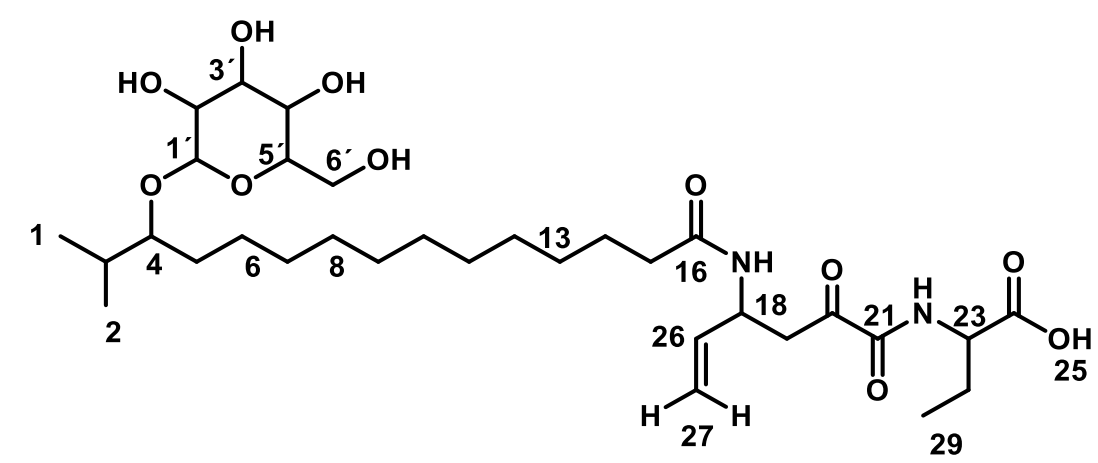

Myxoglucamide A

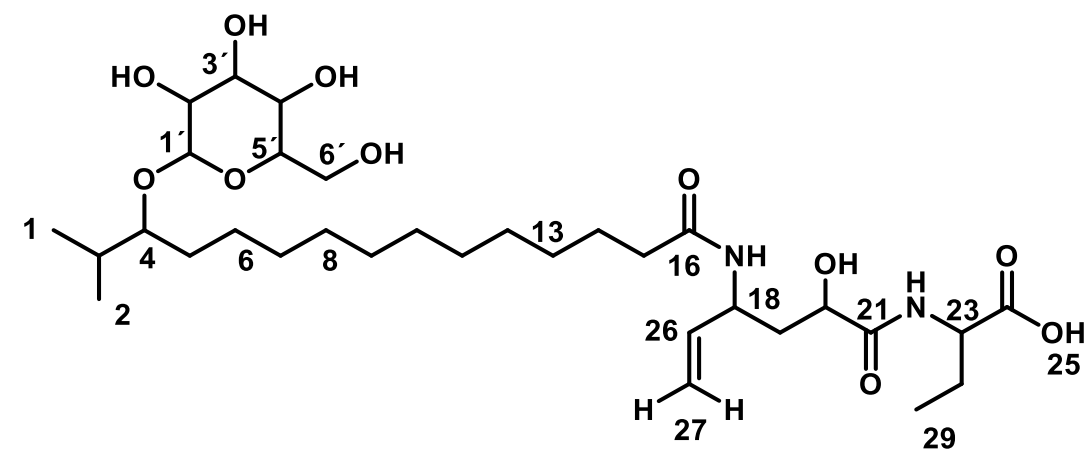

Myxoglucamide B

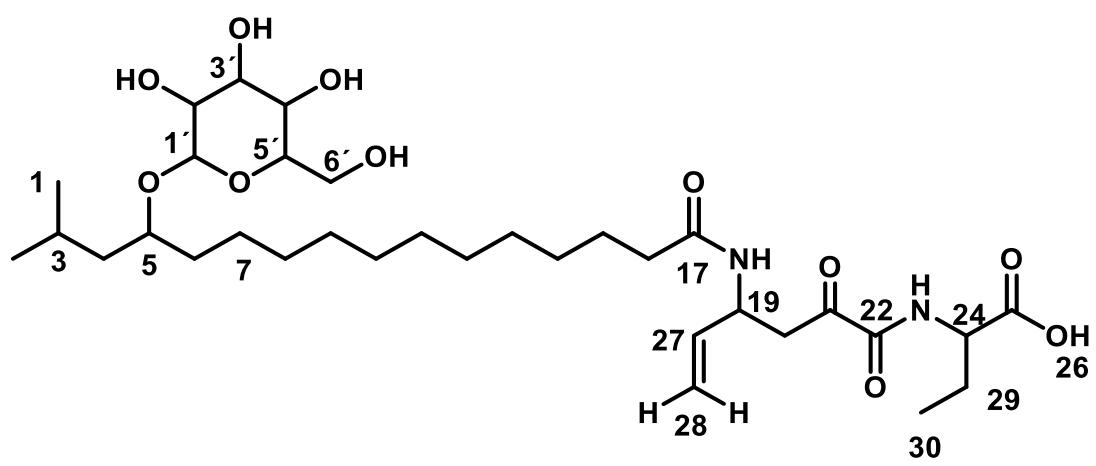

Myxoglucamide C

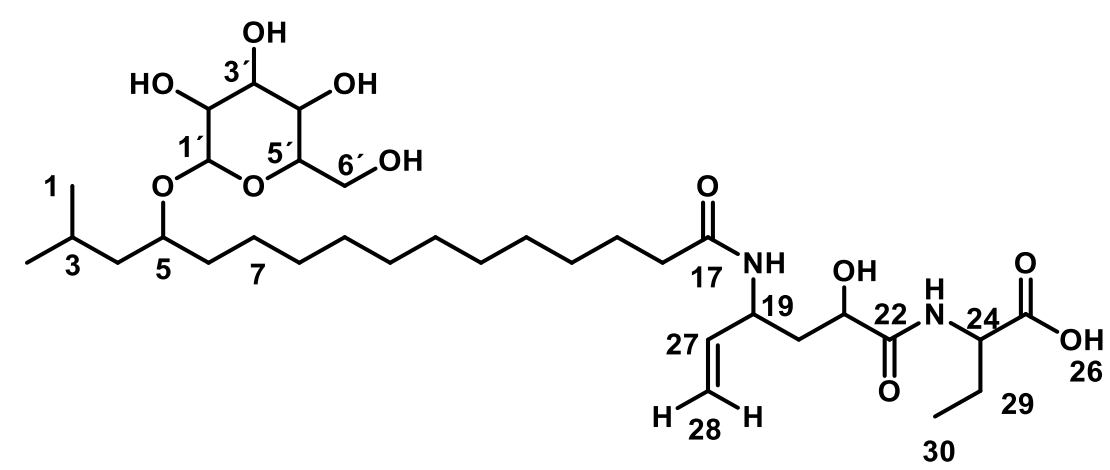

Myxoglucamide D

Table S2. Spectroscopic data of myxoglucamide A acquired in CD_3OD

position	δ_C[a]	δ_H[b]	Multiplicity (J in Hz)	COSY[c]	HMBC[d]
1	18.5	0.94	m	3	2, 3, 4
2	18.1	0.93	m	3	1, 3, 4
3	32.0	1.92	m	1, 2, 4	1, 4
4	85.2	3.49	m	3, 5	1', 1, 2, 3, 5, 6
5	31.9	1.5	m	4	4, [e]
6	26.6	1.37	m	[e]	[e]
7-13	29.9-30.4	1.29-1.32		[e]	[e]
14	27.1	1.60	m	15	16
15	37.2	2.17	t (7.7)	14	14, 16
16	175.6				
18	49.0	4.88	m	19a, 19b, 26, 27a, 27b	16, 19, 20, 26
19a	42.7	3.23	m	18, 19b	18, 20, 26
19b	42.7	2.99	dd (16.8, 8.5)	18, 19a	18, 20, 21, 26
20	196.3				
21	162.0				
23	55.5	4.29	m	28a, 28b	20, 21, 24, 28, 29

24	175.2				
26	138.5	5.86	ddd (17.2, 10.4, 5.5)	18, 27a, 27b	18, 19
27a	115.5	5.19	dt (17.2, 1.4)	18, 26, 27b	18, 26
27b	115.5	5.11	dt (10.4, 1.4)	18, 26, 27a	18, 26
28a	25.9	1.82	dt (13.9, 7.6)	23, 28b, 29	23, 24, 29
28b	25.9	1.97	m	23, 28a, 29	23, 24, 29
29	10.4	0.97	m	[e]	23
1'	103.9	4.30	d (7.7)	2'	2', 3', 4
2'	75.5	3.19	m	1', 3'	1'
3'	78.2	3.35	m	2'	1', 2', 4'
4'	71.8	3.32	m	[e]	5', 6'
5'	77.7	3.23	m	6'a, 6'b	[e]
6'a	63.0	3.85	dd (11.7, 5.5)	5', 6'b	4', 5'
6'b	63.0	3.68	dd (11.7, 2.4)	5', 6'	4', 5'

[a] acquired at 125 MHz, referenced to solvent signal CD_3OD at *δ* 49.0 ppm.

[b] acquired at 500 MHz, referenced to solvent signal CD_3OD at *δ* 3.31 ppm.

[c] proton showing COSY correlation to indicated proton.

[d] proton showing HMBC correlations to indicated carbons.

[e] too crowded to unambiguously assign signals

Table S3. Spectroscopic data of myxoglucamide B acquired in CD_3OD

position	δ_C[a]	δ_H[b]	Multiplicity (*J* in Hz)	COSY[c]	HMBC[d]
1	17.9	0.94	m	3	3, 4
2	18.1	0.92	m	3	3, 4
3	31.6	1.90	m	1, 2, 4	
4	85.1	3.50	m	3, 5	1, 3, 1'
5	31.8	1.49	m	4	7, 11
6	26.0	1.34	m	[e]	[e]
7-13	30.0-31	1.29-1.32		[e]	[e]
14	26.9	1.60	m	15	10, 12, 13, 15
15	37.0	2.18	t (7.6)	14	14, 10, 13, 12, 16
16	174.7				
18	50.5	4.59	m	19a, 19b, 26	19, 27, 26, 16
19a	39.9	1.96	br s	18, 19b, 20	20, 21, 26
19b	39.9	1.78	m	18, 19a, 20	18, 20, 21, 26
20	70.0	4.03	dd (9.7, 3.7)	19a, 19b	19, 18, 21
21	176.3				
23	54.8	4.31	m		29, 6, 24, 21
24	175.2				
26	138.2	5.79	ddd (17.2, 10.3, 6.9)	18, 27a, 27b	19, 18
27a	116.3	5.23	dt (17.2, 1.3)	26, 27b	18, 26
27b	116.3	5.15	dt (10.3, 1.2)	26, 27a	26
28a	26.0	1.93	m	28b, 29	29, 23
28b	26.0	1.78	m	28a, 29	29, 23, 24
29	9.8	0.94	m		
1'	103.7	4.30	d (7.7)	2'	5', 4
2'	75.3	3.17	dd (8.8, 7.9)	4', 3', 1'	3', 1'
3'	78.1	3.33	m	2'	4', 2'
4'	71.4	3.31	m	2', 5'	3', 6', 5'

5'	77.3	3.22	m	4', 6'a, 6'b	6', 4', 1'
6'a	62.8	3.68	dd (11.7, 5.5)	5', 6'b	4', 5'
6'b	62.8	3.85	dd (11.7, 2.4)	5', 6'a	4', 5'

[a] acquired at 175 MHz and assigned from 2D NMR spectra, referenced to solvent signal CD_3OD at *δ* 49.0 ppm.

[b] acquired at 700 MHz, referenced to solvent signal CD_3OD at *δ* 3.31 ppm.

[c] proton showing COSY correlation to indicated proton.

[d] proton showing HMBC correlations to indicated carbons.

[e] too crowded to unambiguously assign signals.

Table S4. Spectroscopic data of myxoglucamide C acquired in CD_3OD

position	δ_C[a]	δ_H[b]	Multiplicity (*J* in Hz)	COSY[c]	HMBC[d]
1, 2	23.1	0.91	d (6.7)	3	3, 4
3	25.2	1.81	m	1,2	[e]
4a	44.8	1.27	m	4b	[e]
4b	44.8	1.51	m	4a, 5	1, 2, 4, 6
5	79.0	3.74	m	4, 6	3, 4, 6, 1´
6a	36.4	1.58	m	[e]	4
6b	36.4	1.52	m	[e]	4
7	25.9	1.38	br d (6.3)	[e]	[e]
8-14	30.0-30.6	1.27-1.34		[e]	[e]
15	27.1	1.58	br d (6.3)	16	16
16	37.1	2.17	m	15	15, 17
17	175.6				
19	48.7	4.87	s	20a, 20b, 28, 27	17, 20, 21, 26, 27
20a	42.7	3.21	m	20b, 19	19, 21, 26
20b	42.7	2.99	dd (16.8, 8.4)	20a, 19	19, 21, 22, 26
21	196.3				
22	162.0				
24	55.5	4.30	m	29	22, 25
25	175.2				
27	138.5	5.86	m	19, 28a, 28b	19, 20
28a	115.5	5.11	m	27, 28b	19, 26
28b	115.5	5.19	br t (1.5)	19, 27, 28a	
29a	25.9	1.97	m		29, 24
29b	25.9	1.8	m	30, 24	29, 24, 25
30	10.4	0.95	m	29	
1'	103.9	4.29	t (7.4)	2'	3'

2'	75.3	3.15	dd (8.9, 8.0)	3', 1'	4', 3'
3'	78.2	3.33	d (6.7)	2'	4'
4'	71.8	3.30	m	5'	6', 2', 3'
5'	77.7	3.23	m	4', 6'a, 6'b	
6'a	62.9	3.68	dd (11.7, 5.5)	5', 6'b	5'
6'b	62.9	3.85	dd (11.7, 2.4)	5', 6'a	4', 5'

[a] acquired at 125 MHz referenced to solvent signal CD_3OD at δ 49.00 ppm.

[b] acquired at 500 MHz, referenced to solvent signal CD_3OD at δ 3.31 ppm.

[c] proton showing COSY correlation to indicated proton.

[d] proton showing HMBC correlations to indicated carbons.

[e] overlap with solvent signal.

[f] too crowded to unambiguously assign signals.

Table S5. Spectroscopic data of myxoglucamide D acquired in CD_3OD

position	δ_C[a]	δ_H[b]	Multiplicity (*J* in Hz)	COSY[c]	HMBC[d]
1, 2	23.2	0.91	m	3	3, 4, 5
3	25.5	1.80	m	1, 2, 4	4, 5
4a	45.0	1.28	m	3, 4b, 5	3, 6, 5
4b	45.0	1.51	m	4a, 5	3
5	79.3	3.75	m	4a, 4b	7, 6, 4, 1'
6a	36.7	1.55	m	6b	4
6b	26.1	1.38	m	6a	7, 4, 5
7	23.2	0.91	m	[f]	4
8-14	29.9-30.4	1.28-1.34			
15	27.1	1.6	m	16	16
16	37.3	2.17	m	15	12, 15, 17
17	175.2				
19	50.4	4.58	m (5.6, 6.8, 9.0)[e]	20, 20, 27	17, 20, 21, 27, 28
20a	40.2	1.96	m (3.5, 9.0, 13.7)[e]	19, 20b, 21	19, 27
20b	40.2	1.77	m (5.5, 9.3, 13.7)[e]	19, 20a, 21	19, 21, 27
21	70.2	4.04	dd (3.7, 9.6)[e]	20a, 20b	19, 20, 22
22	176.9				
24	54.7	4.33	dd (7.5, 5.0)	29, 29	30, 25, 22
25	175.4				
27	138.5	5.79	ddd (17.18, 10.28, 6.9)	19, 28a, 28b	19, 20
28a	116.7	5.23	br dt (17.0, 1.4)	27, 28b	19
28b	116.7	5.16	br dt (10.3, 1.3)	27, 28a	19, 27
29a	26.1	1.93	m	24, 29	24, 25, 30
29b	26.1	1.78	m	30, 29, 24	24, 25, 30
30	10.2	0.95	m	29	24
1'	103.9	4.29	d (7.7)	2'	3', 5
2'	75.3	3.15	dd (9.0, 7.9)	3', 1'	3', 1'
3'	78.2	3.33	m	2'	4', 4', 2', 1'
4'	71.7	3.3	m	5'	6', 3'
5'	77.7	3.23	ddd (9.4, 5.5, 2.4)	4', 6', 6'	6', 4', 2', 3', 1'
6'	62.9	3.85	dd (11.8, 2.4)	5', 6'	4', 5'
6'	62.9	3.68	m	5', 6'	4', 5'

[a] acquired at 175 MHz, referenced to solvent signal CD_3OD at *δ* 49.00 ppm.
[b] acquired at 700 MHz, referenced to solvent signal CD_3OD at *δ* 3.31 ppm.
[c] proton showing COSY correlation to indicated proton.
[d] proton showing HMBC correlations to indicated carbons.
[e] coupling constant values obtained from *J* resolved COSY experiment. [f] too crowded to unambiguously assign signals.

Table S6. Marfey´s analysis of myxoglucamides compared to the L-Abu/D-Abu standards

	m/z	RT D-FDLA [min]	RT L-FDLA [min]
L-Abu	398.17	18.16	15.09
D-Abu	398.17	15.10	18.17
myxoglucamide A	398.17	18.16	15.09
myxoglucamide B	398.17	18.16	15.06
myxoglucamide D	398.17	18.16	15.08
myxoglucamide C	398.17	18.16	15.09

Table S7. Marfey's analysis of myxoglucamides after ozonolysis compared to the L-Asp standards

	m/z	RT D-FDLA [min]	RT L-FDLA [min]
L-Asp	428.14	13.22	12.42
myxoglucamide A	428.14	13.28	12.49
myxoglucamide B	428.14	13.23	12.45
myxoglucamide D	428.14	13.25	12.45
myxoglucamide C	428.14	13.47	12.70

Table S8. Obtained coupling constants for the *J*-based analysis

Segment C19-C20				Segment C20-C21			
^3J H19,Ha20	9.0 Hz		Large	^3J H21,Ha20	3.7 Hz		Small
^3J H19,Hb20	5.6 Hz		medium	^3J H21,Hb20	9.6 Hz		large
^3J H19,C21	2.7 Hz[a]	/ 1.3 Hz[b]	Small	^3J H21,C19	3.6 Hz[a]	/4.0 Hz[b]	Small
^3J H20a,C27	4.2 Hz[a]	/ 3.9 Hz[b]	medium	^3J H20a,C22	3.2 Hz[b]		medium
^3J H20b,C27	4.2 Hz[a]		medium	^3J H20b,C22	n.d.		
^2J H20a,C19	4.6 Hz[a]	/ 4.3 Hz[b]	large	^2J H20a,C21	2.2 Hz[a]	/2.3 Hz[b]	small
^2J H20b,C19	4.8 Hz[a]		large	^2J H20b,C21	6.1 Hz[a]		large

[a] obtained from a dipsi 2 NMR experiment.

[b] obtained from a HSQMBC NMR experiment.

Table S9. Spectroscopic data of *n*-hexane extract from hydrolysis mixture of myxoglucamide A in $CDCl_3$ containing HMPA

position	δ_C[a]	δ_H[b] (*J* in Hz)
1	17.3	0.92 d (6.7)
2	19.0	0.91 d (6.1)
3	33.6	1.64 m
4	77.0	3.37 d,d,d (8.6, 5.3, 3.1)
5	34.3	1.45 m
6		1.37 m
7-13	28.7-30.1	1.18-1.34
14	24.8	1.35 m
15	33.6	2.34 t (7.3)
16	176.6	

[a] acquired at 175 MHz and assigned from 2D NMR spectra, referenced to solvent signal $CDCl_3$ at *δ* 77.16 ppm.

[b] acquired at 700 MHz, referenced to solvent signal $CDCl_3$ at *δ* 7.26 ppm.

Table S10. Chemical shifts of the β-Glucose in the methanol extract from the acidic hydrolysis of myxoglucamide A obtained in D_2O

position	δ_C[a]		δ_H[b] (*J* in Hz)	
	aqueous extract	D-glucose (β-form)	aqueous extract	D-glucose(β-form)
1	98.7	98.7	4.59 d 8.0	
2	76.9	76.9	3.19 dd 9.1, 8.0	3.16 dd 8.9, 8.5
3	78.5	78.5	3.42 m	3.40 m
4	72.3	72.3	3.35 m	3.32 m
5	78.7	78.7	3.41 m	3.39 m
6a	63.5	63.5	3.84 dd 2.3, 12.2	3.82 dd 12.0, 2.0
6b			3.66 m	3.65 m

[a] acquired at 175 MHz in D_2O, referenced to referenced to anomeric proton of β-Glucose at δ 98.66. Reference value obtained Human metabolome database.

[b] acquired at 700 MHz and referenced to solvent signal at δ 4.79.

Table S11. Spectroscopic data of both Mosher esters of the HMPA chain in pyridine-d_5

	(*S*)-MTPA ester		(*R*)-MTPA Ester		$\Delta\delta_{(S-R)}$
Position	δ_H[b]	δ_C[a]	δ_H[b]	δ_C[a]	
1	0.926	17.5	0.855	17.2	0.071
2	0.914	18.6	0.829	18.4	0.085
3	1.933	31.3	1.899	31.2	0.034
4	5.163	82.2	5.170	82.1	-0.007
5a	1.628	30.7	1.705	31.2	-0.077
5b	1.506		1.610		-0.104
6	1.397	25.9	1.372	25.9	0.025

[a] acquired at 175 MHz in pyridine-d_5 and referenced to solvent signal at δ 123.87.

[b] acquired at 700 MHz in pyridine-d_5 and referenced to solvent signal at δ 7.22.

Table S12. Spectroscopic data of *n*-hexane extract from hydrolysis mixture of myxoglucamide C in $CDCl_3$ containing HMHA

position	δ_C[a]	δ_H[b] (*J* in Hz)
1	22.2	0.89 m
2	23.5	0.90 m
3	26.6	1.75 m
4	36.7	1.23 m
		1.37 m
5	70.3	3.68 m
6	37.9	1.42 m
		1.44 m
7		1.64 m
8-14	28.1-30.0	1.19-1.36 m
15	24.7	1.62 m
16	33.4	2.33 t (7.4)
17	175.0	

[a] acquired at 175 MHz and assigned from 2D NMR spectra, referenced to solvent signal $CDCl_3$ at *δ* 77.16 ppm.
[b] acquired at 700 MHz, referenced to solvent signal $CDCl_3$at *δ* 7.26 ppm.

Table S13. Chemical shifts of the β-Glucose in the methanol extract from the acidic hydrolysis of myxoglucamide C obtained in D_2O

position	δ_C[a]		δ_H[b] j (Hz)	
	aqueous extract	D -glucose (β-form)	aqueous extract	D -glucose(β-form)
1	98.7	98.7	4.59 d 8.0	
2	76.9	76.9	3.19 dd 9.1,8.0	3.16 dd 8.9, 8.5
3	78.5	78.5	3.42 m	3.40 m
4	72.3	72.3	3.35 m	3.32 m
5	78.7	78.7	3.41 m	3.39 m
6a	63.5	63.5	3.84 dd 2.3,12.2	3.82 dd 12.0,2.0
6b			3.66 m	3.65 m

[a] acquired at 175 MHz in D_2O, referenced to referenced to anomeric proton of β-Glucose at δ 98.66. Reference value obtained Human metabolome database.
[b] acquired at 700 MHz and referenced to solvent signal at δ 4.79.

Table S14. Spectroscopic data of both Mosher esters of the HMHA chain in pyridine-d_5

Position	(S)-MTPA Ester		(R)-MTPA Ester		$\Delta\delta_{(S-R)}$
	δ_H[b]	δ_C[a]	δ_H[b]	δ_C[a]	
1	0.94	22.3	0.88	21.9	0.06
2	0.91	23.0	0.82	23.0	0.09
3	1.71	25.0	1.54	24.5	0.17
4a	1.70	43.1	1.63	42.8	0.07
4b	1.41		1.34		0.07
5	5.40	76.3	5.40	76.2	
6a	1.64	34.3	1.72	34.6	-0.10
6b	1.59		1.67		-0.08
7	1.24	25.2	1.40	25.5	-0.16

[a] acquired at 175 MHz in pyridine d_5 and referenced to solvent signal at δ 123.87.

[b] acquired at 700 MHz in pyridine d_5 and referenced to solvent signal at δ 7.22.

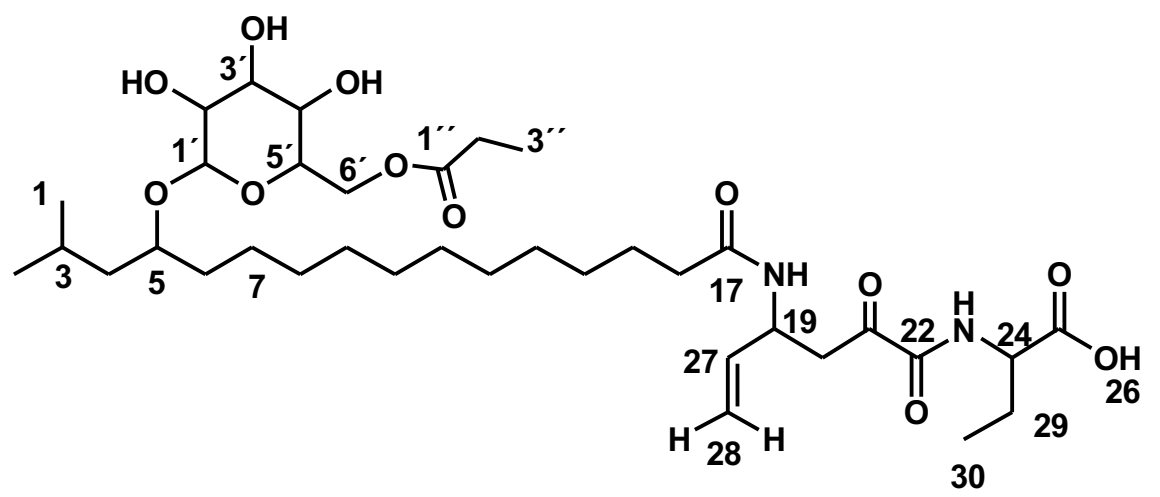

Myxoglucamide E

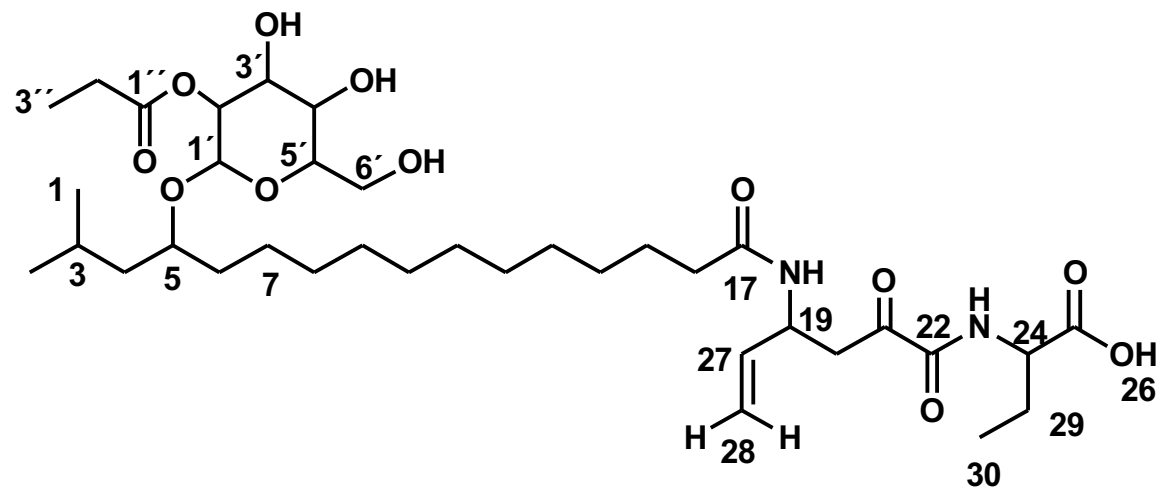

Myxoglucamide F

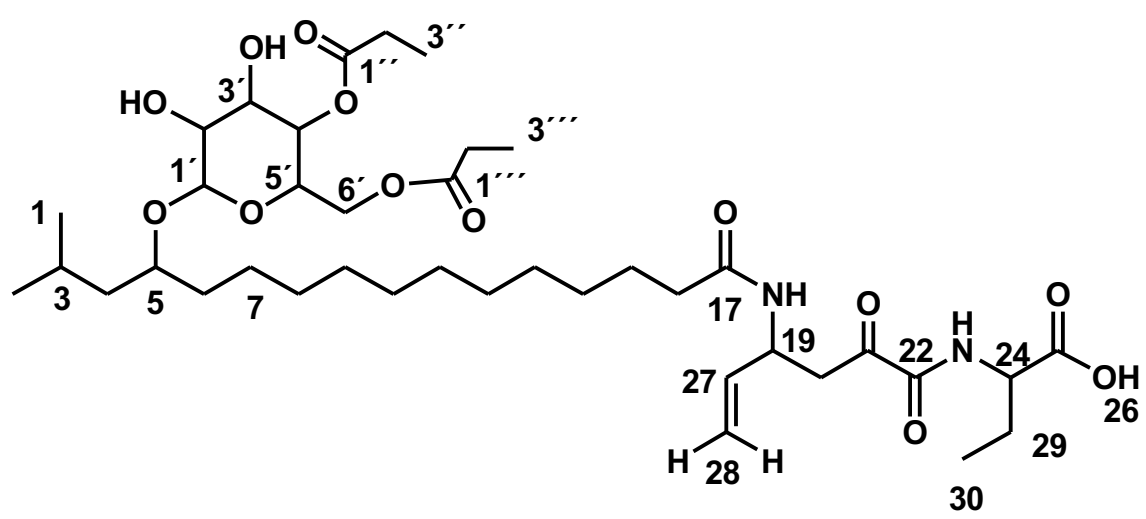

Myxoglucamide G

Table S15. Spectroscopic data myxoglucamide E acquired in CD_3OD

position	δC[a]	δH[b]	Multiplicity (*J* in Hz)	COSY[c]	HMBC[d]
1, 2	22.9	0.91	m	3	3, 4
3	25.1	1.79	tt (14.4, 7.1)	1, 2	1, 2, 4
4a	44.9	1.50	m	[e]	[e]
4b	44.9	1.26	m	[e]	1, 2, 3, 6
5	78.9	3.69	m	[e]	3, 1'
6	36.2	1.52	m	7	3, 5, 8-14
7	25.8	1.35	m	6	8-14
8-14	30.4	1.29	m	15	
15	26.7	1.59	m	8- 14, 16	
16	36.8	2.17	t (7.6)	15	8-14, 15, 17
17	175.2				
19	48.3	4.86	m	20, 27	20
20a	42.0	3.22	m	20b	19, 21
20b	42.0	3.01	dd (17.1, 8.0)	19, 20a	19, 21
21	196.4				
22	161				
24	56.7	4.18	m	[e]	22, 25, 29, 30
25	177.2				
27	138.3	5.86	m	5a, 5b, 19	19, 20
5a	115.0	5.10	m	5b, 27	19
5b	115.0	5.18	m	5a, 27	19
29a	26.3	1.92	m	29b, 30	25

29b	26.3	1.77	m	29a, 30	24, 25, 30
30	9.8	0.89	m	29a, 29b	[e]
1'	103.5	4.28	d (7.7)	2'	5
2'	75	3.16	br dd (9.1, 7.8)	3', 1'	[e]
3'	77.7	3.33	dt (3.3, 1.6)	2'	[e]
4'	71.5	3.27	m	5'	3',6'
5'	74.8	3.42	m	4', 6'	[e]
6'a	64.6	4.16	m	5', 6'b	1''
6'b	64.6	4.41	dd (11.8, 2.0)	6'a	1''
1''	175.5				
2''	28.0	2.36	m	3''	1'', 3''
3''	9.1	1.13	t (7.6)	2''	1'', 2''

[a] acquired at 175 MHz and assigned from 2D NMR spectra, referenced to solvent signal CD_3OD at *δ* 49.0 ppm.
[b] acquired at 700 MHz, referenced to solvent signal CD_3OD at *δ* 3.31 ppm.
[c] proton showing COSY correlation to indicated proton.
[d] proton showing HMBC correlations to indicated carbons.
[e] too crowded to unambiguously assign signals.

Table S16. Spectroscopic data myxoglucamide F acquired in CD_3OD

position	δ_C[a]	δ_H[b]	Multiplicity (*J* in Hz)	COSY[c]	HMBC[d]
1, 2	23.3	0.88	m	[e]	[e]
3	25.0	1.68	br dd (13.8, 6.9)	4a, 4b	[e]
4a	44.7	1.18	m	3, 4b, 5	3, 5
4b	44.7	1.39	m	3, 4a, 5	3, 5
5	80.1	3.66	m	4a, 4b	1´
6a	36.2	1.50	m	[e]	[e]
6b	36.2	1.63	m	[e]	[e]
7	25.6	1.32	m	[e]	[e]
8-14	30.4	1.29	m	15	[e]
15	26.7	1.59	m	8-14, 16	8-14
16	36.8	2.17	br t (7.5)	15	15, 17
17	175.2				
19	48.2	4.86	m	20a, 20b, 27	
20a	42.1	3.00	br dd (17.1, 8.4)	19, 20b	21, 27
20b	42.1	3.22	br dd (17.1, 5.5)	19, 20a	19, 21, 27
21	196.3				
22	160.9				
24	56.8	4.18	m	29a, 29b	22, 25, 29, 30
25	177.5				
27	138.3	5.86	m	5a, 5b, 19	19
28a	115.0	5.10	m	27, 28b	
28b	115.0	5.17	m	27, 28a	19, 27
29a	26.3	1.77	m	24, 29b, 30	[e]
29b	26.3	1.91	m	24, 29a, 30	25
30	9.9	0.90	m	29a, 29b	24
1'	102	4.47	d (8.0)	2'	5
2'	75.1	4.70	br dd (9.5, 8.1)	1', 3'	1'', 3'
3'	76.0	3.49	br t (9.3)	2', 4'	2'
4'	71.3	3.39	m	3', 5'	3', 5', 6'
5'	77.5	3.28	dt (3.3, 1.6)	4', 6'a, 6'b	[e]
6'a	62.3	3.70	m	5', 6'b	4', 5'
6'b	62.3	3.85	br dd (11.8, 2.3)	5', 6'a	4'
1''	174.7				
2''a	28.0	2.42	m	2''b, 3''	1'', 3''
2''b	28.0	2.33	m	3'', 2''a	1'', 3''
3''	9.0	1.13	t (7.51)	2''a, 2''b	1'', 2''

[a] acquired at 125 MHz and assigned from 2D NMR spectra, referenced to solvent signal CD_3OD at δ 49.0 ppm.

[b] acquired at 500 MHz, referenced to solvent signal CD_3OD at δ 3.31 ppm.

[c] proton showing COSY correlation to indicated proton.

[d] proton showing HMBC correlations to indicated carbons.

[e] too crowded to unambiguously assign signals.

Table S17. Spectroscopic data myxoglucamide G acquired in CD_3OD

position	δC[a]	δH[b]	Multiplicity (*J* in Hz)	COSY[c]	HMBC[d]
1, 2	22.9	0.91	m	[e]	3, 4
3	25.1	1.79	m	[e]	
4a	44.9	1.26	m	4b	1, 2, 3
4b	44.9	1.51	m	4a	1, 2, 3, 5
5	79.2	3.70	m	6	4, 6, 7, 1'
6	36.2	1.53	m	5, 7	[e]
7	25.8	1.37	m	6	[e]
8-14	30.4	1.29	m	15	[e]
15	26.7	1.59	m	8-14	8-14
17	175.3				
16	36.8	2.17	t (7.5)	15	15, 17
19	48.4	4.86	m	20a, 20b	
20a	42.1	3.21	m	19, 20b	21
20b	42.1	3.00	dd (17.1, 8.3)	19, 20a	21, 19
21	196.3				
22	160.9				
24	56.9	4.17	m	29	22, 25, 29, 30
25	177.4				
27	138.3	5.86	m	28a, 28b	19
28a	115.0	5.09	dt (10.4, 1.3)	27, 28b	
28b	115.0	5.18	m	27, 28a	19, 27
29a	26.3	1.77	m	24, 29b	
29b	26.3	1.92	m	29a, 30	
30	9.8	0.89	m	29	24, 29
1'	103.5	4.34	d (7.8)	2'	[e]
2'	75.1	3.25	m	1', 3'	1'
3'	75.4	3.53	m	2', 4'	4'
4'	71.8	4.82	m	3', 5'	1'', 3', 5', 6'
5'	72.7	3.62	ddd (10.0, 5.5, 2.3)	4', 6'a, 6'b	[e]
6'a	63.5	4.16	m	5', 6'b	[e]
6'b	63.5	4.07	m	6'a	1'''
1''	174.9				
2''	28.0	2.39	m	3''	1'', 3''
3''	9.0	1.12	m	2''	1'', 2''
1'''	175.3				
2'''	28.1	2.41	m	3'''	1'''
3'''	9.0	1.14	m	2'''	1''', 2'''

[a] acquired at 175 MHz and assigned from 2D NMR spectra, referenced to solvent signal CD_3OD at *δ* 49.0 ppm.

[b] acquired at 700 MHz, referenced to solvent signal CD_3OD at *δ* 3.31 ppm.

[c] proton showing COSY correlation to indicated proton.

[d] proton showing HMBC correlations to indicated carbons.

[e] too crowded to unambiguously assign signals.

Table S18. Proposed acylated product species from *in vitro O*-acylation assays using fatty acyl-CoAs of different chain lengths or their mixtures

Provided CoA(s)	Product species	Proposed acyl composition	Cal. *m/z*	Obs. *m/z*
acetyl CoA (C2:0)	mono-acylated	C2	701.42	701.42, 743.43, 785.45
	di-acylated	C2 + C2	743.43	
	tri-acylated	C2 + C2 + C2	785.44	
n-propionyl CoA (C3:0)	mono-acylated	C3	715.44	715.44, 771.46, 827.49
	di-acylated	C3 + C3	771.46	
	tri-acylated	C3 + C3 + C3	827.49	
n-butyryl CoA (C4:0)	mono-acylated	C4	729.45	729.45, 799.50
	di-acylated	C4 + C4	799.50	
	tri-acylated	C4 + C4 + C4	869.54	
acetyl CoA (C2:0), *n*-propionyl CoA (C3:0)	mono-acylated	C2	701.42	701.42, 715.44, 743.43, 757.45, 771.47, 785.45, 813.48, 827.49
		C3	715.44	
	di-acylated	C2 + C2	743.43	
		C2 + C3	757.45	
		C3 + C3	771.46	
	tri-acylated	C2 + C2 + C2	785.44	
		C2 + C2 + C3	799.46	
		C2 + C3 + C3	813.47	
		C3 + C3 + C3	827.49	
acetyl CoA (C2:0), n-butyryl CoA (C4:0)	mono-acylated	C2	701.42	701.42, 729.45, 743.43, 771.46, 785.45, 799.50, 813.48, 841.51
		C4	729.45	
	di-acylated	C2 + C2	743.43	
		C2 + C4	771.46	
		C4 + C4	799.50	
	tri-acylated	C2 + C2 + C2	785.44	
		C2 + C2 + C4	813.47	
		C2 + C4 + C4	841.51	
		C4 + C4 + C4	869.54	
n-propionyl CoA (C3:0), n-butyryl CoA (C4:0)	mono-acylated	C3	715.44	715.44, 729.45, 771.46, 785.48, 799.50, 855.52
		C4	729.45	
	di-acylated	C3 + C3	771.46	
		C3 + C4	785.48	
		C4 + C4	799.50	
	tri-acylated	C3 + C3 + C3	827.49	
		C3 + C3 + C4	841.51	
		C3 + C4 + C4	855.52	
		C4 + C4 + C4	869.54	
acetyl CoA (C2:0), *n*-propionyl CoA (C3:0), *n*-butyryl CoA (C4:0)	mono-acylated	C2	701.42	701.42, 715.44, 729.45, 743.43, 757.45, 771.46,
		C3	715.44	
		C4	729.45	
		C2 + C2	743.43	

	di-acylated	C2 + C3	757.45	785.48, 799.50, 813.48, 827.49, 841.51, 855.52
		C2 + C4	771.46	
		C3 + C3	771.46	
		C3 + C4	785.48	
		C4 + C4	799.50	
	tri-acylated	C2 + C2 + C2	785.44	
		C2 + C2 + C3	799.46	
		C2 + C2 + C4	813.47	
		C2 + C3 + C3	813.47	
		C2 + C3 + C4	827.49	
		C2 + C4 + C4	841.51	
		C3 + C3 + C3	827.49	
		C3 + C3 + C4	841.51	
		C3 + C4 + C4	855.52	
		C4 + C4 + C4	869.54	

Table S19. A domain substrate specificity prediction

Module	Putative binding-pocket constituents										Predicted amino acid*	Actual amino acid
	235	236	239	278	299	301	322	330	331	517		
A_{MxgB}	D	A	K	D	L	G	V	V	D	K	Glu	Glu
A_{MxgD}	D	T	F	W	L	G	G	T	F	K	Val	2-aminobutyric acid

*The amino acid was predicted according to the alignment for Stachelhaus-code(s) using antismash.

Table S20. Spectroscopic data of intermediate **8** acquired in CD_3OD

position	δ_C[a]	δ_H[b]	Multiplicity (*J* in Hz)	COSY[c]	HMBC[d]
1, 2	23.1	0.91	m	3	3, 4
3	25.5	1.80	m	1, 2	1, 2
4a	45.0	1.28	m	4b, 5	3, 5, 6
4b	45.0	1.52	m	4a, 5	3
5	79.3	3.75	m	4a, 4b	1', 4, 6, 7
6	35.2	1.27	m		4
7	26.3	1.39	m	[f]	
8-13	30.5-30.9	1.29-1.39		[f]	
14	30.4	1.39	m	15	
15	27.2	1.68	m	14, 16	16
16	37.3	2.32	t (7.5)	15	14, 15, 17
17	176.0				
19	135.5				
20a	122.1	5.83	t (8.1)	21	19, 21, 22, 27
21	35.2	3.26	m	20	19, 20, 22
22	172.8				
24	57.4	4.18	m	29a, 29b	29, 30
25	[g]				
27	130.3	6.66	dd (17.0, 10.8)	28a, 28b	19, 20, 28
28a	116.3	5.38	d (17.0)	27, 28b	19, 20, 27
28b	116.3	5.25	d (10.8)	27, 28a	
29a	26.5	1.88	m	24, 29b, 30	24, 30
29b	26.5	1.70	m	24, 29a, 30	24, 30
30	10.6	0.93	m	29	24
1'	103.9/103.3[h]	4.29/4.31[h]	d (7.6)	2'	3', 5
2'	75.3	3.16	m	1', 3'	1', 3'
3'	78.2	3.35	m	2'	1', 2', 4'
4'	71.7	3.30	m	[f]	3', 6'
5'	77.7	3.24	m	4', 6'	1', 4', 6'
6a'	62.9	3.85	dd (11.7, 2.3)	5', 6b'	4', 5'
6b'	62.9	3.68	m	5', 6a'	4', 5'

[a] acquired at 175 MHz referenced to solvent signal CD_3OD at δ 49.00 ppm.
[b] acquired at 700 MHz, referenced to solvent signal CD_3OD at δ 3.31 ppm.
[c] proton showing COSY correlation to indicated proton.
[d] proton showing HMBC correlations to indicated carbons.
[e] overlap with solvent signal.
[f] too crowded to unambiguously assign signals.
[g] signal not assigned.
[h] signal of isomers.

Table S21. Primers used in this study

Primer name	Sequence (5'-3')
001bgc18-NdeI-F	ATATATCATATGAGCCCCAACCTGATCGCG
001bgc18-EcoRI-R	TATATGAATTCTTGCCTCCGGTGGTGATGCA
tn5-HindIII-F	ATATAAAGCTTGCCCTTTGGACAGCAAGCGAAC
tn5-R	ATATAGAATTCATGCTGTACCTCCTTATCCTGTCTCTTGATC
M13-21F	TGTAAAACGACGGCCAGT
M13-24R	CGGATAACAATTTCACACAGG
g001-bgc18-F	GTCGATCTGCCAGAAGAGA
g001-bgc18-R	CGACGATGCGCAATTCATG
Mx8-cos-F	GAAAGGCTCAGTCGAAAGACTGGGCCTTTCGTTTTAATCTGGACAGCAAG CGAACCGGA
Mx8-cos-R	ATTCCGCGAGAGAGCCTAAGTCGTCCGTGAGTGCCTTGCCCCACTGCAA GCTACCTGCT
MxgH-ampF	ACGACTCCGGACTCGGTGAAGCAACAGGCTCGGCACCCATACTAGTGCG CGGAACCCCTATTTGT
MxgA-ampR	CCGCCCGATGCCGGGCCAGCGCGATCAGGTTGGGGCTCATACTAGTAAA GTATATATGAGTAAAC
MxgH-Tn5-F	GCAACAGGCTCGGCACCCATTGCTGTACCTCCTTATCCTG
MxgA-van-R	GCGATCAGGTTGGGGCTCATATGCGTTTCCTCGCATCGTG
Mx8-attB-up2	GCGCACTGGACCATCACGTC
Mx8-attB-down	CGGATAGCTCAGCGGTAGAG
Mx8-attP-up2	CGACGGTGCCGACAAATAC
Mx8-attP-down	GGCTTGTGCCAGTCAACTGCG
mxg_veri_4F	AAGGCCACCAAGGAGCAACT
mxg_veri_4R	GATGTGGTGATAGCTCCAGA
mxg_veri_5F	CTGGGAGTGCTCCTGGAAGA
mxg_veri_5R	TAGGTGTGCAGCATCACTGT
MxgF-KO-F	GCACGGGCCTCCCTGAACGCCCCTTCACCCGCGCGGCGGGGGTTCATG TGCAGCTCCATC
MxgF-KO-Rnew	TCGATGATGCGCACCGCCTGACCGACCGGCTCGGGCGGTGTCAGCCAAT CGACTGGCGAG

MxgFKO-verF	AGACTCCTCGCGGTCGCA
MxgFKO-verR	TGCGTACCGCCTGACCGA
orf4KO-F	CTGCAGCCACACCCCGGCGGCCCCCACGCCCTCTCCGGAAGGTTCATGT GCAGCTCCATC
orf4KO-R	GCCGGGCACCACGCGGTCGTCGTGGTCCGCCGTGGTGACCTCAGCCAA TCGACTGGCGAG
orf4KO-verF	ATCCCATGAGGACCCGTC
orf4KO-verR	CGCCGCCGTGAACTTGAA
orf5-KO-F	CATGACGGCGCCCGTCCCGAGCCACTCGGAGAGAGCTTCCGGTTCATGT GCAGCTCCATC
orf5-KO-R	AGAGCTCTGGGTCATGGGTTCGACTCGGACGGGTAGGAGGTCAGCCAAT CGACTGGCGAG
orf5-KO-verF	AGTAGCGACGAGACGGGC
orf5-KO-verR	ACCAGTGGCCCTTCGGAC
MxgG-KO-F	GAACCCGGACCACGTGAAGCACGTCCTGGTGGAGCACGTCTCAGCCAAT CGACTGGCGAG
MxgG-KO-R	AGCACCTGGGCCACTTCGGTGCGAGCCCGCTCCTCGACGTCACGCTCAG TGGAACGAGGT
MxgGKO-verF	GTAACACCACGGTGCGCG
MxgGKO-verR	TGACCCAGGGCGAGATGG
MxgH-KO-F	CGGCGTGCCCATGCACCTGGACCCACTCACGTCGTCCTGAGGTTCATGT GCAGCTCCATC
MxgH-KO-R	ATGGGTGCCGAGCCTGTTGCTTCACCGAGTCCGGAGTCGTTCAGCCAAT CGACTGGCGAG
MxgHKO-verF	TGCCCATGCACCTGGACC
MxgHKO-verR	TCGCAGCGCATCGCCTTC
MxgA-KO-F	GATCCAATAGGTCGCCGAACCACGATGCGAGGAAACGCATGGTTCATGT GCAGCTCCATC
MxgA-KO-R	TGGGGAAGGGGGCCGTCACTCGGGCTGGGACACGGCGTCCTCAGCCAA TCGACTGGCGAG
MxgAKO-verF	AGCCGACCGACTGAGACG
MxgAKO-verR	ACACGGCGGGAATCTCGG

MxgEKO-F	TCCCGAGGCGCCGGAAGCCCGGCCCGGCTGCCTGCATTGGGGTTCATG TGCAGCTCCATC
MxgEKO-R	GGTGGATGCCGAAGTCGATGAAGCAGGCCAGCTCGTCGGCTCAGCCAAT CGACTGGCGAG
MxgEKO-verF	TGTGTACCGGCTCTCGCC
MxgEKO-verR	TCAGCTGCACGAGGTGCG
orf6KO-F	GGGACTTGTCGCGGCGTCCGCGGAGGCCGCCTGTCAGGACGGTTCATG TGCAGCTCCATC
orf6KO-R	TGACGTACGTGTCCGTCTGCAGCCCGAAGTGGCCCGTGCTTCAGCCAAT CGACTGGCGAG
orf6KO-verF	AGGAAGGAGTCCGTCCCA
orf6KO-verR	CTGGATGAGGTCCACCTG
orf7KO-F	GGAGGCGCGGCTGGCCTCTCGGCCGCCGCGGACGTTCGACGGTTCATG TGCAGCTCCATC
orf7KO-R	GCGCGCTCCCGCCGAGCAGCTCCGCCACGCCGGGCACCTCTCAGCCAA TCGACTGGCGAG
orf7KO-verF	AGTAGCGCCTTCGAGGAC
orf7KO-verR	TGCCCGTGGAGACGTTGT
orf9KO-F	TCCCGGCACACCCGGCGGGCAGGTAGGCTGCCCGACACTGGGTTCATG TGCAGCTCCATC
orf9KO-R	CTCGCCAGTCGATTGGCTGACGCACGGCGCCTGTCAGGAATTTCCCGGC GCGCGCGTCAG
orf9KO-verF	TACGGTCAGCCACGCGAC
orf9KO-verR	TACCAGAGCGTGTCGGGC
orf10KO-F	CCACTCGGGCAGCACCCTTTTCCCTTCCCTCCGCCCCACCGGTTCATGT GCAGCTCCATC
orf10KO-R	GCTGGCGCGAGCGTCAGGCGGTACGGACATTCCCCCGCGTTCAGCCAAT CGACTGGCGAG
orf10KO-verF	ACGGCTTCTCCACTCGGG
orf10KO-verR	TCTGTACGGGATGCGGCG
His-MbtH-F	CACATCACCACCACCATCACATGGCGGACGAGCGCGAAGA
His-MbtH-R	CGTCGTCATCATTCGAACCGTTAGTTCTTCAGCCCCGCCT
Histev-MxgA-F	GAAAACCTGTATTTTCAGGGCAGCCCCAACCTGATCGCGCT

Histev-MxgA-R	CTCGAGTGCGGCCGCAAGCTTCACTCGGGCTGGGACACGG
Histev-FAAL-ACP_{MxgA}-F	GAAAACCTGTATTTTCAGGGCAGCCCCAACCTGATCGCGCT
Histev-FAAL-ACP_{MxgA}-R	CGAGCCGCTTCCCCGAGGATGAGCTTGCGGCCGCACTCGAGC
Histev-Ox_{MxgA}-F	GAAAACCTGTATTTTCAGGGCTCCCGAGCGGAGTGGGTCC
Histev-Ox_{MxgA}-R	GCTCGAGTGCGGCCGCAAGCTCACTCGGGCTGGGACACGG
Histev-MxgB-F	GAAAACCTGTATTTTCAGGGCACGGCCCCCTTCCCCACCGA
Histev-MxgB-R	CTCGAGTGCGGCCGCAAGCTTCATGGCTTCCCTCGTCCTG
Histev-A_{MxgB}-F	GAAAACCTGTATTTTCAGGGCTCCCTGGAAGCGCTGCCGCT
Histev-A_{MxgB}-R	TCGAGTGCGGCCGCAAGCTAGACTTGCAGGGCCTCGCGCC
Histev-9003mbtH-F	GAAAACCTGTATTTTCAGGGCGCGGACGAGCGCGAAGACA
Histev-9003mbtH-R	TGCTCGAGTGCGGCCGCAAGTTAGTTCTTCAGCCCCGCCT
Histev-PCP_{MxgB}-F	GAAAACCTGTATTTTCAGGGCGCGCTTCCCGAGGCCGCGCC
Histev-PCP_{MxgB}-R	CGAGTGCGGCCGCAAGCTCAGTGCCCCTGGGCGAGGTGG
Histev-MxgH-F	GAAAACCTGTATTTTCAGGGCGGTGCCGAGCCTGTTGCTT
Histev-MxgH-R	GCTCGAGTGCGGCCGCAAGCTCACGTCGTCCTGACGCCCT
MxgH_H141A-F2	TTC**GCG**CACGCCATCGGTGATGGTCATATG
MxgH_H141A-R1	TCACCGATGGCGTG**CGC**GAACGACAGGCCC
MxgH_H142A-F2	CAC**GCG**GCCATCGGTGATGGTCATATGGGC
MxgH_H142A-R1	ACCATCACCGATGGC**CGC**GTGGAACGACAG
MxgH_H142F-F2	CAC**TTT**GCCATCGGTGATGGTCATATGGGC
MxgH_H142F-R1	ACCATCACCGATGGC**AAA**GTGGAACGACAG
MxgH_H142K-F2	CAC**AAA**GCCATCGGTGATGGTCATATGGGC
MxgH_H142K-R1	ACCATCACCGATGGC**TTT**GTGGAACGACAG
MxgH_D146A-F2	CACCACGCCATCGGT**GCG**GGTCATATGGGC
MxgH_D146A-R1	ACC**CGC**ACCGATGGCGTGGTGGAACGACAG
MxgH_G147A-F2	CACCACGCCATCGGTGAT**GCG**CATATGGGC
MxgH_G147A-R1	**CGC**ATCACCGATGGCGTGGTGGAACGACAG
IF-MxgC-DH-Amp-ccdb-F1-PmeI	ACACCCCGGAGCGGATGGTGTTCGCCTCGGACATTGGCGATGCGCGCTCGGTTTAAACGAGACAATAACCCTGATAAA

IF-MxgC-DH-Amp-ccdb-R1-PmeI	ACGACGTCGAAGGCTCGCGTCTCGCCATCCGCGCCCGCCTGTTCGAGGAA<u>GTTTAAAC</u>TTTGTTCAAAAAAAAGCCCG
2ndMxgC-DH -F1	ACACCCCGGAGCGGATGGTGTT
IF-MxgC-DH-A-R12	ACGCTGCGCCACCGGGGGCC**CGC**GTCCTCCTCGTACTCCGCTTCG
IF-MxgC-DH-Y-R12	ACGCTGCGCCACCGGGGGCC**GTA**GTCCTCCTCGTACTCCGCTTCG
IF-MxgC-DH-F2-A-mutation	GTCCTCCTCGTACTCCGCTTCG**GCG**GGCCCCCGGTGGCGCAGCGT
IF-MxgC-DH-F2-Y-mutation	GTCCTCCTCGTACTCCGCTTCG**TAC**GGCCCCCGGTGGCGCAGCGT
2ndMxgC-DH-R2	ACGACGTCGAAGGCTCGCGTCT
IF-MxgE-Cdomain-Amp-ccdb-F1-PmeI	TCATCGACATGCCAGGGGACCACTACTCGGTGATGGTCGAGCCCCAGGTC<u>GTTTAAAC</u>GAGACAATAACCCTGATAAA
IF-MxgE-Cdomain-Amp-ccdb-R1-PmeI	TCGGTGTCGGTGGTGAGCACGCTGTCGAAGCGCGTGCCTCCTTCGGAATC<u>GTTTAAAC</u>TTTGTTCAAAAAAAAGCCCG
MxgE-CdomainAmutation-1F	TCATCGACATGCCAGGGGACCAC
IF-MxgE-Cdomain-H138A-1R	CGCCGAGGCCTCATCGAGGATGAGCGG**CGC**CCAGGAGAGGACGACCGTTC
IF-MxgE-Cdomain-H138A-F2	**GCG**CCGCTCATCCTCGATGAGGCCTCGGCGTGGCGCTGCCTCCAGGAGCTG
MxgE-CdomainAmutation-2R	TCGGTGTCGGTGGTGAGCAC
IF-MxgE-Cdomain-P139A-1R	CGCCGAGGCCTCATCGAGGATGAG**CGC**GTGCCAGGAGAGGACGACCGTTC
IF-MxgE-Cdomain-P139A-2F	CAC**GCG**CTCATCCTCGATGAGGCCTCGGCGTGGCGCTGCCTCCAGGAGCTG
IF-MxgE-Cdomain-D143A-1R	CGCCGAGGCCTC**CGC**GAGGATGAGCGGGTGCCAGGAGAGGACGACCGTTC
IF-MxgE-Cdomain-D143A-2F	CACCCGCTCATCCTC**GCG**GAGGCCTCGGCGTGGCGCTGCCTCCAGGAGCTG
MxgE-OXdomain-mutation-F1	TTCTTCGCCCAGGGCATGAAC
IF-MxgE-OXdomain-F511A-mutation-R1	GCGCTCTCGTTGGCGAAGTA**CGC**GAGGCCGAAGCCAGGCTTG
IF-MxgE-OXdomain-H548A-mutation-R1	AGGCCGCCGTGCTCGTCGAA**CGC**GCGCTCGGGCGTCCAGACGG
IF-MxgE-OXdomain-F511A-mutation-F2	CAAGCCTGGCTTCGGCCTC**GCG**TACTTCGCCAACGAGAGCGC

IF-MxgE-Oxdomain-H548A-mutation-F2	CCGTCTGGACGCCCGAGCGC**GCG**TTCGACGAGCACGGCGGCCT
MxgE-OXdomain-mutation-R2	TCAACTCTCAGCTGAGCGTC

* Mutation sites are shown in bold, and restriction enzyme sites are underlined.

Reference

[1] Maja Remškar, PhD thesis, Genome mining in myxobacteria and actinobacteria: Studies of natural products and their biosynthetic pathways, Saarland University (Germany), **2020**.

[2] F. Panter, D. Krug, S. Baumann, R. Müller, "Self-resistance guided genome mining uncovers new topoisomerase inhibitors from myxobacteria" *Chem. Sci.* **2018**, *9*, 4898–4908.

[3] J. Fu, X. Bian, S. Hu, H. Wang, F. Huang, P. M. Seibert, A. Plaza, L. Xia, R. Müller, A. F. Stewart, Y. Zhang, "Full-length RecE enhances linear-linear homologous recombination and facilitates direct cloning for bioprospecting" *Nat. Biotechnol.* **2012**, *30*, 440–446.

[4] H. Wang, Z. Li, R. Jia, Y. Hou, J. Yin, X. Bian, A. Li, R. Müller, A. F. Stewart, J. Fu, Y. Zhang, "RecET direct cloning and Redαβ recombineering of biosynthetic gene clusters, large operons or single genes for heterologous expression" *Nat. Protoc.* **2016**, *11*, 1175–1190.

[5] S. Thomas, N. D. Maynard, J. Gill, "DNA library construction using Gibson Assembly®" *Nat. Methods.* **2015**, *12*, i–ii.

[6] S. Groß, B. Schnell, P. A. Haack, D. Auerbach, R. Müller, "*In vivo* and *in vitro* reconstitution of unique key steps in cystobactamid antibiotic biosynthesis" *Nat. Commun.* **2021**, *12*, 1696.

[7] D. A. Herbst, B. Boll, G. Zocher, T. Stehle, L. Heide, "Structural basis of the interaction of MbtH-like proteins, putative regulators of nonribosomal peptide biosynthesis, with adenylating enzymes" *J. Biol. Chem.* **2013**, *288*, 1991–2003.

[8] L. J. Klau, S. Podell, K. E. Creamer, A. M. Demko, H. W. Singh, E. E. Allen, B. S. Moore, N. Ziemert, A. C. Letzel, P. R. Jensen, "The Natural Product Domain Seeker version 2 (NaPDoS2) webtool relates ketosynthase phylogeny to biosynthetic function" *J. Biol. Chem.* **2022**, *298*, 102480.

[9] H. Chen, L. Zhong, H. Zhou, T. Sun, G. Zhong, Q. Tu, Y. Zhuang, X. Bai, X. Wang, J. Xu, L. Xia, Y. Shen, Y. Zhang, X. Bian, "Biosynthesis of glidomides and elucidation of different mechanisms for formation of β-OH amino acid building blocks" *Angew. Chem. Int.l Ed.* **2022**, *61*, e202203591.

[10] C.-Y. Chang, J. R. Lohman, T. Huang, K. Michalska, L. Bigelow, J. D. Rudolf, R. Jedrzejczak, X. Yan, M. Ma, G. Babnigg, A. Joachimiak, G. N. Jr. Phillips, B. Shen, "Structural insights into the free-standing condensation enzyme SgcC5 catalyzing ester-bond formation in the biosynthesis of the enediyne antitumor antibiotic C-1027" *Biochem.* **2018**, *57*, 3278–3288.

[11] J. Vestola, T. K. Shishido, J. Jokela, D. P. Fewer, O. Aitio, P. Permi, M. Wahlsten, H. Wang, L. Rouhiainen, K. Sivonen, "Hassallidins, antifungal glycolipopeptides, are widespread among cyanobacteria and are the end-product of a nonribosomal pathway" *Pro. Nat. Acad. Sci.* **2014**, *111*, E1909–E1917.

[12] J. B. Patteson, C. M. Fortinez, A. T. Putz, J. Rodriguez-Rivas, L. H. I. Bryant, K. Adhikari, M. Weigt, T. M. Schmeing, B. Li, "Structure and function of a dehydrating condensation domain in nonribosomal peptide biosynthesis" *J. Am. Chem. Soc.* **2022**, *144*, 14057–14070.

[13] G. Bifulco, P. Dambruoso, L. Gomez-Paloma, R. Riccio, "Determination of relative configuration in organic compounds by NMR spectroscopy and computational methods" *Chem. Rev.* **2007**, *107*, 3744–3779.

[14] T. R. Hoye, C. S. Jeffrey, F. Shao, "Mosher ester analysis for the determination of absolute configuration of stereogenic (chiral) carbinol carbons" *Nat. Protoc.* **2007**, *2*, 2451–2458.

[15] K. Zaleta-Rivera, C. Xu, F. Yu, R. A. E. Butchko, R. H. Proctor, M. E. Hidalgo-Lara, A. Raza, P. H. Dussault, L. Du, "A bidomain nonribosomal peptide synthetase encoded by Fum14 catalyzes the formation of tricarballylic esters in the biosynthesis of fumonisins" *Biochem.* **2006**, *45*, 2561–2569.

[16] D. F. Kreitler, E. M. Gemmell, J. E. Schaffer, T. A. Wencewicz, A. M. Gulick, "The structural basis of N-acyl-α-amino-β-lactone formation catalyzed by a nonribosomal peptide synthetase" *Nat. Commun.* **2019**, *10*, 3432.

[17] A. Keatinge-Clay, "Crystal structure of the erythromycin polyketide synthase dehydratase" *J. Mol. Biol.* **2008**, *384*, 941–953.
